# The efficacy and safety of combining different Chinese patent medicines with conventional Western drugs in the treatment of pediatric allergic rhinitis: network meta-analysis

**DOI:** 10.3389/fphar.2025.1693357

**Published:** 2025-11-27

**Authors:** Yue Ma, Wenrui Huang, Hang Wu, Chunbo Zhang, Zhiyue Xiao, Yuying Gao, Junying Xie, Jingyao Xu

**Affiliations:** 1 Changchun University of Chinese Medicine, Changchun, Jilin, China; 2 Guangzhou University of Chinese Medicine, Guangzhou, Guangdong, China; 3 Shenzhen Traditional Chinese Medicine Hospital, Shenzhen, Guangdong, China; 4 The Third Affiliated Hospital of Changchun University of Chinese Medicine, Changchun, Jilin, China; 5 Jilin Central Hospital, Jilin, Jilin, China

**Keywords:** allergic rhinitis, Chinese patent medicines, conventional Western medical therapy, network meta-analysis, randomized controlled trials

## Abstract

**Ethnopharmacological Significance:**

Pediatric allergic rhinitis (AR) is often treated with conventional Western medical therapy (CWMT), but such regimens can cause adverse effects. Evidence suggests that Chinese patent medicines (CPMs) combined with CWMT may improve symptom control and immunological markers, yet no PRISMA-compliant network meta-analysis (NMA) has systematically compared available CPMs.

**Objective:**

This study aims to conduct an NMA of randomised controlled trials (RCTs) comparing the efficacy and safety of CPMs plus CWMT in pediatric AR.

**Methods:**

We conducted a PRISMA-guided NMA of randomized controlled trials evaluating CPMs plus CWMT versus CWMT alone for pediatric allergic rhinitis. Eight databases were searched through May 2025. Risk of bias was assessed using the Cochrane Risk of Bias 2.0 tool, and evidence certainty was graded using the Confidence in Network Meta-Analysis (CINeMA) framework. Continuous outcomes were expressed as standardized mean differences (SMDs) with 95% confidence intervals (CIs), while binary outcomes were summarized as odds ratios (ORs) or risk ratios (RRs) with corresponding 95% CIs. Analyses were performed in StataMP 18, and treatment hierarchies were ranked using the surface under the cumulative ranking curve (SUCRA) method.

**Results:**

A total of 49 RCTs involving 5,062 participants and 13 CPMs were included. Compared with CWMT alone, the combination of CPMs and CWMT significantly improved the Total Nasal Symptom Score (TNSS). Tongqiao Biyan Granules (TBG) achieved the greatest improvements across nasal obstruction (SMD = −1.79, 95% CI: −2.84 to −0.74; SUCRA 72.3%), sneezing (SMD = −2.09, 95% CI: −3.27 to −0.91; SUCRA 78.8%), and rhinorrhea (SMD = −1.88, 95% CI: −3.20 to −0.56; SUCRA 78.2%), indicating consistent superiority over other regimens, and Sanfeng Tongqiao Dropping Pills (STDP) being most effective for nasal pruritus (SMD = −1.57; SUCRA 81.9%). For overall efficacy, all CPM combinations outperformed CWMT, with Cang’er Zibi Yan Pills (CBP) achieving the highest improvement (RR = 1.25, 95% CI: 1.06–1.49; SUCRA 77.2%). Although seven CPMs showed a trend toward reduced serum IgE, none reached statistical significance; Xinqin Granules (XG) ranked highest (SUCRA 76.9%). Adverse events were generally mild and less frequent with combination therapy, with Yuping Feng Granules (YG) showing the lowest risk (OR = 0.33, 95% CI: 0.19–0.55 SUCRA 79.5%). Recurrence analysis (18 trials, 1,511 participants) indicated that most CPM combinations lowered relapse risk, with Huaiqi Huang Granules (HG) performing best (OR = 0.24, 95% CI: 0.06–0.92; SUCRA 84.8%). Sensitivity and meta-regression analyses confirmed the robustness of these findings, and all significant TNSS improvements exceeded the minimal clinically important difference (MCID = 0.55), indicating clinically meaningful symptom relief.

**Conclusion:**

Combining CPMs with CWMT may offer superior efficacy and safety for pediatric AR. These findings support CPMs as an adjunct to standard therapy, though large, high-quality RCTs are warranted for confirmation.

**Systematic Review registration:**

https://www.crd.york.ac.uk/PROSPERO/view/CRD420251080593.

## Highlights

- First PRISMA-compliant network meta-analysis (NMA) of 13 Chinese patent medicines (CPMs) combined with conventional therapy for pediatric allergic rhinitis.
- Tongqiao Biyan Granules (TBG) and Sanfeng Tongqiao Dropping Pills (STDP) show superior efficacy for specific nasal symptoms.
- Cang’er Zibi Yan Pills (CBP) ranked highest for overall efficacy, while Yuping Feng Granules (YG) showed the best safety profile.
- Most CPM combinations significantly reduced recurrence risk, with Huaiqi Huang Granules (HG) being the most effective.
- All significant symptom improvements exceeded the minimal clinically important difference, confirming clinical relevance.

## 1 Introduction

Pediatric allergic rhinitis (AR) is a chronic IgE-mediated inflammation of the nasal mucosa, marked by nasal itching, sneezing, rhinorrhea, and congestion triggered by allergen exposure. According to the World Allergy Organization (WAO) and the Allergic Rhinitis and its Impact on Asthma (ARIA) global reports, AR affects approximately 15%–20% of children worldwide, significantly reducing sleep quality, learning capacity, and overall growth and development (Pawankar, 2014). In school-aged populations, prevalence may reach 25%–40%, with 70%–90% of cases classified as moderate to severe. Around 50% of affected children also develop comorbidities such as asthma or sinusitis (Bousquet et al., 2020). Moreover, about 30% experience persistent AR, which contributes to cognitive and attention deficits (Brożek et al., 2017). Collectively, this condition poses a substantial global socioeconomic burden, as inadequate awareness and delayed diagnosis further elevate healthcare costs (Dierick et al., 2020).

Current first-line treatments for pediatric AR include pharmacotherapy and allergen-specific immunotherapy (AIT), but both approaches have notable limitations. Intranasal corticosteroids, such as fluticasone propionate, are effective in about 60%–70% of patients but may cause nasal mucosal atrophy and growth suppression with long-term use (Lourenço et al., 2020). Oral antihistamines, including cetirizine, are widely prescribed but can induce drowsiness and impair learning in approximately 30% of children (Kim et al., 2024). AIT remains the only disease-modifying option; however, its prolonged 3–5-year regimen is often discontinued by 20%–30% of patients because of systemic allergic reactions or poor adherence. High treatment costs, injection risks, and challenges associated with sublingual formulations further limit its practicality (Zhang et al., 2022). Thus, despite their proven efficacy, existing first-line therapies continue to face safety and adherence issues, underscoring the need for alternative treatments that are safer, more tolerable, and sustainable for long-term management.

In this context, traditional Chinese medicine (TCM), particularly Chinese patent medicines (CPMs), has gained growing attention for their multi-target regulatory effects and holistic therapeutic approach. CPMs such as Cang’erzi Biyan Pian (CBP) and Yupingfeng Granule (YG) demonstrate several advantages, including good safety profiles, ease of administration, and improved patient adherence, alongside notable clinical efficacy. Clinical studies have shown that YG can significantly reduce Total Nasal Symptom Score (TNSS) (Luo et al., 2017), while mechanistic studies indicate that CBP inhibits the IL-4 signaling pathway, thereby lowering serum IgE levels (Liu et al., 2020). However, existing evidence remains limited by methodological shortcomings, particularly the lack of comparative analyses among different CPMs. Previous meta-analyses have not clearly established the relative efficacy of these agents in pediatric AR, which hampers their integration into standardized treatment guidelines.

To address these gaps, the present study conducted a network meta-analysis (NMA) to systematically evaluate the relative efficacy and safety of commonly used CPMs combined with conventional Western therapies for pediatric allergic rhinitis. By integrating both direct and indirect evidence, the study aimed to establish a comparative efficacy ranking of these interventions and provide robust, evidence-based insights to inform clinical decision-making and guide individualized treatment strategies for pediatric AR.

## 2 Methods

This study adhered strictly to the PRISMA-NMA guidelines for conducting NMA (Page et al., 2021; Hutton et al., 2015). The full PRISMA checklist is provided in Supplementary Appendix S1. The study protocol has been registered with the China International Prospective Systematic Review Database (Registration No.: CRD420251080593).

### 2.1 PICOS criteria

This study included children aged 1–18 years diagnosed with AR, irrespective of sex, etiology, race, or disease severity. The control group received conventional Western medicine treatment (CWMT), which comprised intranasal corticosteroids (e.g., fluticasone propionate, budesonide, triamcinolone nasal spray), antihistamines (e.g., cetirizine oral solution, desloratadine suspension), and leukotriene receptor antagonists (e.g., montelukast chewable tablets or granules). This definition of CWMT was based on the most commonly reported pharmacotherapies in the eligible randomized controlled trials (RCTs), consistent with the core first-line recommendations of the Chinese Guidelines for Pediatric AR. Other agents such as mast cell stabilizers, decongestants, anticholinergics, or anti-IgE therapies (e.g., omalizumab) were not included, as they were rarely reported in RCTs combining CPMs with CWMT, and their inclusion would have disrupted network connectivity and increased heterogeneity. The intervention group received CWMT in combination with various CPMs, including YG, Xinqin Granules (XG), CBP, and others.

The primary outcomes were as follows: (1)TNSS (nasal congestion, nasal itching, sneezing, and rhinorrhea); (2) serum IgE levels; (3) efficacy rate, defined as the proportion of cured or markedly effective cases; and (4) adverse events (AEs). Eligible study types included published RCTs—both blinded and open-label—reporting complete patient data in either Chinese or English.

### 2.2 Literature Search method

Two researchers independently conducted a comprehensive search of both Chinese and English-language databases, including international sources such as PubMed, Cochrane Library, Embase, and Web of Science, as well as Chinese databases including China National Knowledge Infrastructure (CNKI), VIP platform, Wanfang Data Knowledge Service Platform, and the Chinese Biomedical Literature Database (CBM). The search was conducted up to May 2025. In addition to database searches, relevant studies were supplemented by reviewing academic conference proceedings, tracking references of key articles, and consulting experts in the field. The search process adhered strictly to the PICOS (Population, Intervention, Comparison, Outcome, Study design) framework. Search terms such as “allergic rhinitis,” “Chinese patent medicine,” and “randomized controlled trial” were used in both Chinese and English, combining subject headings and free-text terms for systematic retrieval. Detailed search strategies are provided in the supplementary materials (Supplementary Appendix S2).

### 2.3 Literature screening and data collection

The literature screening process was conducted in three stages—initial screening, secondary screening, and final confirmation. EndNote software was used for automatic deduplication, followed by manual verification. Independent reviewers screened the studies according to predefined inclusion and exclusion criteria, excluding those that did not meet the requirements. Full-text assessments were then performed to remove studies with incomplete data or interventions inconsistent with the research objectives. Any disagreements were resolved through discussion or consultation with a third expert. Data were extracted using a standardized form that included key study characteristics (authors, publication year, sample size, patient age, disease duration), methodological details (randomization, allocation concealment, blinding, outcome reporting), intervention information (treatment regimens and duration), and primary outcomes such as clinical efficacy, serum IgE levels, TNSS, and adverse events.

### 2.4 Risk of bias assessment

The latest version of the Cochrane Risk of Bias tool (RoB 2.0) was employed to assess the quality of the included studies (Sterne et al., 2019). This tool evaluates the risk of bias across five domains: random sequence generation, implementation of interventions, completeness of data, outcome measurement, and selective reporting of results. Each study was systematically assessed for potential biases in these areas: low risk (compliant in all domains), moderate risk (minor flaws), and high risk (major methodological flaws). Discrepancies were resolved through independent evaluation and consensus. The quality of the evidence was assessed using the CINeMA framework (Nikolakopoulou et al., 2020), which evaluates factors such as within-study bias, reporting bias, indirectness, imprecision, heterogeneity, and inconsistency.

### 2.5 Statistical analysis plan

Data integration and visualization were conducted using StataMP 18 statistical software. A frequentist statistical framework was employed, with the maximum likelihood estimation method applied to calculate treatment effects. Standard errors (SE) were used to assess the precision of parameter estimates. For different types of study outcomes:Binary Variables: Relative risk (RR) and odds ratio (OR), accompanied by their 95% confidence intervals (CI), were used. Continuous Variables: The standardized mean difference (SMD) and its 95% confidence interval were applied. Heterogeneity was assessed using the τ^2^ statistic, with the following classification standards:Low heterogeneity: τ^2^ < 0.04. Moderate-low heterogeneity: 0.04 ≤ τ^2^ < 0.16. Moderate-high heterogeneity: 0.16 ≤ τ^2^ < 0.36. High heterogeneity: τ^2^ ≥ 0.36. Comparisons between treatment regimens were performed using the multiple treatment comparison model, and network diagrams were utilized to visualize the relationships among different interventions. Treatment effect rankings were determined by calculating the surface under the cumulative ranking curve (SUCRA) value, which ranges from 0 to 1; higher values indicate superior efficacy. To assess potential publication bias, visual inspection of funnel plots was performed.

To evaluate the robustness of the findings, sensitivity analyses were performed by sequentially excluding studies with high risk of bias or small sample sizes. The impact of potential effect modifiers—including patient age, dosage form, mechanistic category, and type of CWMT—was explored using meta-regression analyses. In addition, subgroup analyses were conducted based on treatment duration (short-term ≤4 weeks, medium-term 6–8 weeks, and long-term ≥12 weeks) and TCM syndrome pattern (External Pathogen Invading the Lung and Wei Qi Deficiency and Instability and Deficiency of Vital Qi), to examine potential sources of clinical heterogeneity and align with the principle of syndrome differentiation (bianzheng lunzhi) in traditional Chinese medicine.

## 3 Results

### 3.1 Literature Search results

**FIGURE 1:**
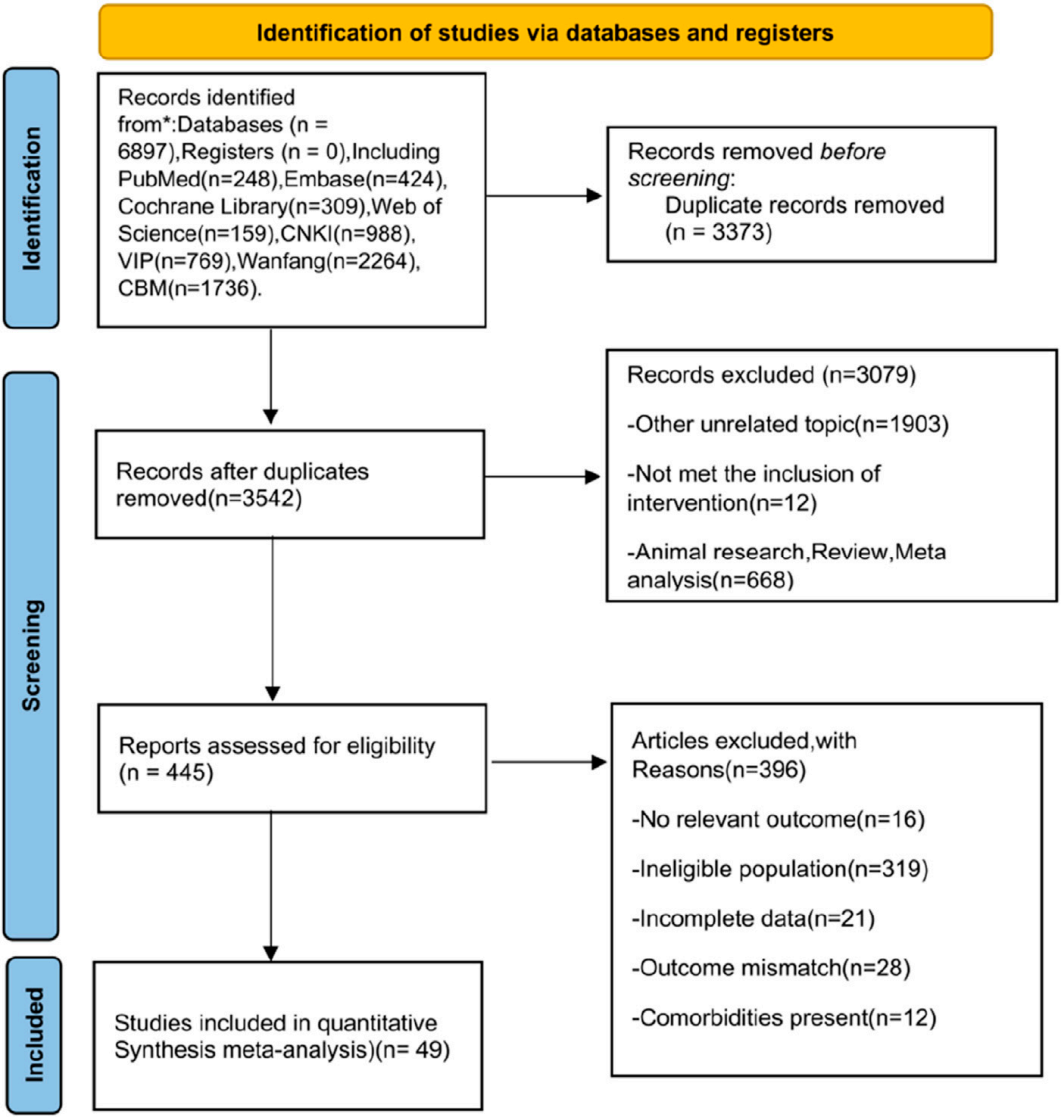
Flowchart of literature search and selection process.

A total of 6,897 records were initially retrieved for this study. After de-duplication using EndNote, 3,542 records remained for title and abstract screening. Of these, 3,079 records were excluded for failing to meet the inclusion criteria. A total of 445 studies were assessed in full, with 396 excluded (380 due to irrelevant interventions and 16 due to missing outcome measures). Ultimately, 49 studies were included in the meta-analysis (Figure 1).

### 3.2 Characteristics of included studies

This NMA included 49 RCTs (Bai, 2019; Cai et al., 2025; Cao, 2014; Chen and Zhao, 2016; Chen, 2023; Chen et al., 2022; Chen, 2017; Chen and Huang, 2008; Chen, 2024; Cui, 2016; Dong et al., 2023; Fan et al., 2017; Fan, 2013; Fang, 2020; Fang et al., 2017; Fu and Qian, 2023; Huang, 2018; Jiang et al., 2018; Jiang et al., 2023; Li et al., 2010; Lin, 2019; Lin, 2021; Liu, 2023; Lü, 2024; Ma, 2020; Sun et al., 2022; Sun et al., 2020; Tu et al., 2020; Wang and Zhao, 2021; Wang et al., 2023; Wang et al., 2019; Wang, 2017; Wang, 2019; Wu et al., 2018; Xie et al., 2018; Xu, 2017; Xu, 2014; Yang and Zhong, 2021; Yang et al., 2019; Yang and Peng, 2018; Ye et al., 2014; Yi and Wen, 2020; Yu et al., 2017; Yu et al., 2015; Yu et al., 2019; Zhang and Jiang, 2013; Zhang, 2012; Zheng, 2023; Zhu, 2023), published from the inception of the databases through May 2025, with a total of 5,062 participants. The control group received CWMT, while the treatment group received CWMT combined with CPMs, such as Nasal Comfort Tablets (NCT), Biyuan Shu Oral Liquid (BYSOL), Biyuan Tongqiao Granules (BYTG), Buzhong Yiqi Pills (BZYQP), Danxi Yuping Feng Granules (DYG), Huaiqi Huang Granules (HG), Lianhua Qingwen Granules (LQG), Xiangju Capsules (XC), Tongqiao Biyan Granules (TBG), CBP, Sanfeng Tongqiao Dropping Pills (STDP), XG, and YG. The patients’ ages ranged from 1 to 18 years, and the sample sizes for individual studies varied between 40 and 320 participants, with treatment durations ranging from 10 days to 3 months. Detailed study information is provided in Supplementary Appendix S3, Supplementary Table S3.1. The CPMs were classified into three categories based on their mechanisms of action:Nasal-specific formulations (NCT, BYSOL, CBP, BYTG, STDP, TBG, XC, XG): These primarily include *Xanthium strumarium* L., *Magnolia liliiflora* Desr., and *Scutellaria baicalensis* Georgi, focusing on promoting nasal passage and expelling wind-heat. Immunomodulatory formulas (YG, DYG, HG, BZYQP): Composed mainly of *Astragalus membranaceus* (Fisch.) Bunge, *Atractylodes macrocephala* Koidz., and *Saposhnikovia divaricata* (Turcz.) Schischk., aimed at tonifying Qi, strengthening the exterior, and modulating immune function. Heat-clearing and detoxifying formulas (LQG): These contain *Forsythia suspensa* (Thunb.) Vahl and *Lonicera japonica* Thunb., with functions to clear heat, detoxify, and promote lung function. Plant names were verified using Real-World Flora Online (www.worldfloraonline.org) or MPNS (http://mpns.kew.org). The characteristics, formulations, manufacturer details, and regulatory information (including approval and batch numbers) of the included Chinese proprietary medicines are summarized in Supplementary Appendix S3, Supplementary Tables S3.2, S3.3.

### 3.3 Methodological quality assessment results

The distribution of bias risk across the included studies is summarized as follows (detailed data can be found in Supplementary Appendix S5): Random Sequence Generation: Forty-seven studies (95.9%) were classified as having a low risk of bias. Implementation of Interventions: Seven studies (14.3%) were rated as having a low risk of bias. Data Integrity: Forty-six studies (93.9%) were rated as having a low risk of bias. Outcome Measurement and Reporting: Thirty-one studies (63.3%) were rated as having a low risk of bias. The primary methodological issues identified were: insufficient reporting of blinding procedures (including blinding of participants, researchers, and outcome assessors) and incomplete reporting of dropout data. Overall Quality Assessment:Low-risk studies: six studies (12.2%). Medium-risk studies: 23 studies (46.9%). High-risk studies: 20 studies (40.8%). The consistency of the evidence was analyzed through both direct and indirect evidence consistency testing, which revealed that most comparisons did not exhibit significant statistical heterogeneity (P > 0.05). The analysis of the τ^2^ value suggested that overall network heterogeneity was at a low to moderate level (see Supplementary Appendix S6). All network comparisons adhered to the transitivity assumption. For the evaluation of evidence certainty, the CINeMA framework was employed, which indicated:High-confidence results: Only a few pairwise comparisons. Moderate to low-confidence results: These constituted the majority of the comparisons (see Supplementary Appendix S10). Publication Bias Assessment: Funnel plot symmetry testing did not reveal significant bias (see Supplementary Appendix S11).

### 3.4 TNSS

**FIGURE 2:**
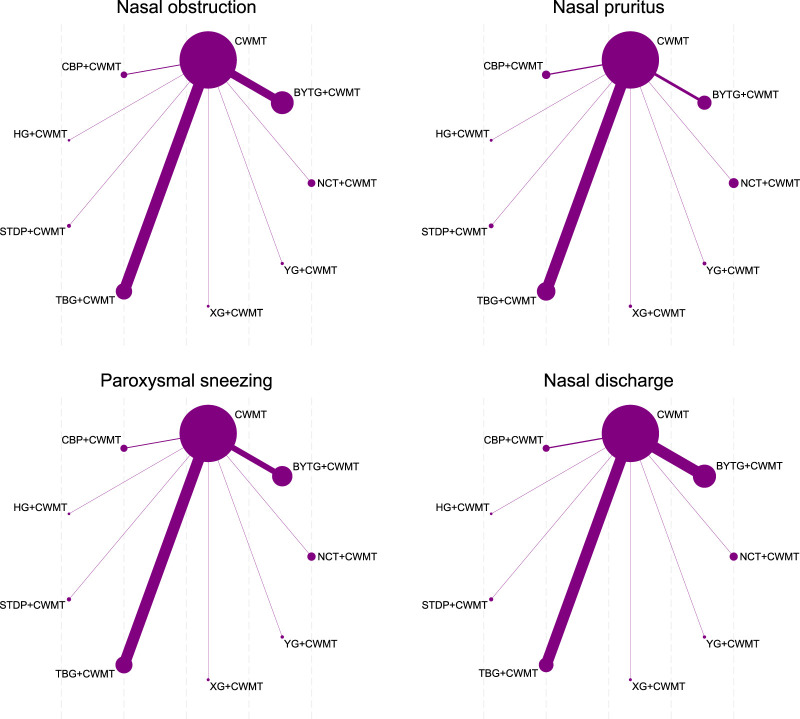
Network plot of direct comparisons for TNSS. Note: Network of available comparisons of CPMs + CWMT and CWMT for pediatric AR. The size of the nodes is proportional to the number of trial participants, and the thickness of the line connecting the nodes is proportional to the randomised number of trial participants directly comparing the two treatments.

**FIGURE 3:**
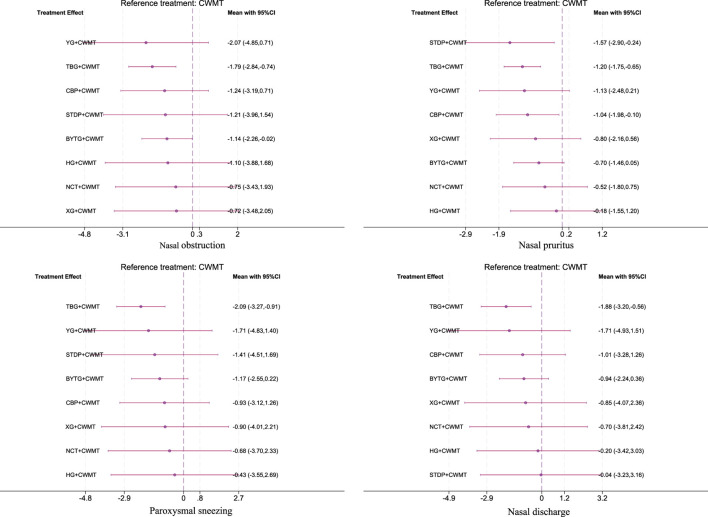
Forest plot of direct comparisons for TNSS. Note: Forest plot of network effect sizes between CPMs + CWMT and CWMT for TNSS.

For the NMA of TNSS, the core symptoms include nasal obstruction, nasal pruritus, paroxysmal sneezing and nasal discharge. Nasal obstruction: TBG + CWMT was the most effective treatment (SMD = −1.79, 95% CI: −2.84 to −0.74; SUCRA 72.3%; low confidence of evidence), followed by BYTG + CWMT (SMD = −1.14, 95% CI: −2.26 to −0.02; SUCRA 51.8%; low confidence of evidence). Nasal pruritus: STDP + CWMT (SMD = −1.57, 95% CI: −2.90 to −0.24; SUCRA 81.9%; low confidence of evidence), TBG + CWMT (SMD = −1.20, 95% CI: −1.75 to −0.65; SUCRA 71.8%; low confidence of evidence) and CBP + CWMT (SMD = −1.04, 95% CI: −1.98 to −0.10; SUCRA 62.5%; low confidence of evidence) significantly improved symptoms. Paroxysmal sneezing: TBG + CWMT showed the most advantageous effect (SMD = −2.09, 95% CI: −3.27 to −0.91; SUCRA 78.8%; low confidence of evidence). Nasal discharge: TBG + CWMT was also the most effective in relieving rhinorrhea (SMD = −1.88, 95% CI: −3.20 to −0.56; SUCRA 78.2%; low confidence of evidence). Indirect comparisons showed no significant differences, but SUCRA rankings indicated that TBG + CWMT performed particularly well for nasal congestion, sneezing, and rhinorrhea, while STDP + CWMT was more effective for nasal itching. Detailed data can be found in Figures 2, 3; Supplementary Appendices S8–S10 (Supplementary Tables S8.1–S8.4; Supplementary Tables S9.1–S9.4; Supplementary Tables S10.2–S10.5).

### 3.5 Serum IgE levels

**FIGURE 4:**
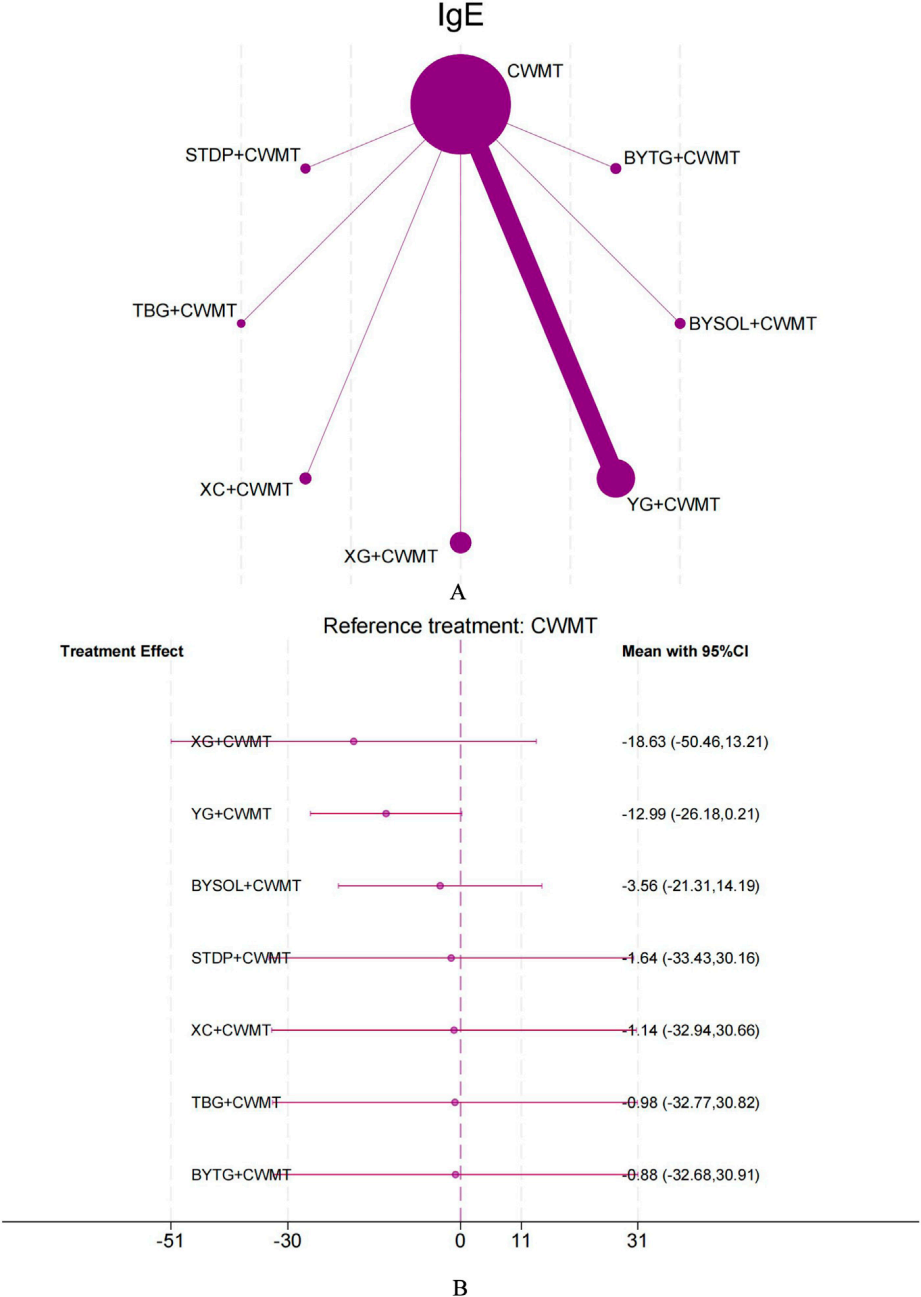
Network plot and forest plot of direct comparisons for IgE. Note: **(A)** Network of available comparisons of CPMs + CWMT and CWMT for pediatric AR. The size of the nodes is proportional to the number of trial participants, and the thickness of the line connecting the nodes is proportional to the randomised number of trial participants directly comparing the two treatments. **(B)** Forest plot of network effect sizes between CPMs + CWMT and CWMT for IgE.

The NMA study on serum IgE levels included 12 trials involving 1,202 participants. The forest plot indicated that the seven CPMs combined with CWMT showed a trend toward reducing serum IgE levels compared to the control group, but none of the results reached statistical significance. The league table revealed no significant statistical differences between indirect comparisons of various interventions. Based on SUCRA rankings, the top three interventions were XG + CWMT (SUCRA 76.9%; moderate confidence of evidence), YG + CWMT (SUCRA 73.8%; low confidence of evidence), and BYSOL + CWMT (SUCRA 46.7%; very low confidence of evidence), with TBG + CWMT ranked last (SUCRA 40.9%; very low confidence of evidence). This suggests that XG + CWMT may hold some potential for improving serum IgE levels (Figure 4; Supplementary Appendix S8; Supplementary Table S8.5; Supplementary Appendix S9; Supplementary Table S9.5; Supplementary Appendix S10; Supplementary TableS10.6).

### 3.6 Efficacy rate

**FIGURE 5:**
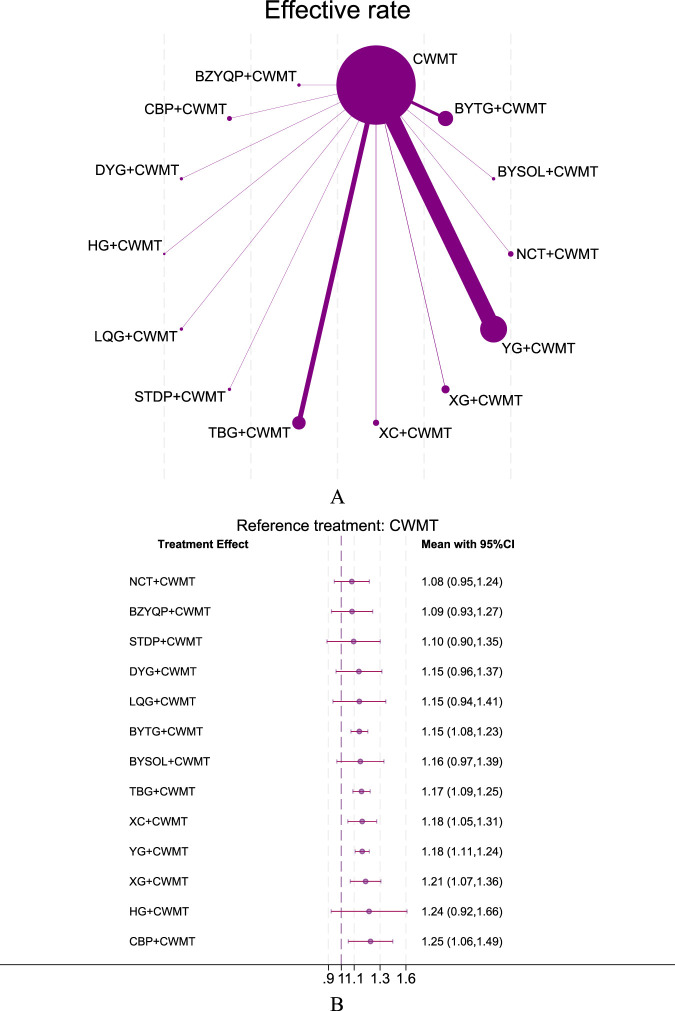
Network plot and forest plot of direct comparisons for Effective rate. Note: **(A)** Network of available comparisons of CPMs + CWMT and CWMT for pediatric AR. The size of the nodes is proportional to the number of trial participants, and the thickness of the line connecting the nodes is proportional to the randomised number of trial participants directly comparing the two treatments. **(B)** Forest plot of network effect sizes between CPMs + CWMT and CWMT for effective rate.

The NMA evaluated efficacy rates across 47 studies involving a total of 4,793 participants. Figure 5 shows direct comparisons between CPMs combined with CWMT and CWMT alone, with the thickness of connecting lines reflecting the frequency of comparisons. YG, TBG, and BYTG combined with CWMT were the most frequently compared interventions. The forest plot indicated that all 13 CPMs combined with CWMT improved efficacy rates compared to the control group, with 6 CPMs showing statistically significant results. Among these, CBP + CWMT demonstrated the greatest improvement in efficacy (RR = 1.25, 95% CI 1.06–1.49, SUCRA 77.2%, low confidence of evidence), followed by XG + CWMT (RR = 1.21, 95% CI 1.07–1.36, SUCRA 68.4%, low confidence of evidence) and YG + CWMT (RR = 1.18, 95% CI 1.11–1.24, SUCRA 60%, low confidence of evidence) (Figure 5; Supplementary Appendix S8; Supplementary Table S8.6). The league table showed no significant statistical differences between indirect comparisons of various interventions (Supplementary Appendix S9; Supplementary Table S9.6). The overall evidence quality for efficacy rates was rated as very low to moderate according to CINeMA evaluation (Supplementary Appendix S10; Supplementary Table S10.7).

### 3.7 AEs

**FIGURE 6:**
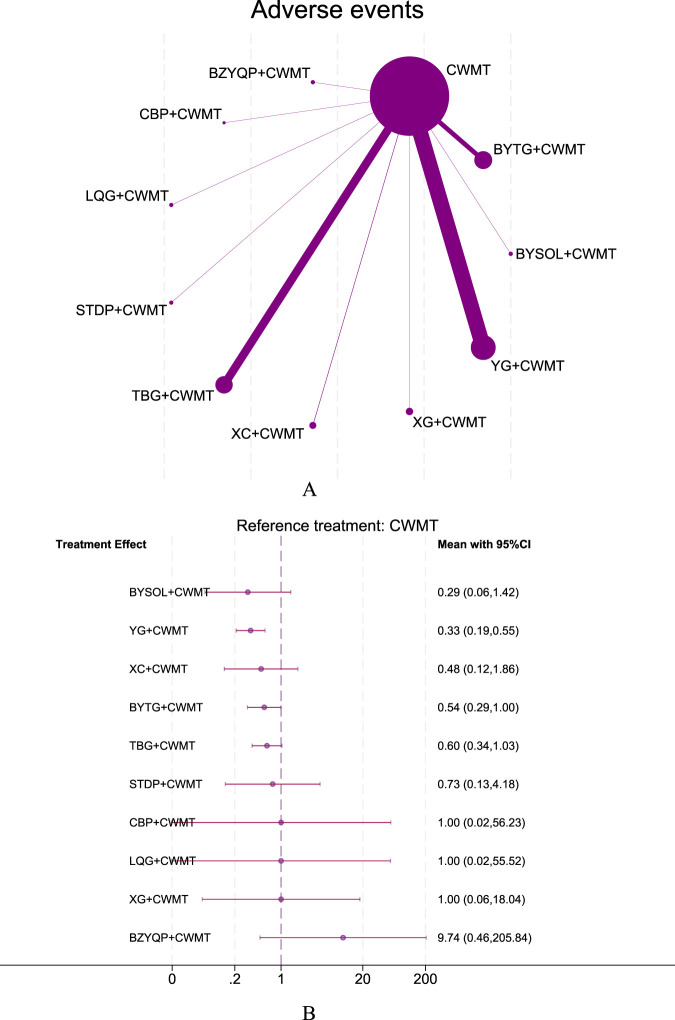
Network plot and forest plot of direct comparisons for AEs. Note: **(A)** Network of available comparisons of CPMs + CWMT and CWMT for pediatric AR. The size of the nodes is proportional to the number of trial participants, and the thickness of the line connecting the nodes is proportional to the randomised number of trial participants directly comparing the two treatments. **(B)** Forest plot of network effect sizes between CPMs + CWMT and CWMT for AEs.

The NMA also evaluated AEs associated with CPMs combined with CWMT versus CWMT alone. The forest plot revealed that 8 CPMs combined with CWMT showed a trend toward improving rhinorrhea symptoms, but only two treatments reached statistical significance: YG + CWMT (OR = 0.33, 95% CI: 0.19–0.55, moderate confidence of evidence) and BYTG + CWMT (OR = 0.54, 95% CI: 0.29–1.00, very low confidence of evidence). The league table showed that BYSOL + CWMT (OR = 0.03, 95% CI: 0.00–0.94) had significantly fewer AEs compared to BZYQP + CWMT. Based on SUCRA rankings, the top three interventions were YG + CWMT (SUCRA 79.5%), BYSOL + CWMT (SUCRA 77.1%), and XC + CWMT (SUCRA 61.8%, very low confidence of evidence), with BZYQP + CWMT ranked last (SUCRA 8.1%, very low confidence of evidence). This suggests that YG + CWMT is effective in reducing AEs (Figure 6; Supplementary Appendix S8; Supplementary Table S8.7; Supplementary Appendix S9; Supplementary Table S9.7; Supplementary Appendix S10; Supplementary Table S10.8).

Common AEs were predominantly mild to moderate and reversible. Across all studies, the most frequently reported AEs in the combination group (CPMs + CWMT) were headache (n = 20), drowsiness (n = 19), gastrointestinal discomfort (n = 14), nausea (n = 16), dry mouth (n = 14), and facial flushing (n = 16). Less frequent reactions included nasal dryness (n = 2), nasal bleeding (n = 13), dizziness (n = 7), vomiting (n = 5), and mild rash (n = 2). In comparison, the CWMT-alone group exhibited higher frequencies across most categories, including headache (n = 45), drowsiness (n = 40), gastrointestinal discomfort (n = 29), nausea (n = 32), and dry mouth (n = 24). Local nasal and skin reactions—such as nasal dryness, epistaxis, burning sensation, and rash—were also more common in the CWMT-only group (3–7 vs. 2–3 cases).

When summarized by system, gastrointestinal (nausea, vomiting, abdominal discomfort) and central nervous system AEs (headache, dizziness, drowsiness) were the most frequent, followed by mucosal reactions (nasal dryness, bleeding) and metabolic responses (dry mouth, hot flashes). Overall, CPM + CWMT regimens reduced total AE incidence by approximately 15%–30% compared with CWMT monotherapy (OR = 0.33–0.60, P < 0.05), with the lowest risk observed in YG + CWMT (OR = 0.33, 95% CI: 0.19–0.55, moderate confidence). No serious AEs or AE-related withdrawals were reported. However, potential pharmacological concerns should be noted: Xanthium strumarium–containing preparations may cause transient liver enzyme elevation, while Ephedra (Ma Huang)–based formulas can occasionally induce palpitations or mild cardiovascular stimulation. Pediatric populations require careful dosing and monitoring due to their unique pharmacokinetic and metabolic profiles. A detailed summary of AE types, frequencies, and system-based classifications is provided in Supplementary Appendix S12 (Supplementary Table S12.1).

### 3.8 Recurrence rate

The NMA included 18 RCTs involving a total of 1,511 participants to evaluate recurrence outcomes. The network diagram indicated that combinations such as YG + CWMT, BYTG + CWMT, and DYG + CWMT were the most frequently compared interventions. When compared with CWMT alone, the forest plot showed that most CPMs combined with CWMT significantly reduced the risk of recurrence. The most notable effect was observed for HG + CWMT (OR = 0.24, 95% CI: 0.06–0.92; SUCRA = 84.8%), followed by DYG + CWMT (OR = 0.36, 95% CI: 0.14–0.92; SUCRA = 72.1%) and YG + CWMT (OR = 0.42, 95% CI: 0.30–0.58; SUCRA = 66.3%). BYTG + CWMT also demonstrated a significant improvement (OR = 0.50, 95% CI: 0.29–0.84; SUCRA = 53.3%) (Supplementary Appendix S7; Supplementary Figure S7.1; Supplementary Appendix S8; Supplementary Figure S8.8; Supplementary Table S8.8; Supplementary Appendix S9; Supplementary Table S9.8). The league table revealed no statistically significant indirect differences among the included interventions. According to the CINeMA assessment, the certainty of evidence was moderate for the comparison between BYTG + CWMT and CWMT alone, while all other comparisons were rated as very low. Overall, although most CPM combinations exhibited a trend toward reduced recurrence risk compared with CWMT monotherapy, the overall strength of evidence remained limited (Supplementary Appendix S10; Supplementary Table S10.9).

### 3.9 Sensitivity analyses and meta-regressions

To assess the robustness and reliability of the results, sensitivity analyses were performed for the primary outcomes by sequentially excluding studies with a high risk of bias and those with small sample sizes, as evaluated by the Cochrane RoB 2.0 tool. After removing high-risk and small-sample trials, the pooled estimates remained stable across nasal congestion, rhinorrhea, sneezing, and overall efficacy outcomes (Supplementary Appendix S13). The SUCRA rankings of all interventions were unchanged, suggesting that the conclusions were not materially affected by study quality or sample size. Since most Chinese studies did not report prospective trial registration, sensitivity analyses excluding unregistered studies were not feasible; however, the consistency of SUCRA ranking patterns and effect sizes further supported the robustness of our findings.

The impact of potential baseline effect modifiers on the primary outcomes was assessed through meta-regression analyses. Patient age, dosage form, mechanistic category, and type of CWMT (antihistamines, intranasal corticosteroids, leukotriene receptor antagonists) were evaluated. Across nasal obstruction, rhinorrhea, sneezing, and overall efficacy, none of the coefficients reached statistical significance (all P ≥ 0.05 and 95% CIs crossed zero), indicating that neither these covariates nor the CWMT type meaningfully influenced the primary outcomes (Supplementary Appendix S14).

### 3.10 Subgroup analyses of treatment duration

The efficacy of CPMs combined with CWMT may be influenced by treatment duration. To explore this, we categorised the intervention length into short-term (≤4 weeks), medium-term (6–8 weeks), and long-term (≥12 weeks) subgroups and conducted network meta-analyses for major outcomes (Supplementary Appendix S15; Supplementary Table S15.1). Overall, the pooled estimates for nasal obstruction, rhinorrhea, sneezing, and total efficacy were comparable across duration subgroups, with largely overlapping 95% confidence intervals. No consistent duration–response pattern was observed. Although several CPMs (such as Cang’er Zibi Yan Pills and Tongqiao Biyan Granules) showed numerically greater improvements in nasal symptoms after longer treatment (≥12 weeks), the pattern was inconsistent across interventions and outcomes, and the wide CIs suggested limited precision. These findings indicate that extending treatment beyond 4 to 8 weeks did not consistently enhance efficacy, implying that treatment duration was unlikely to be a major driver of heterogeneity in this network.

### 3.11 Subgroup analyses based on syndrome pattern

Given that syndrome differentiation is a core principle in TCM, we conducted subgroup analyses according to TCM syndrome patterns. Among trials classified as External Pathogen Invading the Lung, consistent improvements were observed across all major nasal symptoms compared with CWMT alone. Combinations such as TBG + CWMT and BYTG + CWMT demonstrated the most pronounced benefits, particularly for nasal obstruction and sneezing, with mean differences ranging from approximately −1.2 to −1.8 versus CWMT. Other combinations (e.g., CBP + CWMT and STDP + CWMT) also showed favorable but statistically nonsignificant effects, likely due to limited study numbers. Importantly, the direction and magnitude of effect estimates remained stable across endpoints—including nasal obstruction, itching, sneezing, and rhinorrhea—indicating a robust and coherent therapeutic trend. Consistent findings in total response rate (odds ratios generally between 1.1 and 1.2) further support the enhanced clinical efficacy of CPM + CWMT combinations for this syndrome type (Supplementary Appendix S15; Supplementary Figure S15.1.1). In the Deficiency of Vital Qi subgroup, YG + CWMT produced the most pronounced improvement in total nasal symptom score. (Supplementary Appendix S15; Supplementary Figure S15.1.3).In the Wei Qi Deficiency and Instability subgroup, both DYG + CWMT and XG + CWMT demonstrated significant improvements in overall efficacy compared with CWMT monotherapy(Supplementary Appendix S15; Supplementary Figure S15.1.2).

## 4 Discussion

### 4.1 Summary of findings

This network meta-analysis synthesized 49 RCTs involving 5,062 children with allergic rhinitis and evaluated 13 compound CPMs in combination with CWMT. Overall, CPM + CWMT combinations achieved greater symptom improvement and safety compared with CWMT monotherapy. The most consistent benefits were observed for nasal obstruction, sneezing, and rhinorrhea, particularly with TBG and STDP based regimens. Although reductions in total serum IgE did not reach statistical significance, XG + CWMT showed a favorable trend, suggesting potential immunomodulatory effects. In contrast, CBP + CWMT demonstrated the highest efficacy ranking for overall treatment response, while YG + CWMT achieved the lowest incidence of adverse events. Importantly, recurrence analyses indicated that CPM + CWMT combinations substantially reduced relapse risk relative to CWMT alone.

Sensitivity, subgroup, and meta-regression analyses consistently confirmed the robustness of the findings. Excluding high-risk or small-sample studies did not materially alter pooled estimates or SUCRA rankings, indicating stable network performance. Neither study quality, patient age, dosage form, mechanistic category, nor CWMT type significantly influenced outcomes, and treatment duration or TCM syndrome pattern showed no systematic bias. Furthermore, when TNSS improvements were interpreted against the established minimal clinically important difference (MCID) threshold of 0.55 points (Barnes et al., 2010), all statistically significant interventions exceeded this benchmark, suggesting that the observed symptom reductions were not only statistically significant but also clinically meaningful. Overall, CPM + CWMT combinations demonstrated durable efficacy and improved tolerability for pediatric allergic rhinitis; however, given the generally low-to-moderate certainty of evidence under the CINeMA framework, these conclusions should be interpreted with caution and validated in large, well-designed multicenter RCTs.

### 4.2 Mechanisms of combining TCM and Western medicine for pediatric AR

The combination of TCM and Western medicine in treating pediatric AR works through a multi-target, synergistic regulation of the immune-inflammatory cascade, enhancing efficacy while minimizing the side effects commonly associated with Western drugs. The core mechanisms of this combined approach can be summarized across three key dimensions: Immune Homeostasis Reconstruction: YG (*Astragalus membranaceus*, *Atractylodes macrocephala*, and *Saposhnikovia divaricata*) enhances the activity of regulatory T cells (Treg), inhibits excessive IgE production, and balances the Th1/Th2 ratio. This effect, when combined with second-generation antihistamines, also reduces the incidence of sedation (Zhou et al., 2020). Inflammation and Vascular Permeability Control: XG (*Asarum heterotropoides* and *Scutellaria baicalensis*) blocks H1 receptors, showing potency comparable to cetirizine, and inhibits mast cell degranulation. When combined with nasal corticosteroids, XG also reduces the risk of nasal dryness, likely through modulation of histamine receptors and suppression of inflammatory pathways (Thangam et al., 2018). Mucosal Barrier Repair: TBG (*Xanthium strumarium* and *Magnolia liliiflora*) reduces vascular leakage by inhibiting the NF-κB pathway, thereby shortening the duration of nasal congestion (Song et al., 2024). Syndrome Differentiation: In TCM, treatment is often based on syndrome differentiation. For the lung-spleen Qi deficiency type, which accounts for 62% of pediatric AR cases, YG is the first-line treatment (Chen et al., 2023). XG is recommended for the wind-cold attacking the lungs type, and TBG is used for the lung channel heat accumulation type (Liang and Gu, 2021; Cheong et al., 2022). Safety: This combined approach minimizes the risks typically associated with Western medications, such as the central nervous system suppression of first-generation antihistamines and growth retardation caused by prolonged nasal corticosteroid use (height = −0.7 cm). However, formulations containing *X. strumarium* should be used cautiously, with treatment durations limited to ≤4 weeks. Relevant studies confirm that the integration of TCM and Western medicine provides a favorable safety profile (Zhao et al., 2025).

### 4.3 Pharmacokinetic and pharmacodynamic considerations

Herb–drug interactions may arise when CPM constituents inhibit or induce CYP/UGT pathways governing CWMT clearance (e.g., CYP3A4/2D6 for loratadine, CYP3A4 for intranasal corticosteroids, CYP2C8 ± UGT1A3 for montelukast), whereas cetirizine is largely non-CYP and thus less susceptible (Tenero et al., 2023; Cardoso et al., 2015). Baicalein/baicalin from Scutellaria can inhibit CYP3A4; honokiol from Magnoliae Flos potently inhibits CYP2C8 and UGT1A9; licorice shows context-dependent CYP3A modulation, underscoring assay-to-clinic gaps (Liu et al., 2023; Hou et al., 2012). In children, enzyme ontogeny (CYP3A7→CYP3A4 switch, age-dependent CYP “overshoot,” and delayed, isoform-specific UGT maturation) makes systemic exposure a moving target across ages, magnifying interaction uncertainty and the need for cautious co-prescribing and monitoring (Li and Lampe, 2019; de Wildt et al., 1999; Miyagi and Collier, 2007). Pragmatically, pairing CPMs with CWMT regimens less reliant on modulated pathways (e.g., cetirizine) and avoiding combinations at highest theoretical risk (e.g., CYP2C8-inhibiting components with montelukast) may mitigate interaction-driven variability pending pediatric PK/PD studies.

### 4.4 Challenges and future directions

While combining TCM and Western medicine for the treatment of pediatric AR holds substantial promise, several challenges remain, particularly regarding drug interactions, immune modulation, and the safety of child growth and development. The combination may induce complex interactions, such as metabolic conflicts through competition for P450 enzymes (e.g., Xanthium Glycoside with desloratadine) or overlapping immune pathways (e.g., Astragalus polysaccharides enhancing the Th2 inhibitory effect of antihistamines). High-quality, large-scale RCTs are essential to clarify the optimal dosage windows and long-term risks. For instance, the cumulative impact of nasal corticosteroids combined with CPMs on children’s growth and height requires further investigation. The current combination strategy enhances efficacy through a threefold synergistic mechanism: immune homeostasis regulation (YG enhances Treg activity and reduces IgE), inflammation-vascular control (XG inhibits mast cell degranulation), and mucosal barrier repair (TBG downregulates NF-κB to reduce edema). To optimize therapeutic benefit, a pediatric-specific risk-warning model should be developed, which includes:A metabolic interaction database (e.g., Total Glycosides of Paeonia Lactiflora increasing cetirizine concentration by 23%); Growth and development monitoring indicators (e.g., limiting Ma Huang-containing formulas to ≤2 weeks); A syndrome-biomarker association system (e.g., for lung and spleen Qi deficiency, using IL-13 baseline levels to guide YG dosage). Multi-center RCTs are necessary to further elucidate the mechanisms and facilitate the design of personalized treatment plans, achieving the goal of “enhancing efficacy while reducing toxicity” in pediatric AR.

### 4.5 Strengths and limitations

This study represents the most comprehensive NMA to date evaluating CPMs combined with CWMT for pediatric AR, using the CINeMA framework to enhance methodological rigor. However, several limitations should be acknowledged. Variability in baseline characteristics, treatment durations, and CPM formulations may have contributed to heterogeneity. Some included RCTs lacked details on randomization and blinding, and none were registered, which reduces transparency. Given the inherent challenges of maintaining blinding in trials involving traditional Chinese medicine—due to distinct odors, tastes, and dosage forms—it is likely that some studies achieved only partial or single blinding. This limitation may have introduced performance or detection bias, although sensitivity analyses suggested the overall results remained robust.

Furthermore, although CPMs were grouped into three mechanism-based categories to enhance pharmacological interpretability and ensure network consistency, this approach does not fully align with the TCM principle of syndrome differentiation (bianzheng lunzhi). To mitigate this limitation, syndrome-based subgroup and meta-regression analyses were conducted, showing no material effect on the overall outcomes.

Although sensitivity analyses excluding high-risk and small-sample studies confirmed the robustness of results, most trials had small sample sizes and short follow-up periods, limiting the evaluation of long-term outcomes such as sustained IgE suppression. To partially address this, we added recurrence rate as a long-term endpoint, which showed consistent advantages for CPM + CWMT combinations. Subgroup analyses by treatment duration and syndrome pattern, along with meta-regression on age, dosage form, and mechanism, did not materially change the results. Nonetheless, all included studies were derived from Chinese databases, which may introduce language and regional bias and restrict the generalizability of the findings.

## 5 Conclusion

Combining CPMs with CWMT provides superior symptom control and fewer adverse events compared with CWMT alone in pediatric allergic rhinitis. TBG + CWMT and STDP + CWMT yielded the most consistent symptom relief, while YG + CWMT showed the best safety profile. Although reductions in total IgE were not statistically significant, all TNSS improvements exceeded the minimal clinically important difference, indicating meaningful clinical benefit. Given the overall low-to-moderate evidence certainty, these findings should be interpreted cautiously. Larger, rigorously designed multicenter RCTs with standardized outcomes and long-term follow-up are warranted to confirm efficacy and safety.

## Data Availability

The original contributions presented in the study are included in the article/Supplementary Material, further inquiries can be directed to the corresponding author.

## Glossary

AR: Allergic rhinitis
WAO: the World Allergy Organization
ARIA: Allergic Rhinitis and its Impact on Asthma
AIT: allergen-specific immunotherapy
TCM: Traditional Chinese medicine
CPMs: Chinese patent medicines
CBP: Cang’er Zibi Yan Pills
YG: Yuping Feng Granules
TNSS: Total Nasal Symptom Score
NMA: Network meta-analysis
CWMT: Conventional Western medicine treatment
RCTs: Randomized controlled trials
XG: Xinqin Granules
TNSS: Total Nasal Symptom Score
AEs: adverse events
CNKI: China National Knowledge Infrastructure
CBM: China Biomedical Literature Database
SE: Standard error
RR: Relative risk
OR: Odds ratios
CI: Confidence intervals
SMD: Standardized mean differences
SUCRA: Surface under the cumulative ranking curve
NCT: Nasal Comfort Tablets
BYSOL: Biyuan Shu Oral Liquid
BYTG: Biyuan Tongqiao Granules
BZYQP: Buzhong Yi Qi Pills
DYG: Danxi Yuping Feng Granules
HG: Huaiqi Huang Granules
LQG: Lianhua Qingwen Granules
XC: Xiangju Capsules
TBG: Tongqiao Biyan Granules
STDP: Sanfeng Tongqiao Dropping Pills
