# Supplementary material for: The efficacy and safety of combining different Chinese patent medicines with conventional Western drugs in the treatment of pediatric allergic rhinitis: network meta-analysis
Source: Front Pharmacol. 2025 Nov 27;16:1693357. doi: 10.3389/fphar.2025.1693357 (PMC12695863; doi:10.3389/fphar.2025.1693357)
Supplement: Supplementary file 1 [file Supplementaryfile1.docx]

**Supplementary Appendix**

**The efficacy and safety of combining different Chinese patent medicines with conventional Western drugs in the treatment of pediatric allergic rhinitis：Network meta-analysis**

**Table of contents**

[*Appendix 1: PRISMA NMA Checklist 2*](#_Toc528349955)

[*Appendix 2: Search strategy 6*](#_Toc132813340)

[*Appendix 3: Characteristics of included studies 11*](#_Toc958296147)

[*Appendix 4: List of data extracted from the included randomized clinical trials 26*](#_Toc2103473776)

[*Appendix 5: Risk of bias of randomized clinical trials 27*](#_Toc1207956318)

[*Appendix 6: Evaluation of inconsistency and heterogeneity 30*](#_Toc1958921535)

[*Appendix 7: Network maps and forest plots of secondary outcomes 39*](#_Toc522446588)

[*Appendix 8: SUCRA and cumulative probability plots 41*](#_Toc1846655580)

[*Appendix 9:League table of summary estimates for different Chinese patent medicines combined with conventional Western medicine in treating pediatric allergic rhinitis, derived from network meta-analysis of 49 trials. 51*](#_Toc1306666616)

[*Appendix 10: CINeMA Assessment 59*](#_Toc978040890)

[*Appendix 11: Funnel plots 94*](#_Toc1093404092)

[*Appendix 12:Summary of Adverse Events 102*](#_Toc825006865)

[*Appendix 13:Sensitivity Analysis 103*](#_Toc1735955023)

[*Appendix 14:Data for regression analysis 109*](#_Toc483243419)

[*Appendix 15:Subgroup Analysis 110*](#_Toc88990179)

**Appendix 1: PRISMA NMA Checklist**

| **Section/Topic** | **Item #** | **Checklist Item** | **Reported on Page #** |
| --- | --- | --- | --- |
| **TITLE** |  |  |  |
| Title | 1 | Identify the report as a systematic review *incorporating a network meta-analysis (or related form of meta-analysis).* | 1 |
|  |  |  |  |
| **ABSTRACT** |  |  |  |
| Structured summary | 2 | Provide a structured summary including, as applicable:  **Background:** main objectives  **Methods:** data sources; study eligibility criteria, participants, and interventions; study appraisal; and *synthesis methods, such as network meta-analysis.*  **Results:** number of studies and participants identified; summary estimates with corresponding confidence/credible intervals; *treatment rankings may also be discussed. Authors may choose to summarize pairwise comparisons against a chosen treatment included in their analyses for brevity.*  **Discussion/Conclusions:** limitations; conclusions and implications of findings.  **Other:** primary source of funding; systematic review registration number with registry name. | 2 |
|  |  |  |  |
| **INTRODUCTION** |  |  |  |
| Rationale | 3 | Describe the rationale for the review in the context of what is already known*, including mention of why a network meta-analysis has been conducted.* | *4* |
| Objectives | 4 | Provide an explicit statement of questions being addressed, with reference to participants, interventions, comparisons, outcomes, and study design (PICOS). | 5 |
|  |  |  |  |
| **METHODS** |  |  |  |
| Protocol and registration | 5 | Indicate whether a review protocol exists and if and where it can be accessed (e.g., Web address); and, if available, provide registration information, including registration number. | 5 |
| Eligibility criteria | 6 | Specify study characteristics (e.g., PICOS, length of follow-up) and report characteristics (e.g., years considered, language, publication status) used as criteria for eligibility, giving rationale. *Clearly describe eligible treatments included in the treatment network, and note whether any have been clustered or merged into the same node (with justification).* | *5* |
| Information sources | 7 | Describe all information sources (e.g., databases with dates of coverage, contact with study authors to identify additional studies) in the search and date last searched. | 5 |
| Search | 8 | Present full electronic search strategy for at least one database, including any limits used, such that it could be repeated. | 5 |
| Study selection | 9 | State the process for selecting studies (i.e., screening, eligibility, included in systematic review, and, if applicable, included in the meta-analysis). | 5 |
| Data collection process | 10 | Describe method of data extraction from reports (e.g., piloted forms, independently, in duplicate) and any processes for obtaining and confirming data from investigators. | 5 |
| Data items | 11 | List and define all variables for which data were sought (e.g., PICOS, funding sources) and any assumptions and simplifications made. | 5 |
| **Geometry of the network** | **S1** | Describe methods used to explore the geometry of the treatment network under study and potential biases related to it. This should include how the evidence base has been graphically summarized for presentation, and what characteristics were compiled and used to describe the evidence base to readers. | *6* |
| Risk of bias within individual studies | 12 | Describe methods used for assessing risk of bias of individual studies (including specification of whether this was done at the study or outcome level), and how this information is to be used in any data synthesis. | 6 |
| Summary measures | 13 | State the principal summary measures (e.g., risk ratio, difference in means). *Also describe the use of additional summary measures assessed, such as treatment rankings and surface under the cumulative ranking curve (SUCRA) values, as well as modified approaches used to present summary findings from meta-analyses.* | 6 |
| Planned methods of analysis | 14 | Describe the methods of handling data and combining results of studies for each network meta-analysis. This should include, but not be limited to:   - *Handling of multi-arm trials;* - *Selection of variance structure;* - *Selection of prior distributions in Bayesian analyses; and* - *Assessment of model fit.* | 6 |
| **Assessment of Inconsistency** | **S2** | Describe the statistical methods used to evaluate the agreement of direct and indirect evidence in the treatment network(s) studied. Describe efforts taken to address its presence when found. | 6 |
| Risk of bias across studies | 15 | Specify any assessment of risk of bias that may affect the cumulative evidence (e.g., publication bias, selective reporting within studies). | 6 |
| Additional analyses | 16 | Describe methods of additional analyses if done, indicating which were pre-specified. This may include, but not be limited to, the following:   - Sensitivity or subgroup analyses; - Meta-regression analyses; - *Alternative formulations of the treatment network; and* - *Use of alternative prior distributions for Bayesian analyses (if applicable).* | *6* |
| **RESULTS†** |  |  |  |
| Study selection | 17 | Give numbers of studies screened, assessed for eligibility, and included in the review, with reasons for exclusions at each stage, ideally with a flow diagram. | 7 |
| **Presentation of network structure** | **S3** | Provide a network graph of the included studies to enable visualization of the geometry of the treatment network. | *9* |
| **Summary of network geometry** | **S4** | Provide a brief overview of characteristics of the treatment network. This may include commentary on the abundance of trials and randomized patients for the different interventions and pairwise comparisons in the network, gaps of evidence in the treatment network, and potential biases reflected by the network structure. | *9* |
| Study characteristics | 18 | For each study, present characteristics for which data were extracted (e.g., study size, PICOS, follow-up period) and provide the citations. | 9 |
| Risk of bias within studies | 19 | Present data on risk of bias of each study and, if available, any outcome level assessment. | 9 |
| Results of individual studies | 20 | For all outcomes considered (benefits or harms), present, for each study: 1) simple summary data for each intervention group, and 2) effect estimates and confidence intervals. *Modified approaches may be needed to deal with information from larger networks.* | *Appendix3* |
| Synthesis of results | 21 | Present results of each meta-analysis done, including confidence/credible intervals. *In larger networks, authors may focus on comparisons versus a particular comparator (e.g. placebo or standard care), with full findings presented in an appendix. League tables and forest plots may be considered to summarize pairwise comparisons.* If additional summary measures were explored (such as treatment rankings), these should also be presented. | 9 |
| **Exploration for inconsistency** | **S5** | Describe results from investigations of inconsistency. This may include such information as measures of model fit to compare consistency and inconsistency models, *P* values from statistical tests, or summary of inconsistency estimates from different parts of the treatment network. | *9* |
| Risk of bias across studies | 22 | Present results of any assessment of risk of bias across studies for the evidence base being studied. | 9 |
| Results of additional analyses | 23 | Give results of additional analyses, if done (e.g., sensitivity or subgroup analyses, meta-regression analyses*, alternative network geometries studied, alternative choice of prior distributions for Bayesian analyses,* and so forth). | *13* |
| **DISCUSSION** |  |  |  |
| Summary of evidence | 24 | Summarize the main findings, including the strength of evidence for each main outcome; consider their relevance to key groups (e.g., healthcare providers, users, and policy-makers). | 15 |
| Limitations | 25 | Discuss limitations at study and outcome level (e.g., risk of bias), and at review level (e.g., incomplete retrieval of identified research, reporting bias). *Comment on the validity of the assumptions, such as transitivity and consistency. Comment on any concerns regarding network geometry (e.g., avoidance of certain comparisons).* | 17 |
| Conclusions | 26 | Provide a general interpretation of the results in the context of other evidence, and implications for future research. | 17 |
| **FUNDING** |  |  |  |
| Funding | 27 | Describe sources of funding for the systematic review and other support (e.g., supply of data); role of funders for the systematic review. This should also include information regarding whether funding has been received from manufacturers of treatments in the network and/or whether some of the authors are content experts with professional conflicts of interest that could affect use of treatments in the network. | *18* |

**Appendix 2: Search strategy**

**Table S1.** Search strategy of PubMed

| **#** | **Searches** |
| --- | --- |
| **1** | "Rhinitis, Allergic"[Mesh] |
| **2** | allergic rhinitis[Title/Abstract] OR hypersensitive rhinitis[Title/Abstract] OR anaphylactic rhinitis[Title/Abstract] OR rhinitis[Title/Abstract] OR allergic rhinitis[Title/Abstract] OR allergic coryza[Title/Abstract] OR allergia rhinitis[Title/Abstract] OR allergy rhinitis[Title/Abstract] OR perennial allergic rhinitis[Title/Abstract] OR seasonal allergic rhinitis[Title/Abstract] OR allergic nasosinusitis[Title/Abstract] OR allergic caryza[Title/Abstract] OR allergic sinusitis[Title/Abstract] OR irritability rhinitis[Title/Abstract] OR irritable rhinitis[Title/Abstract] OR sinus allergy[Title/Abstract] OR anaphylaxis rhinitis[Title/Abstract] OR allergic rhinitis children[Title/Abstract] OR Allergic Rhinitides[Title/Abstract] OR Rhinitides, Allergic[Title/Abstract] OR Allergic Rhinitis[Title/Abstract] |
| **3** | #1 OR #2 |
| **4** | chinese patent medicines[Title/Abstract] OR chinese medicine[Title/Abstract] OR proprietary chinese medicine[Title/Abstract] OR chinese medicines[Title/Abstract] OR herbal medicines[Title/Abstract] OR chinese herbal medicine[Title/Abstract] OR chinese drugs[Title/Abstract] OR prepared chinese medicine[Title/Abstract] |
| **5** | #3 AND #4 |

**Table S2.** Search strategy of Web of Science: Science Citation Index Expanded

| **#** | **Searches** |
| --- | --- |
| **1** | (TI=(allergic rhinitis or hypersensitive rhinitis or anaphylactic rhinitis or rhinitis or allergic rhinitis or allergic coryza or allergia rhinitis or allergy rhinitis or perennial allergic rhinitis or seasonal allergic rhinitis or allergic nasosinusitis or allergic caryza or allergic sinusitis or irritability rhinitis or irritable rhinitis or sinus allergy or anaphylaxis rhinitis or allergic rhinitis children or Allergic Rhinitides or Rhinitides, Allergic or Allergic Rhinitis)) OR AB=(allergic rhinitis or hypersensitive rhinitis or anaphylactic rhinitis or rhinitis or allergic rhinitis or allergic coryza or allergia rhinitis or allergy rhinitis or perennial allergic rhinitis or seasonal allergic rhinitis or allergic nasosinusitis or allergic caryza or allergic sinusitis or irritability rhinitis or irritable rhinitis or sinus allergy or anaphylaxis rhinitis or allergic rhinitis children or Allergic Rhinitides or Rhinitides, Allergic or Allergic Rhinitis) and Preprint Citation Index (Exclude – Database) |
| **2** | AB=(chinese patent medicines or chinese medicine or proprietary chinese medicine or chinese medicines or herbal medicines or chinese herbal medicine or chinese drugs or prepared chinese medicine) and Preprint Citation Index (Exclude – Database) |
| **3** | (TS=(Randomized or randomized or randomized controlled or randomized controlor randomised controlled or randomized comparative or randomized study or randomly controlled or randomized comparative study or random contrast or random control or random comparison or randomly comparison or randomization or randomized comparison or randomized controll or stochastic comparison or randomly trial or randomized compare or randomized controls or randomly control or randomized comparative method or randomized trial or randomized controlling or contrast randomly or random compared or random compare or randomized contrast or randomized method or random )) OR AB=(Randomized or randomized or randomized controlled or randomized controlor randomised controlled or randomized comparative or randomized study or randomly controlled or randomized comparative study or random contrast or random control or random comparison or randomly comparison or randomization or randomized comparison or randomized controll or stochastic comparison or randomly trial or randomized compare or randomized controls or randomly control or randomized comparative method or randomized trial or randomized controlling or contrast randomly or random compared or random compare or randomized contrast or randomized method or random ) and Preprint Citation Index (Exclude – Database) |
| **4** | #1 AND #2 AND #3 and Preprint Citation Index (Exclude – Database) |

**Table S3.** Search strategy of Cochrane Central Register of Controlled Trials

(CENTRAL)

| **#** | **Searches** |
| --- | --- |
| **1** | MeSH descriptor: [Rhinitis, Allergic] explode all trees |
| **2** | (allergic rhinitis or hypersensitive rhinitis or anaphylactic rhinitis or rhinitis or allergic rhinitis or allergic coryza or allergia rhinitis or allergy rhinitis or perennial allergic rhinitis or seasonal allergic rhinitis or allergic nasosinusitis or allergic caryza or allergic sinusitis or irritability rhinitis or irritable rhinitis or sinus allergy or anaphylaxis rhinitis or allergic rhinitis children or Allergic Rhinitides or Rhinitides, Allergic or Allergic Rhinitis):ti,ab |
| **3** | #1 or #2 |
| **4** | (chinese patent medicines or chinese medicine or proprietary chinese medicine or chinese medicines or herbal medicines or chinese herbal medicine or chinese drugs or prepared chinese medicine):ti,ab,kw |
| **5** | #3 AND #4 |

**Table S4.** Search strategy of Embase

| **#** | **Searches** |
| --- | --- |
| **1** | 'allergic rhinitis'/exp |
| **2** | 'hypersensitive rhinitis':ab,ti OR 'anaphylactic rhinitis':ab,ti OR rhinitis:ab,ti OR 'allergic coryza':ab,ti OR 'allergia rhinitis':ab,ti OR 'allergy rhinitis':ab,ti OR 'perennial allergic rhinitis':ab,ti OR 'seasonal allergic rhinitis':ab,ti OR 'allergic nasosinusitis':ab,ti OR 'allergic caryza':ab,ti OR 'allergic sinusitis':ab,ti OR 'irritability rhinitis':ab,ti OR 'irritable rhinitis':ab,ti OR 'sinus allergy':ab,ti OR 'anaphylaxis rhinitis':ab,ti OR 'allergic rhinitis children':ab,ti OR 'allergic rhinitides':ab,ti OR 'rhinitides, allergic':ab,ti OR 'allergic rhinitis':ab,ti |
| **3** | #1 or #2 |
| **4** | 'chinese patent medicines':ab,ti OR 'chinese medicine':ab,ti OR 'proprietary chinese medicine':ab,ti OR 'chinese medicines':ab,ti OR 'herbal medicines':ab,ti OR 'chinese herbal medicine':ab,ti OR 'chinese drugs':ab,ti OR 'prepared chinese medicine':ab,ti |
| **5** | #3 AND #4 |

**Table S5.** Search strategy of CNKI、 VIP、Wanfang and CBM

TS = Allergic rhinitis OR allergic rhinitis OR perennial allergic rhinitis OR perennial allergic rhinitis OR perennial rhinitis OR perennial allergic rhinitis OR perennial allergic rhinitis OR allergic rhinitis OR rhinitis

TS = Chinese patent medicine OR prepared formula preparations OR granules OR tablets OR capsules OR oral liquids OR pills OR powders OR ointments

Abstract = Randomized controlled OR randomized grouping OR randomized OR RCT

**Appendix 3: Characteristics of included studies**

**Table S3.1:** Baseline of characteristics of included studies

| **Study** | **Country and region** | **Design** | **Follow-up duration** | **Number of participants** | **Randomised treatments** | **Dose and frequency(mean ± SD)** | **Allergic rhinitis duration (mean ± SD)** | **Age (mean ± SD), years** | **Outcome indicator** |
| --- | --- | --- | --- | --- | --- | --- | --- | --- | --- |
| Ma Shufang (2020). | China | RCT | 4 weeks | 240 | Nasal Comfort Tablets + Budesonide Nasal Spray 120 | Budesonide nasal spray 1 spray/nostril BID intranasal ×4 weeks Nasal Comfort Tablets 1.4 g (4 tabs) TID po ×4 weeks | 3.17±0.80 years | 8.20±1.39 | 13 |
|  |  |  |  |  | Budesonide Nasal Spray 120 |  | 3.09±0.85 years | 8.11±1.45 |  |
| Fang Chunru (2020). | China | RCT | 10 days | 108 | Biyuan Shu Oral Liquid+ Cetirizine Drops 54 | Cetirizine HCl drops(10mL:0.1g)<6y: 0.5 mL QD po≥6y: 1 mL QD po×10d Biyuan Shu Oral Liquid(10mL/支)<6y: 5 mL TID po≥6y: 10 mL TID po×10d | 4.21±0.12 days | 7.68±1.22 | 124 |
|  |  |  |  |  | Cetirizine Drops 54 |  | 4.05±0.32 days | 7.19±1.38 |  |
| Lin Xiaoyan (2019). | China | RCT | 8 weeks | 110 | Biyuan Tongqiao Granules + Mometasone Furoate Nasal Spray 55 | Mometasone furoate nasal spray (50μg/puff)2 puffs/nostril QD intranasal ×8w Biyuan Tongqiao Granules (15g/bag)15 g TID po ×8w | 14.38±4.14 weeks | 8.49±2.31 | 1234 |
|  |  |  |  |  | Mometasone Furoate Nasal Spray 55 |  | 14.17±4.23 weeks | 8.74±2.06 |  |
| Xie Liangchao (2018). | China | RCT | 2 weeks | 150 | Biyuan Tongqiao Granules + Montelukast Sodium Chewable Tablets 75 | Montelukast chewable tab 4mg QD po ×2w  Biyuan Tongqiao Granules15mg TID po (dissolve) ×2w | 7.9±1.2 weeks | 8.9±1.3 | 13 |
|  |  |  |  |  | Montelukast Sodium Chewable Tablets 75 |  | 7.8±1.1 weeks | 8.7±1.5 |  |
| Wang Ranran (2019). | China | RCT | 2 weeks | 150 | Biyuan Tongqiao Granules + Montelukast Sodium Chewable Tablets 75 | Montelukast chewable tab <6y: 4mg QD po ≥6y: 5mg QD po ×2w  Biyuan Tongqiao Granules(15g/bag) 5-15g TID po (dissolve, avoid spicy) ×2w | - | 6.97±1.69 | 134 |
|  |  |  |  |  | Montelukast Sodium Chewable Tablets 75 |  | - | 6.56±2.06 |  |
| Lin Zhihua (2021). | China | RCT | 8 weeks | 158 | Biyuan Tongqiao Granules + Pidotimod Oral Solution 79 | Pidotimod oral soln 7mL BID po ×8w  Biyuan Tongqiao Granules 3-6y: 7.5mg TID po 7-13y: 15mg TID po ×8w | 3.52±0.68 years | 7.31±1.72 | 13 |
|  |  |  |  |  | Pidotimod Oral Solution 79 |  | 3.48±0.72 years | 7.24±1.56 |  |
| Fan Jinping (2017). | China | RCT | 3 months | 86 | Biyuan Tongqiao Granules + Pidotimod Oral Solution 43 | Pidotimod oral solution(10mL:0.4g) 1 ampoule BID ×3m  Biyuan Tongqiao Granules (15g/bag) 15g TID ×3m | 7.2±2.8 weeks | 8.4±3.3 | 14 |
|  |  |  |  |  | Pidotimod Oral Solution 43 |  | 7.4±3.1 weeks | 8.3±2.8 |  |
| Yu Wangbo (2019). | China | RCT | - | 142 | Biyuan Tongqiao Granules + Pidotimod Oral Solution 71 | Biyuan Tongqiao Granules (5g/bag) 15g TID ×duration  Pidotimod oral soln (10mL:0.4g/amp) 1 amp BID ×duration | - | - | 34 |
|  |  |  |  |  | Pidotimod Oral Solution 71 |  | - | - |  |
| Sun Xinxin (2020). | China | RCT | 4 weeks | 88 | Biyuan Tongqiao Granules + Cetirizine Hydrochloride Drops 44 | Cetirizine HCl drops3-6y: 0.5mL QD 6-14y: 1mL QD ×4w  Biyuan Tongqiao Granules 3-6y: 10g BID 6-14y: 15g TID ×4w | 8.4±2.5 months | 7.2±1.7 | 14 |
|  |  |  |  |  | Cetirizine Hydrochloride Drops 44 |  | 8.2±2.7 months | 7.1±1.9 |  |
| Dong Tianjin (2023). | China | RCT | 1 month | 100 | Biyuan Tongqiao Granules + Cetirizine 50 | Cetirizine tab (10mg/tab) 10mg QD pc ×1m  Biyuan Tongqiao Granules (15g/bag) 7.5g TID po (warm water) ×1m | 7.34±3.18 weeks | 7.85±3.21 | 134 |
|  |  |  |  |  | Cetirizine 50 |  | 8.35±3.23 weeks | 8.43±2.33 |  |
| Chen Lilei (2016). | China | RCT | - | 98 | Danxi Yuping Feng Granules + Fluticasone Propionate Nasal Spray 49 | Fluticasone nasal spray (120 sprays/bottle) <12y: 1 spray/nostril QD AM (100μg) ≥12y: 2 sprays/nostril QD AM (200μg)  Danxi Yuping Feng Granules 5g TID | - | 10-15 | 1 |
|  |  |  |  |  | Fluticasone Propionate Nasal Spray 49 |  | - | 10-15 |  |
| Jiang Hua (2018). | China | RCT | 12 weeks | 140 | Cang'er Zibi Yan Pills+ Montelukast 70 | Montelukast 4mg QD ×12w  Cang'er Zibi Yan Pills14 pills TID pc ×12w | 7.03±3.14 months | 6.69±2.77 | 13 |
|  |  |  |  |  | Montelukast 70 |  | 6.26±2.72 months | 6.08±2.39 |  |
| Tu Jing (2020). | China | RCT | 6 weeks | 60 | Cang'er Zibi Yan Pills + Montelukast 30 | Montelukast 4mg QD ×6w  Cang'er Zibi Yan Pills1.2g TID ×6w | 3.51±0.60 years | 8.16±1.52 | 134 |
|  |  |  |  |  | Montelukast 30 |  | 3.47±0.56 years | 8.03±1.45 |  |
| Sun Jingyuan (2022). | China | RCT | 4 weeks | 102 | Lianhua Qingwen Granules + Desloratadine 51 | Desloratadine1-5y: 1.25mg QD 6-11y: 2.5mg QD ≥12y: 5mg QD ×4w  Lianhua Qingwen Granules 3-6y: 3g TID 7-9y: 4.5g TID ≥10y: 6g TID <3y: PRN ×4w | 33.25 ± 5.62 months | 6.94 ± 1.52 | 14 |
|  |  |  |  |  | Desloratadine 51 |  | 32.15±6.80 months | 7.04 ± 1.24 |  |
| Zhu Lang (2023). | China | RCT | 2 weeks | 48 | Huaiqi Huang Granules + Mometasone Furoate Nasal Spray + Desloratadine Dry Suspension 24 | Mometasone nasal spray 3-11y: 1 spray/nostril QD (100μg) ≥12y: 2 sprays/nostril QD (200μg)  Desloratadine susp 1-5y: 1.25mg QD 6-11y: 2.5mg QD ≥12y: 5mg QD  Huaiqi Huang Granules 3-12y: 1 bag BID | - | 6.8±1.9 | 13 |
|  |  |  |  |  | Mometasone Furoate Nasal Spray + Desloratadine Dry Suspension 24 |  | - | 7.7±1.8 |  |
| Xu Yan (2017). | China | RCT | 1 month | 68 | Tongqiao Biyan Granules + Montelukast Chewable Tablets 34 | Montelukast chewable tab 2-5y: 4mg QD HS ≥6y: 5mg QD HS ×1m  Tongqiao Biyan Granules 2-5y: 1/3 pack TID ≥6y: 1/2 pack TID ×1m | - | 5.78±1.06 | 13 |
|  |  |  |  |  | Montelukast Chewable Tablets 34 |  | - | 5.67±1.23 |  |
| Liu Zhaohong (2023). | China | RCT | 1 month | 40 | Yuping Feng Granules + Montelukast Sodium 20 | Montelukast(5mg/tab) <6y: 5mg QD ≥6y: 10mg QD ×1m  Yuping Feng Granules (5g/bag) <4y: 2.5g TID ≥4y: 5g TID ×1m | 25.23±2.54 months | 6.21±0.53 | 12 |
|  |  |  |  |  | Montelukast Sodium 20 |  | 25.39±2.62 months | 6.38±0.62 |  |
| Lv Min (2024). | China | RCT | 1 month | 96 | Yuping Feng Granules + Montelukast Sodium Tablets 48 | Montelukast <6y: 5mg QD ≥6y: 10mg QD ×1m  Yuping Feng Granules<4y: 0.5 bag TID (dissolve) ≥4y: 1 bag TID (dissolve) ×1m | 25.5±3.6 months | 6.5±1.5 | 124 |
|  |  |  |  |  | Montelukast Sodium Tablets 48 |  | 25.1±3.4 months | 6.3±1.2 |  |
| Chen Longfeng (2023). | China | RCT | 1 month | 72 | Yuping Feng Granules + Montelukast Sodium 36 | Montelukast (5mg×6) 5mg QD ×1m  Yuping Feng Granules(5g/bag) 5g TID ×1m | 1.86±0.37 years | 8.14±1.29 | 12 |
|  |  |  |  |  | Montelukast Sodium 36 |  | 1.95±0.42 years | 8.28±1.32 |  |
| Bai Huiping (2019). | China | RCT | 1 month | 40 | Yuping Feng Granules + Montelukast Natrium 20 | Montelukast 4mg QD ×1m  Yuping Feng Granules2.5g TID (dissolve) ×1m | 1.6±0.3 years | 5.4±0.4 | 14 |
|  |  |  |  |  | Montelukast Sodium 20 |  | 1.6±0.4 years | 5.3±0.5 |  |
| Fang Ruiping (2017). | China | RCT | 1 month | 60 | Yuping Feng Granules + Montelukast Sodium 30 | Montelukast 1 tab QD ×1m  Yuping Feng Granules 1 bag TID (dissolve) ×1m | 2.78±0.35 years | 7.45±0.82 | 124 |
|  |  |  |  |  | Montelukast Sodium 30 |  | 2.78±0.35 years | 7.82±0.98 |  |
| Zhang Yang (2012). | China | RCT | 1 month | 100 | Yuping Feng Granules + Mometasone Furoate Spray 55 | Mometasone nasal spray 1 spray/nostril QD AM ×1m  Yuping Feng Granules 0.5 bag TID ×1m | - | 9.2 | 1 |
|  |  |  |  |  | Mometasone Furoate Spray 45 |  | - | 10.5 |  |
| Zhang Gengchang (2013). | China | RCT | 60 days | 100 | Xinqin Granules + Pidotimod Oral Solution 50 | Pidotimod oral solution - Acute phase: 400mg BID ×2w (2 courses) -Prevention: 400mg QD ×60d (2 courses) Xinqin Granules 5g TID ×2w (2 courses) | 2.1±1.1 years | 8.5±2.9 | 1 |
|  |  |  |  |  | Pidotimod Oral Solution 50 |  | 2.2±1.4 years | 9.2±2.3 |  |
| Jiang Lianying (2023). | China | RCT | 3 weeks | 100 | Sanfeng Tongqiao Dropping Pills + Loratadine Tablets 50 | Loratadine tab (10mg/tab) >12y & >30kg: 10mg QD ≤12y & ≤30kg: 5mg QD ×3w  Sanfeng Tongqiao Dropping Pills (0.76g/20pills) 10 pills TID×3w | 4.92±1.58 weeks | 9.45±1.16 | 1234 |
|  |  |  |  |  | Loratadine Tablets 50 |  | 5.52±1.06 weeks | 9.87±1.05 |  |
| Chen Qiaxin (2017). | China | RCT | 1 month | 320 | Yuping Feng Granules + Montelukast 170 | Montelukast 4mg QD ×1m  Yuping Feng Granules 5mg TID ×1m | 3.9±0.2 years | 8.4±0.4 | 1 |
|  |  |  |  |  | Montelukast 150 |  | 3.8±0.1 years | 8.3±0.3 |  |
| Yang Na (2019). | China | RCT | 4 weeks | 69 | Tongqiao Biyan Granules + Triamcinolone Acetonide Nasal Spray 33 | Triamcinolone nasal spray (55μg/spray) 6-12y: 1 spray/nostril QD (110μg/d) >12y: 2 sprays/nostril QD (220μg/d)  Tongqiao Biyan Granules (2g/bag) 6-8y: 1g TID >8y: 2g TID×4w | 4-14 weeks | 6-14 | 134 |
|  |  |  |  |  | Triamcinolone Acetonide Nasal Spray 36 |  | 4-14 weeks | 6-14 |  |
| Wang Jiaping (2023). | China | RCT | 4 weeks | 104 | Tongqiao Biyan Granules + Oxymetazoline Hydrochloride Nasal Spray 52 | Oxymetazoline nasal spray (50g/bottle)1-3 sprays/nostril BID ×4w   Tongqiao Biyan Granules (2g/bag) 4-9y: 1g TID (dissolve) 10-15y: 2g TID (dissolve)×4w | 9.71±2.15 months | 8.43±1.52 | 134 |
|  |  |  |  |  | Oxymetazoline Hydrochloride Nasal Spray 52 |  | 12.03±3.29 months | 10.61±1.87 |  |
| Yi Peiyu (2020). | China | RCT | 2 weeks | 100 | Tongqiao Biyan Granules + Mometasone Furoate Nasal Spray 50 | Mometasone nasal spray (50μg/spray) 1 spray/nostril QD ×2w  Tongqiao Biyan Granules (2g/bag) 4-6y: 1g TID 6-12y: 2g BID 8-12y: 2g TID ×2w | 1.5±0.7 years | 7.8±3.1 | 14 |
|  |  |  |  |  | Mometasone Furoate Nasal Spray 50 |  | 1.4±0.6 years | 7.9±3.4 |  |
| Huang Yong (2018). | China | RCT | 2 weeks | 90 | Tongqiao Biyan Granules + Ketotifen 45 | Tongqiao Biyan Granules 2g TID (dissolve)  Ketotifen 0.5mg BID×2w | 7.2±2.8 weeks | 8.4±3.3 | 14 |
|  |  |  |  |  | Ketotifen 45 |  | 7.4±3.1 weeks | 8.3±2.8 |  |
| Wang Yan (2017). | China | RCT | 2 weeks | 80 | Tongqiao Biyan Granules + Cetirizine Hydrochloride Drops 40 | Cetirizine drops (50mg:50mL) Option 1: 0.5mL QD Option 2: 0.25mL BID ×2w  Tongqiao Biyan Granules(28g/bag) 28g TID (dissolve) ×2w | 1.03±0.41 years | 6.24±1.65 | 134 |
|  |  |  |  |  | Cetirizine Hydrochloride Drops 40 |  | 1.01±0.39 years | 6.27±1.69 |  |
| Chen Pingping (2022). | China | RCT | 2 months | 80 | Tongqiao Biyan Granules + Azelastine Nasal Spray + Cetirizine Oral 40 | Azelastine nasal spray (10ml:10mg) 2 sprays BID  Cetirizine oral soln (10ml:10mg) 5ml BID ×2m  Tongqiao Biyan Granules (2g/bag) 2g TID (dissolve) ×2m | 8.61±1.43 months | 7.85±1.53 | 1234 |
|  |  |  |  |  | Azelastine Nasal Spray + Cetirizine Oral 40 |  | 8.76±1.38 months | 7.96±1.73 |  |
| Cui Long (2016). | China | RCT | 2 weeks | 50 | Tongqiao Biyan Tablets + Mometasone Furoate Nasal Spray 25 | Mometasone nasal spray (50μg/spray) 2 sprays/nostril QD (adjust PRN) ×2w  Tongqiao Biyan Tablets 5-7 tabs TID ×2w | 2.2±0.5 weeks | 6.1±2.2 | 134 |
|  |  |  |  |  | Mometasone Furoate Nasal Spray 25 |  | 2.4±0.6 weeks | 6.3±2.5 |  |
| Wang Qing (2021). | China | RCT | 90 days | 84 | Tongqiao Biyan Tablets + Loratadine Tablets + Pitomide Oral Solution 42 | Loratadine tab 10mg QD  Pidotimod oral soln 1 amp BID ×90d  Tongqiao Biyan Granules 5-7 tabs TID ×90d | 1.28±0.34 years | 8.82±1.35 | 134 |
|  |  |  |  |  | Loratadine Tablets + Pitomide Oral Solution 42 |  | 1.24±0.31 years | 8.79±1.32 |  |
| Cao Zhihong (2014). | China | RCT | 14 days | 60 | Xiangju Capsules + Claritin Syrup 30 | Loratadine syrup 2-5y: 5ml QD 6-14y: 10ml QD ×14d  Xiangju Capsules 2-5y: 1 cap TID 6-14y: 2 caps TID ×14d | - | 2-14 | 1 |
|  |  |  |  |  | Claritin Syrup 30 |  | - | 2-14 |  |
| Chen Wenli (2024). | China | RCT | 4 weeks | 98 | Xiangju Capsules + Cetirizine 49 | Cetirizine 10mg QD ×4w  Xiangju Capsules (0.3g/cap) 0.6g (2 caps) TID ×4w | - | 9.65±1.24 | 14 |
|  |  |  |  |  | Cetirizine 49 |  | - | 9.63±1.22 |  |
| Fu Quansheng (2023). | China | RCT | 4 weeks | 122 | Xiangju Capsules + Cetirizine Hydrochloride Drops 61 | Cetirizine HCl drops (10mL:0.1g) 1mL QD ×4w  Xiangju Capsules (0.3g/cap) 2-4 caps TID ×4w | 1.33±0.22 years | 9.77±2.16 | 124 |
|  |  |  |  |  | Cetirizine Hydrochloride Drops 61 |  | 1.35±0.25 years | 9.52±2.33 |  |
| Li Xiangyi (2010). | China | RCT | 3 months | 128 | Yuping Feng Granules + Budesonide Nasal Spray 64 | Budesonide nasal spray (64μg/spray) 2 sprays/nostril BID ×3m  Yuping Feng Granules 1 bags 2-3 times daily ×3m | 2.4 years | 8.9 | 14 |
|  |  |  |  |  | Budesonide Nasal Spray 64 |  | 2.6 years | 8.5 |  |
| Yu Xiaoyan (2017). | China | RCT | 30 days | 238 | Xinqin Granules + Mometasone Furoate Nasal Spray 119 | Mometasone nasal spray (50μg/spray) 2 sprays QD AM ×30d  Xinqin Granules (5g/bag) 5g BID ×30d | 11.56±7.56 months | 4.22±1.12 | 124 |
|  |  |  |  |  | Mometasone Furoate Nasal Spray 119 |  | 11.35±6.24 months | 4.12±1.03 |  |
| Zheng Meng (2023). | China | RCT | 28 days | 60 | Xinqin Granules + Mometasone Furoate Nasal Spray 30 | Mometasone nasal spray 2 sprays QD ×28d  Xinqin Granules 5g QID ×28d | 15.13±1.58 months | 6.73±1.17 | 13 |
|  |  |  |  |  | Mometasone Furoate Nasal Spray 30 |  | 15.18±1.62 months | 6.75±1.20 |  |
| Xu Sufen (2014). | China | RCT | 3 weeks | 64 | Yuping Feng Granules + Loratadine Syrup 32 | Loratadine syrup 5ml QD ×3w  Yuping Feng Granules 5g TID (pre-meal) ×3w | 2.36 years | 5.2 | 1 |
|  |  |  |  |  | Loratadine Syrup 32 |  | 2.41 years | 4.9 |  |
| Yang Zhihua (2018). | China | RCT | 4 weeks | 126 | Yuping Feng Granules + Montelukast Sodium Chewable Tablets 63 | Montelukast chewable tab <6y: 4mg QD ≥6y: 5mg QD ×4w  Yuping Feng Granules (5g/bag) 5g TID ×4w | 1.81±1.44 years | 8.57±2.12 | 14 |
|  |  |  |  |  | Montelukast Sodium Chewable Tablets 63 |  | 1.83±0.42 years | 8.63±2.07 |  |
| Fan Ruimin (2013). | China | RCT | 2 weeks | 66 | Yuping Feng Granules + Desloratadine Dry Suspension 36 | Desloratadine susp 1-5y: 1.25mg QD 6-11y: 2.5mg QD ×2w Yuping Feng Granules (5g/bag) <4y: 2.5g BID ≥4y: 5g BID ×2w | 1 month to 5 years | 3-12 | 14 |
|  |  |  |  |  | Desloratadine Dry Suspension 30 |  | 2 months to 5 years | 3-13 |  |
| Wu Tingting (2018). | China | RCT | 4 weeks | 72 | Yuping Feng Granules + Loratadine 36 | Loratadine (10mg/tab) ≤30kg: 5mg QD >30kg: 10mg QD ×4w  Yuping Feng Granules 5g TID (dissolve)×4w + 3m FU | 2.56±0.96 years | 7.08±1.09 | 124 |
|  |  |  |  |  | Loratadine 36 |  | 2.65±0.85 years | 7.25±1.22 |  |
| Wang Yinjia (2019). | China | RCT | 1 month | 120 | Yuping Feng Granules + Montelukast Sodium 60 | Montelukast 1 tab QD ×1m  Yuping Feng Granules 5g TID ×1m | 27.4±0.8 months | 7.23±0.65 | 24 |
|  |  |  |  |  | Montelukast Sodium 60 |  | 26.5±0.8 months | 7.61±0.89 |  |
| Cai Jianliang (2025). | China | RCT | 30 days | 100 | Yuping Feng Granules + Montelukast Sodium Granules 50 | Montelukast granules (0.5g:4mg) 4mg QD ×30d  Yuping Feng Granules (5g/bag) 2.5g TID ×30d | 1.95±0.49 | 8.93±1.25 | 14 |
|  |  |  |  |  | Montelukast Sodium Granules 50 |  | 1.98±0.51 | 8.49±1.12 |  |
| Ye Qing (2014). | China | RCT | 4 weeks | 119 | Yuping Feng Granules + Montelukast Sodium Tablets 61 | Montelukast tab2-5y: 4mg QD HS 6-13y: 5mg QD HS ×4w  Yuping Feng Granules <3y: 2g TID 3-6y: 3g TID >6y: 5g TID ×4w | 4 weeks - 9 months | 3-13 | 1 |
|  |  |  |  |  | Montelukast Sodium Tablets 58 |  | 4 weeks - 8 months | 4-13 |  |
| Yang Mei (2021). | China | RCT | 2 weeks | 80 | Yuping Feng Granules + Montelukast Sodium 40 | Montelukast 1-2y: 2mg QD >2y: 4mg QD ×1w  Yuping Feng Granules 1 bag TID ×2w | 18.53±6.65 months | 3.12±2.75 | 14 |
|  |  |  |  |  | Montelukast Sodium 40 |  | 18.55±5.12 months | 3.23±2.32 |  |
| Yu Meijia (2015). | China | RCT | 30 days | 72 | Yuping Feng Granules + Montelukast Sodium 36 | Montelukast 4mg QD ×30d  Yuping Feng Granules 5mg TID (dissolve) ×30d | 4.8±2.5 years | 7.3±3.5 | 124 |
|  |  |  |  |  | Montelukast Sodium 36 |  | 4.9±2.7 years | 7.4±3.6 |  |
| Chen Sihong (2008). | China | RCT | 6 months | 104 | Buzhong Yiqi Pills + Loratadine Tablets 52 | Loratadine tab (10mg/tab) <25kg: 5mg QD HS ≥25kg: 10mg QD HS ×3m → QOD ×3m  Buzhong Yiqi Pills (10g/pill) <25kg: 0.5 pill BID AC ≥25kg: 1 pill BID AC ×3m → QOD ×3m | 0.5-5 years | 7-13 | 14 |
|  |  |  |  |  | Loratadine Tablets 52 |  | 0.5-5 years | 6-14 |  |

Abbreviations: PO, oral intake; QD, once daily; BID, twice daily; TID, three times daily; y, years old; m, month; w, week; d, day; pc, after meals; HS, at bedtime; QOD, every other day; AC, before meals; →, indicates subsequent adjustment of regimen ;Outcome indicator : 1 , Effective rate ; 2 , IgE ; 3 , VAS score for the core symptoms of allergic rhinitis (Nasal obstruction, Nasal pruritus, Paroxysmal sneezing, Nasal discharge) ; 4 , Adverse event rate .

**Table S3.2:** Characteristics and Formulations of Included Chinese Proprietary Medicines

| Medicine | Syndrome Pattern | Therapeutic Action | Indication | Composition | Note |
| --- | --- | --- | --- | --- | --- |
| Nasal Comfort Tablets | External Pathogen Invading the Lung--Wind-Heat invading the Lung | Clears heat and resolves toxicity, disperses wind and reduces swelling, soothes the throat and unblocks the orifices. | Acute and chronic rhinitis, sinusitis, and pharyngitis due to Wind-Heat. | *Duhaldea cappa* (Buchanan-Hamilton ex D. Don) Pruski & Anderberg*、Houttuynia cordata* Thunb.*、Spiraea salicifolia* L.*、Cirsium japonicum* Fisch. ex DC.*、Sagina japonica* (Sw.) Ohwi*、Liquidambar formosana Hance、Centipeda minima (L.) A. Braun e tAschers.、Starch* |  |
| Biyuan Shu Oral Liquid | External Pathogen Invading the Lung--Wind-Heat invading the Lung | Disperses wind and clears heat, eliminates dampness and unblocks the orifices. | Rhinitis and sinusitis attributed to Wind-Heat of the Lung Channel or Stagnant Heat in the Gallbladder. | *Xanthium strumarium* L.*、Magnolia biondii* Pamp.*、Mentha canadensis* L.*、Angelica dahurica* (Fisch. ex Hoffm.) Benth. & Hook. f. ex Franch. & Sav.*、Scutellaria baicalensis* Georgi*、Gardenia jasminoides* J.Ellis*、Bupleurum chinense* DC.*、Asarum heterotropoides* F. Schmidt*、Ligusticum chuanriong* Hort.*、Astragalus membranaceus* (Fisch.) Bunge*、Clematis armandii Franch.、Platycodon grandiflorus* (Jacq.) A.DC. | ⚠ *Xanthium strumarium* L.: It contains toxic principles and must undergo Pao Zhi (processing) before it can be used medicinally.  ⚠ *Asarum heterotropoides* F. Schmidt: The presence of aristolochic acid imparts a well-established risk of nephrotoxicity.  ⚠ *Clematis armandii* Franch.:It has been historically mistaken for Aristolochia manshuriensis (Guang Mutong). There are documented reports of nephrotoxicity associated with the use of Aristolochia manshuriensis. |
| Biyuan Tongqiao Granules | External Pathogen Invading the Lung--External pathogen invading the Lung syndrome | Disperses wind and clears heat, diffuses the Lung and unblocks the orifices. | Acute sinusitis (Jí Bí Yuān) due to External Pathogen Invading the Lung syndrome. Clinical manifestations: frontal or zygomatic tenderness, intermittent nasal obstruction, mucoid or purulent Nasal discharge, headache, possible fever, thin yellow or white tongue coating, floating pulse. | *Magnolia biondii* Pamp.*、Xanthium strumarium* L.(Stir-frying)*、Ephedra sinica* Stapf*、Angelica dahurica* (Fisch. ex Hoffm.) Benth. & Hook. f. ex Franch. & Sav.*、Mentha canadensis* L.*、Conioselinum anthriscoides* (H. Boissieu) Pimenov & Kljuykov*、Scutellaria baicalensis* Georgi*、Forsythia suspensa* (Thunb.) Vahl*、Chrysanthemum indicum* L.*、Trichosanthes kirilowii* Maxim.*、Rehmanniaglutinosa* (Gaertn.) Libosch. ex Fisch. & C. A. Mey.*、Salvia miltiorrhiza* Bunge*、Poria cocos* (Schw.) Wolf*、Glycyrrhiza uralensis* Fisch.*。* | ⚠ *Xanthium strumarium* L.: It contains toxic principles and must undergo Pao Zhi (processing) before it can be used medicinally.  ⚠ *Ephedra sinica* Stapf:Its use is relatively contraindicated in patients with cardiovascular disease due to the presence of ephedrine.  ⚠ *Glycyrrhiza uralensis* Fisch.: Prolonged administration at high doses may lead to water and sodium retention. |
| Buzhong Yiqi Pills | Deficiency of Vital Qi--Spleen Qi deficiency | Tonifies the Middle Jiao and augments Qi, raises Yang and lifts prolapse; with additional effects of immunomodulation, anemia improvement and fatigue resistance. | Symptoms including fatigue, poor appetite, abdominal distension, chronic diarrhea, rectal prolapse, and uterine prolapse caused by Spleen-Stomach Deficiency and Sinking of Middle Qi. | *Astragalus membranaceus* (Fisch.) Bunge（Honey-processing）*、Codonopsis pilosula (Franch.)* Nannf.*、Glycyrrhiza uralensis* Fisch.（Honey-processing）*、Atractylodes macrocephala* Koidz.（Stir-frying）*、Angelica sinensis* (Oliv.) Diels*、Actaea cimicifuga* L.*、Bupleurum chinense* DC.*、Citrus reticulata Blanco.、Zingiber officinale* Roscoe (fresh)*、Ziziphus jujuba* Mill.*。* | ⚠ *Glycyrrhiza uralensis* Fisch.: Prolonged administration at high doses may lead to water and sodium retention. |
| Cang'er Zibi Yan Pills | External Pathogen Invading the Lung--Wind-Heat invading the Lung | Dispels wind and unblocks the orifices, clears heat and resolves toxicity; ameliorates nasal inflammatory responses. | Adjunctive therapy for conditions such as acute and chronic rhinitis, and allergic rhinitis. | *Xanthium strumarium* L. Extract Powder*、Gypsum Fibrosum* Extract Powder*、Angelica dahurica* (Fisch. ex Hoffm.) Benth. & Hook. f. ex Franch. & Sav. Extract Powder*、Cinnamomum camphora* (L.) Presl*、Magnolia biondii* Pamp.*、Mentha canadensis* L.*、Magnolia biondii* Pamp. Extract Powder*、Scutellaria baicalensis* Georgi Extract Powder | ⚠ *Xanthium strumarium* L.: It contains toxic principles and must undergo Pao Zhi (processing) before it can be used medicinally. |
| Danxi Yuping Feng Granules | Wei Qi Deficiency and Instability--Exterior deficiency failing to secure | Augments Qi, stabilizes the Exterior and arrests sweating. | Exterior Deficiency and Insecurity presenting with spontaneous sweating, aversion to wind, or susceptibility to external pathogens due to constitutional deficiency. | *Astragalus membranaceus* (Fisch.) Bunge*、Saposhnikovia divaricata* (Turcz.) Schischk.*、Atractylodes macrocephala* Koidz.(Stir-frying)*、*Sucrose、Maltodextrin*。* |  |
| Huaiqi Huang Granules | Deficiency of Vital Qi--Qi and Yin deficiency | Augments Qi and nourishes Yin. | Constitutional weakness in children with recurrent colds or post-illness debility in the elderly due to Qi and Yin Deficiency. Symptoms include: dizziness, fatigue, dry mouth, shortness of breath, palpitations, hyperhidrosis, poor appetite, and constipation. | *Sophora japonica-fungus fermented substrate、Lycium barbarum* L.*、Polygonatum sibiricum* Delar. ex Redoute*，*Sucrose、Soluble Starch*。* |  |
| Lianhua Qingwen Granules | External Pathogen Invading the Lung--Heat-toxin assailing the Lung | Clears epidemic toxins, diffuses the Lung and drains heat. | Treatment of influenza characterized by Heat-Toxin Attacking the Lung syndrome. Symptoms include: fever or high fever with chills, myalgia, nasal congestion and discharge, cough, headache, dry and sore throat, reddish tongue with yellow or yellow-greasy coating. | *Forsythia suspensa* (Thunb.) Vahl*、Lonicera japonica* Thunb.*、*Honey-fried *Ephedra sinica* Stapf*、*Stir-frying *Prunus armeniaca* L. var. ansuMaxim.*、Gypsum Fibrosum、Isatis indigotica* Fort.*、Dryopteris crassirhizoma* Nakai*、Houttuynia cordata* Thunb.*、Pogostemon cablin* (Blanco) Benth.*、Rheum palmatum* L.*、Rhodiola rosea* L*.、Mentha canadensis* L.*、Glycyrrhiza uralensis* Fisch. | ⚠ *Ephedra sinica* Stapf:Its use is relatively contraindicated in patients with cardiovascular disease due to the presence of ephedrine.  ⚠ *Glycyrrhiza uralensis* Fisch.: Prolonged administration at high doses may lead to water and sodium retention.  ⚠ *Prunus armeniaca* L. var. ansuMaxim.:It contains cyanogenic glycosides and must be processed to achieve detoxification.  ⚠ *Dryopteris crassirhizoma* Nakai:It has inherent toxicity, which requires careful dosage control.  ⚠ *Rheum palmatum* L. :Long-term administration of its anthraquinone components is associated with the development of melanosis coli. |
| Sanfeng Tongqiao Dropping Pills | External Pathogen Invading the Lung--Wind-Heat accumulating in the Lung, complicated by exterior deficiency | Disperses wind and clears heat, diffuses the Lung and unblocks the nasal orifices. | Alleviates symptoms including nasal congestion, Nasal discharge, and headache associated with acute/chronic rhinitis and allergic rhinitis. | *Scutellaria baicalensis* Georgi*、Nepeta cataria* L*.、Hansenia weberbaueriana* (Fedde ex H. Wolff) Pimenov & Kljuykov*、Asarum heterotropoides* F. Schmidt | ⚠ *Asarum heterotropoides* F. Schmidt: The presence of aristolochic acid imparts a well-established risk of nephrotoxicity. |
| Tongqiao Biyan Granules | External Pathogen Invading the Lung--Wind-Heat accumulating in the Lung, complicated by exterior deficiency | Disperses wind and reduces inflammation, diffuses the Lung and unblocks the nasal orifices. | Sinusitis (Bí Yuān), nasal congestion, Nasal discharge, frontal headache; rhinitis, sinusitis, and allergic rhinitis. | *Xanthium strumarium* L.(Stir-frying)*、Saposhnikovia divaricata* (Turcz.) Schischk.*、Astragalus membranaceus* (Fisch.) Bunge*、Angelica dahurica* (Fisch. ex Hoffm.) Benth. & Hook. f. ex Franch. & Sav.*、Magnolia biondii* Pamp.*、Atractylodes macrocephala* Koidz.(Stir-frying)*、Mentha canadensis* L. | ⚠ *Xanthium strumarium* L.: It contains toxic principles and must undergo Pao Zhi (processing) before it can be used medicinally. |
| Xiangju Capsules | External Pathogen Invading the Lung--Wind-Heat invading the Lung | Releases pathogens acridly to disperse wind, clears heat and unblocks the orifices. | Nasal congestion and headache due to acute/chronic sinusitis; nasal mucosal congestion caused by allergic rhinitis; olfactory decline resulting from chronic rhinitis (these indications have been clinically validated). | Defruited Inflorescence of Xiang Shu (seeds removed)*、Prunella vulgaris* L.*、Chrysanthemum indicum* L.*、Astragalus membranaceus* (Fisch.) Bunge*、Magnolia biondii* Pamp.*、Saposhnikovia divaricata* (Turcz.) Schischk.*、Angelica dahurica (*Fisch. ex Hoffm.) Benth. & Hook. f. ex Franch. & Sav.*、Ligusticum chuanriong* Hort.*、Glycyrrhiza uralensis* Fisch. | ⚠ *Glycyrrhiza uralensis* Fisch.: Prolonged administration at high doses may lead to water and sodium retention. |
| Xinqin Granules | Wei Qi Deficiency and Instability--Exterior deficiency failing to secure | Augments Qi and stabilizes the Exterior, dispels wind and unblocks the orifices. | For nasal itching, sneezing, clear Nasal discharge, and susceptibility to common cold due to Lung Qi Deficiency and external Wind invasion; Allergic rhinitis presenting with the aforementioned manifestations. | *Asarum heterotropoides* F. Schmidt*、Scutellaria baicalensis* Georgi*、Xanthium strumarium* L.*、Angelica dahurica* (Fisch. ex Hoffm.) Benth. & Hook. f. ex Franch. & Sav.*、Nepeta cataria* L.*、Saposhnikovia divaricata* (Turcz.) Schischk.*、Acorus gramineus、Atractylodes macrocephala* Koidz.*、Cinnamomum cassia* (L.) D. Don*、Astragalus membranaceus* (Fisch.) Bunge*。* | ⚠ *Xanthium strumarium* L.: It contains toxic principles and must undergo Pao Zhi (processing) before it can be used medicinally. ⚠ *Asarum heterotropoides* F. Schmidt: The presence of aristolochic acid imparts a well-established risk of nephrotoxicity.  ⚠ *Acorus gramineus*: Its volatile oil content is associated with dose-dependent neurotoxicity. |
| Yuping Feng Granules | Deficiency of Vital Qi--Exterior deficiency failing to secure | Augments Qi, stabilizes the Exterior and arrests sweating. | Exterior Deficiency and Insecurity characterized by spontaneous sweating, aversion to wind, pale complexion, or susceptibility to external pathogens due to constitutional deficiency. | *Astragalus membranaceus* (Fisch.) Bunge*、Atractylodes macrocephala* Koidz.（Stir-frying）*、Saposhnikovia divaricata* (Turcz.) Schischk.*。* |  |

**Table S3.3:** Manufacturer Details and Regulatory Information of Included Medicines

| Medicine | Manufacturer | Specification | Approval Number | Production Batch Number |
| --- | --- | --- | --- | --- |
| Nasal Comfort Tablets | Guizhou Guangzheng Pharmaceutical | 0.35g/tablet | Z20073048 | 20180925 |
| Bi Yuan Shu Oral Liquid | Chengdu Huashen Group Co., Ltd. Pharmaceutical Factory | 10 mL/tube | Z51020208 | 201710011、201911024 |
| Biyuan Tongqiao Granules | Shandong Xinshidai Pharmaceutical Co., Ltd. | 15 g/bag | Z20030071 | 17062010、18092330、20150125、2150226、20160215、20181009 |
| Buzhong Yiqi Pills | Yunnan Province Tengchong Pharmaceutical Factory Production | 10g x 10 pills | Z53021041 | - |
| Cang'er Zibi Yan Pills | Sichuan Zhiku Mountain Pharmaceutical Co., Ltd. | 1.2g (28 pills) x 6 sets | Z20090560 | - |
| Danxi Yuping Feng Granules | Yunnan Baiyao Group Co., Ltd. | 5g x 6 packs | Z53021556 | - |
| Huaiqi Huang Granules | Qidong Gaitianli Pharmaceutical Co., Ltd. | 16g×6 bags | B20020074 | - |
| Lianhua Qingwen Granules | Beijing Yiling Pharmaceutical Co., Ltd. | 6g x 10 bags | Z20100040 | - |
| Sanfeng Tongqiao Dropping Pills | Yangtze River Pharmaceutical Group Co., Ltd. | 0.76 g/20 pills | Z20194026 | 21102591、22012491 |
| Tongqiao Biyan Granules | Chengdu Dekang Technology Pharmaceutical Co., Ltd. | 2 g x 18 bags | Z10980073 | 160109、170507、171201 |
|  | Sichuan Chuanda Huaxi Pharmaceutical Co., Ltd. | 2 g x 9 bags | Z10970123 | - |
| Xiangju Capsules | Shandong Buchang Pharmaceutical Co., Ltd. | 0.3 g/tablet | Z19991040 | 220920 |
| Xinqin Granules | Sichuan Zhiyuan Guanghe Pharmaceutical Co., Ltd. | 5 g/bag | Z51020011 | 1503125 |
|  | Jiangxi Xinglin Bai마 Pharmaceutical Co., Ltd. | 5g×12 bags | Z31020014 | - |
| Yuping Feng Granules | Guangdong Global Pharmaceutical Co., Ltd. | 5 g/bag | Z10930036 | 20150413、09050 |
|  | North China Pharmaceutical Co., Ltd. Production |  | - | - |

| Medicine | Manufacturer | Specification | Approval Number | Production Batch Number |
| --- | --- | --- | --- | --- |
| Fluticasone Propionate Nasal Spray | GLAXOSMITHKLINE S.A. | 120 sprays per bottle | HJ20140117 | - |
| Budesonide Nasal Spray | AstraZeneca Pharmaceutical Co., Ltd. | 120 sprays | J201400483 | 20171205 20181022 |
|  | Shandong Jinan Yonghe Pharmaceutical Co., Ltd. Production | 64ug per spray | J20190031 | - |
| Azelastine Nasal Spray | Guizhou Yunfeng Pharmaceutical Co., Ltd. Production | 10 ml ∶ 10 mg | H20041039 | - |
| Desloratadine Dry Suspension | Hainan Puli Pharmaceutical Co., Ltd. | 2.5mg/bag | H20041111 | - |
| Claritin Syrup | Schering-Plough Pharmaceutical Factory Licensed in Belgium | 1ml: 1mg | H20050245 | - |
| Mometasone Furoate Nasal Spray | Zhejiang Xianju Pharmaceutical Co., Ltd. | 50 μg per puff | H20113481 | 20170715、20180624 |
|  | Schering-Plough Labo N.V. |  | H20140100 | 1412043 |
|  | Merck Sharp & Dohme Pharmaceutical Co., Ltd. |  | H20090192 | - |
| Loratadine Tablets | Xian-Janssen Pharmaceutical Ltd. Production | 10 mg/tablet | H20070030 | 20210406、20220302 |
|  | Guangdong Yishu Pharmaceutical Co., Ltd. |  | 20052214 | 20191213 |
|  | Shenzhen HaiBin Pharmaceutical Co., Ltd. |  | H20031299 | - |
|  | Jiangsu Huanghe Pharmaceutical Production |  | H20050953 | - |
| Montelukast Sodium | Sichuan Dajia Pharmaceutical Co., Ltd. | 10 mg/tablet | H20064370 | - |
| Montelukast Sodium Chewable Tablets | Lunan Better Pharmaceutical Co., Ltd. | 5mg per tablet | H20083372 | 20150222 |
|  | Merck Sharp & Dohme Pharmaceutical Co., Ltd. |  | J20130054 | 080525 |
|  |  | 10mg x 5 tablets | J20120072 | - |
|  |  |  | J20130047 | - |
| Montelukast Sodium Granules | Suzhou Wugujing Pharmaceutical Co., Ltd. | 0.5g: 4mg | H20203429 | - |
| Pidotimod Oral Solution | Jiangsu Wuzhong Pharmaceutical Group Co., Ltd. Suzhou Pharmaceutical Factory | 10 mL: 0.4 g per stick | H20030463 | 20150123、2150218、20160305 |
|  | POLICHEM S.R.I | 7ml: 400mg | H20150635 | 20191216 |
| Triamcinolone Acetonide Nasal Spray | Jiangxi Zhen Shi Ming Pharmaceutical Co., Ltd. | 6.6 mg ∶ 6 ml 55 μg/spray | H20010780 | 151201、160903、171201 |
| Cetirizine | Suzhou Dongrui Pharmaceutical Co., Ltd. | 10 mg/tablet | H19980014 | - |
|  | Shandong Sibondo Pharmaceutical Co., Ltd. |  | H20084632 | - |
| Cetirizine Oral Solution | Beijing Hanmei Pharmaceutical Co., Ltd. production | 10 ml: 10 mg | H20093320 | - |
| Xylometazoline Hydrochloride Nasal Spray | Beijing Tongrentang Co., Ltd. Tongrentang Pharmaceutical Factory | 50 g/bottle | Z11020600 | - |
| Cetirizine Hydrochloride Drops | Chengdu Minyi Pharmaceutical Co., Ltd. | 10 mL: 0.1 g | H20000723 | 201709003、201908012、22D19 |
|  | Amerigen Pharmaceuticals |  | HC20181014 | 20181014 |
| Ketotifen | Unspecified | - | - | - |

**Appendix 4: List of data extracted from the included randomized clinical trials**

| Data category | List of variables |
| --- | --- |
| Study | Primary author, year of publication, duration of study, total number of patients in each group |
| Patients | Age, nationality, allergic rhinitis duration, Symptom Scores(Nasal obstruction,Nasal pruritus,Paroxysmal sneezing,Nasal discharge) and IgE at baseline |
| Interventions | Drug class, dose and duration of the primary intervention and strategies used for implementing them |
|  |  |
| Efficacy outcome | Mean of change in Total Nasal Symptom Score(Nasal obstruction,Nasal pruritus,Paroxysmal sneezing,Nasal discharge) , IgE and Effective rate respective standard deviation from baseline |
|  |  |
| Adverse events | Various adverse events reported in included trials |

**Appendix 5: Risk of bias of randomized clinical trials**

**Figure S5:** Overall risk of bias presented as percentage of each risk of bias item across all included studies. Green = Low risk, Red = High risk, Yellow = Some concerns.


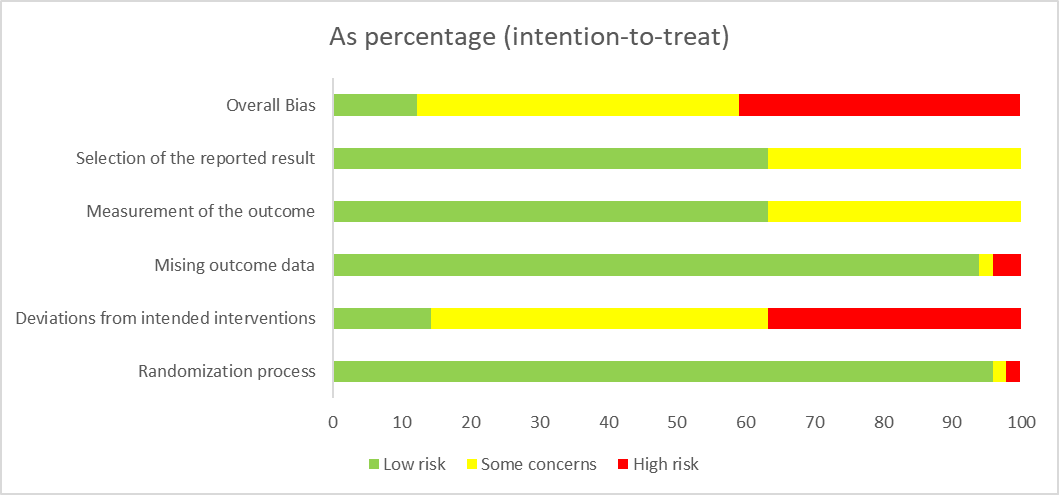


**Table S5:** Study level risk of bias assessment using Cochrane risk of bias tool 2.0 for assessing risk of bias of randomized clinical trials.

| Unique ID | Study ID | Randomization process | Deviations from intended  interventions | Mising outcome data | Measurement of the outcome | Selection of the  reported result | Over all |
| --- | --- | --- | --- | --- | --- | --- | --- |

| Ma Shufang (2020). | DOI:10.19829/j.zgfybj.issn.1001-4411.2020.19.028 | Low | Low | Low | Low | Low | Low |
| --- | --- | --- | --- | --- | --- | --- | --- |
| Fang Chunru (2020). | DOI: 10.7501/i.issn.1674-5515.2020.11.028 | Low | Some concerns | Low | Low | Low | Some concerns |
| Lin Xiaoyan (2019). | DOI:10.7501/i.issn.1674-5515.2019.06.030 | Low | Some concerns | Low | Low | Low | Some concerns |
| Xie Liangchao (2018). | 10.3969/j.issn.1004-2814.2018.07.037 | Low | High | Low | Some concerns | Some concerns | High |
| Wang Ranran (2019). | 10.11798/j.issn.1007-1520.201903018 | Low | Low | Low | Low | Low | Low |
| Lin Zhihua (2021). | NA | Low | Some concerns | Low | Low | Low | Some concerns |
| Fan Jinping (2017). | 10.7501/j.issn.1674-5515.2017.08.021 | Low | Some concerns | Low | Low | Low | Some concerns |
| Yu Wangbo (2019). | 10.3969/j.issn.1672-3511.2019.03.018 | Low | Some concerns | Low | Low | Low | Some concerns |
| Sun Xinxin (2020). | 10.3969/j.issn.1009-0959.2020.11.009 | Low | Some concerns | Some concerns | Low | Low | Some concerns |
| Dong Tianjin (2023). | 10.12010/j.issn.1673-5846.2023.06.009 | Low | Some concerns | Low | Low | Low | Some concerns |
| Chen Lilei (2016). | 10.3969/j.issn.1671-3141.2016.38.062 | Low | High | Low | Some concerns | Some concerns | High |
| Jiang Hua (2018). | 10.3969/j.issn.1000-7369.2018.07.011 | Low | Low | Low | Low | Low | Low |
| Tu Jing (2020). | 10.3969/j.issn.1672-6170.2020.05.045 | Low | Some concerns | Low | Low | Low | Some concerns |
| Sun Jingyuan (2022). | 10.3969/j.issn.1002-7386.2022.09.011 | Low | Some concerns | Low | Low | Low | Some concerns |
| Zhu Lang (2023). | NA | Low | High | High | Some concerns | Some concerns | High |
| Xu Yan (2017). | 1007-8231（2017）05-0142-02 | Low | High | Low | Some concerns | Some concerns | High |
| Liu Zhaohong (2023). | 10.19617/j.issn1001-1307.2023.01.55 | Low | Some concerns | Low | Low | Low | Some concerns |
| Lv Min (2024). | 1002-8714(2024)03-0105-03 | Low | Some concerns | Low | Low | Low | Some concerns |
| Chen Longfeng (2023). | 10.19617/j.issn1001-1307.2023.06.103 | Low | Some concerns | Low | Low | Low | Some concerns |
| Bai Huiping (2019). | DOI:10.19335/j.cnki.2096-1219.2019.21.068 | Low | High | Low | Some concerns | Some concerns | High |
| Fang Ruiping (2017). | 10.13241/j.cnki.pmb.2017.05.033 | Low | Low | Low | Low | Low | Low |
| Zhang Yang (2012). | NA | Low | High | Low | Some concerns | Some concerns | High |
| Zhang Gengchang (2013). | 10.3969/j.issn.1671-8194.2013.15.481 | Low | High | Low | Some concerns | Some concerns | High |
| Jiang Lianying (2023). | 10.7501/j.issn.1674-5515.2023.10.019 | Low | Some concerns | Low | Low | Low | Some concerns |
| Chen Qiaxin (2017). | DOI:10.13638/i.issn.1671-4040.2017.07.059 | Low | Some concerns | Low | Low | Low | Some concerns |
| Yang Na (2019). | 10.13683/j.wph.2019.12.008 | Low | Some concerns | Low | Low | Low | Some concerns |
| Wang Jiaping (2023). | 10.16840/j.issn1673-4297.2023.01.12 | Low | Some concerns | Low | Low | Low | Some concerns |
| Yi Peiyu (2020). | 10.11954/ytctyy.202007049 | Low | Some concerns | High | Low | Low | High |
| Huang Yong (2018). | 10.19613/j.cnki.1671-3141.2018.80.080 | Low | High | Low | Some concerns | Some concerns | High |
| Wang Yan (2017). | DOI:10.13407/j.cnki jpp.1672-108X.2017.03.010 | Low | High | Low | Some concerns | Some concerns | High |
| Chen Pingping (2022). | 10.15887/j.cnki.13-1389/r.2022.30.039 | Low | Some concerns | Low | Low | Low | Some concerns |
| Cui Long (2016). | 1000-3649(2016)06-0193-03 | High | Low | Low | Low | Low | High |
| Wang Qing (2021). | 10.3969/j.issn.1674-4721.2021.26.042 | Low | Some concerns | Low | Low | Low | Some concerns |
| Cao Zhihong (2014). | 1006-0979(2014)02-0019-02 | Low | High | Low | Some concerns | Some concerns | High |
| Chen Wenli (2024). | 10.3969/j.issn.1671-5217.2024.20.068 | Low | High | Low | Some concerns | Some concerns | High |
| Fu Quansheng (2023). | 10.7501/j.issn.1674-5515.2023.05.023 | Low | Some concerns | Low | Low | Low | Some concerns |
| Li Xiangyi (2010). | 10.3969/j.issn.1674-4721.2010.35.126 | Low | High | Low | Some concerns | Some concerns | High |
| Yu Xiaoyan (2017). | DOl:10.7501/i.issn.1674-5515.2017.07.029 | Low | Some concerns | Low | Low | Low | Some concerns |
| Zheng Meng (2023). | 10.7501/j.issn.1674-5515.2017.07.029 | Low | High | Low | Some concerns | Some concerns | High |
| Xu Sufen (2014). | 2095- 7629- （2014）19- 0172- 02 | Low | High | Low | Some concerns | Some concerns | High |
| Yang Zhihua (2018). | 10.3969/j.issn.1008-9926.2018.03.020 | Low | Some concerns | Low | Low | Low | Some concerns |
| Fan Ruimin (2013). | DOI:10.3969/i.issn.1672-2779.2013.11.026 | Low | High | Low | Some concerns | Some concerns | High |
| Wu Tingting (2018). | 10.16840/j.issn1673-4297.2018.06.14 | Low | Some concerns | Low | Low | Low | Some concerns |
| Wang Yinjia (2019). | NA | Low | Low | Low | Low | Low | Low |
| Cai Jianliang (2025). | 10.3969/j.issn.1672-8351.2025.01.046 | Low | Some concerns | Low | Low | Low | Some concerns |
| Ye Qing (2014). | 10.3969/j.issn.1006-1959.2014.22.285 | Some concerns | High | Low | Some concerns | Some concerns | High |
| Yang Mei (2021). | 10.14164/j.cnki.cn11-5581/r.2021.03.048 | Low | High | Low | Some concerns | Some concerns | High |
| Yu Meijia (2015). | 10.13192/j.issn.1000-1719.2015.05.038 | Low | Low | Low | Low | Low | Low |
| Chen Sihong (2008). | 10.3969/j.issn.1007-2349.2008.08.051 | Low | High | Low | Some concerns | Some concerns | High |

**Appendix 6: Evaluation of inconsistency and heterogeneity**

**Table S6.1:** Global consistency and Heterogeneity score

| **Clinical outcome** | **Chi square** | **P value** | **τ2 network** |
| --- | --- | --- | --- |
| Nasal obstruction | 2.12 | 0.1450 | 0.12 |
| Nasal pruritus | 2.71 | 0.0996 | 0.14 |
| Paroxysmal sneezing | 1.16 | 0.2822 | 0.14 |
| Nasal discharge | 1.08 | 0.2993 | 0.16 |
| IgE | 2.80 | 0.0944 | 0.18 |
| Effective rate | 33.56 | 0.0732 | 0.02 |
| Adverse events | 17.18 | 0.0646 | 0.16 |
| Recurrence Rate | 26.57 | 0.0583 | 0.13 |

**Table S6.2:** Side-splitting of Nasal obstruction. The test for the inconsistency of the results between the treatment plan combining traditional Chinese medicine with conventional Western medicine and the conventional Western medicine treatment plan.

| Comparison | Direct | | Indirect | | Difference | | |
| --- | --- | --- | --- | --- | --- | --- | --- |
|  | Coef. | Std. Err. | Coef. | Std. Err. | Coef. | Std. Err. | P>\|z\| |
| NCT+CWMT v.s. CWMT | 0.7505364 | 1.396494 | 0.359803 | 22.94377 | 0.3907334 | 22.98623 | 0.986 |
| BYTG+CWMT v.s. CWMT | 1.13938 | 0.5728193 | 0.3578997 | 81.70196 | 0.7814805 | 81.70404 | 0.992 |
| CBP+CWMT v.s. CWMT | -1.243453 | 0.9979212 | -2.742225 | 141.4624 | 1.498772 | 141.4657 | 0.992 |
| HG+CWMT v.s. CWMT | -1.100084 | 1.424435 | -2.598572 | 200.0135 | 1.498487 | 200.0181 | 0.994 |
| STDP+CWMT v.s. CWMT | -1.207932 | 1.406894 | -2.706395 | 200.0547 | 1.498463 | 200.0595 | 0.994 |
| TBG+CWMT v.s. CWMT | -1.790333 | 0.5373028 | -3.29056 | 75.65298 | 1.500227 | 75.65472 | 0.984 |
| XG+CWMT v.s. CWMT | -0.7167317 | 1.415277 | -2.215213 | 200.019 | 1.498481 | 200.0237 | 0.994 |
| YG+CWMT v.s. CWMT | -2.070658 | 1.420959 | -3.569145 | 200.0743 | 1.498487 | 200.0789 | 0.994 |

Abbreviations: CWMT , Conventional Western Medicine Treatment ;

NCT ,Nasal Comfort Tablets ;

BYTG , Biyuan Tongqiao Granules ;

CBP , Cang'er Zibi Yan Pills ;

HG , Huaiqi Huang Granules ;

STDP , Sanfeng Tongqiao Dropping Pills ;

TBG , Tongqiao Biyan Granules ;

XG , Xinqin Granules ;

YG , Yuping Feng Granules .

**Table S6.3:** Side-splitting of Nasal pruritus. The test for the inconsistency of the results between the treatment plan combining traditional Chinese medicine with conventional Western medicine and the conventional Western medicine treatment plan.

| Comparison | Direct | | Indirect | | Difference | | |
| --- | --- | --- | --- | --- | --- | --- | --- |
|  | Coef. | Std. Err. | Coef. | Std. Err. | Coef. | Std. Err. | P>\|z\| |
| NCT+CWMT v.s. CWMT | 0.5209435 | 0.6533325 | 0.1403696 | 25.81922 | 0.3805739 | 25.82749 | 0.988 |
| BYTG+CWMT v.s. CWMT | 0.7018519 | 0.3859386 | 0.3396446 | 115.4627 | 0.3622074 | 115.4634 | 0.997 |
| CBP+CWMT v.s. CWMT | -1.040674 | 0.4819199 | -2.082164 | 141.2987 | 1.041491 | 141.2993 | 0.994 |
| HG+CWMT v.s. CWMT | -0.1749711 | 0.7036123 | -1.216428 | 200.0281 | 1.041457 | 200.0289 | 0.996 |
| STDP+CWMT v.s. CWMT | -1.572022 | 0.6800986 | -2.613483 | 200.0109 | 1.041461 | 200.0118 | 0.996 |
| TBG+CWMT v.s. CWMT | -1.195904 | 0.2810174 | -2.237565 | 81.71778 | 1.041661 | 81.71813 | 0.99 |
| XG+CWMT v.s. CWMT | -0.80156 | 0.6942927 | -1.843007 | 199.9685 | 1.041447 | 199.9693 | 0.996 |
| YG+CWMT v.s. CWMT | -1.134444 | 0.6889745 | -2.175921 | 199.8185 | 1.041477 | 199.8194 | 0.996 |

Abbreviations: CWMT , Conventional Western Medicine Treatment ;

NCT ,Nasal Comfort Tablets ;

BYTG , Biyuan Tongqiao Granules ;

CBP , Cang'er Zibi Yan Pills ;

HG , Huaiqi Huang Granules ;

STDP , Sanfeng Tongqiao Dropping Pills ;

TBG , Tongqiao Biyan Granules ;

XG , Xinqin Granules ;

YG , Yuping Feng Granules .

**Table S6.4:** Side-splitting of Paroxysmal sneezing. The test for the inconsistency of the results between the treatment plan combining traditional Chinese medicine with conventional Western medicine and the conventional Western medicine treatment plan.

| Comparison | Direct | | Indirect | | Difference | | |
| --- | --- | --- | --- | --- | --- | --- | --- |
|  | Coef. | Std. Err. | Coef. | Std. Err. | Coef. | Std. Err. | P>\|z\| |
| NCT+CWMT v.s. CWMT | 0.6818157 | 1.575983 | 0.3237198 | 23.57284 | 0.3580959 | 23.62546 | 0.988 |
| BYTG+CWMT v.s. CWMT | 1.165394 | 0.7077043 | 0.1938521 | 89.50406 | 0.9715424 | 89.50694 | 0.991 |
| CBP+CWMT v.s. CWMT | -0.9319111 | 1.122168 | -2.29303 | 141.443 | 1.361119 | 141.4472 | 0.992 |
| HG+CWMT v.s. CWMT | -0.4323595 | 1.597619 | -1.793142 | 200.0276 | 1.360782 | 200.0335 | 0.995 |
| STDP+CWMT v.s. CWMT | -1.410582 | 1.586024 | -2.771364 | 200.0453 | 1.360782 | 200.0513 | 0.995 |
| TBG+CWMT v.s. CWMT | -2.089951 | 0.6061929 | -3.452754 | 75.66904 | 1.362803 | 75.67127 | 0.986 |
| XG+CWMT v.s. CWMT | -0.90149 | 1.59344 | -2.262272 | 200.0699 | 1.360782 | 200.0759 | 0.995 |
| YG+CWMT v.s. CWMT | -1.714649 | 1.594504 | -3.075431 | 200.0462 | 1.360782 | 200.0521 | 0.995 |

Abbreviations: CWMT , Conventional Western Medicine Treatment ;

NCT ,Nasal Comfort Tablets ;

BYTG , Biyuan Tongqiao Granules ;

CBP , Cang'er Zibi Yan Pills ;

HG , Huaiqi Huang Granules ;

STDP , Sanfeng Tongqiao Dropping Pills ;

TBG , Tongqiao Biyan Granules ;

XG , Xinqin Granules ;

YG , Yuping Feng Granules .

**Table S6.5:** Side-splitting of Nasal discharge . The test for the inconsistency of the results between the treatment plan combining traditional Chinese medicine with conventional Western medicine and the conventional Western medicine treatment plan.

| Comparison | Direct | | Indirect | | Difference | | |
| --- | --- | --- | --- | --- | --- | --- | --- |
|  | Coef. | Std. Err. | Coef. | Std. Err. | Coef. | Std. Err. | P>\|z\| |
| NCT+CWMT v.s. CWMT | 0.6999124 | 1.630996 | 0.3132425 | 23.57307 | 0.3866698 | 23.62943 | 0.987 |
| BYTG+CWMT v.s. CWMT | 0.9397306 | 0.6682055 | 0.4556486 | 81.72462 | 0.484082 | 81.72743 | 0.995 |
| CBP+CWMT v.s. CWMT | -1.00658 | 1.162246 | -2.403461 | 141.4522 | 1.396881 | 141.4567 | 0.992 |
| HG+CWMT v.s. CWMT | -0.1964995 | 1.651311 | -1.593011 | 200.0688 | 1.396511 | 200.0752 | 0.994 |
| STDP+CWMT v.s. CWMT | -0.0366484 | 1.637447 | -1.43316 | 200.0459 | 1.396512 | 200.0524 | 0.994 |
| TBG+CWMT v.s. CWMT | -1.881339 | 0.6761517 | -3.279701 | 81.71407 | 1.398362 | 81.71666 | 0.986 |
| XG+CWMT v.s. CWMT | -0.8513783 | 1.647559 | -2.24789 | 200.0451 | 1.396512 | 200.0515 | 0.994 |
| YG+CWMT v.s. CWMT | -1.711237 | 1.648803 | -3.107748 | 200.0951 | 1.396511 | 200.1015 | 0.994 |

Abbreviations: CWMT , Conventional Western Medicine Treatment ;

NCT ,Nasal Comfort Tablets ;

BYTG , Biyuan Tongqiao Granules ;

CBP , Cang'er Zibi Yan Pills ;

HG , Huaiqi Huang Granules ;

STDP , Sanfeng Tongqiao Dropping Pills ;

TBG , Tongqiao Biyan Granules ;

XG , Xinqin Granules ;

YG , Yuping Feng Granules .

**Table S6.6:** Side-splitting of IgE. The test for the inconsistency of the results between the treatment plan combining traditional Chinese medicine with conventional Western medicine and the conventional Western medicine treatment plan.

| Comparison | Direct | | Indirect | | Difference | | |
| --- | --- | --- | --- | --- | --- | --- | --- |
|  | Coef. | Std. Err. | Coef. | Std. Err. | Coef. | Std. Err. | P>\|z\| |
| BYSOL+CWMT v.s. CWMT | 0.9360019 | 20.29475 | 0.0478229 | 30.76574 | 0.888179 | 36.85658 | 0.981 |
| BYTG+CWMT v.s. CWMT | 0.5260532 | 19.8042 | 0.8330231 | 205.7075 | -0.3069698 | 206.6588 | 0.999 |
| STDP+CWMT v.s. CWMT | -1.32148 | 19.80445 | -2.709385 | 205.7077 | 1.387905 | 206.6586 | 0.995 |
| TBG+CWMT v.s. CWMT | -0.6245283 | 19.80454 | -2.012433 | 205.7075 | 1.387905 | 206.6584 | 0.995 |
| XC+CWMT v.s. CWMT | -0.7967848 | 19.80411 | -2.18469 | 205.7073 | 1.387905 | 206.6582 | 0.995 |
| XG+CWMT v.s. CWMT | -19.28838 | 19.82376 | -20.67629 | 205.7096 | 1.387905 | 206.6587 | 0.995 |
| YG+CWMT v.s. CWMT | -13.48031 | 8.210993 | -15.08426 | 90.60697 | 1.603947 | 90.97039 | 0.986 |

Abbreviations: CWMT , Conventional Western Medicine Treatment ;

BYSOL , Biyuan Shu Oral Liquid;

BYTG , Biyuan Tongqiao Granules ;

STDP , Sanfeng Tongqiao Dropping Pills ;

TBG , Tongqiao Biyan Granules ;

XC , Xiangju Capsules ;

XG , Xinqin Granules ;

YG , Yuping Feng Granules .

**Table S6.7:** Side-splitting of Effective rate. The test for the inconsistency of the results between the treatment plan combining traditional Chinese medicine with conventional Western medicine and the conventional Western medicine treatment plan.

| Comparison | Direct | | Indirect | | Difference | | |
| --- | --- | --- | --- | --- | --- | --- | --- |
|  | Coef. | Std. Err. | Coef. | Std. Err. | Coef. | Std. Err. | P>\|z\| |
| NCT+CWMT v.s. CWMT | -0.081493 | 0.0693172 | -0.0244857 | 14.85885 | -0.0570073 | 14.85902 | 0.997 |
| BYSOL+CWMT v.s. CWMT | -0.147636 | 0.0929516 | -0.0152601 | 197.5152 | -0.1323759 | 197.5152 | 0.999 |
| BYTG+CWMT v.s. CWMT | -0.1398546 | 0.0333528 | -0.0231269 | 75.41936 | -0.1167278 | 75.41938 | 0.999 |
| BZYQP+CWMT v.s. CWMT | 0.0833816 | 0.0813604 | 0.2464493 | 200.843 | -0.1630677 | 200.843 | 0.999 |
| CBP+CWMT v.s. CWMT | 0.2262629 | 0.0876903 | 0.3892549 | 143.2107 | -0.162992 | 143.2106 | 0.999 |
| DYG+CWMT v.s. CWMT | 0.1365755 | 0.0905121 | 0.3054814 | 197.113 | -0.1689058 | 197.113 | 0.999 |
| HG+CWMT v.s. CWMT | 0.2152695 | 0.1499169 | 0.3782524 | 201.1312 | -0.1629829 | 201.1311 | 0.999 |
| LQG+CWMT v.s. CWMT | 0.1397619 | 0.1042371 | 0.3054488 | 200.3355 | -0.1656869 | 200.3355 | 0.999 |
| STDP+CWMT v.s. CWMT | 0.0953102 | 0.1052102 | 0.2587451 | 202.1225 | -0.1634349 | 202.1225 | 0.999 |
| TBG+CWMT v.s. CWMT | 0.156527 | 0.0341939 | 0.3250377 | 64.65057 | -0.1685107 | 64.65057 | 0.998 |
| XC+CWMT v.s. CWMT | 0.1617636 | 0.0568519 | 0.3238289 | 120.6087 | -0.1620653 | 120.6087 | 0.999 |
| XG+CWMT v.s. CWMT | 0.1880779 | 0.0604255 | 0.3505073 | 121.2589 | -0.1624293 | 121.2589 | 0.999 |
| YG+CWMT v.s. CWMT | 0.1619644 | 0.0279573 | 0.3245622 | 49.22337 | -0.1625978 | 49.22336 | 0.997 |

Abbreviations:CWMT , Conventional Western Medicine Treatment ;

NCT ,Nasal Comfort Tablets ;

BYSOL , Biyuan Shu Oral Liquid;

BYTG , Biyuan Tongqiao Granules ;

BZYQP , Buzhong Yiqi Pills ;

CBP , Cang'er Zibi Yan Pills ;

DYG , Danxi Yuping Feng Granules ;

HG , Huaiqi Huang Granules ;

LQG , Lianhua Qingwen Granules ;

STDP , Sanfeng Tongqiao Dropping Pills ;

TBG , Tongqiao Biyan Granules ;

XC , Xiangju Capsules ;

XG , Xinqin Granules ;

YG , Yuping Feng Granules .

**Table S6.8:** Side-splitting of Adverse events. The test for the inconsistency of the results between the treatment plan combining traditional Chinese medicine with conventional Western medicine and the conventional Western medicine treatment plan.

| Comparison | Direct | | Indirect | | Difference | | |
| --- | --- | --- | --- | --- | --- | --- | --- |
|  | Coef. | Std. Err. | Coef. | Std. Err. | Coef. | Std. Err. | P>\|z\| |
| BYSOL+CWMT v.s. CWMT | 1.223775 | 0.8047843 | 0.1158786 | 17.67876 | 1.107897 | 17.69706 | 0.95 |
| BYTG+CWMT v.s. CWMT | 0.6180605 | 0.3147417 | 1.825478 | 81.67812 | -1.207418 | 81.67962 | 0.988 |
| BZYQP+CWMT v.s. CWMT | 2.276474 | 1.556674 | -0.1666523 | 200.0094 | 2.443126 | 200.0041 | 0.99 |
| CBP+CWMT v.s. CWMT | 4.70e-11 | 2.055948 | -2.443126 | 200.0077 | 2.443126 | 199.998 | 0.99 |
| LQG+CWMT v.s. CWMT | 4.44e-11 | 2.049434 | -2.443126 | 200.0141 | 2.443126 | 200.0044 | 0.99 |
| STDP+CWMT v.s. CWMT | -0.3091883 | 0.8875221 | -2.752315 | 200.0765 | 2.443126 | 200.0754 | 0.99 |
| TBG+CWMT v.s. CWMT | -0.5182805 | 0.2789644 | -2.962513 | 70.7504 | 2.444232 | 70.75013 | 0.972 |
| XC+CWMT v.s. CWMT | -0.7321115 | 0.6893195 | -3.175396 | 141.394 | 2.443285 | 141.3929 | 0.986 |
| XG+CWMT v.s. CWMT | 5.07e-11 | 1.475905 | -2.443127 | 200.0029 | 2.443127 | 199.9982 | 0.99 |
| YG+CWMT v.s. CWMT | -1.119082 | 0.2699763 | -3.563789 | 60.34471 | 2.444706 | 60.34434 | 0.968 |

Abbreviations: CWMT , Conventional Western Medicine Treatment ;

BYSOL , Biyuan Shu Oral Liquid;

BYTG , Biyuan Tongqiao Granules ;

BZYQP , Buzhong Yiqi Pills ;

CBP , Cang'er Zibi Yan Pills ;

LQG , Lianhua Qingwen Granules ;

STDP , Sanfeng Tongqiao Dropping Pills ;

TBG , Tongqiao Biyan Granules ;

XC , Xiangju Capsules ;

XG , Xinqin Granules ;

YG , Yuping Feng Granules .

**Table S6.9:** Side-splitting of Recurrence Rate. The test for the inconsistency of the results between the treatment plan combining traditional Chinese medicine with conventional Western medicine and the conventional Western medicine treatment plan.

| Comparison | Direct | | Indirect | | Difference | | |
| --- | --- | --- | --- | --- | --- | --- | --- |
|  | Coef. | Std. Err. | Coef. | Std. Err. | Coef. | Std. Err. | P>\|z\| |
| BYTG+CWMT v.s. CWMT | 0.7013196 | 0.2708301 | 0.8200519 | 25.82168 | -0.1187323 | 25.82299 | 0.996 |
| DYG+CWMT v.s. CWMT | 1.034896 | 0.4831078 | 0.3677982 | 199.8111 | 0.6670983 | 199.8128 | 0.997 |
| HG+CWMT v.s. CWMT | 1.442384 | 0.692206 | -0.0397142 | 200.0335 | 1.482098 | 200.037 | 0.994 |
| TBG+CWMT v.s. CWMT | 0.6376667 | 0.3393145 | 0.7650144 | 141.4854 | -0.1273477 | 141.4866 | 0.999 |
| XG+CWMT v.s. CWMT | -0.1643031 | 0.5835316 | 1.56711 | 200.0528 | -1.731413 | 200.0553 | 0.993 |
| YG+CWMT v.s. CWMT | 0.8712469 | 0.1690348 | 0.5314048 | 63.25194 | 0.339842 | 63.25256 | 0.996 |

Abbreviations: CWMT , Conventional Western Medicine Treatment ;

BYTG , Biyuan Tongqiao Granules ;

DYG , Danxi Yuping Feng Granules ;

HG , Huaiqi Huang Granules ;

TBG , Tongqiao Biyan Granules ;

XG , Xinqin Granules ;

YG , Yuping Feng Granules .

**Appendix 7: Network maps and forest plots of secondary outcomes**

**Figure S7.1:** Network map of the effect on Recurrence Rate, and forest plot of network effect sizes for compared with Conventional Western Medicine Treatment. The size of the nodes was proportional to the number of participants included in the trial, and the thickness of lines between the interventions relates to the number of studies for that comparison.Results are expressed as Odds Ratio (OR) and 95% confidence intervals (CI), derived from a random-effects model.An OR <1 favors the intervention, indicating a higher response rate compared to the control. The size of the data markers corresponds to the relative weight of each comparison in the network.


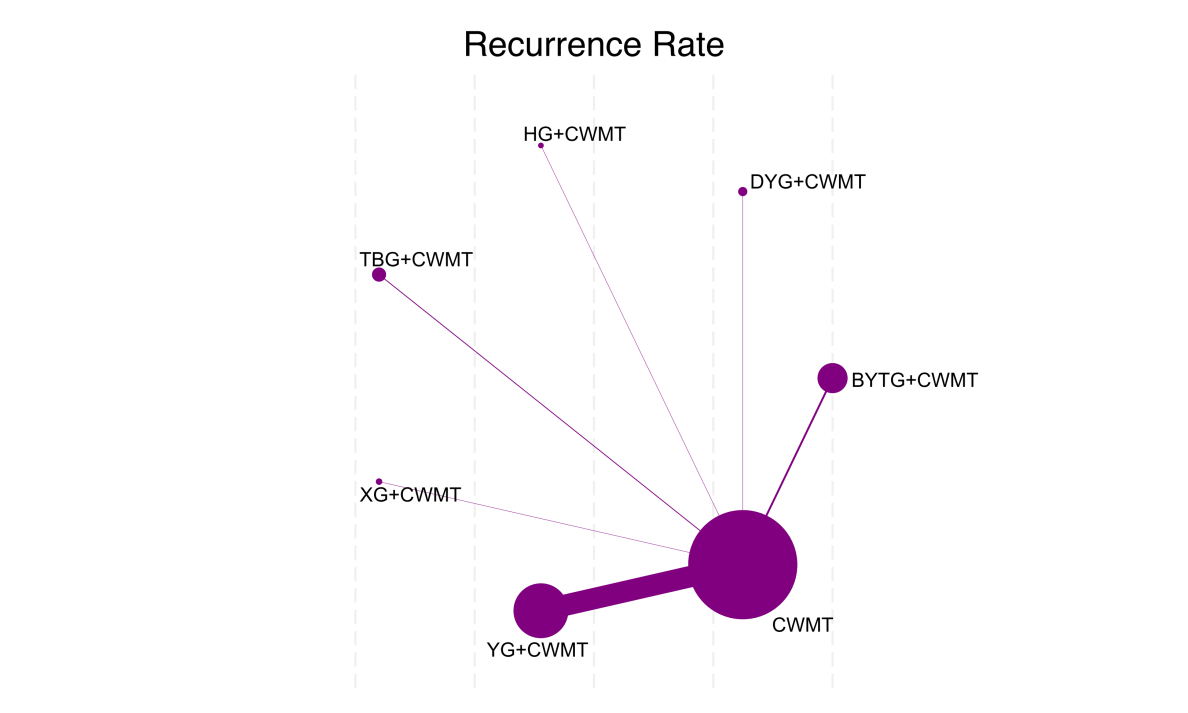

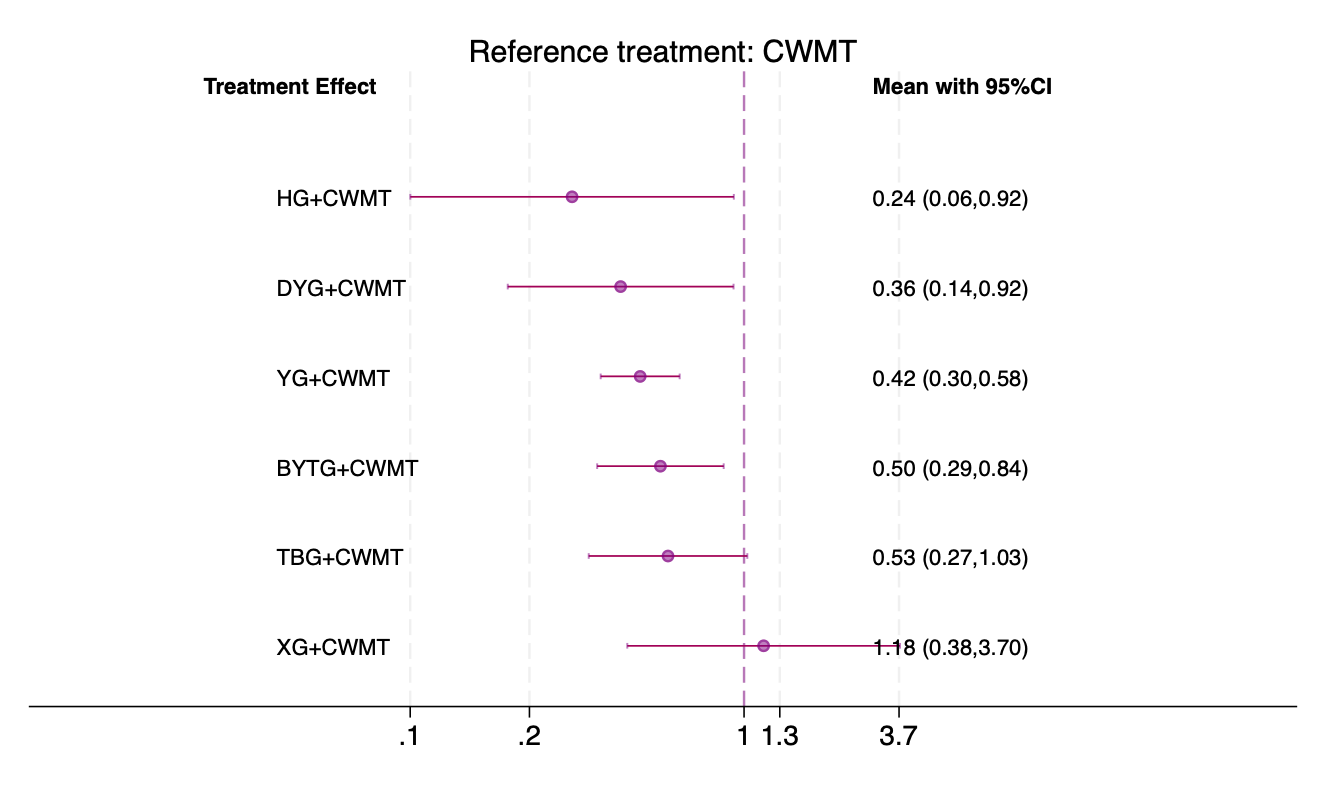


Abbreviations: CWMT , Conventional Western Medicine Treatment ;

BYTG , Biyuan Tongqiao Granules ;

DYG , Danxi Yuping Feng Granules ;

HG , Huaiqi Huang Granules ;

TBG , Tongqiao Biyan Granules ;

XG , Xinqin Granules ;

YG , Yuping Feng Granules .

**Appendix 8: SUCRA and cumulative probability plots**

**Figure S8.1:** Cumulative ranking curve plots of nasal obstruction symptoms for different Chinese patent medicines combined with conventional Western medicine in treating pediatric allergic rhinitis within the scope of the network. The larger the area under the curve, the more effective the treatment.


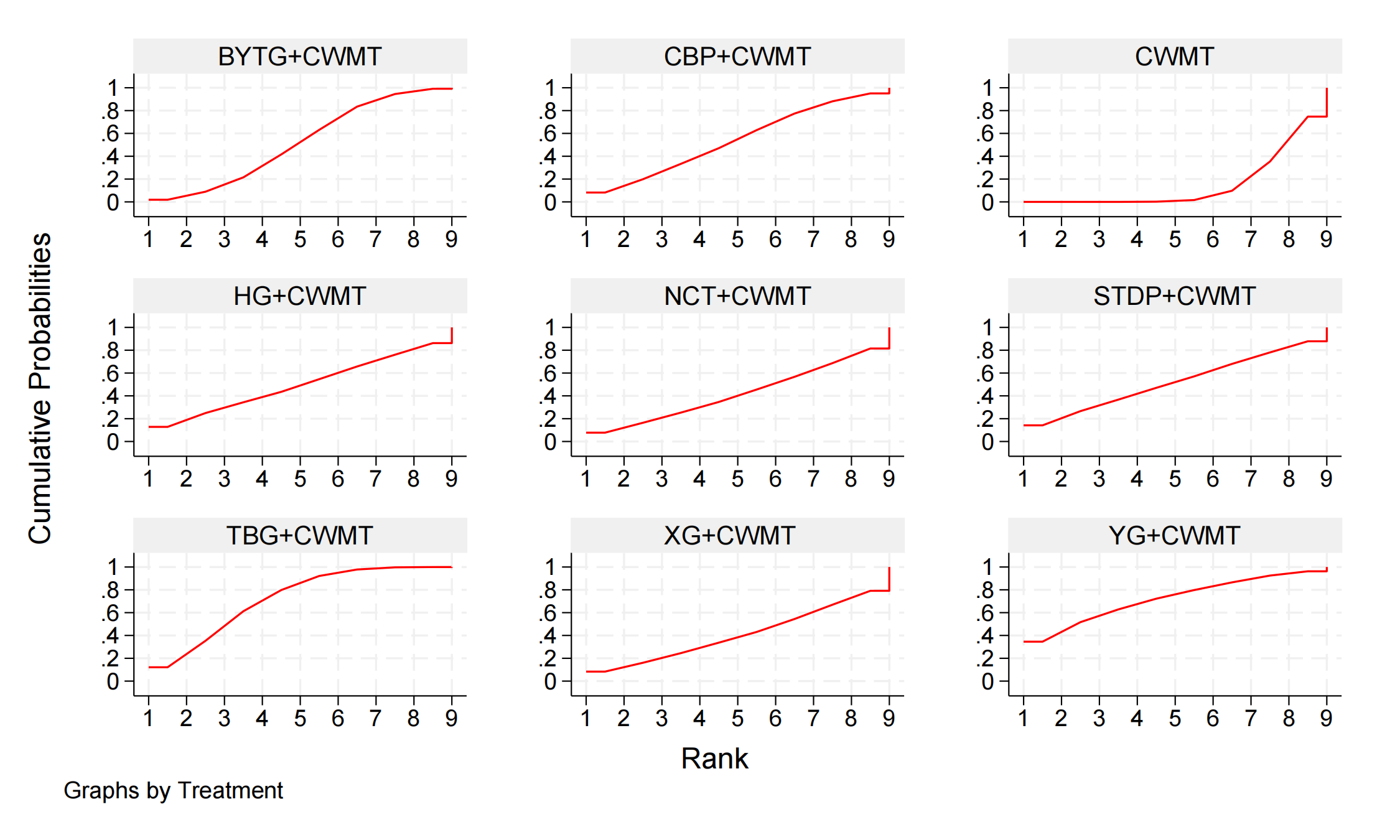


**Table S8.1:** SUCRA rankings of nasal obstruction improvement for various Chinese patent medicines combined with conventional Western medicine in treating pediatric allergic rhinitis.

| **Treatment** | **SUCRA** | **PrBest** | | **MeanRank** |
| --- | --- | --- | --- | --- |
| TBG+CWMT | 72.3 | 12.2 | 3.2 | |
| YG+CWMT | 72.1 | 34.6 | 3.2 | |
| CBP+CWMT | 54 | 8.2 | 4.7 | |
| STDP+CWMT | 51.9 | 14.2 | 4.8 | |
| BYTG+CWMT | 51.8 | 1.9 | 4.9 | |
| HG+CWMT | 49.8 | 12.8 | 5 | |
| NCT+CWMT | 42.1 | 7.8 | 5.6 | |
| XG+CWMT | 40.8 | 8.4 | 5.7 | |
| CWMT | 15.2 | 0 | 7.8 | |

Abbreviations: SUCRA, surface under the cumulative ranking curve;

CWMT , Conventional Western Medicine Treatment ;

NCT ,Nasal Comfort Tablets ;

BYTG , Biyuan Tongqiao Granules ;

CBP , Cang'er Zibi Yan Pills ;

HG , Huaiqi Huang Granules ;

STDP , Sanfeng Tongqiao Dropping Pills ;

TBG , Tongqiao Biyan Granules ;

XG , Xinqin Granules ;

YG , Yuping Feng Granules .

**Figure S8.2:** Cumulative ranking curve plots of nasal pruritus relief for different Chinese patent medicines combined with conventional Western medicine in treating pediatric allergic rhinitis within the network scope. The larger the area under the curve, the more effective the treatment.


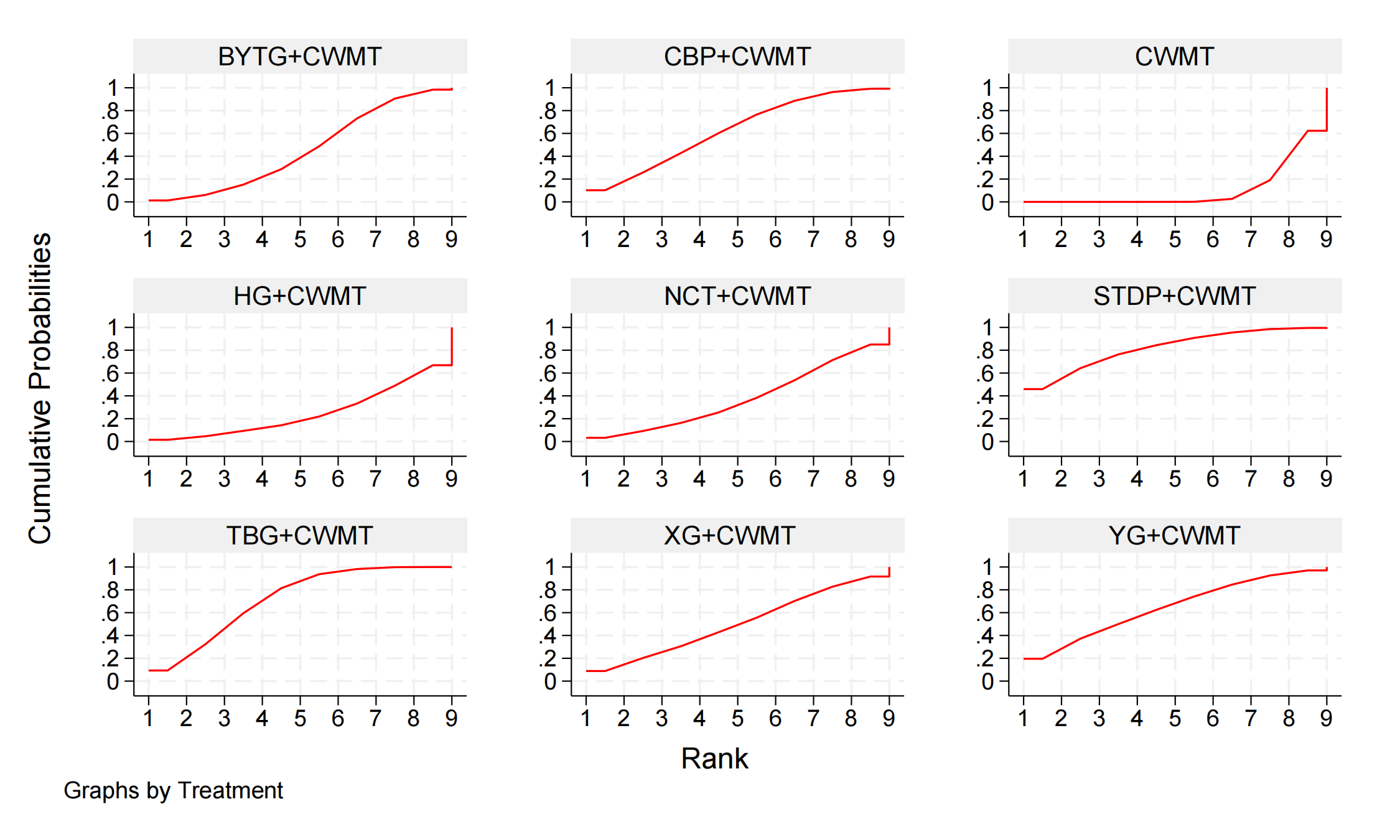


**Table S8.2:** SUCRA rankings of nasal pruritus improvement for various Chinese patent medicines combined with conventional Western medicine in treating pediatric allergic rhinitis.

| **Treatment** | **SUCRA** | **PrBest** | **MeanRank** |
| --- | --- | --- | --- |
| STDP+CWMT | 81.9 | 45.9 | 2.4 |
| TBG+CWMT | 71.8 | 9.3 | 3.3 |
| YG+CWMT | 64.7 | 19.6 | 3.8 |
| CBP+CWMT | 62.5 | 10.2 | 4 |
| XG+CWMT | 50.4 | 8.9 | 5 |
| BYTG+CWMT | 45.3 | 1.3 | 5.4 |
| NCT+CWMT | 37.8 | 3.3 | 6 |
| HG+CWMT | 25.1 | 1.5 | 7 |
| CWMT | 10.5 | 0 | 8.2 |

Abbreviations: SUCRA, surface under the cumulative ranking curve;

CWMT , Conventional Western Medicine Treatment ;

NCT ,Nasal Comfort Tablets ;

BYTG , Biyuan Tongqiao Granules ;

CBP , Cang'er Zibi Yan Pills ;

HG , Huaiqi Huang Granules ;

STDP , Sanfeng Tongqiao Dropping Pills ;

TBG , Tongqiao Biyan Granules ;

XG , Xinqin Granules ;

YG , Yuping Feng Granules .

**Figure S8.3:** Cumulative ranking curve plots of sneezing symptom relief for different Chinese patent medicines combined with conventional Western medicine in treating pediatric allergic rhinitis within the network scope. The larger the area under the curve, the more effective the treatment.


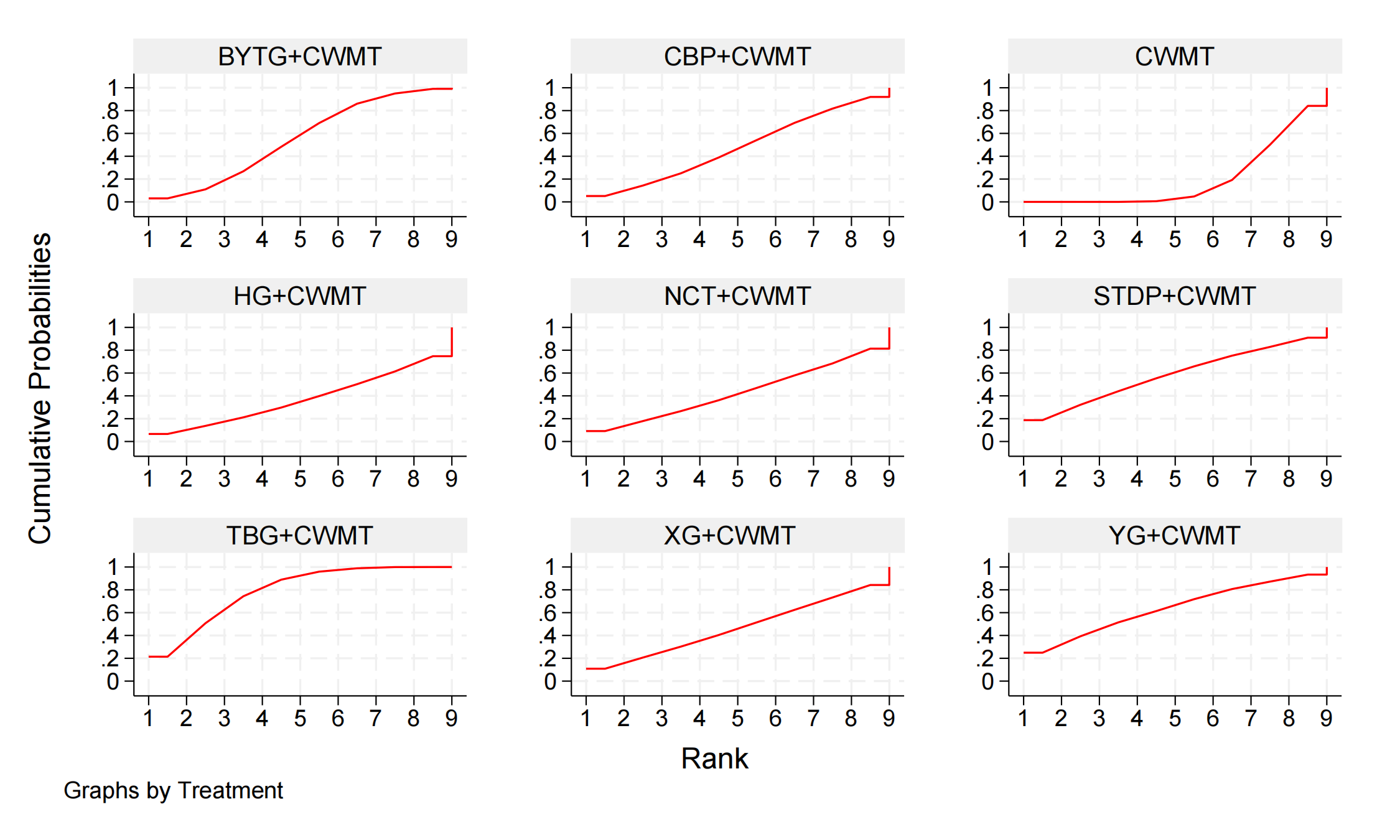


**Table S8.3:** SUCRA rankings of sneezing symptom improvement for various Chinese patent medicines combined with conventional Western medicine in treating pediatric allergic rhinitis.

| **Treatment** | **SUCRA** | **PrBest** | **MeanRank** |
| --- | --- | --- | --- |
| TBG+CWMT | 78.8 | 21.4 | 2.7 |
| YG+CWMT | 63.8 | 24.9 | 3.9 |
| STDP+CWMT | 58.2 | 18.7 | 4.3 |
| BYTG+CWMT | 54.8 | 3.1 | 4.6 |
| CBP+CWMT | 47.6 | 5.1 | 5.2 |
| XG+CWMT | 46.7 | 10.9 | 5.3 |
| NCT+CWMT | 43.1 | 9.1 | 5.6 |
| HG+CWMT | 37.2 | 6.6 | 6 |
| CWMT | 19.9 | 0 | 7.4 |

Abbreviations: SUCRA, surface under the cumulative ranking curve;

CWMT , Conventional Western Medicine Treatment ;

NCT ,Nasal Comfort Tablets ;

BYTG , Biyuan Tongqiao Granules ;

CBP , Cang'er Zibi Yan Pills ;

HG , Huaiqi Huang Granules ;

STDP , Sanfeng Tongqiao Dropping Pills ;

TBG , Tongqiao Biyan Granules ;

XG , Xinqin Granules ;

YG , Yuping Feng Granules .

**Figure S8.4:** Cumulative ranking curve plots of Nasal discharge relief for different Chinese patent medicines combined with conventional Western medicine in treating pediatric allergic rhinitis within the network scope. The larger the area under the curve, the more effective the treatment.


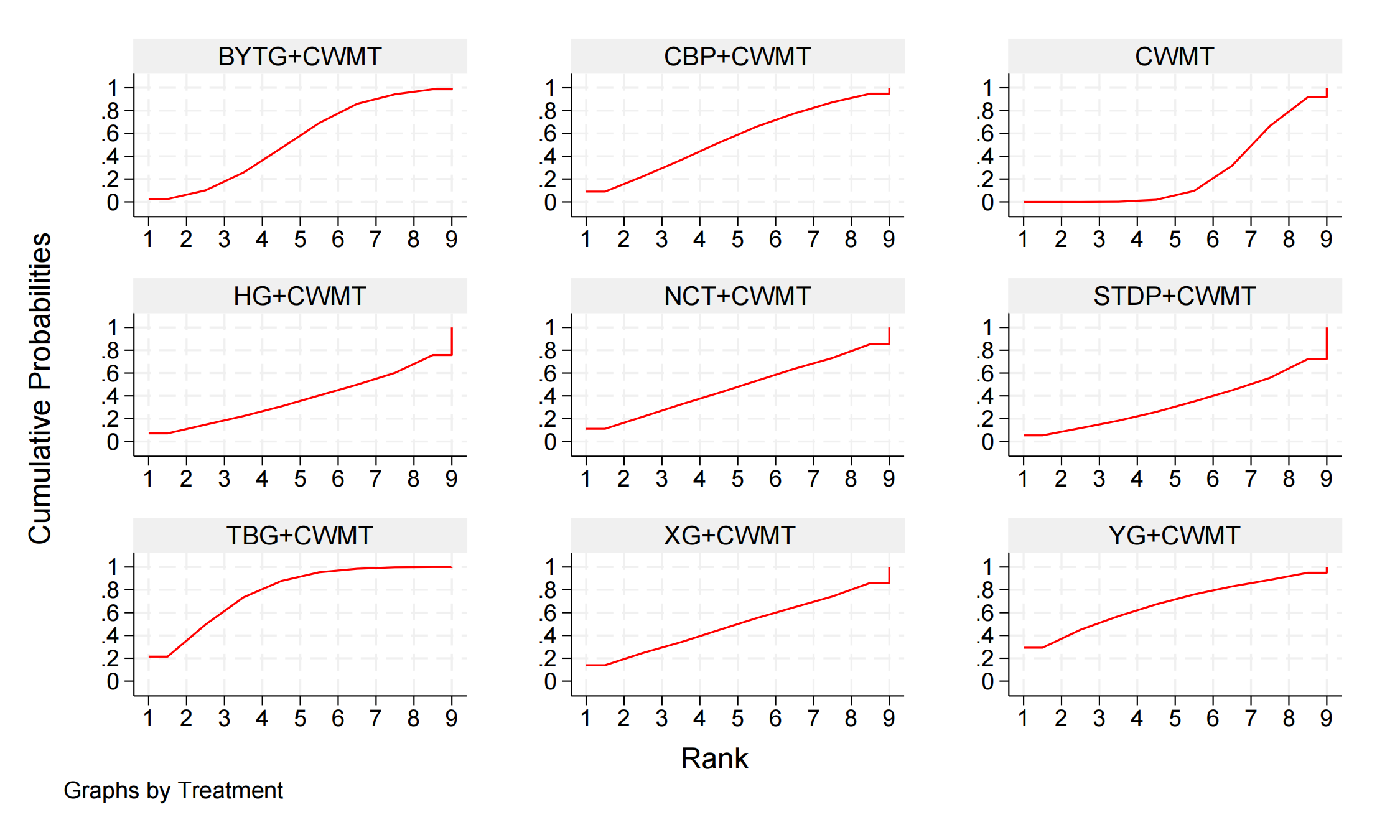


**Table S8.4:**SUCRA rankings of Nasal discharge improvement for various Chinese patent medicines combined with conventional Western medicine in treating pediatric allergic rhinitis.

| **Treatment** | **SUCRA** | **PrBest** | **MeanRank** |
| --- | --- | --- | --- |
| TBG+CWMT | 78.2 | 21.5 | 2.7 |
| YG+CWMT | 67.7 | 29.3 | 3.6 |
| CBP+CWMT | 55.7 | 9.1 | 4.5 |
| BYTG+CWMT | 54.2 | 2.5 | 4.7 |
| XG+CWMT | 49.7 | 14 | 5 |
| NCT+CWMT | 47.9 | 11.2 | 5.2 |
| HG+CWMT | 37.6 | 7.1 | 6 |
| STDP+CWMT | 33.7 | 5.4 | 6.3 |
| CWMT | 25.2 | 0 | 7 |

Abbreviations: SUCRA, surface under the cumulative ranking curve;

CWMT , Conventional Western Medicine Treatment ;

NCT ,Nasal Comfort Tablets ;

BYTG , Biyuan Tongqiao Granules ;

CBP , Cang'er Zibi Yan Pills ;

HG , Huaiqi Huang Granules ;

STDP , Sanfeng Tongqiao Dropping Pills ;

TBG , Tongqiao Biyan Granules ;

XG , Xinqin Granules ;

YG , Yuping Feng Granules .

**Figure S8.5:** Cumulative ranking curve plots of IgE level reduction for different Chinese patent medicines combined with conventional Western medicine in treating pediatric allergic rhinitis within the network scope. The larger the area under the curve, the more effective the treatment.


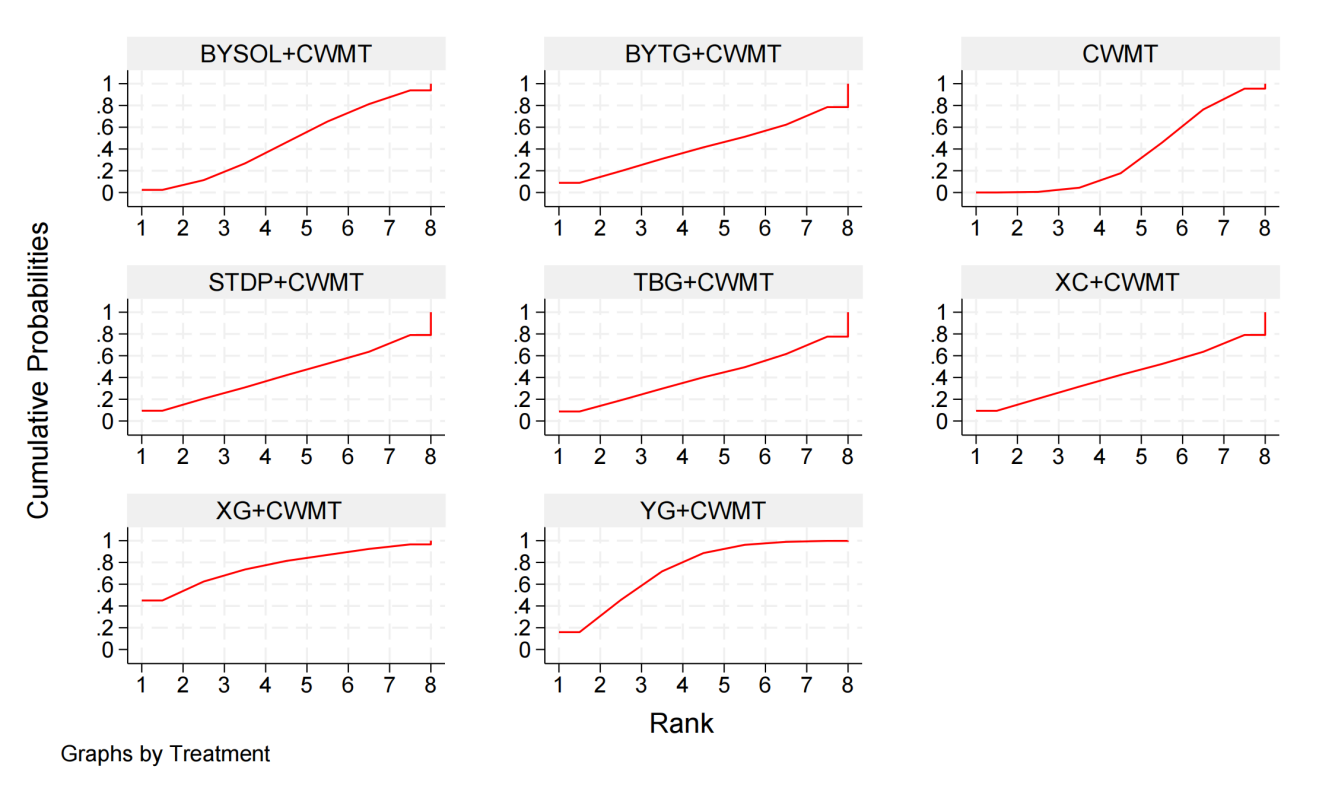


**Table S8.5:** SUCRA rankings of IgE levels for various Chinese patent medicines combined with conventional Western medicine in treating pediatric allergic rhinitis.

| **Treatment** | **SUCRA** | **PrBest** | **MeanRank** |
| --- | --- | --- | --- |
| XG+CWMT | 76.9 | 45.1 | 2.6 |
| YG+CWMT | 73.8 | 15.9 | 2.8 |
| BYSOL+CWMT | 46.7 | 2.4 | 4.7 |
| XC+CWMT | 42.7 | 9.4 | 5 |
| STDP+CWMT | 42.6 | 9.5 | 5 |
| BYTG+CWMT | 41.9 | 8.9 | 5.1 |
| TBG+CWMT | 40.9 | 8.8 | 5.1 |
| CWMT | 34.3 | 0 | 5.6 |

Abbreviations: SUCRA, surface under the cumulative ranking curve;

CWMT , Conventional Western Medicine Treatment ;

BYSOL , Biyuan Shu Oral Liquid;

BYTG , Biyuan Tongqiao Granules ;

STDP , Sanfeng Tongqiao Dropping Pills ;

TBG , Tongqiao Biyan Granules ;

XC , Xiangju Capsules ;

XG , Xinqin Granules ;

YG , Yuping Feng Granules .

**Figure S8.6:** Cumulative ranking curve plots of the efficacy rates of different Chinese patent medicines combined with conventional Western medicine treatments of pediatric allergic rhinitis, within the scope of the network. The larger the area under the curve, the higher the effective rate.


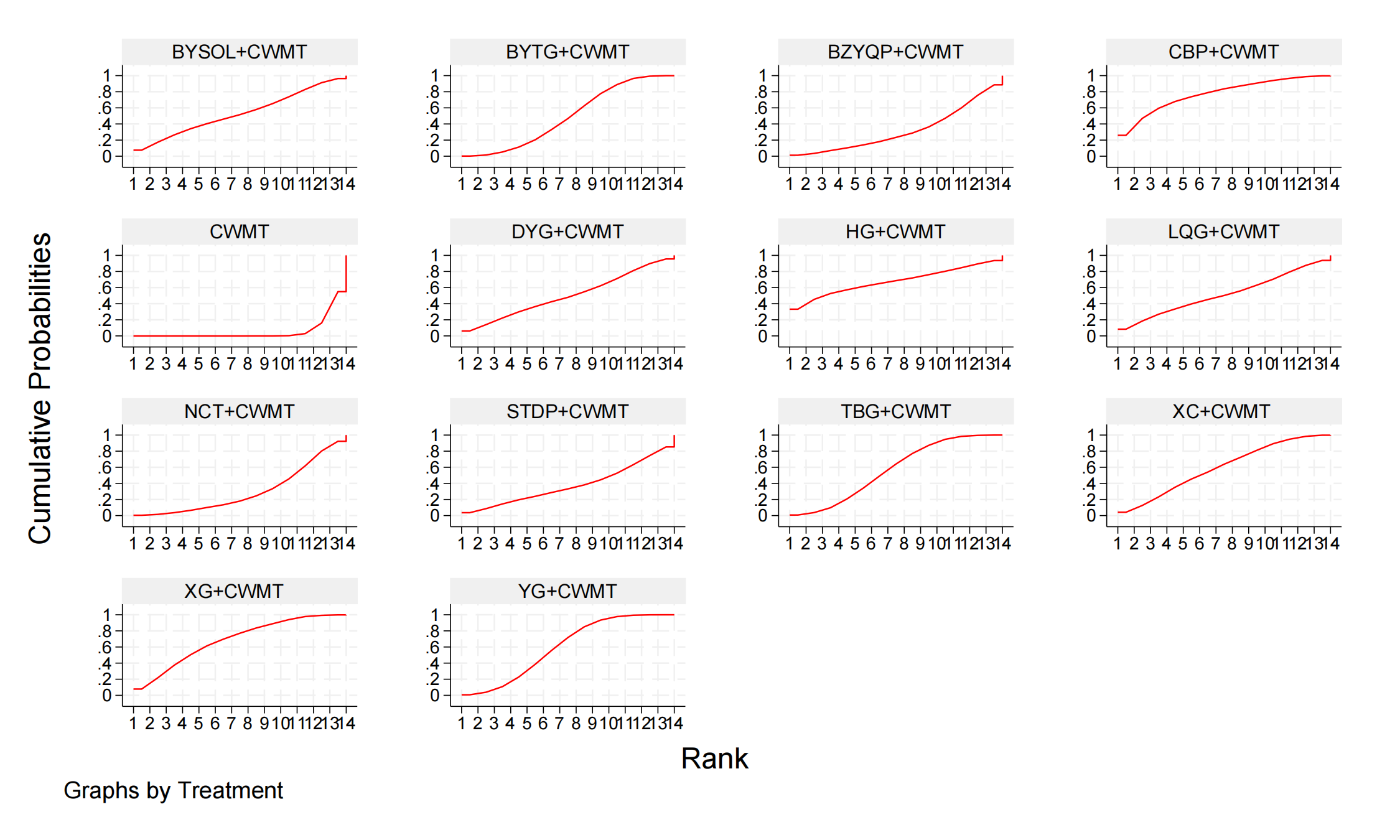


**Table S8.6:** SUCRA of the effective rates of various Chinese patent medicines combined with conventional Western medicine treatments of pediatric allergic rhinitis.

| **Treatment** | **SUCRA** | **PrBest** | **MeanRank** |
| --- | --- | --- | --- |
| CBP+CWMT | 77.2 | 25.9 | 4 |
| XG+CWMT | 68.4 | 7.8 | 5.1 |
| HG+CWMT | 67.7 | 33.2 | 5.2 |
| YG+CWMT | 60 | 0.7 | 6.2 |
| XC+CWMT | 59.6 | 4.2 | 6.3 |
| TBG+CWMT | 56.9 | 0.8 | 6.6 |
| BYSOL+CWMT | 53.2 | 7.5 | 7.1 |
| LQG+CWMT | 51.7 | 8.4 | 7.3 |
| DYG+CWMT | 50.3 | 6.2 | 7.5 |
| BYTG+CWMT | 49.5 | 0.2 | 7.6 |
| STDP+CWMT | 37.7 | 3.6 | 9.1 |
| BZYQP+CWMT | 31.9 | 1.2 | 9.9 |
| NCT+CWMT | 30.1 | 0.4 | 10.1 |
| CWMT | 5.7 | 0 | 13.3 |

Abbreviations:SUCRA, surface under the cumulative ranking curve;

CWMT , Conventional Western Medicine Treatment ;

NCT ,Nasal Comfort Tablets ;

BYSOL , Biyuan Shu Oral Liquid;

BYTG , Biyuan Tongqiao Granules ;

BZYQP , Buzhong Yiqi Pills ;

CBP , Cang'er Zibi Yan Pills ;

DYG , Danxi Yuping Feng Granules ;

HG , Huaiqi Huang Granules ;

LQG , Lianhua Qingwen Granules ;

STDP , Sanfeng Tongqiao Dropping Pills ;

TBG , Tongqiao Biyan Granules ;

XC , Xiangju Capsules ;

XG , Xinqin Granules ;

YG , Yuping Feng Granules

**Figure S8.7:** Cumulative ranking curve plots of adverse events for different Chinese patent medicines combined with conventional Western medicine in the treatment of pediatric allergic rhinitis,within the scope of the network. The smaller the area under the curve, the safer the medication.


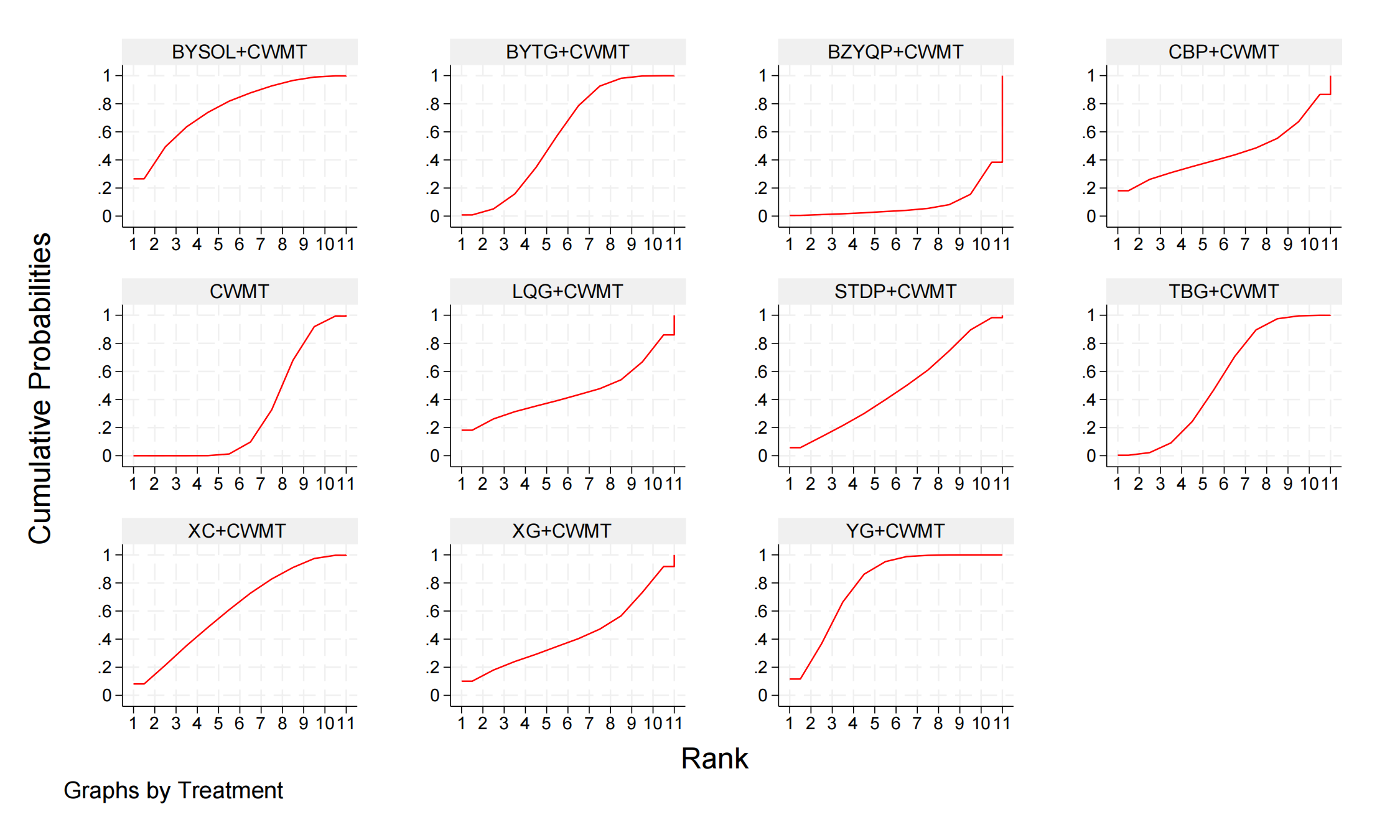


**Table S8.7:** SUCRA rankings of adverse events for various Chinese patent medicines combined with conventional Western medicine in treating pediatric allergic rhinitis.

| **Treatment** | **SUCRA** | **PrBest** | **MeanRank** |
| --- | --- | --- | --- |
| YG+CWMT | 79.5 | 11.6 | 3.1 |
| BYSOL+CWMT | 77.1 | 26.5 | 3.3 |
| XC+CWMT | 61.8 | 8.1 | 4.8 |
| BYTG+CWMT | 58.3 | 0.8 | 5.2 |
| TBG+CWMT | 54 | 0.3 | 5.6 |
| STDP+CWMT | 48.4 | 5.7 | 6.2 |
| CBP+CWMT | 45.1 | 18.1 | 6.5 |
| LQG+CWMT | 44.8 | 18.2 | 6.5 |
| XG+CWMT | 42.5 | 10.1 | 6.7 |
| CWMT | 30.3 | 0 | 8 |
| BZYQP+CWMT | 8.1 | 0.5 | 10.2 |

Abbreviations: SUCRA, surface under the cumulative ranking curve;

CWMT , Conventional Western Medicine Treatment ;

BYSOL , Biyuan Shu Oral Liquid;

BYTG , Biyuan Tongqiao Granules ;

BZYQP , Buzhong Yiqi Pills ;

CBP , Cang'er Zibi Yan Pills ;

LQG , Lianhua Qingwen Granules ;

STDP , Sanfeng Tongqiao Dropping Pills ;

TBG , Tongqiao Biyan Granules ;

XC , Xiangju Capsules ;

XG , Xinqin Granules ;

YG , Yuping Feng Granules.

**Figure S8.8:** Cumulative ranking curve plots of recurrence rate for different Chinese patent medicines combined with conventional Western medicine in the treatment of pediatric allergic rhinitis,within the scope of the network. The smaller the area under the curve, the safer the medication.


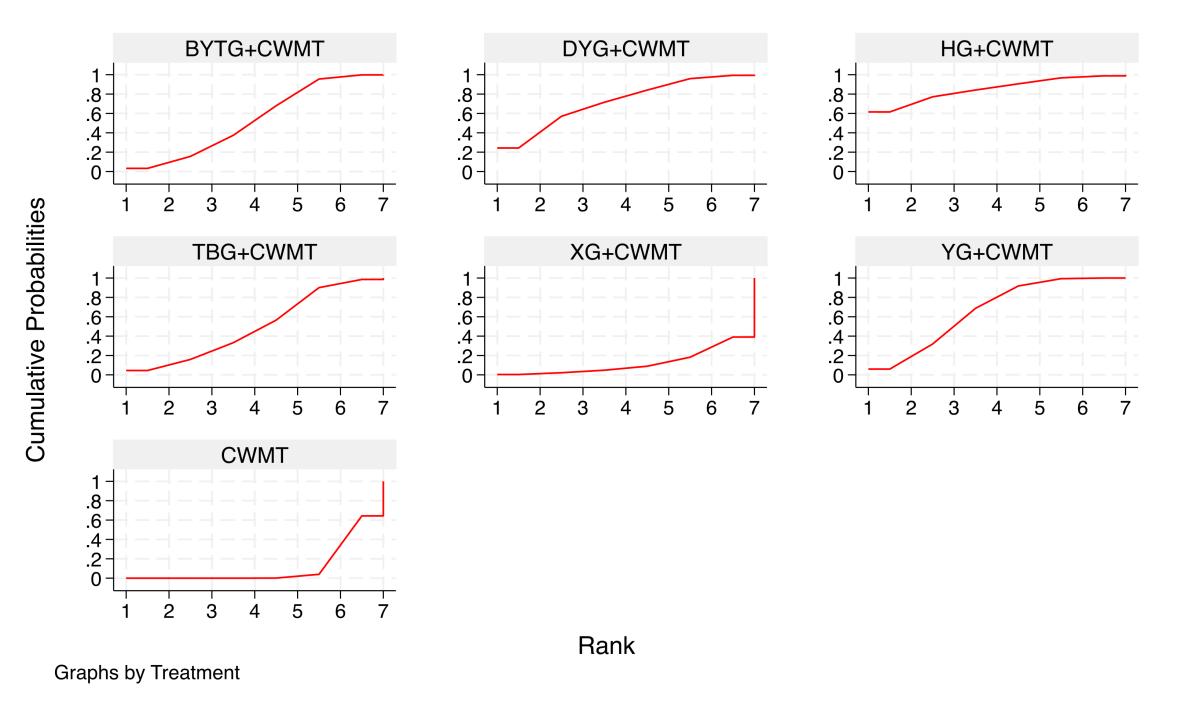


**Table S8.8:** SUCRA rankings of recurrence rate for various Chinese patent medicines combined with conventional Western medicine in treating pediatric allergic rhinitis.

| **Treatment** | **SUCRA** | **PrBest** | **MeanRank** |
| --- | --- | --- | --- |
| HG+CWMT | 84.8 | 61.6 | 1.9 |
| DYG+CWMT | 72.1 | 24.3 | 2.7 |
| YG+CWMT | 66.3 | 6 | 3 |
| BYTG+CWMT | 53.3 | 3.3 | 3.8 |
| TBG+CWMT | 49.8 | 4.5 | 4 |
| XG+CWMT | 12.3 | 0.4 | 6.3 |
| CWMT | 11.4 | 0 | 6.3 |

Abbreviations: CWMT , Conventional Western Medicine Treatment ;

BYTG , Biyuan Tongqiao Granules ;

DYG , Danxi Yuping Feng Granules ;

HG , Huaiqi Huang Granules ;

TBG , Tongqiao Biyan Granules ;

XG , Xinqin Granules ;

YG , Yuping Feng Granules .

**Appendix 9:League table of summary estimates for different Chinese patent medicines combined with conventional Western medicine in treating pediatric allergic rhinitis, derived from network meta-analysis of 49 trials.**

**Table S9.1:** Nasal obstruction

The columns represent the comparison of the row drug class to the column drug class. The rows represent the comparison of the row drug class to the column drug class. The effect estimates are expressed as mean difference and 95% confidence interval. For example,the standardized mean difference in nasal obstruction score for Nasal Comfort Tablets + CWMT compared to Biyuan Tongqiao Granules + CWMT is 0.39 (95% confidence interval -2.51 to 3.29). Mean difference <0 favors the drug in the column, and mean difference >0 favors the drug in the row.

| Nasal Comfort Tablets + CWMT |  |  |  |  |  |  |  |  |
| --- | --- | --- | --- | --- | --- | --- | --- | --- |
| 0.39 (-2.51,3.29) | Biyuan Tongqiao Granules + CWMT |  |  |  |  |  |  |  |
| -0.75 (-3.43,1.93) | -1.14 (-2.26,-0.02) | CWMT |  |  |  |  |  |  |
| 0.49 (-2.82,3.81) | 0.10 (-2.14,2.35) | 1.24 (-0.71,3.19) | Cang'er Zibi Yan Pills + CWMT |  |  |  |  |  |
| 0.35 (-3.51,4.21) | -0.04 (-3.04,2.96) | 1.10 (-1.68,3.88) | -0.14 (-3.54,3.26) | Huaiqi Huang Granules + CWMT |  |  |  |  |
| 0.46 (-3.38,4.30) | 0.07 (-2.90,3.04) | 1.21 (-1.54,3.96) | -0.04 (-3.41,3.34) | 0.11 (-3.80,4.02) | Sanfeng Tongqiao Dropping Pills + CWMT |  |  |  |
| 1.04 (-1.84,3.92) | 0.65 (-0.88,2.19) | 1.79 (0.74,2.84) | 0.55 (-1.67,2.76) | 0.69 (-2.29,3.66) | 0.58 (-2.36,3.52) | Tongqiao Biyan Granules + CWMT |  |  |
| -0.03 (-3.88,3.82) | -0.42 (-3.41,2.56) | 0.72 (-2.05,3.48) | -0.53 (-3.91,2.86) | -0.38 (-4.31,3.54) | -0.49 (-4.39,3.41) | -1.07 (-4.03,1.89) | Xinqin Granules + CWMT |  |
| 1.32 (-2.54,5.18) | 0.93 (-2.06,3.92) | 2.07 (-0.71,4.85) | 0.83 (-2.57,4.22) | 0.97 (-2.96,4.90) | 0.86 (-3.05,4.77) | 0.28 (-2.69,3.25) | 1.35 (-2.57,5.27) | Yuping Feng Granules + CWMT |

Abbreviations: CWMT , Conventional Western Medicine Treatment.

**Table S9.2:** Nasal pruritus

The columns represent the comparison of the row drug class to the column drug class. The rows represent the comparison of the row drug class to the column drug class. The effect estimates are expressed as mean difference and 95% confidence interval. For example, the standardized mean difference in nasal pruritus score for Nasal Comfort Tablets + CWMT compared to Biyuan Tongqiao Granules + CWMT is 0.18 (95% confidence interval -1.30 to 1.66). Mean difference <0 favors the drug in the column, and mean difference >0 favors the drug in the row.

| Nasal Comfort Tablets + CWMT |  |  |  |  |  |  |  |  |
| --- | --- | --- | --- | --- | --- | --- | --- | --- |
| 0.18 (-1.30,1.66) | Biyuan Tongqiao Granules +CWMT |  |  |  |  |  |  |  |
| -0.52 (-1.80,0.75) | -0.70 (-1.46,0.05) | CWMT |  |  |  |  |  |  |
| 0.52 (-1.07,2.11) | 0.34 (-0.87,1.55) | 1.04 (0.10,1.98) | Cang'er Zibi Yan Pills + CWMT |  |  |  |  |  |
| -0.35 (-2.22,1.53) | -0.53 (-2.10,1.04) | 0.18 (-1.20,1.55) | -0.87 (-2.54,0.80) | Huaiqi Huang Granules +CWMT |  |  |  |  |
| 1.05 (-0.79,2.89) | 0.87 (-0.66,2.40) | 1.57 (0.24,2.90) | 0.53 (-1.10,2.16) | 1.40 (-0.52,3.31) | Sanfeng Tongqiao Dropping Pills + CWMT |  |  |  |
| 0.68 (-0.71,2.06) | 0.49 (-0.44,1.43) | 1.20 (0.65,1.75) | 0.16 (-0.94,1.25) | 1.02 (-0.46,2.50) | -0.38 (-1.82,1.07) | Tongqiao Biyan Granules +CWMT |  |  |
| 0.28 (-1.58,2.14) | 0.10 (-1.46,1.66) | 0.80 (-0.56,2.16) | -0.24 (-1.89,1.42) | 0.63 (-1.31,2.56) | -0.77 (-2.67,1.13) | -0.39 (-1.86,1.07) | Xinqin Granules + CWMT |  |
| 0.61 (-1.24,2.47) | 0.43 (-1.11,1.98) | 1.13 (-0.21,2.48) | 0.09 (-1.55,1.74) | 0.96 (-0.97,2.89) | -0.44 (-2.33,1.46) | -0.06 (-1.52,1.40) | 0.33 (-1.58,2.25) | Yuping Feng Granules +CWMT |

Abbreviations: CWMT , Conventional Western Medicine Treatment.

**Table S9.3:** Paroxysmal sneezing

The columns represent the comparison of the row drug class to the column drug class. The rows represent the comparison of the row drug class to the column drug class. The effect estimates are expressed as mean difference and 95% confidence interval. For example, the standardized mean difference in sneezing score for Nasal Comfort Tablets + CWMT compared to Biyuan Tongqiao Granules + CWMT is 0.48 (95% confidence interval -2.83 to 3.79). Mean difference <0 favors the drug in the column, and mean difference >0 favors the drug in the row.

| Nasal Comfort Tablets + CWMT |  |  |  |  |  |  |  |  |
| --- | --- | --- | --- | --- | --- | --- | --- | --- |
| 0.48 (-2.83,3.79) | Biyuan Tongqiao Granules+CWMT |  |  |  |  |  |  |  |
| -0.68 (-3.70,2.33) | -1.17 (-2.55,0.22) | CWMT |  |  |  |  |  |  |
| 0.25 (-3.47,3.97) | -0.23 (-2.82,2.36) | 0.93 (-1.26,3.12) | Cang'er Zibi Yan Pills + CWMT |  |  |  |  |  |
| -0.25 (-4.58,4.08) | -0.73 (-4.14,2.68) | 0.43 (-2.69,3.55) | -0.50 (-4.31,3.31) | Huaiqi Huang Granules+CWMT |  |  |  |  |
| 0.73 (-3.59,5.04) | 0.25 (-3.15,3.64) | 1.41 (-1.69,4.51) | 0.48 (-3.31,4.27) | 0.98 (-3.42,5.37) | Sanfeng Tongqiao Dropping Pills + CWMT |  |  |  |
| 1.40 (-1.83,4.64) | 0.92 (-0.90,2.74) | 2.09 (0.91,3.27) | 1.16 (-1.33,3.65) | 1.66 (-1.68,4.99) | 0.68 (-2.64,3.99) | Tongqiao Biyan Granules+CWMT |  |  |
| 0.22 (-4.11,4.54) | -0.26 (-3.67,3.14) | 0.90 (-2.21,4.01) | -0.03 (-3.84,3.77) | 0.47 (-3.94,4.87) | -0.51 (-4.90,3.88) | -1.19 (-4.52,2.14) | Xinqin Granules + CWMT |  |
| 1.03 (-3.30,5.36) | 0.55 (-2.86,3.95) | 1.71 (-1.40,4.83) | 0.78 (-3.02,4.59) | 1.28 (-3.13,5.69) | 0.30 (-4.09,4.69) | -0.37 (-3.71,2.96) | 0.81 (-3.59,5.21) | Yuping Feng Granules+CWMT |

Abbreviations: CWMT , Conventional Western Medicine Treatment.

**Table S9.4:** Nasal discharge

The columns represent the comparison of the row drug class to the column drug class. The rows represent the comparison of the row drug class to the column drug class. The effect estimates are expressed as mean difference and 95% confidence interval. For example, the standardized mean difference in Nasal discharge score for Nasal Comfort Tablets + CWMT compared to Biyuan Tongqiao Granules + CWMT is 0.24 (95% confidence interval -3.12 to 3..61). SMD <0 favors the drug in the column, and SMD >0 favors the drug in the row.

| Nasal Comfort Tablets + CWMT |  |  |  |  |  |  |  |  |
| --- | --- | --- | --- | --- | --- | --- | --- | --- |
| 0.24 (-3.12,3.61) | Biyuan Tongqiao Granules+CWMT |  |  |  |  |  |  |  |
| -0.70 (-3.81,2.42) | -0.94 (-2.24,0.36) | CWMT |  |  |  |  |  |  |
| 0.31 (-3.54,4.16) | 0.07 (-2.55,2.68) | 1.01 (-1.26,3.28) | Cang'er Zibi Yan Pills + CWMT |  |  |  |  |  |
| -0.50 (-4.97,3.98) | -0.74 (-4.22,2.73) | 0.20 (-3.03,3.42) | -0.81 (-4.75,3.13) | Huaiqi Huang Granules+CWMT |  |  |  |  |
| -0.66 (-5.11,3.80) | -0.90 (-4.35,2.55) | 0.04 (-3.16,3.23) | -0.97 (-4.89,2.95) | -0.16 (-4.70,4.38) | Sanfeng Tongqiao Dropping Pills + CWMT |  |  |  |
| 1.19 (-2.19,4.56) | 0.94 (-0.91,2.80) | 1.88 (0.56,3.20) | 0.87 (-1.75,3.50) | 1.68 (-1.80,5.17) | 1.84 (-1.61,5.30) | Tongqiao Biyan Granules+CWMT |  |  |
| 0.16 (-4.32,4.63) | -0.09 (-3.56,3.38) | 0.85 (-2.36,4.07) | -0.16 (-4.09,3.78) | 0.65 (-3.90,5.21) | 0.81 (-3.72,5.35) | -1.03 (-4.50,2.45) | Xinqin Granules + CWMT |  |
| 1.02 (-3.46,5.49) | 0.77 (-2.70,4.24) | 1.71 (-1.51,4.93) | 0.70 (-3.23,4.64) | 1.51 (-3.04,6.07) | 1.67 (-2.86,6.21) | -0.17 (-3.65,3.31) | 0.86 (-3.69,5.41) | Yuping Feng Granules+CWMT |

Abbreviations: CWMT , Conventional Western Medicine Treatment.

**Table S9.5:** IgE

The columns represent the comparison of the row drug class to the column drug class. The rows represent the comparison of the row drug class to the column drug class. The effect estimates are expressed as mean difference and 95% confidence interval. For example, the standardized mean difference in IgE levels for Biyuan Shu Oral Liquid+ CWMT compared to Biyuan Tongqiao Granules + CWMT is -2.68 (95% confidence interval -38.13 to 32.78). SMD <0 favors the drug in the row, and SMD >0 favors the drug in the column.

| Biyuan Shu Oral Liquid+ CWMT |  |  |  |  |  |  |  |
| --- | --- | --- | --- | --- | --- | --- | --- |
| -2.68 (-38.13,32.78) | Biyuan Tongqiao Granules + CWMT |  |  |  |  |  |  |
| -3.56 (-21.31,14.19) | -0.88 (-32.68,30.91) | CWMT |  |  |  |  |  |
| -1.92 (-37.38,33.53) | 0.75 (-44.13,45.63) | 1.64 (-30.16,33.43) | Sanfeng Tongqiao Dropping Pills + CWMT |  |  |  |  |
| -2.58 (-38.04,32.87) | 0.09 (-44.79,44.97) | 0.98 (-30.82,32.77) | -0.66 (-45.54,44.22) | Tongqiao Biyan Granules + CWMT |  |  |  |
| -2.42 (-37.88,33.04) | 0.26 (-44.62,45.14) | 1.14 (-30.66,32.94) | -0.50 (-45.38,44.38) | 0.16 (-44.72,45.04) | Xiangju Capsules + CWMT |  |  |
| 15.07 (-20.44,50.57) | 17.74 (-27.17,62.66) | 18.63 (-13.21,50.46) | 16.99 (-27.93,61.91) | 17.65 (-27.27,62.57) | 17.49 (-27.43,62.40) | Xinqin Granules + CWMT |  |
| 9.43 (-11.05,29.91) | 12.10 (-22.21,46.41) | 12.99 (-0.21,26.18) | 11.35 (-22.96,45.66) | 12.01 (-22.30,46.32) | 11.85 (-22.47,46.16) | -5.64 (-40.00,28.72) | Yuping Feng Granules + CWMT |

Abbreviations: CWMT , Conventional Western Medicine Treatment.

**Table S9.6:** Effective rate

The columns represent the comparison of the row drug class to the column drug class. The rows represent the comparison of the row drug class to the column drug class. The effect estimates are expressed as mean difference and 95% confidence interval. For example, the odds ratio for Nasal Comfort Tablets + CWMT compared to Biyuan Shu Oral Liquid+ CWMT is 0.94 (95% confidence interval 0.75 to 1.17). Odds ratio <1 favors the drug in the row, and odds ratio >1 favors the drug in the column.

| Nasal Comfort Tablets + CWMT |  |  |  |  |  |  |  |  |  |  |  |  |  |
| --- | --- | --- | --- | --- | --- | --- | --- | --- | --- | --- | --- | --- | --- |
| 0.94 (0.75,1.17) | Biyuan Shu Oral Liquid+ CWMT |  |  |  |  |  |  |  |  |  |  |  |  |
| 0.94 (0.81,1.10) | 1.01 (0.83,1.22) | Biyuan Tongqiao Granules +CWMT |  |  |  |  |  |  |  |  |  |  |  |
| 1.08 (0.95,1.24) | 1.16 (0.97,1.39) | 1.15 (1.08,1.23) | CWMT |  |  |  |  |  |  |  |  |  |  |
| 1.00 (0.81,1.23) | 1.07 (0.84,1.36) | 1.06 (0.89,1.26) | 0.92 (0.78,1.08) | Buzhong Yiqi Pills + CWMT |  |  |  |  |  |  |  |  |  |
| 0.87 (0.70,1.08) | 0.92 (0.72,1.19) | 0.92 (0.76,1.10) | 0.80 (0.67,0.95) | 0.87 (0.69,1.10) | Cang'er Zibi Yan Pills + CWMT |  |  |  |  |  |  |  |  |
| 0.95 (0.76,1.18) | 1.01 (0.78,1.30) | 1.00 (0.83,1.21) | 0.87 (0.73,1.04) | 0.95 (0.75,1.20) | 1.09 (0.85,1.40) | Danxi Yuping Feng Granules +CWMT |  |  |  |  |  |  |  |
| 0.87 (0.63,1.21) | 0.93 (0.66,1.32) | 0.93 (0.69,1.25) | 0.81 (0.60,1.08) | 0.88 (0.63,1.22) | 1.01 (0.72,1.42) | 0.92 (0.66,1.30) | Huaiqi Huang Granules +CWMT |  |  |  |  |  |  |
| 0.94 (0.74,1.21) | 1.01 (0.77,1.33) | 1.00 (0.81,1.24) | 0.87 (0.71,1.07) | 0.95 (0.73,1.22) | 1.09 (0.83,1.42) | 1.00 (0.76,1.31) | 1.08 (0.75,1.54) | Lianhua Qingwen Granules +CWMT |  |  |  |  |  |
| 0.99 (0.77,1.26) | 1.05 (0.80,1.39) | 1.05 (0.84,1.30) | 0.91 (0.74,1.12) | 0.99 (0.76,1.28) | 1.14 (0.87,1.49) | 1.04 (0.79,1.37) | 1.13 (0.79,1.61) | 1.05 (0.78,1.40) | Sanfeng Tongqiao Dropping Pills + CWMT |  |  |  |  |
| 0.93 (0.80,1.08) | 0.99 (0.82,1.20) | 0.98 (0.90,1.08) | 0.86 (0.80,0.91) | 0.93 (0.78,1.10) | 1.07 (0.89,1.29) | 0.98 (0.81,1.18) | 1.06 (0.78,1.43) | 0.98 (0.79,1.22) | 0.94 (0.76,1.17) | Tongqiao Biyan Granules +CWMT |  |  |  |
| 0.92 (0.77,1.10) | 0.99 (0.80,1.22) | 0.98 (0.86,1.11) | 0.85 (0.76,0.95) | 0.92 (0.76,1.12) | 1.07 (0.87,1.31) | 0.98 (0.79,1.20) | 1.05 (0.77,1.44) | 0.98 (0.78,1.23) | 0.94 (0.74,1.18) | 0.99 (0.87,1.13) | Xiangju Capsules + CWMT |  |  |
| 0.90 (0.75,1.08) | 0.96 (0.77,1.19) | 0.95 (0.83,1.09) | 0.83 (0.74,0.93) | 0.90 (0.74,1.10) | 1.04 (0.84,1.28) | 0.95 (0.77,1.18) | 1.03 (0.75,1.41) | 0.95 (0.75,1.21) | 0.91 (0.72,1.16) | 0.97 (0.85,1.11) | 0.97 (0.83,1.15) | Xinqin Granules + CWMT |  |
| 0.92 (0.80,1.07) | 0.99 (0.82,1.19) | 0.98 (0.90,1.06) | 0.85 (0.81,0.90) | 0.92 (0.78,1.09) | 1.07 (0.89,1.28) | 0.97 (0.81,1.17) | 1.05 (0.78,1.42) | 0.98 (0.79,1.21) | 0.94 (0.76,1.16) | 0.99 (0.92,1.08) | 1.00 (0.88,1.13) | 1.03 (0.90,1.17) | Yuping Feng Granules + CWMT |

Abbreviations: CWMT , Conventional Western Medicine Treatment.

**Table S9.7:** Adverse events

The columns represent the comparison of the row drug class to the column drug class. The rows represent the comparison of the row drug class to the column drug class. The effect estimates are expressed as mean difference and 95% confidence interval. For example, the odds ratio for adverse effects of Biyuan Shu Oral Liquid+ CWMT compared to Biyuan Tongqiao Granules + CWMT is 0.55 (95% confidence interval 0.10 to 2.97). Odds ratio <1 favors the drug in the row, and odds ratio >1 favors the drug in the column.

| Biyuan Shu Oral Liquid+ CWMT |  |  |  |  |  |  |  |  |  |  |
| --- | --- | --- | --- | --- | --- | --- | --- | --- | --- | --- |
| 0.55 (0.10,2.97) | Biyuan Tongqiao Granules + CWMT |  |  |  |  |  |  |  |  |  |
| 0.29 (0.06,1.42) | 0.54 (0.29,1.00) | CWMT |  |  |  |  |  |  |  |  |
| 0.03 (0.00,0.94) | 0.06 (0.00,1.24) | 0.10 (0.00,2.17) | Buzhong Yiqi Pills + CWMT |  |  |  |  |  |  |  |
| 0.29 (0.00,22.30) | 0.54 (0.01,31.77) | 1.00 (0.02,56.24) | 9.74 (0.06,1526.10) | Cang'er Zibi Yan Pills + CWMT |  |  |  |  |  |  |
| 0.29 (0.00,22.04) | 0.54 (0.01,31.37) | 1.00 (0.02,55.52) | 9.74 (0.06,1510.65) | 1.00 (0.00,295.78) | Lianhua Qingwen Granules + CWMT |  |  |  |  |  |
| 0.40 (0.04,4.20) | 0.73 (0.12,4.65) | 1.36 (0.24,7.76) | 13.27 (0.40,444.69) | 1.36 (0.02,109.74) | 1.36 (0.02,108.47) | Sanfeng Tongqiao Dropping Pills + CWMT |  |  |  |  |
| 0.49 (0.09,2.62) | 0.91 (0.40,2.06) | 1.68 (0.97,2.90) | 16.35 (0.74,362.86) | 1.68 (0.03,97.98) | 1.68 (0.03,96.75) | 1.23 (0.20,7.63) | Tongqiao Biyan Granules + CWMT |  |  |  |
| 0.61 (0.08,4.88) | 1.12 (0.25,4.95) | 2.08 (0.54,8.03) | 20.25 (0.72,569.63) | 2.08 (0.03,145.78) | 2.08 (0.03,144.03) | 1.53 (0.17,13.81) | 1.24 (0.29,5.32) | Xiangju Capsules + CWMT |  |  |
| 0.29 (0.01,7.94) | 0.54 (0.03,10.38) | 1.00 (0.06,18.04) | 9.74 (0.15,652.28) | 1.00 (0.01,142.64) | 1.00 (0.01,141.17) | 0.73 (0.03,21.46) | 0.60 (0.03,11.31) | 0.48 (0.02,11.71) | Xinqin Granules + CWMT |  |
| 0.90 (0.17,4.75) | 1.65 (0.74,3.71) | 3.06 (1.80,5.20) | 29.82 (1.35,659.68) | 3.06 (0.05,178.25) | 3.06 (0.05,176.02) | 2.25 (0.36,13.85) | 1.82 (0.86,3.88) | 1.47 (0.35,6.28) | 3.06 (0.16,57.96) | Yuping Feng Granules + CWMT |

Abbreviations: CWMT , Conventional Western Medicine Treatment.

**Table S9.8:** Recurrence Rate

The columns represent the comparison of the row drug class to the column drug class. The rows represent the comparison of the row drug class to the column drug class. The effect estimates are expressed as mean difference and 95% confidence interval. For example, the odds ratio for adverse effects of Biyuan Tongqiao Granules + CWMT compared to Danxi Yuping Feng Granules+CWMT is 1.40 (95% confidence interval 0.47 to 4.13). Odds ratio <1 favors the drug in the row, and odds ratio >1 favors the drug in the column.

| Biyuan Tongqiao Granules+CWMT |  |  |  |  |  |  |
| --- | --- | --- | --- | --- | --- | --- |
| 1.40 (0.47,4.13) | Danxi Yuping Feng Granules+CWMT |  |  |  |  |  |
| 2.10 (0.49,9.01) | 1.50 (0.29,7.86) | Huaiqi Huang Granules+CWMT |  |  |  |  |
| 0.94 (0.40,2.20) | 0.67 (0.21,2.14) | 0.45 (0.10,2.03) | Tongqiao Biyan Granules+CWMT |  |  |  |
| 0.42 (0.12,1.48) | 0.30 (0.07,1.33) | 0.20 (0.03,1.18) | 0.45 (0.12,1.68) | Xinqin Granules+CWMT |  |  |
| 1.19 (0.64,2.19) | 0.85 (0.31,2.32) | 0.56 (0.14,2.28) | 1.26 (0.60,2.66) | 2.82 (0.86,9.26) | Yuping Feng Granules+CWMT |  |
| 0.50 (0.29,0.84) | 0.36 (0.14,0.92) | 0.24 (0.06,0.92) | 0.53 (0.27,1.03) | 1.18 (0.38,3.70) | 0.42 (0.30,0.58) | CWMT |

Abbreviations: CWMT , Conventional Western Medicine Treatment.

**Appendix 10: CINeMA Assessment**

We use the CINeMA framework to evidence certainty, assessing it for each network estimate based on the following criteria:

**A: Within study bias:** We classified the overall risk of bias for each study as low risk of bias, the risk of bias as moderate when none of the four assessed risk of bias items were rated as high risk, and the risk of bias as high when one or both items were rated as high risk. See Appendix 4 for the bias assessment. The risk of bias for a pairwise comparison of each drug is shown in figure S10.1-10.14.

**Figure S10.1:** Risk of bias contribution by intervention group in Nasal obstruction


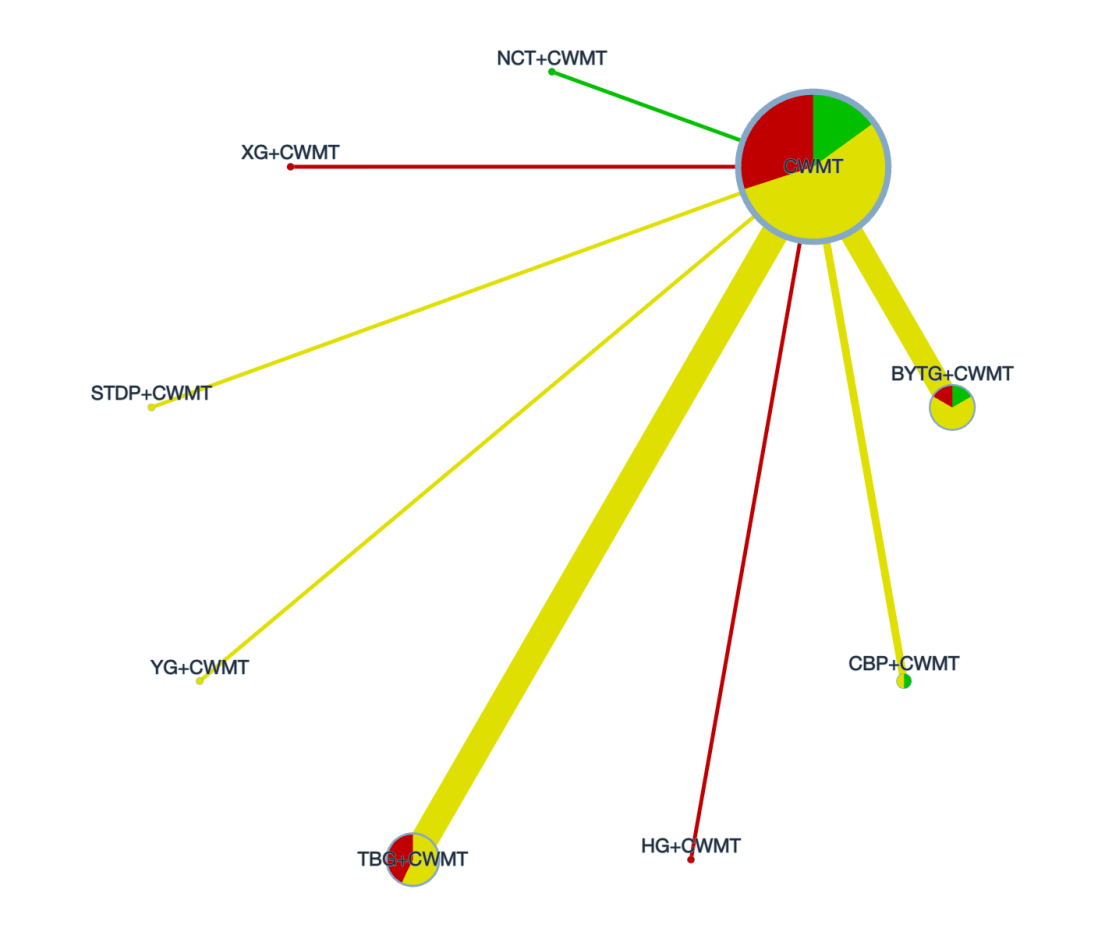


**Figure S10.2:** Overall risk of bias by treatment comparison in Nasal obstruction


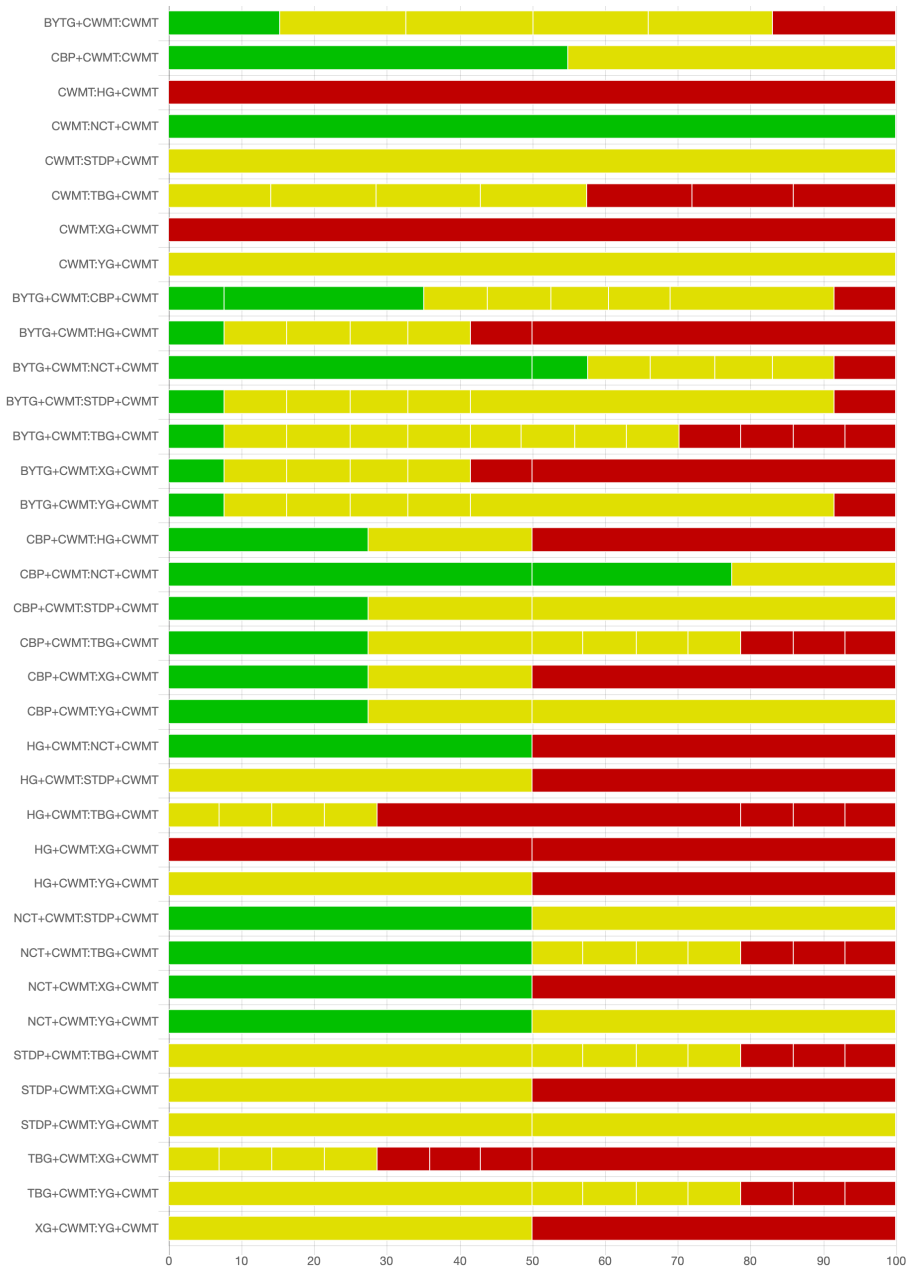


Abbreviations: CWMT , Conventional Western Medicine Treatment ;

NCT ,Nasal Comfort Tablets ;

BYTG , Biyuan Tongqiao Granules ;

CBP , Cang'er Zibi Yan Pills ;

HG , Huaiqi Huang Granules ;

STDP , Sanfeng Tongqiao Dropping Pills ;

TBG , Tongqiao Biyan Granules ;

XG , Xinqin Granules ;

YG , Yuping Feng Granules .

**Figure S10.3:** Risk of bias contribution by intervention group in Nasal pruritus


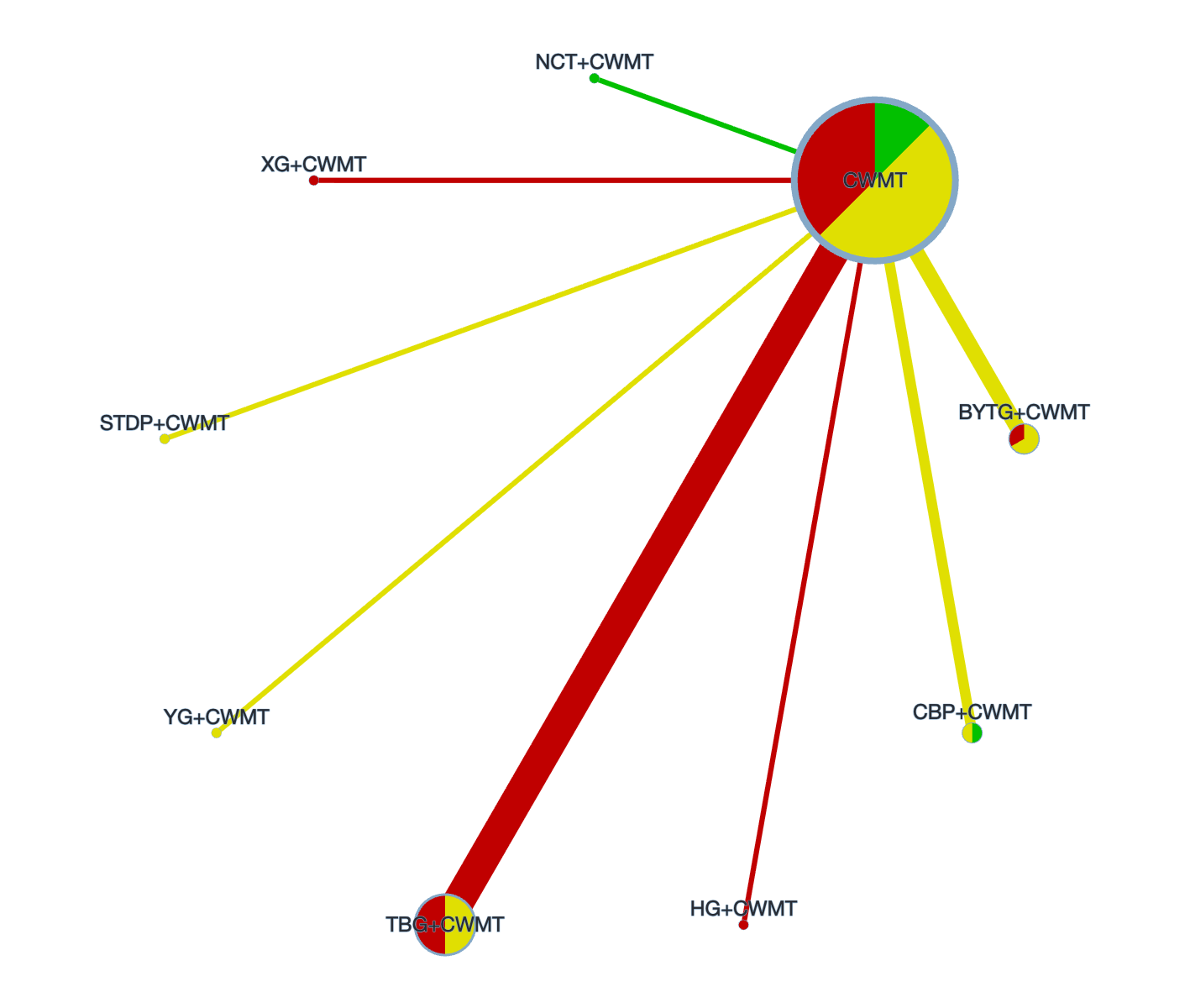


**Figure S10.4:** Overall risk of bias by treatment comparison in Nasal pruritus


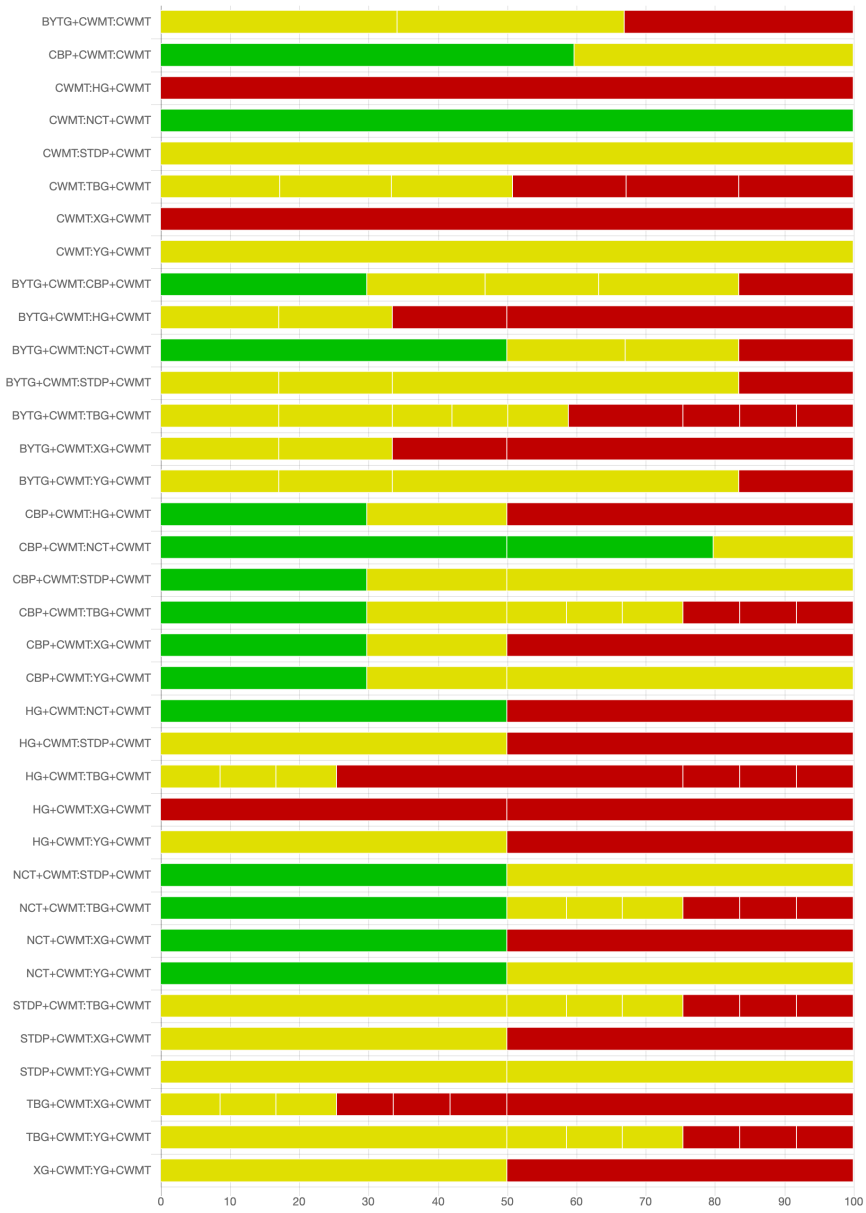


Abbreviations: CWMT , Conventional Western Medicine Treatment ;

NCT ,Nasal Comfort Tablets ;

BYTG , Biyuan Tongqiao Granules ;

CBP , Cang'er Zibi Yan Pills ;

HG , Huaiqi Huang Granules ;

STDP , Sanfeng Tongqiao Dropping Pills ;

TBG , Tongqiao Biyan Granules ;

XG , Xinqin Granules ;

YG , Yuping Feng Granules .

**Figure S10.5:** Risk of bias contribution by intervention group in Paroxysmal sneezing


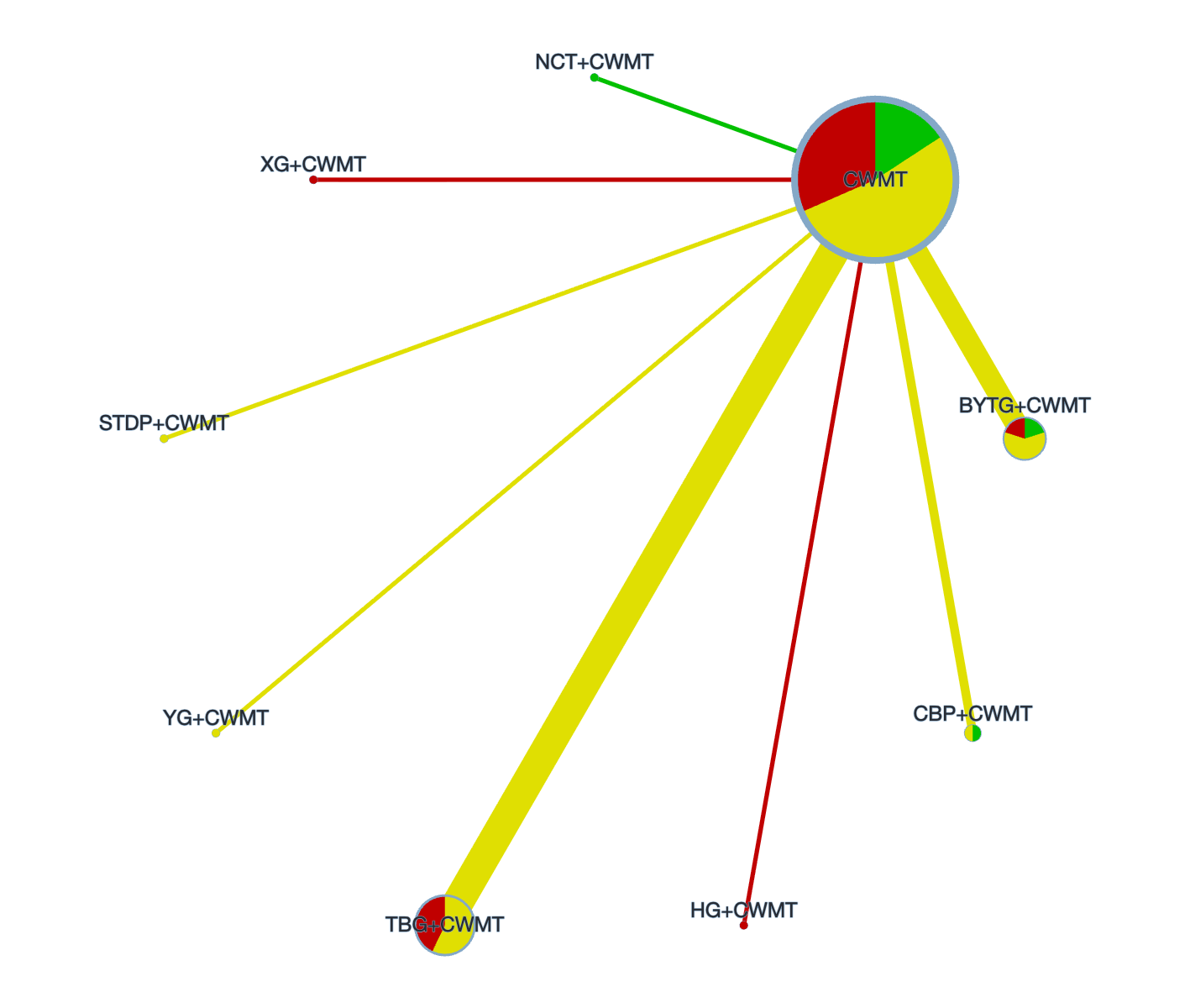


**Figure S10.6:** Overall risk of bias by treatment comparison in Paroxysmal sneezing


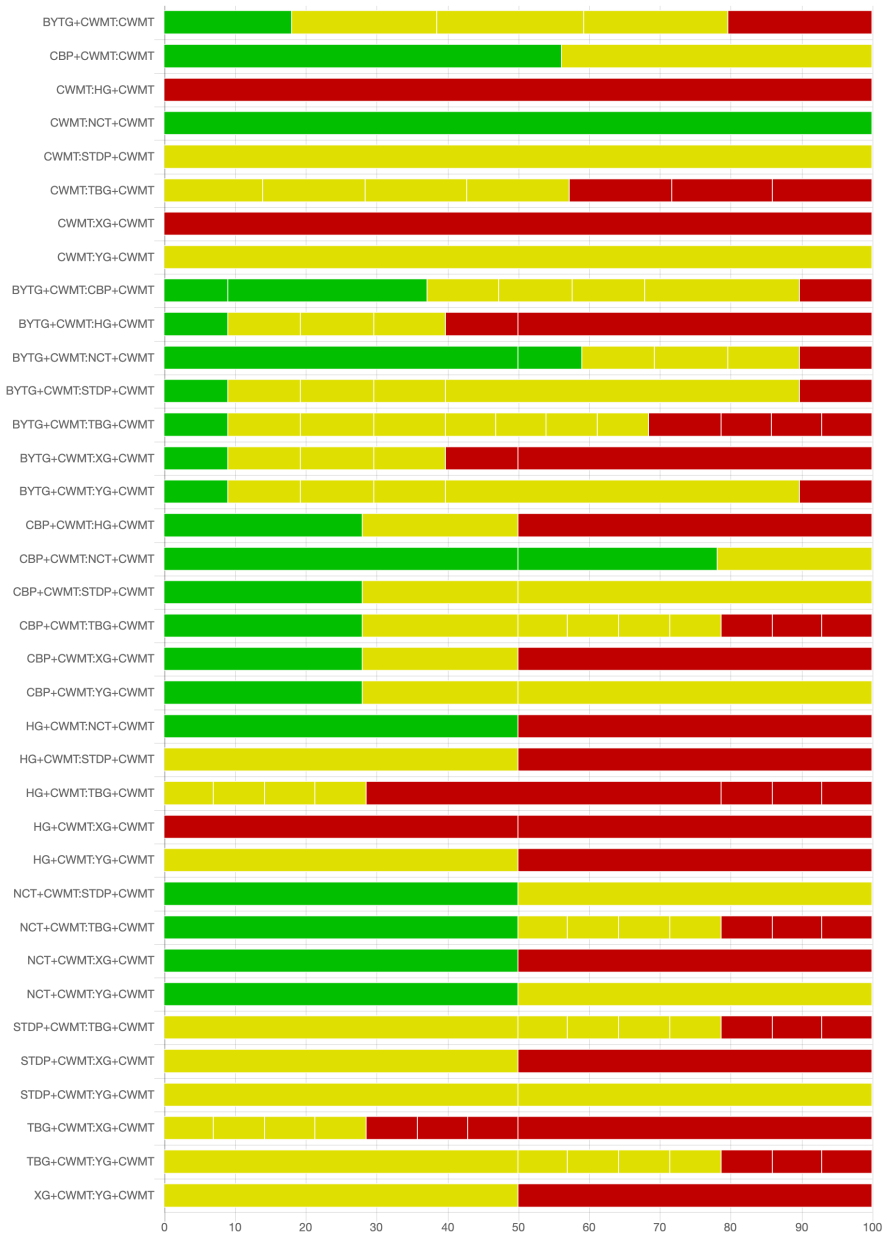


Abbreviations: CWMT , Conventional Western Medicine Treatment ;

NCT ,Nasal Comfort Tablets ;

BYTG , Biyuan Tongqiao Granules ;

CBP , Cang'er Zibi Yan Pills ;

HG , Huaiqi Huang Granules ;

STDP , Sanfeng Tongqiao Dropping Pills ;

TBG , Tongqiao Biyan Granules ;

XG , Xinqin Granules ;

YG , Yuping Feng Granules .

**Figure S10.7:** Risk of bias contribution by intervention group in Nasal discharge


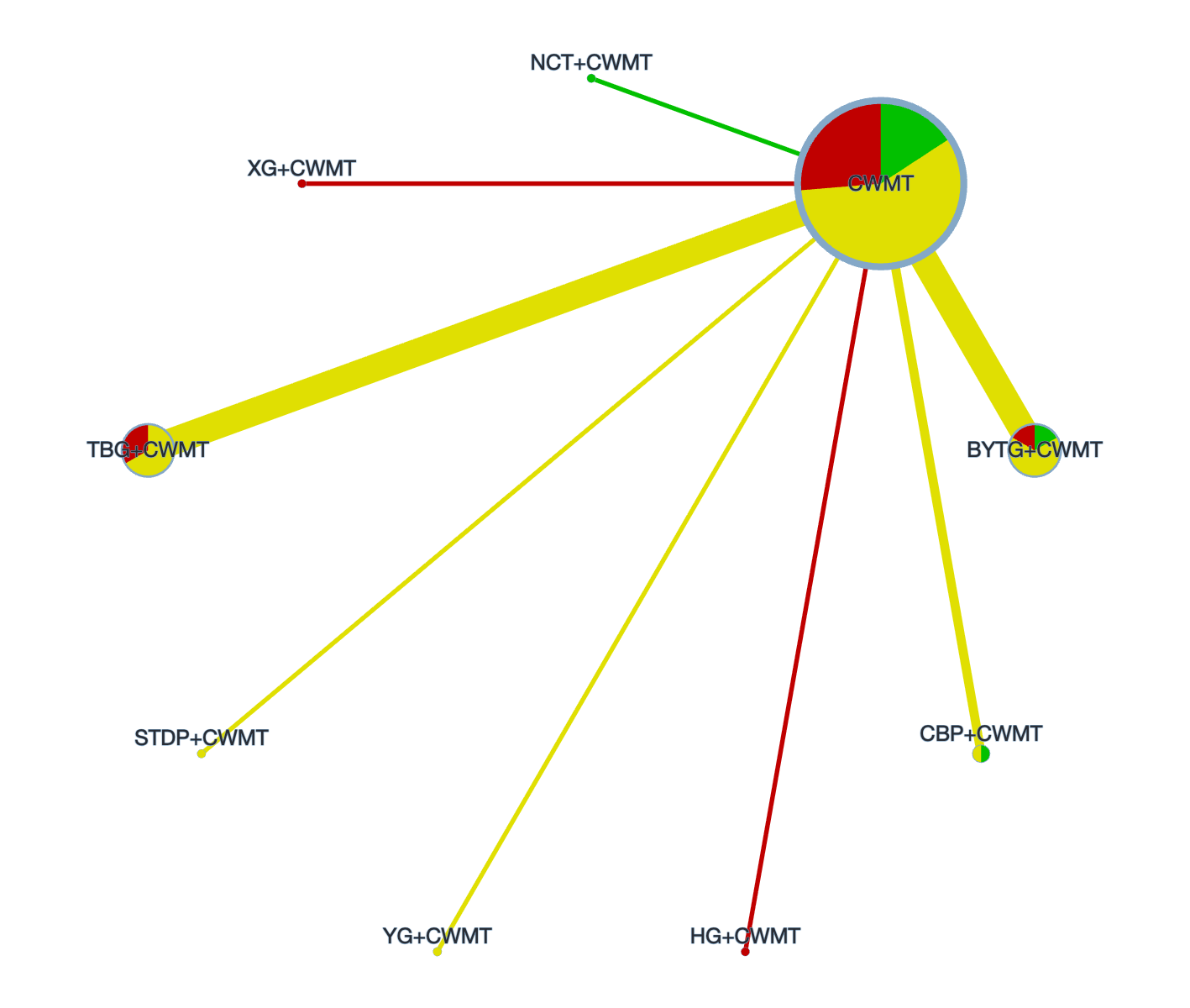


**Figure S10.8:** Overall risk of bias by treatment comparison in Nasal discharge


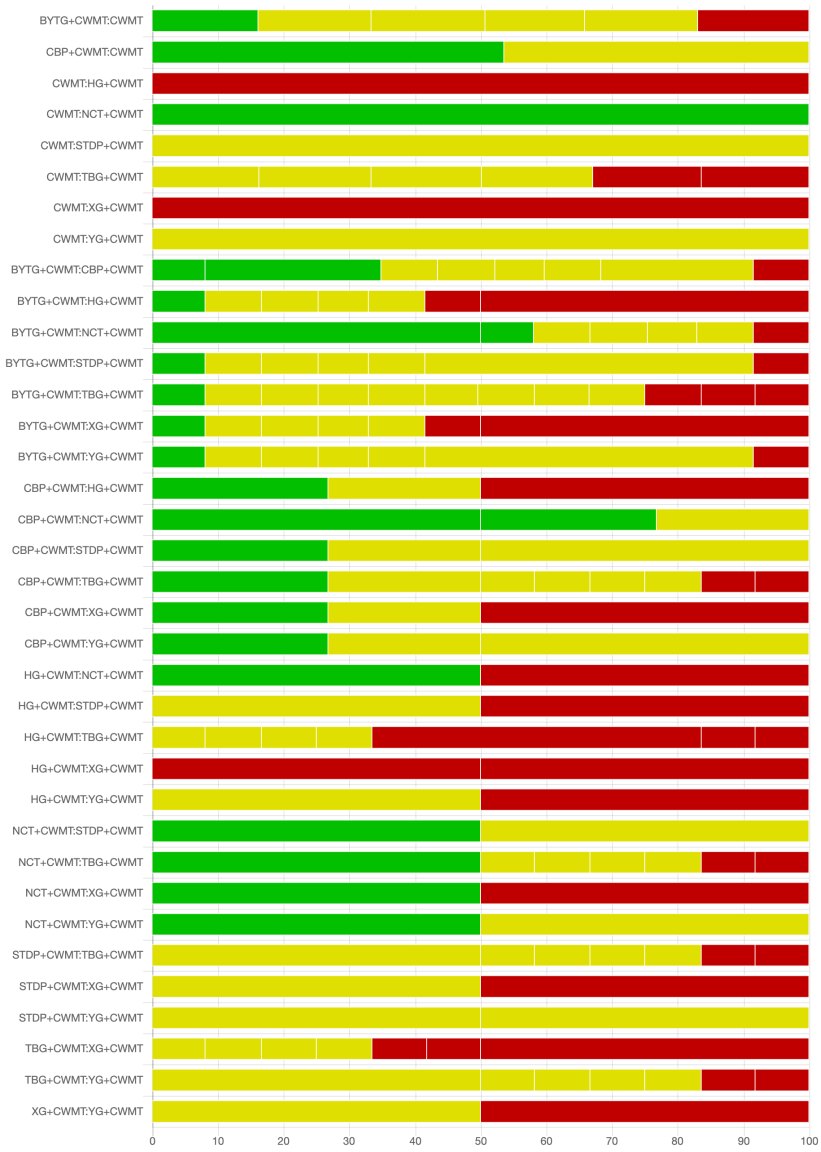


Abbreviations: CWMT , Conventional Western Medicine Treatment ;

NCT ,Nasal Comfort Tablets ;

BYTG , Biyuan Tongqiao Granules ;

CBP , Cang'er Zibi Yan Pills ;

HG , Huaiqi Huang Granules ;

STDP , Sanfeng Tongqiao Dropping Pills ;

TBG , Tongqiao Biyan Granules ;

XG , Xinqin Granules ;

YG , Yuping Feng Granules .

**Figure S10.9:** Risk of bias contribution by intervention group in IgE


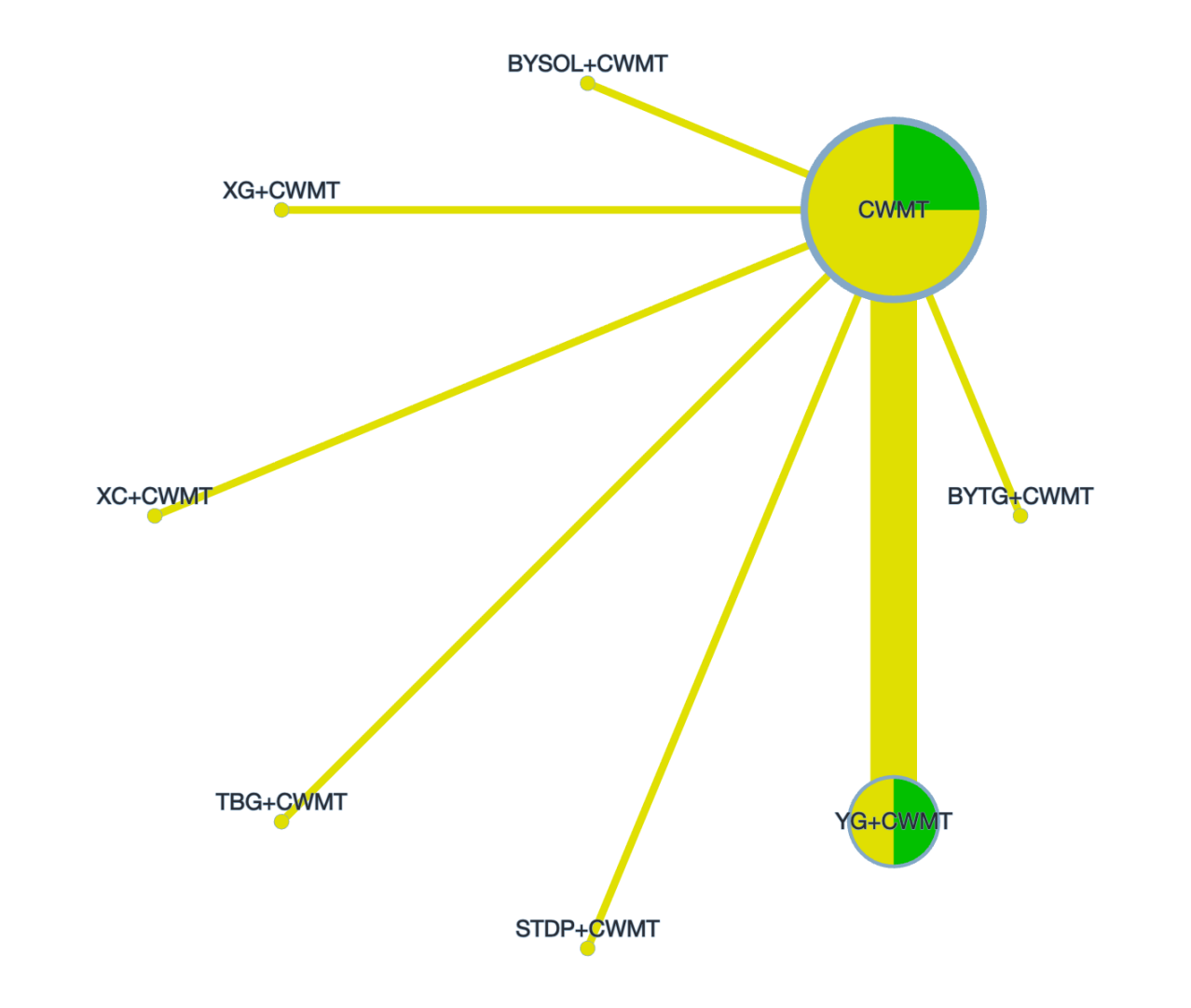


**Figure S10.10:** Overall risk of bias by treatment comparison in IgE


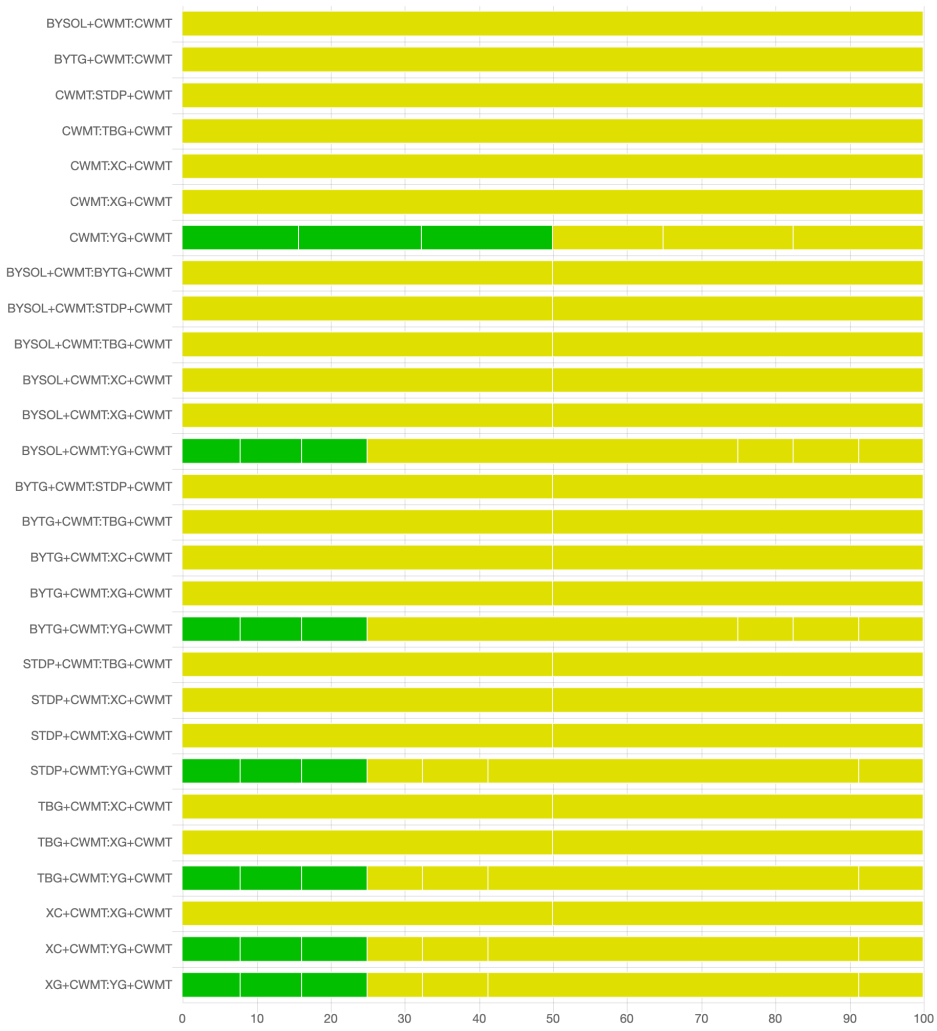


Abbreviations: CWMT , Conventional Western Medicine Treatment ;

BYSOL , Biyuan Shu Oral Liquid;

BYTG , Biyuan Tongqiao Granules ;

STDP , Sanfeng Tongqiao Dropping Pills ;

TBG , Tongqiao Biyan Granules ;

XC , Xiangju Capsules ;

XG , Xinqin Granules ;

YG , Yuping Feng Granules .

**Figure S10.11:** Risk of bias contribution by intervention group in Effective rate


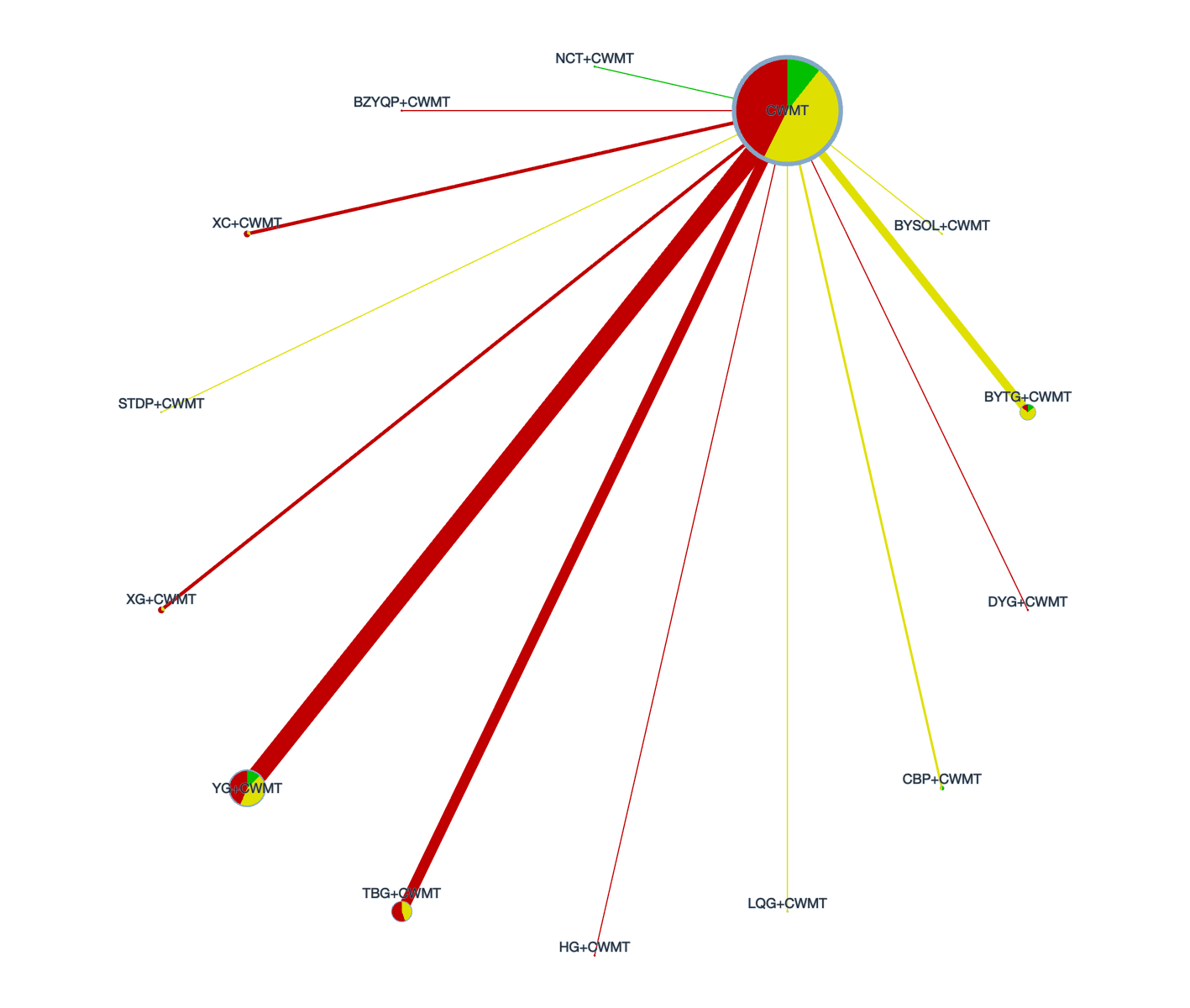


**Figure S10.12:** Overall risk of bias by treatment comparison in Effective rate


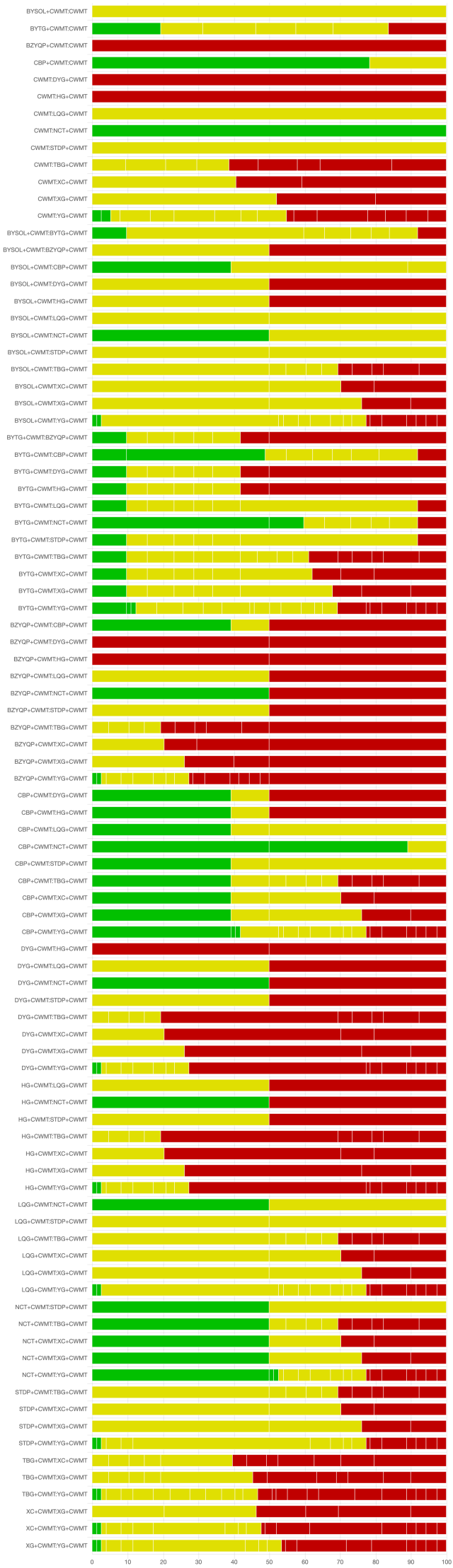


Abbreviations: CWMT , Conventional Western Medicine Treatment ;

NCT ,Nasal Comfort Tablets ;

BYSOL , Biyuan Shu Oral Liquid;

BYTG , Biyuan Tongqiao Granules ;

BZYQP , Buzhong Yiqi Pills ;

CBP , Cang'er Zibi Yan Pills ;

DYG , Danxi Yuping Feng Granules ;

HG , Huaiqi Huang Granules ;

LQG , Lianhua Qingwen Granules ;

STDP , Sanfeng Tongqiao Dropping Pills ;

TBG , Tongqiao Biyan Granules ;

XC , Xiangju Capsules ;

XG , Xinqin Granules ;

YG , Yuping Feng Granules .

**Figure S10.13:** Risk of bias contribution by intervention group in Adverse events


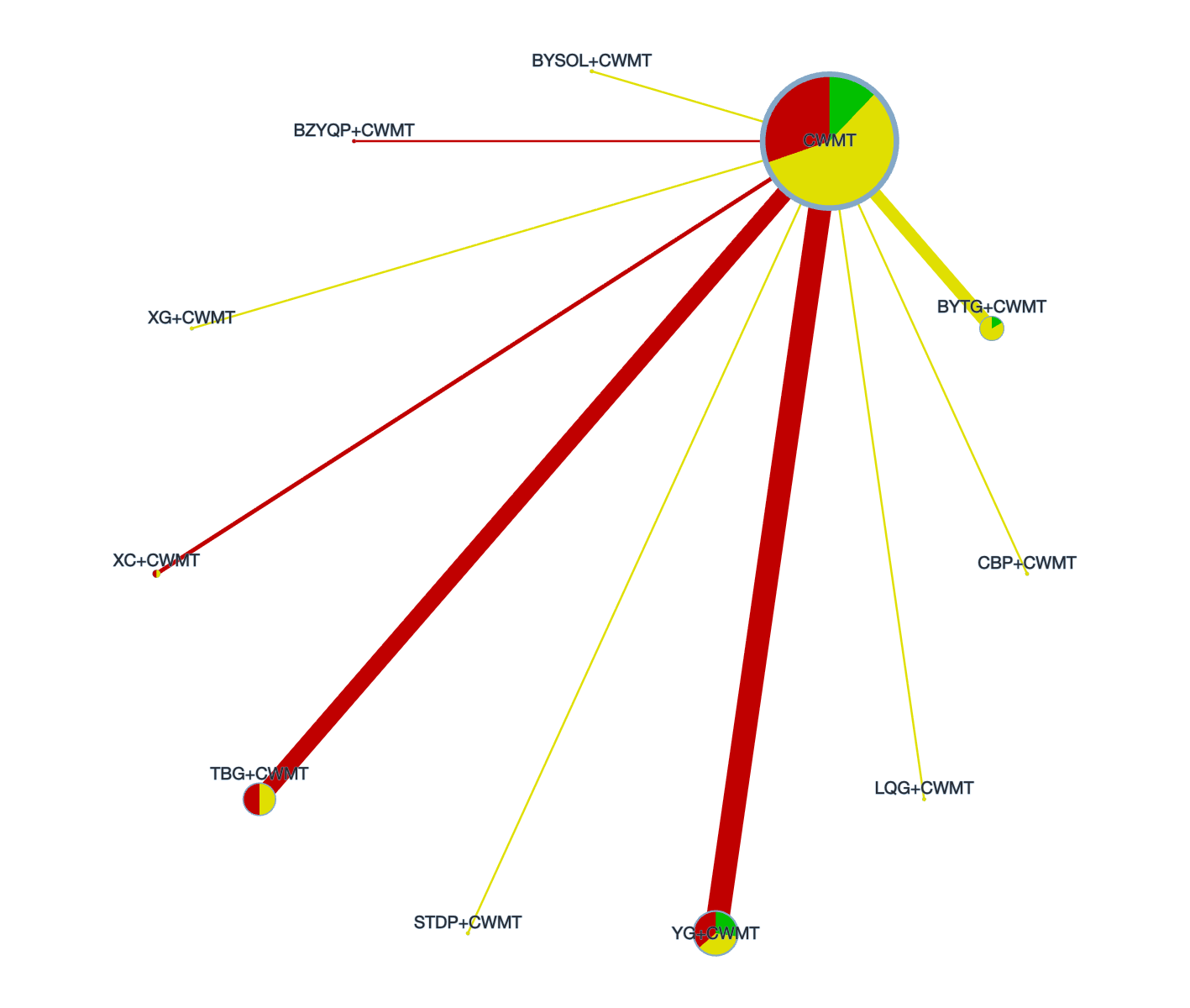


**Figure S10.14:** Overall risk of bias by treatment comparison in Adverse events


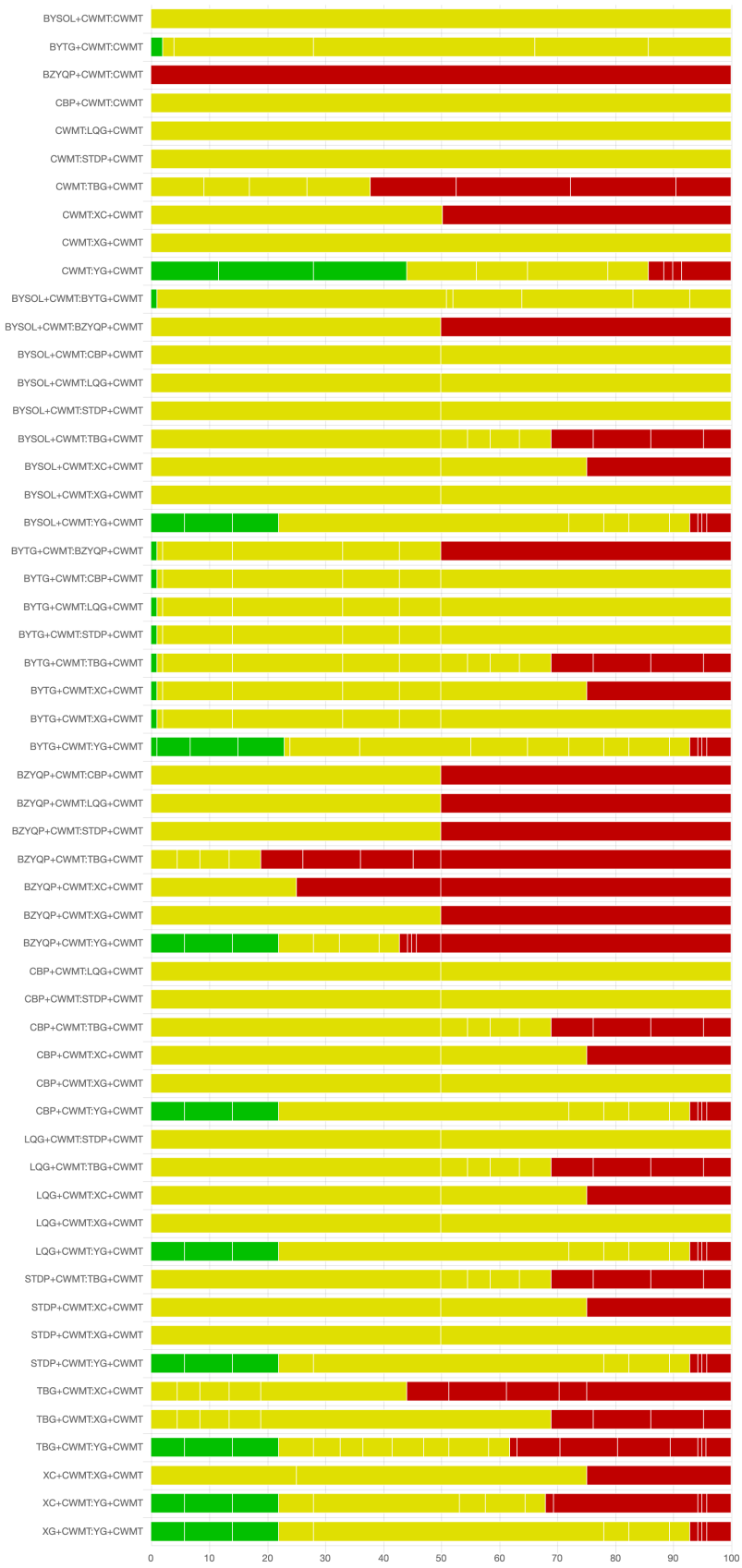


Abbreviations: CWMT , Conventional Western Medicine Treatment ;

BYSOL , Biyuan Shu Oral Liquid;

BYTG , Biyuan Tongqiao Granules ;

BZYQP , Buzhong Yiqi Pills ;

CBP , Cang'er Zibi Yan Pills ;

LQG , Lianhua Qingwen Granules ;

STDP , Sanfeng Tongqiao Dropping Pills ;

TBG , Tongqiao Biyan Granules ;

XC , Xiangju Capsules ;

XG , Xinqin Granules ;

YG , Yuping Feng Granules

**Figure S10.15:** Risk of bias contribution by intervention group in Recurrence Rate


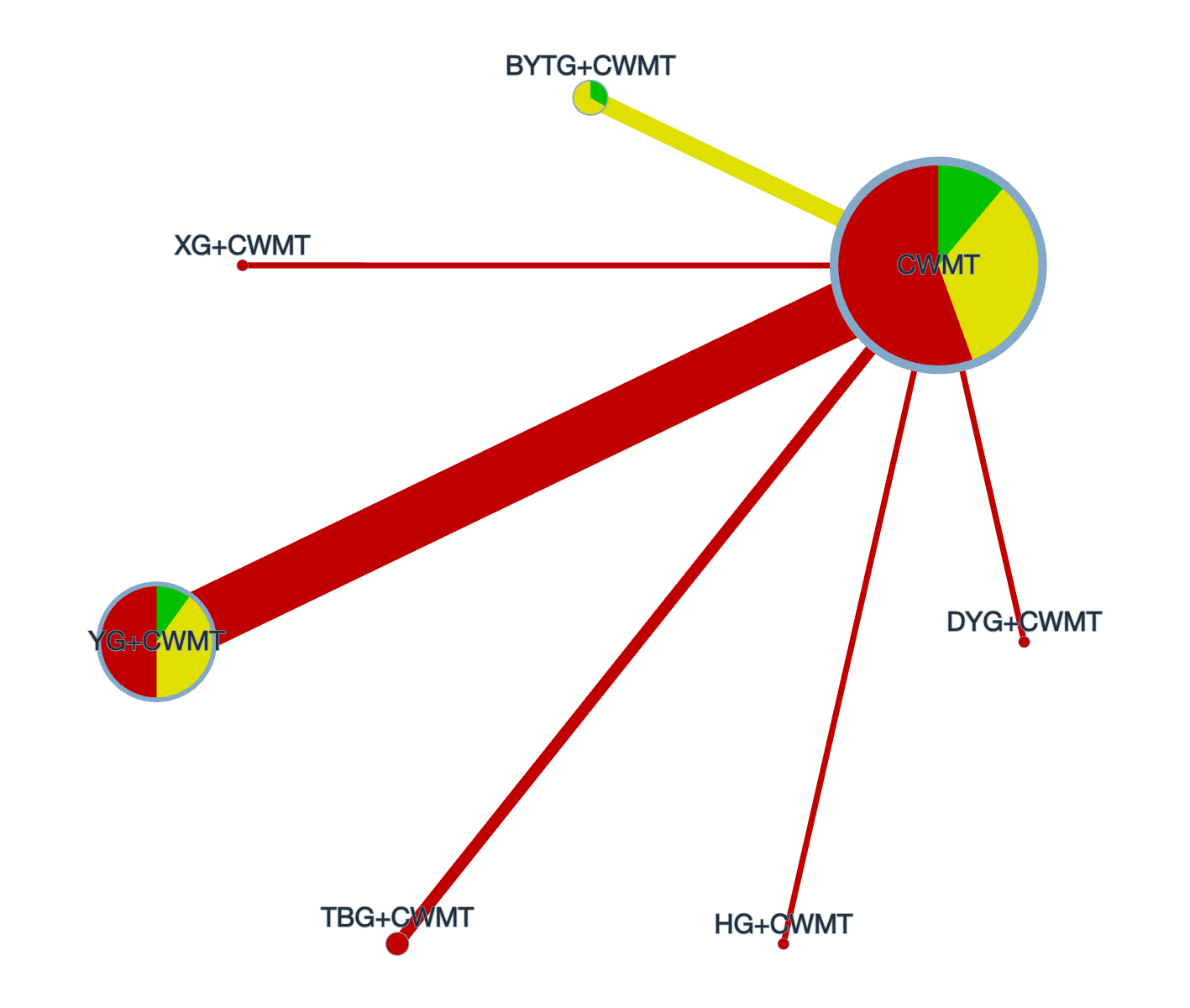


**Figure S10.16:** Overall risk of bias by treatment comparison in Recurrence Rate


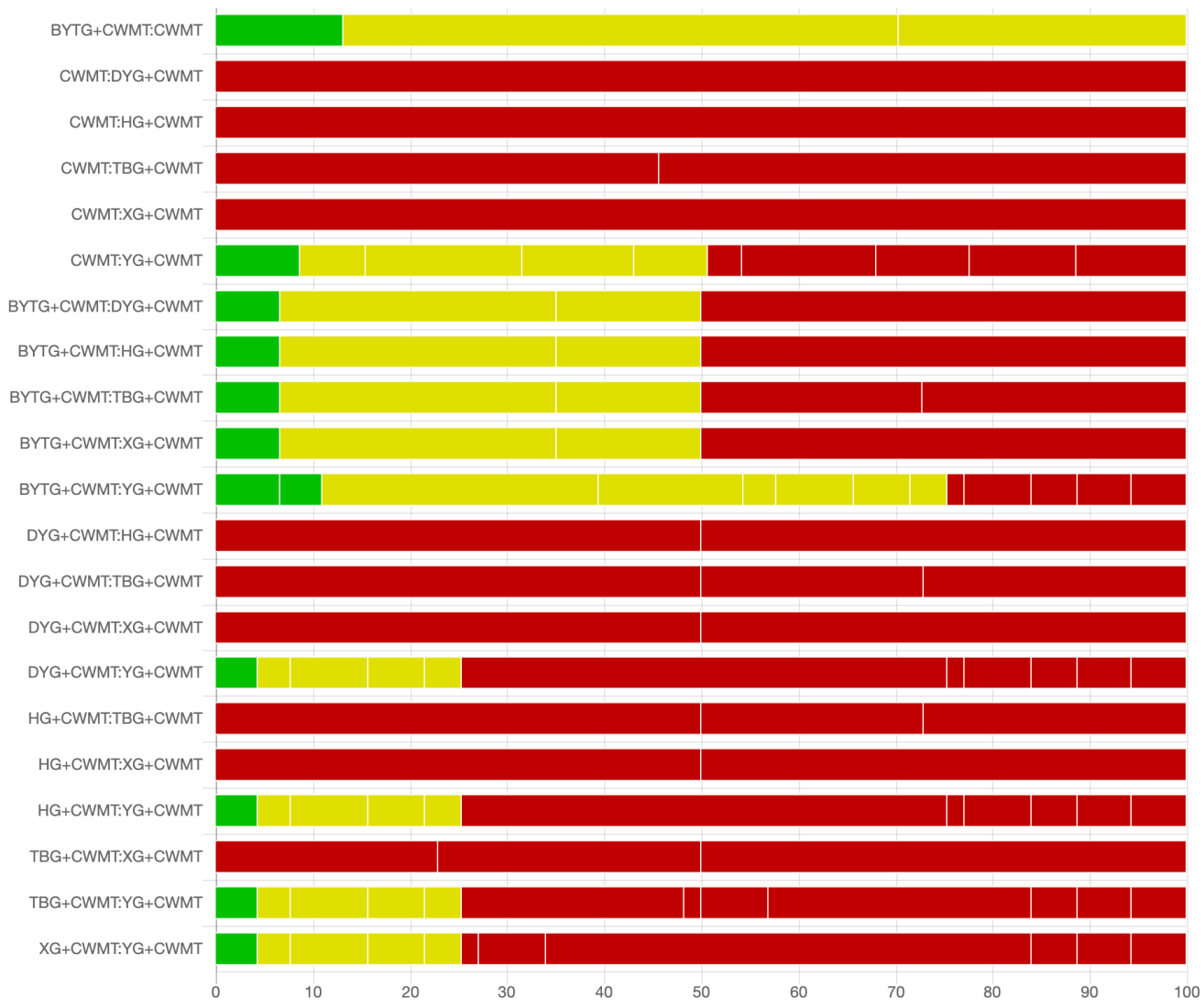


Abbreviations: CWMT , Conventional Western Medicine Treatment ;

BYTG , Biyuan Tongqiao Granules ;

DYG , Danxi Yuping Feng Granules ;

HG , Huaiqi Huang Granules ;

TBG , Tongqiao Biyan Granules ;

XG , Xinqin Granules ;

YG , Yuping Feng Granules .

- **Reporting bias:** We judged it visually by a funnel plot **(Appendix 11)**.
- **Indirectness:** Indirectness was evaluated by comparing key clinical and methodological characteristics across interventions to ensure the validity of transitivity assumptions. For each Chinese patent medicine (CPM), we summarized in Table S10.1 the number of randomized controlled trials (RCTs), total participants, mean age, average disease duration, and overall risk of bias among the included studies.

**Table S10.1:** Transitivity (Indirectness) Assessment

| CPM | Number of RCTs | Total participants | Mean age (years) | Mean disease duration (months) | Overall risk of bias (RoB 2.0) |
| --- | --- | --- | --- | --- | --- |
| Nasal Comfort Tablets | 1 | 120 | 8.20±1.39 | 38.0±9.6 | Low 1 |
| Biyuan Shu Oral Liquid | 1 | 54 | 7.68±1.22 | 0.138±0.004 | Some concerns 1 |
| Biyuan Tongqiao Granules | 8 | 492 | 7.87 ±2.18 | 9.85±2.21 | Low 1  Some concerns 6  High 1 |
| Buzhong Yiqi Pills | 1 | 52 | 7-13 | 6-30 | High 1 |
| Cang'er Zibi Yan Pills | 2 | 100 | 7.43±2.15 | 24.58±5.17 | Low 1  Some concerns 1 |
| Danxi Yuping Feng Granules | 1 | 49 | - | - | High 1 |
| Huaiqi Huang Granules | 1 | 24 | 6.8±1.9 | - | High 1 |
| Lianhua Qingwen Granules | 1 | 51 | 6.94 ± 1.52 | 33. 25 ± 5. 62 | Some concerns 1 |
| Sanfeng Tongqiao Dropping Pills | 1 | 50 | 9.45±1.16 | 1.13±0.36 | Some concerns 1 |
| Tongqiao Biyan Granules | 9 | 361 | 7.43±1.96 | 9.46±3.11 | Some concerns 4  High 5 |
| Xiangju Capsules | 3 | 140 | 9.71±1.70 | 15.96±2.64 | Some concerns 1  High 2 |
| Xinqin Granules | 3 | 199 | 6.48±1.73 | 17.30±7.45 | Some concerns 1  High 2 |
| Yuping Feng Granules | 17 | 857 | 7.18±1.36 | 29.32±7.74 | Low 3  Some concerns 7  High 7 |

- **Imprecision:** We use the CINeMA website to grade the accuracy of each comparison.
- **Heterogeneity:** We assessed the degree of worry by comparing clinical reasoning based on 95% confidence intervals (CIs) while applying the same clinical reasoning framework as for inaccuracy. In particular, we judged the consistency of our findings based on the confidence and prediction intervals associated with clinically important effect sizes. And we used the same thresholds of clinical significance as described above and followed the recommendations automatically provided by CINeMA (https://cinema.ispm.unibe.ch/).
- Inconsistency: For inconsistency, we looked at the results for node splitting (Appendix 6) and we saw major problems when p<0.10, but otherwise no problems.

Table S10.2: CINeMA Results of Nasal obstruction

| Comparison | Within-study bias | Reporting bias | Indirectness | Imprecision | Heterogeneity | Incoherence | Confidence rating |
| --- | --- | --- | --- | --- | --- | --- | --- |
| BYTG+CWMT:CWMT | Some concerns | Low risk | No concerns | No concerns | Major concerns | Major concerns | Low |
| CBP+CWMT:CWMT | No concerns | Low risk | No concerns | No concerns | Major concerns | Major concerns | Low |
| CWMT:HG+CWMT | Major concerns | Low risk | No concerns | Major concerns | No concerns | Major concerns | Very low |
| CWMT:NCT+CWMT | No concerns | Low risk | No concerns | Major concerns | No concerns | Major concerns | Very low |
| CWMT:STDP+CWMT | Some concerns | Low risk | No concerns | Major concerns | No concerns | Major concerns | Very low |
| CWMT:TBG+CWMT | Some concerns | Low risk | No concerns | No concerns | Major concerns | Major concerns | Low |
| CWMT:XG+CWMT | Major concerns | Low risk | No concerns | Major concerns | No concerns | Major concerns | Very low |
| CWMT:YG+CWMT | Some concerns | Low risk | No concerns | Major concerns | No concerns | Major concerns | Very low |
| BYTG+CWMT:CBP+CWMT | Some concerns | Low risk | No concerns | Major concerns | No concerns | Major concerns | Very low |
| BYTG+CWMT:HG+CWMT | Major concerns | Low risk | No concerns | Major concerns | No concerns | Major concerns | Very low |
| BYTG+CWMT:NCT+CWMT | No concerns | Low risk | No concerns | Major concerns | No concerns | Major concerns | Very low |
| BYTG+CWMT:STDP+CWMT | Some concerns | Low risk | No concerns | Major concerns | No concerns | Major concerns | Very low |
| BYTG+CWMT:TBG+CWMT | Some concerns | Low risk | No concerns | Major concerns | No concerns | Major concerns | Very low |
| BYTG+CWMT:XG+CWMT | Major concerns | Low risk | No concerns | Major concerns | No concerns | Major concerns | Very low |
| BYTG+CWMT:YG+CWMT | Some concerns | Low risk | No concerns | Major concerns | No concerns | Major concerns | Very low |
| CBP+CWMT:HG+CWMT | Major concerns | Low risk | No concerns | Major concerns | No concerns | Major concerns | Very low |
| CBP+CWMT:NCT+CWMT | No concerns | Low risk | No concerns | Major concerns | No concerns | Major concerns | Very low |
| CBP+CWMT:STDP+CWMT | Some concerns | Low risk | No concerns | Major concerns | No concerns | Major concerns | Very low |
| CBP+CWMT:TBG+CWMT | Some concerns | Low risk | No concerns | Major concerns | No concerns | Major concerns | Very low |
| CBP+CWMT:XG+CWMT | Major concerns | Low risk | No concerns | Major concerns | No concerns | Major concerns | Very low |
| CBP+CWMT:YG+CWMT | Some concerns | Low risk | No concerns | Major concerns | No concerns | Major concerns | Very low |
| HG+CWMT:NCT+CWMT | No concerns | Low risk | No concerns | Major concerns | No concerns | Major concerns | Very low |
| HG+CWMT:STDP+CWMT | Some concerns | Low risk | No concerns | Major concerns | No concerns | Major concerns | Very low |
| HG+CWMT:TBG+CWMT | Major concerns | Low risk | No concerns | Major concerns | No concerns | Major concerns | Very low |
| HG+CWMT:XG+CWMT | Major concerns | Low risk | No concerns | Major concerns | No concerns | Major concerns | Very low |
| HG+CWMT:YG+CWMT | Some concerns | Low risk | No concerns | Major concerns | No concerns | Major concerns | Very low |
| NCT+CWMT:STDP+CWMT | No concerns | Low risk | No concerns | Major concerns | No concerns | Major concerns | Very low |
| NCT+CWMT:TBG+CWMT | No concerns | Low risk | No concerns | Major concerns | No concerns | Major concerns | Very low |
| NCT+CWMT:XG+CWMT | No concerns | Low risk | No concerns | Major concerns | No concerns | Major concerns | Very low |
| NCT+CWMT:YG+CWMT | No concerns | Low risk | No concerns | Major concerns | No concerns | Major concerns | Very low |
| STDP+CWMT:TBG+CWMT | Some concerns | Low risk | No concerns | Major concerns | No concerns | Major concerns | Very low |
| STDP+CWMT:XG+CWMT | Some concerns | Low risk | No concerns | Major concerns | No concerns | Major concerns | Very low |
| STDP+CWMT:YG+CWMT | Some concerns | Low risk | No concerns | Major concerns | No concerns | Major concerns | Very low |
| TBG+CWMT:XG+CWMT | Major concerns | Low risk | No concerns | Major concerns | No concerns | Major concerns | Very low |
| TBG+CWMT:YG+CWMT | Some concerns | Low risk | No concerns | Major concerns | No concerns | Major concerns | Very low |
| XG+CWMT:YG+CWMT | Some concerns | Low risk | No concerns | Major concerns | No concerns | Major concerns | Very low |

Abbreviations: CWMT , Conventional Western Medicine Treatment ;

NCT ,Nasal Comfort Tablets ;

BYTG , Biyuan Tongqiao Granules ;

CBP , Cang'er Zibi Yan Pills ;

HG , Huaiqi Huang Granules ;

STDP , Sanfeng Tongqiao Dropping Pills ;

TBG , Tongqiao Biyan Granules ;

XG , Xinqin Granules ;

YG , Yuping Feng Granules .

Table S10.3: CINeMA Results of Nasal pruritus

| Comparison | Within-study bias | Reporting bias | Indirectness | Imprecision | Heterogeneity | Incoherence | Confidence rating |
| --- | --- | --- | --- | --- | --- | --- | --- |
| BYTG+CWMT:CWMT | Some concerns | Low risk | No concerns | Major concerns | No concerns | Major concerns | Very low |
| CBP+CWMT:CWMT | No concerns | Low risk | No concerns | No concerns | Major concerns | Major concerns | Low |
| CWMT:HG+CWMT | Major concerns | Low risk | No concerns | Major concerns | No concerns | Major concerns | Very low |
| CWMT:NCT+CWMT | No concerns | Low risk | No concerns | Major concerns | No concerns | Major concerns | Very low |
| CWMT:STDP+CWMT | Some concerns | Low risk | No concerns | No concerns | Major concerns | Major concerns | Low |
| CWMT:TBG+CWMT | Some concerns | Low risk | No concerns | No concerns | Major concerns | Major concerns | Low |
| CWMT:XG+CWMT | Major concerns | Low risk | No concerns | Major concerns | No concerns | Major concerns | Very low |
| CWMT:YG+CWMT | Some concerns | Low risk | No concerns | Major concerns | No concerns | Major concerns | Very low |
| BYTG+CWMT:CBP+CWMT | Some concerns | Low risk | No concerns | Major concerns | No concerns | Major concerns | Very low |
| BYTG+CWMT:HG+CWMT | Major concerns | Low risk | No concerns | Major concerns | No concerns | Major concerns | Very low |
| BYTG+CWMT:NCT+CWMT | No concerns | Low risk | No concerns | Major concerns | No concerns | Major concerns | Very low |
| BYTG+CWMT:STDP+CWMT | Some concerns | Low risk | No concerns | Major concerns | No concerns | Major concerns | Very low |
| BYTG+CWMT:TBG+CWMT | Some concerns | Low risk | No concerns | Major concerns | No concerns | Major concerns | Very low |
| BYTG+CWMT:XG+CWMT | Major concerns | Low risk | No concerns | Major concerns | No concerns | Major concerns | Very low |
| BYTG+CWMT:YG+CWMT | Some concerns | Low risk | No concerns | Major concerns | No concerns | Major concerns | Very low |
| CBP+CWMT:HG+CWMT | Major concerns | Low risk | No concerns | Major concerns | No concerns | Major concerns | Very low |
| CBP+CWMT:NCT+CWMT | No concerns | Low risk | No concerns | Major concerns | No concerns | Major concerns | Very low |
| CBP+CWMT:STDP+CWMT | Some concerns | Low risk | No concerns | Major concerns | No concerns | Major concerns | Very low |
| CBP+CWMT:TBG+CWMT | Some concerns | Low risk | No concerns | Major concerns | No concerns | Major concerns | Very low |
| CBP+CWMT:XG+CWMT | Major concerns | Low risk | No concerns | Major concerns | No concerns | Major concerns | Very low |
| CBP+CWMT:YG+CWMT | Some concerns | Low risk | No concerns | Major concerns | No concerns | Major concerns | Very low |
| HG+CWMT:NCT+CWMT | No concerns | Low risk | No concerns | Major concerns | No concerns | Major concerns | Very low |
| HG+CWMT:STDP+CWMT | Some concerns | Low risk | No concerns | Major concerns | No concerns | Major concerns | Very low |
| HG+CWMT:TBG+CWMT | Major concerns | Low risk | No concerns | Major concerns | No concerns | Major concerns | Very low |
| HG+CWMT:XG+CWMT | Major concerns | Low risk | No concerns | Major concerns | No concerns | Major concerns | Very low |
| HG+CWMT:YG+CWMT | Some concerns | Low risk | No concerns | Major concerns | No concerns | Major concerns | Very low |
| NCT+CWMT:STDP+CWMT | No concerns | Low risk | No concerns | Major concerns | No concerns | Major concerns | Very low |
| NCT+CWMT:TBG+CWMT | No concerns | Low risk | No concerns | Major concerns | No concerns | Major concerns | Very low |
| NCT+CWMT:XG+CWMT | No concerns | Low risk | No concerns | Major concerns | No concerns | Major concerns | Very low |
| NCT+CWMT:YG+CWMT | No concerns | Low risk | No concerns | Major concerns | No concerns | Major concerns | Very low |
| STDP+CWMT:TBG+CWMT | Some concerns | Low risk | No concerns | Major concerns | No concerns | Major concerns | Very low |
| STDP+CWMT:XG+CWMT | Some concerns | Low risk | No concerns | Major concerns | No concerns | Major concerns | Very low |
| STDP+CWMT:YG+CWMT | Some concerns | Low risk | No concerns | Major concerns | No concerns | Major concerns | Very low |
| TBG+CWMT:XG+CWMT | Major concerns | Low risk | No concerns | Major concerns | No concerns | Major concerns | Very low |
| TBG+CWMT:YG+CWMT | Some concerns | Low risk | No concerns | Major concerns | No concerns | Major concerns | Very low |
| XG+CWMT:YG+CWMT | Some concerns | Low risk | No concerns | Major concerns | No concerns | Major concerns | Very low |

Abbreviations: CWMT , Conventional Western Medicine Treatment ;

NCT ,Nasal Comfort Tablets ;

BYTG , Biyuan Tongqiao Granules ;

CBP , Cang'er Zibi Yan Pills ;

HG , Huaiqi Huang Granules ;

STDP , Sanfeng Tongqiao Dropping Pills ;

TBG , Tongqiao Biyan Granules ;

XG , Xinqin Granules ;

YG , Yuping Feng Granules .

Table S10.4: CINeMA Results of Paroxysmal sneezing

| Comparison | Within-study bias | Reporting bias | Indirectness | Imprecision | Heterogeneity | Incoherence | Confidence rating |
| --- | --- | --- | --- | --- | --- | --- | --- |
| BYTG+CWMT:CWMT | Some concerns | Low risk | No concerns | No concerns | Major concerns | Major concerns | Low |
| CBP+CWMT:CWMT | No concerns | Low risk | No concerns | Major concerns | No concerns | Major concerns | Very low |
| CWMT:HG+CWMT | Major concerns | Low risk | No concerns | Major concerns | No concerns | Major concerns | Very low |
| CWMT:NCT+CWMT | No concerns | Low risk | No concerns | Major concerns | No concerns | Major concerns | Very low |
| CWMT:STDP+CWMT | Some concerns | Low risk | No concerns | Major concerns | No concerns | Major concerns | Very low |
| CWMT:TBG+CWMT | Some concerns | Low risk | No concerns | No concerns | Major concerns | Major concerns | Low |
| CWMT:XG+CWMT | Major concerns | Low risk | No concerns | Major concerns | No concerns | Major concerns | Very low |
| CWMT:YG+CWMT | Some concerns | Low risk | No concerns | Major concerns | No concerns | Major concerns | Very low |
| BYTG+CWMT:CBP+CWMT | Some concerns | Low risk | No concerns | Major concerns | No concerns | Major concerns | Very low |
| BYTG+CWMT:HG+CWMT | Major concerns | Low risk | No concerns | Major concerns | No concerns | Major concerns | Very low |
| BYTG+CWMT:NCT+CWMT | No concerns | Low risk | No concerns | Major concerns | No concerns | Major concerns | Very low |
| BYTG+CWMT:STDP+CWMT | Some concerns | Low risk | No concerns | Major concerns | No concerns | Major concerns | Very low |
| BYTG+CWMT:TBG+CWMT | Some concerns | Low risk | No concerns | Major concerns | No concerns | Major concerns | Very low |
| BYTG+CWMT:XG+CWMT | Major concerns | Low risk | No concerns | Major concerns | No concerns | Major concerns | Very low |
| BYTG+CWMT:YG+CWMT | Some concerns | Low risk | No concerns | Major concerns | No concerns | Major concerns | Very low |
| CBP+CWMT:HG+CWMT | Major concerns | Low risk | No concerns | Major concerns | No concerns | Major concerns | Very low |
| CBP+CWMT:NCT+CWMT | No concerns | Low risk | No concerns | Major concerns | No concerns | Major concerns | Very low |
| CBP+CWMT:STDP+CWMT | Some concerns | Low risk | No concerns | Major concerns | No concerns | Major concerns | Very low |
| CBP+CWMT:TBG+CWMT | Some concerns | Low risk | No concerns | Major concerns | No concerns | Major concerns | Very low |
| CBP+CWMT:XG+CWMT | Major concerns | Low risk | No concerns | Major concerns | No concerns | Major concerns | Very low |
| CBP+CWMT:YG+CWMT | Some concerns | Low risk | No concerns | Major concerns | No concerns | Major concerns | Very low |
| HG+CWMT:NCT+CWMT | No concerns | Low risk | No concerns | Major concerns | No concerns | Major concerns | Very low |
| HG+CWMT:STDP+CWMT | Some concerns | Low risk | No concerns | Major concerns | No concerns | Major concerns | Very low |
| HG+CWMT:TBG+CWMT | Major concerns | Low risk | No concerns | Major concerns | No concerns | Major concerns | Very low |
| HG+CWMT:XG+CWMT | Major concerns | Low risk | No concerns | Major concerns | No concerns | Major concerns | Very low |
| HG+CWMT:YG+CWMT | Some concerns | Low risk | No concerns | Major concerns | No concerns | Major concerns | Very low |
| NCT+CWMT:STDP+CWMT | No concerns | Low risk | No concerns | Major concerns | No concerns | Major concerns | Very low |
| NCT+CWMT:TBG+CWMT | No concerns | Low risk | No concerns | Major concerns | No concerns | Major concerns | Very low |
| NCT+CWMT:XG+CWMT | No concerns | Low risk | No concerns | Major concerns | No concerns | Major concerns | Very low |
| NCT+CWMT:YG+CWMT | No concerns | Low risk | No concerns | Major concerns | No concerns | Major concerns | Very low |
| STDP+CWMT:TBG+CWMT | Some concerns | Low risk | No concerns | Major concerns | No concerns | Major concerns | Very low |
| STDP+CWMT:XG+CWMT | Some concerns | Low risk | No concerns | Major concerns | No concerns | Major concerns | Very low |
| STDP+CWMT:YG+CWMT | Some concerns | Low risk | No concerns | Major concerns | No concerns | Major concerns | Very low |
| TBG+CWMT:XG+CWMT | Major concerns | Low risk | No concerns | Major concerns | No concerns | Major concerns | Very low |
| TBG+CWMT:YG+CWMT | Some concerns | Low risk | No concerns | Major concerns | No concerns | Major concerns | Very low |
| XG+CWMT:YG+CWMT | Some concerns | Low risk | No concerns | Major concerns | No concerns | Major concerns | Very low |

Abbreviations: CWMT , Conventional Western Medicine Treatment ;

NCT ,Nasal Comfort Tablets ;

BYTG , Biyuan Tongqiao Granules ;

CBP , Cang'er Zibi Yan Pills ;

HG , Huaiqi Huang Granules ;

STDP , Sanfeng Tongqiao Dropping Pills ;

TBG , Tongqiao Biyan Granules ;

XG , Xinqin Granules ;

YG , Yuping Feng Granules .

Table S10.5: CINeMA Results of Nasal discharge

| Comparison | Within-study bias | Reporting bias | Indirectness | Imprecision | Heterogeneity | Incoherence | Confidence rating |
| --- | --- | --- | --- | --- | --- | --- | --- |
| BYTG+CWMT:CWMT | Some concerns | Low risk | No concerns | No concerns | Major concerns | Major concerns | Low |
| CBP+CWMT:CWMT | No concerns | Low risk | No concerns | No concerns | Major concerns | Major concerns | Low |
| CWMT:HG+CWMT | Major concerns | Low risk | No concerns | Major concerns | No concerns | Major concerns | Very low |
| CWMT:NCT+CWMT | No concerns | Low risk | No concerns | Major concerns | No concerns | Major concerns | Very low |
| CWMT:STDP+CWMT | Some concerns | Low risk | No concerns | Major concerns | No concerns | Major concerns | Very low |
| CWMT:TBG+CWMT | Some concerns | Low risk | No concerns | No concerns | Major concerns | Major concerns | Low |
| CWMT:XG+CWMT | Major concerns | Low risk | No concerns | Major concerns | No concerns | Major concerns | Very low |
| CWMT:YG+CWMT | Some concerns | Low risk | No concerns | Major concerns | No concerns | Major concerns | Very low |
| BYTG+CWMT:CBP+CWMT | Some concerns | Low risk | No concerns | Major concerns | No concerns | Major concerns | Very low |
| BYTG+CWMT:HG+CWMT | Major concerns | Low risk | No concerns | Major concerns | No concerns | Major concerns | Very low |
| BYTG+CWMT:NCT+CWMT | No concerns | Low risk | No concerns | Major concerns | No concerns | Major concerns | Very low |
| BYTG+CWMT:STDP+CWMT | Some concerns | Low risk | No concerns | Major concerns | No concerns | Major concerns | Very low |
| BYTG+CWMT:TBG+CWMT | Some concerns | Low risk | No concerns | Major concerns | No concerns | Major concerns | Very low |
| BYTG+CWMT:XG+CWMT | Major concerns | Low risk | No concerns | Major concerns | No concerns | Major concerns | Very low |
| BYTG+CWMT:YG+CWMT | Some concerns | Low risk | No concerns | Major concerns | No concerns | Major concerns | Very low |
| CBP+CWMT:HG+CWMT | Major concerns | Low risk | No concerns | Major concerns | No concerns | Major concerns | Very low |
| CBP+CWMT:NCT+CWMT | No concerns | Low risk | No concerns | Major concerns | No concerns | Major concerns | Very low |
| CBP+CWMT:STDP+CWMT | Some concerns | Low risk | No concerns | Major concerns | No concerns | Major concerns | Very low |
| CBP+CWMT:TBG+CWMT | Some concerns | Low risk | No concerns | Major concerns | No concerns | Major concerns | Very low |
| CBP+CWMT:XG+CWMT | Major concerns | Low risk | No concerns | Major concerns | No concerns | Major concerns | Very low |
| CBP+CWMT:YG+CWMT | Some concerns | Low risk | No concerns | Major concerns | No concerns | Major concerns | Very low |
| HG+CWMT:NCT+CWMT | No concerns | Low risk | No concerns | Major concerns | No concerns | Major concerns | Very low |
| HG+CWMT:STDP+CWMT | Some concerns | Low risk | No concerns | Major concerns | No concerns | Major concerns | Very low |
| HG+CWMT:TBG+CWMT | Major concerns | Low risk | No concerns | Major concerns | No concerns | Major concerns | Very low |
| HG+CWMT:XG+CWMT | Major concerns | Low risk | No concerns | Major concerns | No concerns | Major concerns | Very low |
| HG+CWMT:YG+CWMT | Some concerns | Low risk | No concerns | Major concerns | No concerns | Major concerns | Very low |
| NCT+CWMT:STDP+CWMT | No concerns | Low risk | No concerns | Major concerns | No concerns | Major concerns | Very low |
| NCT+CWMT:TBG+CWMT | No concerns | Low risk | No concerns | Major concerns | No concerns | Major concerns | Very low |
| NCT+CWMT:XG+CWMT | No concerns | Low risk | No concerns | Major concerns | No concerns | Major concerns | Very low |
| NCT+CWMT:YG+CWMT | No concerns | Low risk | No concerns | Major concerns | No concerns | Major concerns | Very low |
| STDP+CWMT:TBG+CWMT | Some concerns | Low risk | No concerns | Major concerns | No concerns | Major concerns | Very low |
| STDP+CWMT:XG+CWMT | Some concerns | Low risk | No concerns | Major concerns | No concerns | Major concerns | Very low |
| STDP+CWMT:YG+CWMT | Some concerns | Low risk | No concerns | Major concerns | No concerns | Major concerns | Very low |
| TBG+CWMT:XG+CWMT | Major concerns | Low risk | No concerns | Major concerns | No concerns | Major concerns | Very low |
| TBG+CWMT:YG+CWMT | Some concerns | Low risk | No concerns | Major concerns | No concerns | Major concerns | Very low |
| XG+CWMT:YG+CWMT | Some concerns | Low risk | No concerns | Major concerns | No concerns | Major concerns | Very low |

Abbreviations: CWMT , Conventional Western Medicine Treatment ;

NCT ,Nasal Comfort Tablets ;

BYTG , Biyuan Tongqiao Granules ;

CBP , Cang'er Zibi Yan Pills ;

HG , Huaiqi Huang Granules ;

STDP , Sanfeng Tongqiao Dropping Pills ;

TBG , Tongqiao Biyan Granules ;

XG , Xinqin Granules ;

YG , Yuping Feng Granules .

Table S10.6: CINeMA Results of IgE

| Comparison | Within-study bias | Reporting bias | Indirectness | Imprecision | Heterogeneity | Incoherence | Confidence rating |
| --- | --- | --- | --- | --- | --- | --- | --- |
| BYSOL+CWMT:CWMT | Some concerns | Low risk | No concerns | Major concerns | No concerns | Major concerns | Very low |
| BYTG+CWMT:CWMT | Some concerns | Low risk | No concerns | Major concerns | No concerns | Major concerns | Very low |
| CWMT:STDP+CWMT | Some concerns | Low risk | No concerns | Major concerns | No concerns | Major concerns | Very low |
| CWMT:TBG+CWMT | Some concerns | Low risk | No concerns | Major concerns | No concerns | Major concerns | Very low |
| CWMT:XC+CWMT | Some concerns | Low risk | No concerns | Major concerns | No concerns | Major concerns | Very low |
| CWMT:XG+CWMT | Some concerns | Low risk | No concerns | No concerns | No concerns | Major concerns | Moderate |
| CWMT:YG+CWMT | Some concerns | Low risk | No concerns | No concerns | Major concerns | Major concerns | Low |
| BYSOL+CWMT:BYTG+CWMT | Some concerns | Low risk | No concerns | Major concerns | No concerns | Major concerns | Very low |
| BYSOL+CWMT:STDP+CWMT | Some concerns | Low risk | No concerns | Major concerns | No concerns | Major concerns | Very low |
| BYSOL+CWMT:TBG+CWMT | Some concerns | Low risk | No concerns | Major concerns | No concerns | Major concerns | Very low |
| BYSOL+CWMT:XC+CWMT | Some concerns | Low risk | No concerns | Major concerns | No concerns | Major concerns | Very low |
| BYSOL+CWMT:XG+CWMT | Some concerns | Low risk | No concerns | No concerns | No concerns | Major concerns | Moderate |
| BYSOL+CWMT:YG+CWMT | Some concerns | Low risk | No concerns | No concerns | Major concerns | Major concerns | Low |
| BYTG+CWMT:STDP+CWMT | Some concerns | Low risk | No concerns | Major concerns | No concerns | Major concerns | Very low |
| BYTG+CWMT:TBG+CWMT | Some concerns | Low risk | No concerns | Major concerns | No concerns | Major concerns | Very low |
| BYTG+CWMT:XC+CWMT | Some concerns | Low risk | No concerns | Major concerns | No concerns | Major concerns | Very low |
| BYTG+CWMT:XG+CWMT | Some concerns | Low risk | No concerns | No concerns | No concerns | Major concerns | Moderate |
| BYTG+CWMT:YG+CWMT | Some concerns | Low risk | No concerns | No concerns | Major concerns | Major concerns | Low |
| STDP+CWMT:TBG+CWMT | Some concerns | Low risk | No concerns | Major concerns | No concerns | Major concerns | Very low |
| STDP+CWMT:XC+CWMT | Some concerns | Low risk | No concerns | Major concerns | No concerns | Major concerns | Very low |
| STDP+CWMT:XG+CWMT | Some concerns | Low risk | No concerns | No concerns | No concerns | Major concerns | Moderate |
| STDP+CWMT:YG+CWMT | Some concerns | Low risk | No concerns | Major concerns | No concerns | Major concerns | Very low |
| TBG+CWMT:XC+CWMT | Some concerns | Low risk | No concerns | Major concerns | No concerns | Major concerns | Very low |
| TBG+CWMT:XG+CWMT | Some concerns | Low risk | No concerns | No concerns | No concerns | Major concerns | Moderate |
| TBG+CWMT:YG+CWMT | Some concerns | Low risk | No concerns | No concerns | Major concerns | Major concerns | Low |
| XC+CWMT:XG+CWMT | Some concerns | Low risk | No concerns | No concerns | No concerns | Major concerns | Moderate |
| XC+CWMT:YG+CWMT | Some concerns | Low risk | No concerns | No concerns | Major concerns | Major concerns | Low |
| XG+CWMT:YG+CWMT | Some concerns | Low risk | No concerns | No concerns | Major concerns | Major concerns | Low |

Abbreviations: CWMT , Conventional Western Medicine Treatment ;

BYSOL , Biyuan Shu Oral Liquid;

BYTG , Biyuan Tongqiao Granules ;

STDP , Sanfeng Tongqiao Dropping Pills ;

TBG , Tongqiao Biyan Granules ;

XC , Xiangju Capsules ;

XG , Xinqin Granules ;

YG , Yuping Feng Granules .

Table S10.7: CINeMA Results of Effective rate

| Comparison | Within-study bias | Reporting bias | Indirectness | Imprecision | Heterogeneity | Incoherence | Confidence rating |
| --- | --- | --- | --- | --- | --- | --- | --- |
| BYSOL+CWMT:CWMT | Some concerns | Low risk | No concerns | Major concerns | No concerns | Major concerns | Very low |
| BYTG+CWMT:CWMT | Some concerns | Low risk | No concerns | No concerns | Major concerns | Major concerns | Low |
| BZYQP+CWMT:CWMT | Major concerns | Low risk | No concerns | Major concerns | No concerns | Major concerns | Very low |
| CBP+CWMT:CWMT | No concerns | Low risk | No concerns | No concerns | Major concerns | Major concerns | Low |
| CWMT:DYG+CWMT | Major concerns | Low risk | No concerns | Major concerns | No concerns | Major concerns | Very low |
| CWMT:HG+CWMT | Major concerns | Low risk | No concerns | Major concerns | No concerns | Major concerns | Very low |
| CWMT:LQG+CWMT | Some concerns | Low risk | No concerns | Major concerns | No concerns | Major concerns | Very low |
| CWMT:NCT+CWMT | No concerns | Low risk | No concerns | Major concerns | No concerns | Major concerns | Very low |
| CWMT:STDP+CWMT | Some concerns | Low risk | No concerns | Major concerns | No concerns | Major concerns | Very low |
| CWMT:TBG+CWMT | Major concerns | Low risk | No concerns | No concerns | Major concerns | Major concerns | Low |
| CWMT:XC+CWMT | Major concerns | Low risk | No concerns | No concerns | Major concerns | Major concerns | Low |
| CWMT:XG+CWMT | Some concerns | Low risk | No concerns | No concerns | Major concerns | Major concerns | Low |
| CWMT:YG+CWMT | Some concerns | Low risk | No concerns | No concerns | No concerns | Major concerns | Low |
| BYSOL+CWMT:BYTG+CWMT | Some concerns | Low risk | No concerns | Major concerns | No concerns | Major concerns | Very low |
| BYSOL+CWMT:BZYQP+CWMT | Some concerns | Low risk | No concerns | Major concerns | No concerns | Major concerns | Very low |
| BYSOL+CWMT:CBP+CWMT | Some concerns | Low risk | No concerns | Major concerns | No concerns | Major concerns | Very low |
| BYSOL+CWMT:DYG+CWMT | Some concerns | Low risk | No concerns | Major concerns | No concerns | Major concerns | Very low |
| BYSOL+CWMT:HG+CWMT | Some concerns | Low risk | No concerns | Major concerns | No concerns | Major concerns | Very low |
| BYSOL+CWMT:LQG+CWMT | Some concerns | Low risk | No concerns | Major concerns | No concerns | Major concerns | Very low |
| BYSOL+CWMT:NCT+CWMT | No concerns | Low risk | No concerns | Major concerns | No concerns | Major concerns | Very low |
| BYSOL+CWMT:STDP+CWMT | Some concerns | Low risk | No concerns | Major concerns | No concerns | Major concerns | Very low |
| BYSOL+CWMT:TBG+CWMT | Some concerns | Low risk | No concerns | Major concerns | No concerns | Major concerns | Very low |
| BYSOL+CWMT:XC+CWMT | Some concerns | Low risk | No concerns | Major concerns | No concerns | Major concerns | Very low |
| BYSOL+CWMT:XG+CWMT | Some concerns | Low risk | No concerns | Major concerns | No concerns | Major concerns | Very low |
| BYSOL+CWMT:YG+CWMT | Some concerns | Low risk | No concerns | Major concerns | No concerns | Major concerns | Very low |
| BYTG+CWMT:BZYQP+CWMT | Major concerns | Low risk | No concerns | Major concerns | No concerns | Major concerns | Very low |
| BYTG+CWMT:CBP+CWMT | No concerns | Low risk | No concerns | Major concerns | No concerns | Major concerns | Very low |
| BYTG+CWMT:DYG+CWMT | Major concerns | Low risk | No concerns | Major concerns | No concerns | Major concerns | Very low |
| BYTG+CWMT:HG+CWMT | Major concerns | Low risk | No concerns | Major concerns | No concerns | Major concerns | Very low |
| BYTG+CWMT:LQG+CWMT | Some concerns | Low risk | No concerns | Major concerns | No concerns | Major concerns | Very low |
| BYTG+CWMT:NCT+CWMT | No concerns | Low risk | No concerns | Major concerns | No concerns | Major concerns | Very low |
| BYTG+CWMT:STDP+CWMT | Some concerns | Low risk | No concerns | Major concerns | No concerns | Major concerns | Very low |
| BYTG+CWMT:TBG+CWMT | Some concerns | Low risk | No concerns | Major concerns | No concerns | Major concerns | Very low |
| BYTG+CWMT:XC+CWMT | Some concerns | Low risk | No concerns | Major concerns | No concerns | Major concerns | Very low |
| BYTG+CWMT:XG+CWMT | Some concerns | Low risk | No concerns | Major concerns | No concerns | Major concerns | Very low |
| BYTG+CWMT:YG+CWMT | Some concerns | Low risk | No concerns | Major concerns | No concerns | Major concerns | Very low |
| BZYQP+CWMT:CBP+CWMT | Major concerns | Low risk | No concerns | Major concerns | No concerns | Major concerns | Very low |
| BZYQP+CWMT:DYG+CWMT | Major concerns | Low risk | No concerns | Major concerns | No concerns | Major concerns | Very low |
| BZYQP+CWMT:HG+CWMT | Major concerns | Low risk | No concerns | Major concerns | No concerns | Major concerns | Very low |
| BZYQP+CWMT:LQG+CWMT | Some concerns | Low risk | No concerns | Major concerns | No concerns | Major concerns | Very low |
| BZYQP+CWMT:NCT+CWMT | No concerns | Low risk | No concerns | Major concerns | No concerns | Major concerns | Very low |
| BZYQP+CWMT:STDP+CWMT | Some concerns | Low risk | No concerns | Major concerns | No concerns | Major concerns | Very low |
| BZYQP+CWMT:TBG+CWMT | Major concerns | Low risk | No concerns | Major concerns | No concerns | Major concerns | Very low |
| BZYQP+CWMT:XC+CWMT | Major concerns | Low risk | No concerns | Major concerns | No concerns | Major concerns | Very low |
| BZYQP+CWMT:XG+CWMT | Major concerns | Low risk | No concerns | Major concerns | No concerns | Major concerns | Very low |
| BZYQP+CWMT:YG+CWMT | Major concerns | Low risk | No concerns | Major concerns | No concerns | Major concerns | Very low |
| CBP+CWMT:DYG+CWMT | Major concerns | Low risk | No concerns | Major concerns | No concerns | Major concerns | Very low |
| CBP+CWMT:HG+CWMT | Major concerns | Low risk | No concerns | Major concerns | No concerns | Major concerns | Very low |
| CBP+CWMT:LQG+CWMT | Some concerns | Low risk | No concerns | Major concerns | No concerns | Major concerns | Very low |
| CBP+CWMT:NCT+CWMT | No concerns | Low risk | No concerns | Major concerns | No concerns | Major concerns | Very low |
| CBP+CWMT:STDP+CWMT | Some concerns | Low risk | No concerns | Major concerns | No concerns | Major concerns | Very low |
| CBP+CWMT:TBG+CWMT | No concerns | Low risk | No concerns | Major concerns | No concerns | Major concerns | Very low |
| CBP+CWMT:XC+CWMT | No concerns | Low risk | No concerns | Major concerns | No concerns | Major concerns | Very low |
| CBP+CWMT:XG+CWMT | No concerns | Low risk | No concerns | Major concerns | No concerns | Major concerns | Very low |
| CBP+CWMT:YG+CWMT | No concerns | Low risk | No concerns | Major concerns | No concerns | Major concerns | Very low |
| DYG+CWMT:HG+CWMT | Major concerns | Low risk | No concerns | Major concerns | No concerns | Major concerns | Very low |
| DYG+CWMT:LQG+CWMT | Some concerns | Low risk | No concerns | Major concerns | No concerns | Major concerns | Very low |
| DYG+CWMT:NCT+CWMT | No concerns | Low risk | No concerns | Major concerns | No concerns | Major concerns | Very low |
| DYG+CWMT:STDP+CWMT | Some concerns | Low risk | No concerns | Major concerns | No concerns | Major concerns | Very low |
| DYG+CWMT:TBG+CWMT | Major concerns | Low risk | No concerns | Major concerns | No concerns | Major concerns | Very low |
| DYG+CWMT:XC+CWMT | Major concerns | Low risk | No concerns | Major concerns | No concerns | Major concerns | Very low |
| DYG+CWMT:XG+CWMT | Major concerns | Low risk | No concerns | Major concerns | No concerns | Major concerns | Very low |
| DYG+CWMT:YG+CWMT | Major concerns | Low risk | No concerns | Major concerns | No concerns | Major concerns | Very low |
| HG+CWMT:LQG+CWMT | Some concerns | Low risk | No concerns | Major concerns | No concerns | Major concerns | Very low |
| HG+CWMT:NCT+CWMT | No concerns | Low risk | No concerns | Major concerns | No concerns | Major concerns | Very low |
| HG+CWMT:STDP+CWMT | Some concerns | Low risk | No concerns | Major concerns | No concerns | Major concerns | Very low |
| HG+CWMT:TBG+CWMT | Major concerns | Low risk | No concerns | Major concerns | No concerns | Major concerns | Very low |
| HG+CWMT:XC+CWMT | Major concerns | Low risk | No concerns | Major concerns | No concerns | Major concerns | Very low |
| HG+CWMT:XG+CWMT | Major concerns | Low risk | No concerns | Major concerns | No concerns | Major concerns | Very low |
| HG+CWMT:YG+CWMT | Major concerns | Low risk | No concerns | Major concerns | No concerns | Major concerns | Very low |
| LQG+CWMT:NCT+CWMT | No concerns | Low risk | No concerns | Major concerns | No concerns | Major concerns | Very low |
| LQG+CWMT:STDP+CWMT | Some concerns | Low risk | No concerns | Major concerns | No concerns | Major concerns | Very low |
| LQG+CWMT:TBG+CWMT | Some concerns | Low risk | No concerns | Major concerns | No concerns | Major concerns | Very low |
| LQG+CWMT:XC+CWMT | Some concerns | Low risk | No concerns | Major concerns | No concerns | Major concerns | Very low |
| LQG+CWMT:XG+CWMT | Some concerns | Low risk | No concerns | Major concerns | No concerns | Major concerns | Very low |
| LQG+CWMT:YG+CWMT | Some concerns | Low risk | No concerns | Major concerns | No concerns | Major concerns | Very low |
| NCT+CWMT:STDP+CWMT | No concerns | Low risk | No concerns | Major concerns | No concerns | Major concerns | Very low |
| NCT+CWMT:TBG+CWMT | No concerns | Low risk | No concerns | Major concerns | No concerns | Major concerns | Very low |
| NCT+CWMT:XC+CWMT | No concerns | Low risk | No concerns | Major concerns | No concerns | Major concerns | Very low |
| NCT+CWMT:XG+CWMT | No concerns | Low risk | No concerns | Major concerns | No concerns | Major concerns | Very low |
| NCT+CWMT:YG+CWMT | No concerns | Low risk | No concerns | Major concerns | No concerns | Major concerns | Very low |
| STDP+CWMT:TBG+CWMT | Some concerns | Low risk | No concerns | Major concerns | No concerns | Major concerns | Very low |
| STDP+CWMT:XC+CWMT | Some concerns | Low risk | No concerns | Major concerns | No concerns | Major concerns | Very low |
| STDP+CWMT:XG+CWMT | Some concerns | Low risk | No concerns | Major concerns | No concerns | Major concerns | Very low |
| STDP+CWMT:YG+CWMT | Some concerns | Low risk | No concerns | Major concerns | No concerns | Major concerns | Very low |
| TBG+CWMT:XC+CWMT | Major concerns | Low risk | No concerns | Major concerns | No concerns | Major concerns | Very low |
| TBG+CWMT:XG+CWMT | Major concerns | Low risk | No concerns | Major concerns | No concerns | Major concerns | Very low |
| TBG+CWMT:YG+CWMT | Major concerns | Low risk | No concerns | Major concerns | No concerns | Major concerns | Very low |
| XC+CWMT:XG+CWMT | Major concerns | Low risk | No concerns | Major concerns | No concerns | Major concerns | Very low |
| XC+CWMT:YG+CWMT | Major concerns | Low risk | No concerns | Major concerns | No concerns | Major concerns | Very low |
| XG+CWMT:YG+CWMT | Some concerns | Low risk | No concerns | Major concerns | No concerns | Major concerns | Very low |

Abbreviations: CWMT , Conventional Western Medicine Treatment ;

NCT ,Nasal Comfort Tablets ;

BYSOL , Biyuan Shu Oral Liquid;

BYTG , Biyuan Tongqiao Granules ;

BZYQP , Buzhong Yiqi Pills ;

CBP , Cang'er Zibi Yan Pills ;

DYG , Danxi Yuping Feng Granules ;

HG , Huaiqi Huang Granules ;

LQG , Lianhua Qingwen Granules ;

STDP , Sanfeng Tongqiao Dropping Pills ;

TBG , Tongqiao Biyan Granules ;

XC , Xiangju Capsules ;

XG , Xinqin Granules ;

YG , Yuping Feng Granules .

Table S10.8: CINeMA Results of Adverse events

| Comparison | Within-study bias | Reporting bias | Indirectness | Imprecision | Heterogeneity | Incoherence | Confidence rating |
| --- | --- | --- | --- | --- | --- | --- | --- |
| BYSOL+CWMT:CWMT | Some concerns | Low risk | No concerns | Major concerns | No concerns | Major concerns | Very low |
| BYTG+CWMT:CWMT | Some concerns | Low risk | No concerns | No concerns | Major concerns | Major concerns | Low |
| BZYQP+CWMT:CWMT | Major concerns | Low risk | No concerns | Major concerns | No concerns | Major concerns | Very low |
| CBP+CWMT:CWMT | Some concerns | Low risk | No concerns | Major concerns | No concerns | Major concerns | Very low |
| CWMT:LQG+CWMT | Some concerns | Low risk | No concerns | Major concerns | No concerns | Major concerns | Very low |
| CWMT:STDP+CWMT | Some concerns | Low risk | No concerns | Major concerns | No concerns | Major concerns | Very low |
| CWMT:TBG+CWMT | Major concerns | Low risk | No concerns | No concerns | Major concerns | Major concerns | Low |
| CWMT:XC+CWMT | Some concerns | Low risk | No concerns | Major concerns | No concerns | Major concerns | Very low |
| CWMT:XG+CWMT | Some concerns | Low risk | No concerns | Major concerns | No concerns | Major concerns | Very low |
| CWMT:YG+CWMT | No concerns | Low risk | No concerns | No concerns | No concerns | Major concerns | Moderate |
| BYSOL+CWMT:BYTG+CWMT | Some concerns | Low risk | No concerns | Major concerns | No concerns | Major concerns | Very low |
| BYSOL+CWMT:BZYQP+CWMT | Some concerns | Low risk | No concerns | No concerns | Major concerns | Major concerns | Low |
| BYSOL+CWMT:CBP+CWMT | Some concerns | Low risk | No concerns | Major concerns | No concerns | Major concerns | Very low |
| BYSOL+CWMT:LQG+CWMT | Some concerns | Low risk | No concerns | Major concerns | No concerns | Major concerns | Very low |
| BYSOL+CWMT:STDP+CWMT | Some concerns | Low risk | No concerns | Major concerns | No concerns | Major concerns | Very low |
| BYSOL+CWMT:TBG+CWMT | Some concerns | Low risk | No concerns | Major concerns | No concerns | Major concerns | Very low |
| BYSOL+CWMT:XC+CWMT | Some concerns | Low risk | No concerns | Major concerns | No concerns | Major concerns | Very low |
| BYSOL+CWMT:XG+CWMT | Some concerns | Low risk | No concerns | Major concerns | No concerns | Major concerns | Very low |
| BYSOL+CWMT:YG+CWMT | Some concerns | Low risk | No concerns | Major concerns | No concerns | Major concerns | Very low |
| BYTG+CWMT:BZYQP+CWMT | Major concerns | Low risk | No concerns | Major concerns | No concerns | Major concerns | Very low |
| BYTG+CWMT:CBP+CWMT | Some concerns | Low risk | No concerns | Major concerns | No concerns | Major concerns | Very low |
| BYTG+CWMT:LQG+CWMT | Some concerns | Low risk | No concerns | Major concerns | No concerns | Major concerns | Very low |
| BYTG+CWMT:STDP+CWMT | Some concerns | Low risk | No concerns | Major concerns | No concerns | Major concerns | Very low |
| BYTG+CWMT:TBG+CWMT | Some concerns | Low risk | No concerns | Major concerns | No concerns | Major concerns | Very low |
| BYTG+CWMT:XC+CWMT | Some concerns | Low risk | No concerns | Major concerns | No concerns | Major concerns | Very low |
| BYTG+CWMT:XG+CWMT | Some concerns | Low risk | No concerns | Major concerns | No concerns | Major concerns | Very low |
| BYTG+CWMT:YG+CWMT | Some concerns | Low risk | No concerns | Major concerns | No concerns | Major concerns | Very low |
| BZYQP+CWMT:CBP+CWMT | Some concerns | Low risk | No concerns | Major concerns | No concerns | Major concerns | Very low |
| BZYQP+CWMT:LQG+CWMT | Some concerns | Low risk | No concerns | Major concerns | No concerns | Major concerns | Very low |
| BZYQP+CWMT:STDP+CWMT | Some concerns | Low risk | No concerns | Major concerns | No concerns | Major concerns | Very low |
| BZYQP+CWMT:TBG+CWMT | Major concerns | Low risk | No concerns | Major concerns | No concerns | Major concerns | Very low |
| BZYQP+CWMT:XC+CWMT | Major concerns | Low risk | No concerns | Major concerns | No concerns | Major concerns | Very low |
| BZYQP+CWMT:XG+CWMT | Some concerns | Low risk | No concerns | Major concerns | No concerns | Major concerns | Very low |
| BZYQP+CWMT:YG+CWMT | Major concerns | Low risk | No concerns | No concerns | No concerns | Major concerns | Low |
| CBP+CWMT:LQG+CWMT | Some concerns | Low risk | No concerns | Major concerns | No concerns | Major concerns | Very low |
| CBP+CWMT:STDP+CWMT | Some concerns | Low risk | No concerns | Major concerns | No concerns | Major concerns | Very low |
| CBP+CWMT:TBG+CWMT | Some concerns | Low risk | No concerns | Major concerns | No concerns | Major concerns | Very low |
| CBP+CWMT:XC+CWMT | Some concerns | Low risk | No concerns | Major concerns | No concerns | Major concerns | Very low |
| CBP+CWMT:XG+CWMT | Some concerns | Low risk | No concerns | Major concerns | No concerns | Major concerns | Very low |
| CBP+CWMT:YG+CWMT | Some concerns | Low risk | No concerns | Major concerns | No concerns | Major concerns | Very low |
| LQG+CWMT:STDP+CWMT | Some concerns | Low risk | No concerns | Major concerns | No concerns | Major concerns | Very low |
| LQG+CWMT:TBG+CWMT | Some concerns | Low risk | No concerns | Major concerns | No concerns | Major concerns | Very low |
| LQG+CWMT:XC+CWMT | Some concerns | Low risk | No concerns | Major concerns | No concerns | Major concerns | Very low |
| LQG+CWMT:XG+CWMT | Some concerns | Low risk | No concerns | Major concerns | No concerns | Major concerns | Very low |
| LQG+CWMT:YG+CWMT | Some concerns | Low risk | No concerns | Major concerns | No concerns | Major concerns | Very low |
| STDP+CWMT:TBG+CWMT | Some concerns | Low risk | No concerns | Major concerns | No concerns | Major concerns | Very low |
| STDP+CWMT:XC+CWMT | Some concerns | Low risk | No concerns | Major concerns | No concerns | Major concerns | Very low |
| STDP+CWMT:XG+CWMT | Some concerns | Low risk | No concerns | Major concerns | No concerns | Major concerns | Very low |
| STDP+CWMT:YG+CWMT | Some concerns | Low risk | No concerns | Major concerns | No concerns | Major concerns | Very low |
| TBG+CWMT:XC+CWMT | Major concerns | Low risk | No concerns | Major concerns | No concerns | Major concerns | Very low |
| TBG+CWMT:XG+CWMT | Some concerns | Low risk | No concerns | Major concerns | No concerns | Major concerns | Very low |
| TBG+CWMT:YG+CWMT | Some concerns | Low risk | No concerns | Major concerns | No concerns | Major concerns | Very low |
| XC+CWMT:XG+CWMT | Some concerns | Low risk | No concerns | Major concerns | No concerns | Major concerns | Very low |
| XC+CWMT:YG+CWMT | Some concerns | Low risk | No concerns | Major concerns | No concerns | Major concerns | Very low |
| XG+CWMT:YG+CWMT | Some concerns | Low risk | No concerns | Major concerns | No concerns | Major concerns | Very low |

Abbreviations: CWMT , Conventional Western Medicine Treatment ;

BYSOL , Biyuan Shu Oral Liquid;

BYTG , Biyuan Tongqiao Granules ;

BZYQP , Buzhong Yiqi Pills ;

CBP , Cang'er Zibi Yan Pills ;

LQG , Lianhua Qingwen Granules ;

STDP , Sanfeng Tongqiao Dropping Pills ;

TBG , Tongqiao Biyan Granules ;

XC , Xiangju Capsules ;

XG , Xinqin Granules ;

YG , Yuping Feng Granules .

Table S10.9: CINeMA Results of Recurrence Rate

| Comparison | Within-study bias | Reporting bias | Indirectness | Imprecision | Heterogeneity | Incoherence | Confidence rating |
| --- | --- | --- | --- | --- | --- | --- | --- |
| BYTG+CWMT:CWMT | Some concerns | Low risk | No concerns | No concerns | No concerns | Major concerns | Moderate |
| CWMT:DYG+CWMT | Major concerns | Low risk | No concerns | No concerns | Major concerns | Major concerns | Very low |
| CWMT:HG+CWMT | Major concerns | Low risk | No concerns | No concerns | Major concerns | Major concerns | Very low |
| CWMT:TBG+CWMT | Major concerns | Low risk | No concerns | Major concerns | No concerns | Major concerns | Very low |
| CWMT:XG+CWMT | Major concerns | Low risk | No concerns | Major concerns | No concerns | Major concerns | Very low |
| CWMT:YG+CWMT | Major concerns | Low risk | No concerns | No concerns | No concerns | Major concerns | Very low |
| BYTG+CWMT:DYG+CWMT | Major concerns | Low risk | No concerns | Major concerns | No concerns | Major concerns | Very low |
| BYTG+CWMT:HG+CWMT | Major concerns | Low risk | No concerns | Major concerns | No concerns | Major concerns | Very low |
| BYTG+CWMT:TBG+CWMT | Major concerns | Low risk | No concerns | Major concerns | No concerns | Major concerns | Very low |
| BYTG+CWMT:XG+CWMT | Major concerns | Low risk | No concerns | Major concerns | No concerns | Major concerns | Very low |
| BYTG+CWMT:YG+CWMT | Some concerns | Low risk | No concerns | Major concerns | No concerns | Major concerns | Very low |
| DYG+CWMT:HG+CWMT | Major concerns | Low risk | No concerns | Major concerns | No concerns | Major concerns | Very low |
| DYG+CWMT:TBG+CWMT | Major concerns | Low risk | No concerns | Major concerns | No concerns | Major concerns | Very low |
| DYG+CWMT:XG+CWMT | Major concerns | Low risk | No concerns | Major concerns | No concerns | Major concerns | Very low |
| DYG+CWMT:YG+CWMT | Major concerns | Low risk | No concerns | Major concerns | No concerns | Major concerns | Very low |
| HG+CWMT:TBG+CWMT | Major concerns | Low risk | No concerns | Major concerns | No concerns | Major concerns | Very low |
| HG+CWMT:XG+CWMT | Major concerns | Low risk | No concerns | Major concerns | No concerns | Major concerns | Very low |
| HG+CWMT:YG+CWMT | Major concerns | Low risk | No concerns | Major concerns | No concerns | Major concerns | Very low |
| TBG+CWMT:XG+CWMT | Major concerns | Low risk | No concerns | Major concerns | No concerns | Major concerns | Very low |
| TBG+CWMT:YG+CWMT | Major concerns | Low risk | No concerns | Major concerns | No concerns | Major concerns | Very low |
| XG+CWMT:YG+CWMT | Major concerns | Low risk | No concerns | Major concerns | No concerns | Major concerns | Very low |

Abbreviations: CWMT , Conventional Western Medicine Treatment ;

BYTG , Biyuan Tongqiao Granules ;

DYG , Danxi Yuping Feng Granules ;

HG , Huaiqi Huang Granules ;

TBG , Tongqiao Biyan Granules ;

XG , Xinqin Granules ;

YG , Yuping Feng Granules.

**Appendix 11: Funnel plots**

The funnel plot illustrates the assessment of small study effect bias in studies investigating the effects of various Chinese patent medicines combined with conventional Western drugs versus conventional Western drug monotherapy on pediatric allergic rhinitis. The figure encompasses all included trials that compared at least one combination therapy with conventional Western drug alone as the control.

**Figure S11.1**: Funnel plot of Nasal obstruction


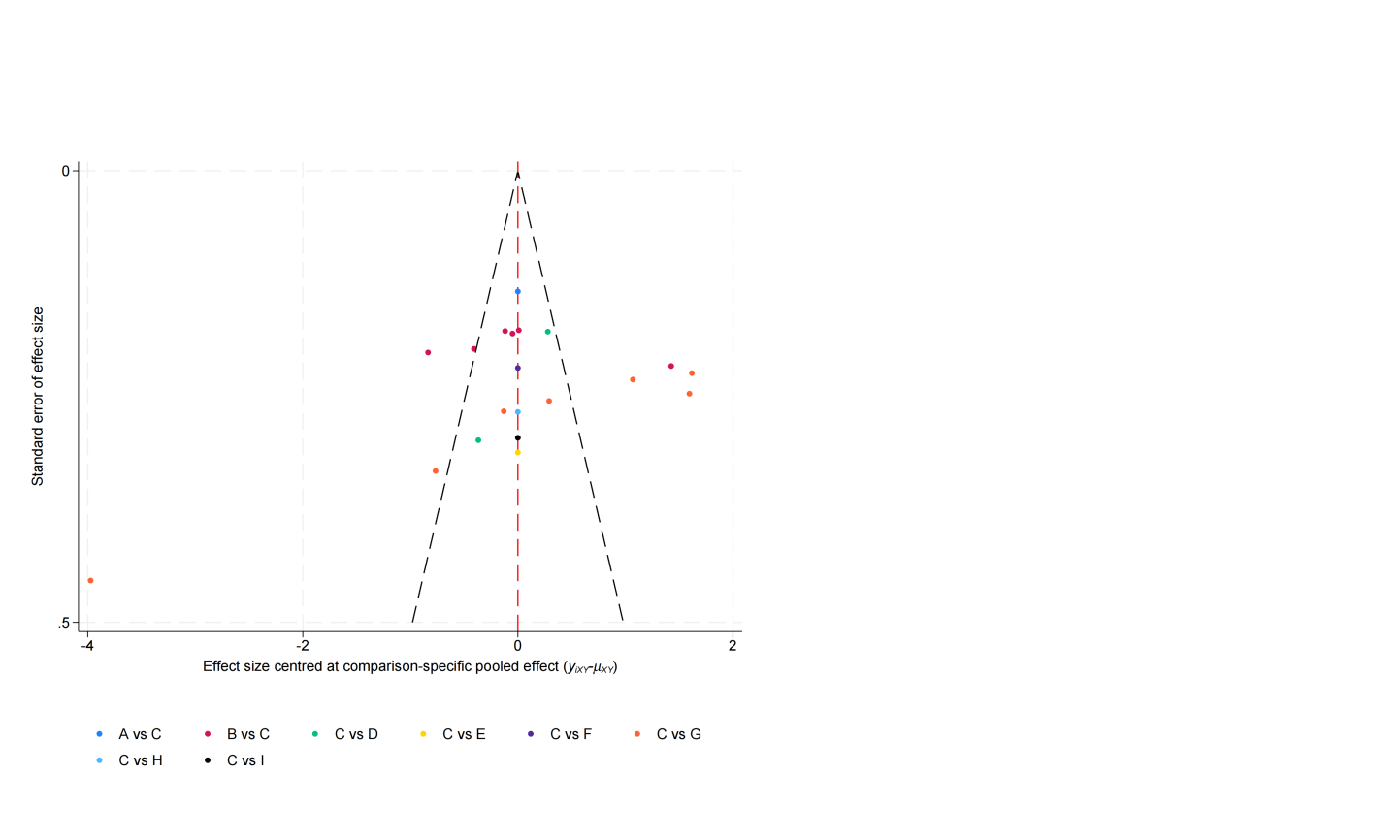


Abbreviations: CWMT , Conventional Western Medicine Treatment ;

A , Nasal Comfort Tablets + CWMT ;

B, Biyuan Tongqiao Granules + CWMT ;

C, CWMT ;

D, Cang'er Zibi Yan Pills + CWMT ;

E, Huaiqi Huang Granules + CWMT ;

F, Sanfeng Tongqiao Dropping Pills + CWMT ;

G, Tongqiao Biyan Granules + CWMT ;

H, Xinqin Granules + CWMT ;

I, Yuping Feng Granules + CWMT .

**Figure S11.2:** Funnel plot of Nasal pruritus


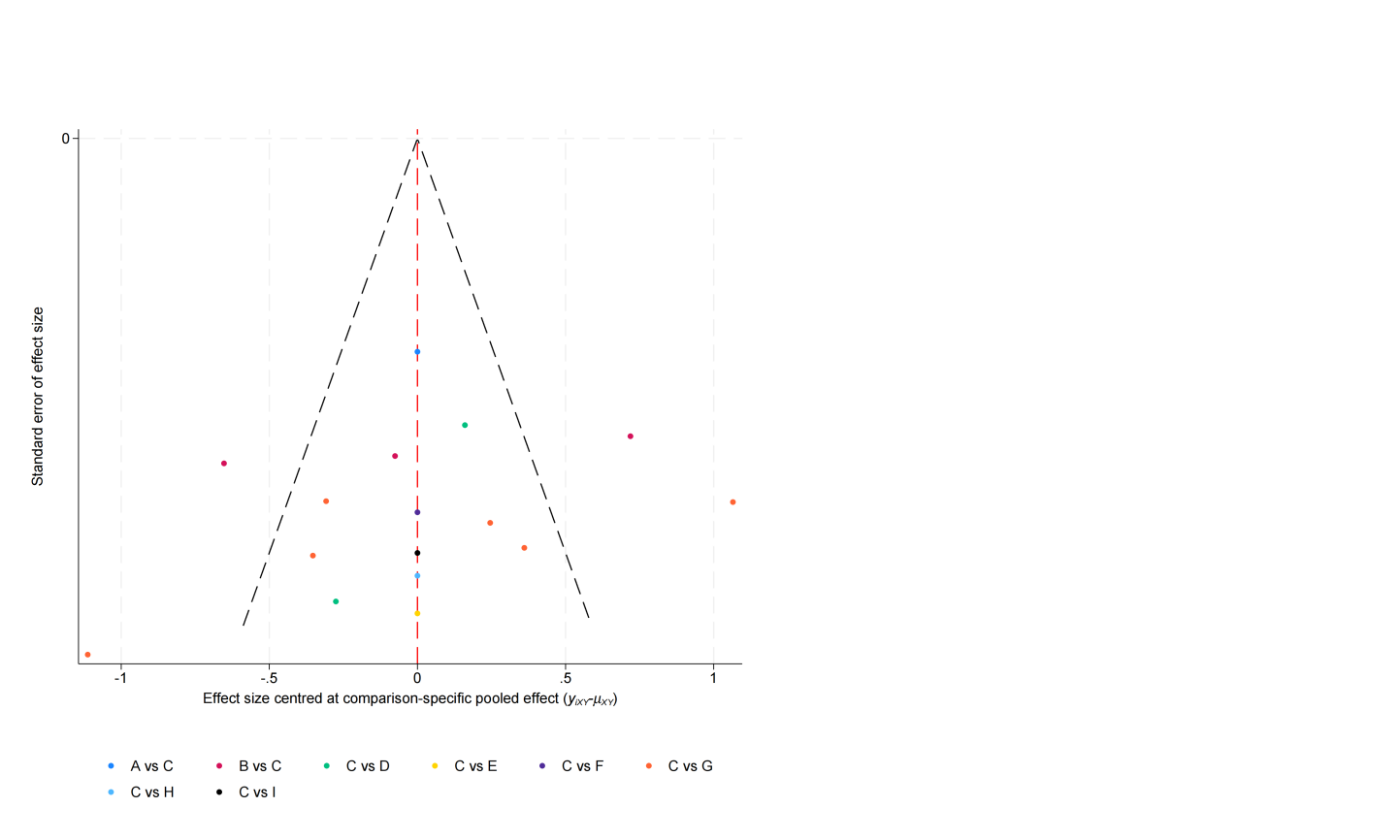


Abbreviations: CWMT , Conventional Western Medicine Treatment ;

A, Nasal Comfort Tablets + CWMT ;

B, Biyuan Tongqiao Granules + CWMT ;

C, CWMT ;

D, Cang'er Zibi Yan Pills + CWMT ;

E, Huaiqi Huang Granules + CWMT ;

F, Sanfeng Tongqiao Dropping Pills + CWMT ;

G, Tongqiao Biyan Granules + CWMT ;

H, Xinqin Granules + CWMT ;

I, Yuping Feng Granules + CWMT .

**Figure S11.3**: Funnel plot of Paroxysmal sneezing


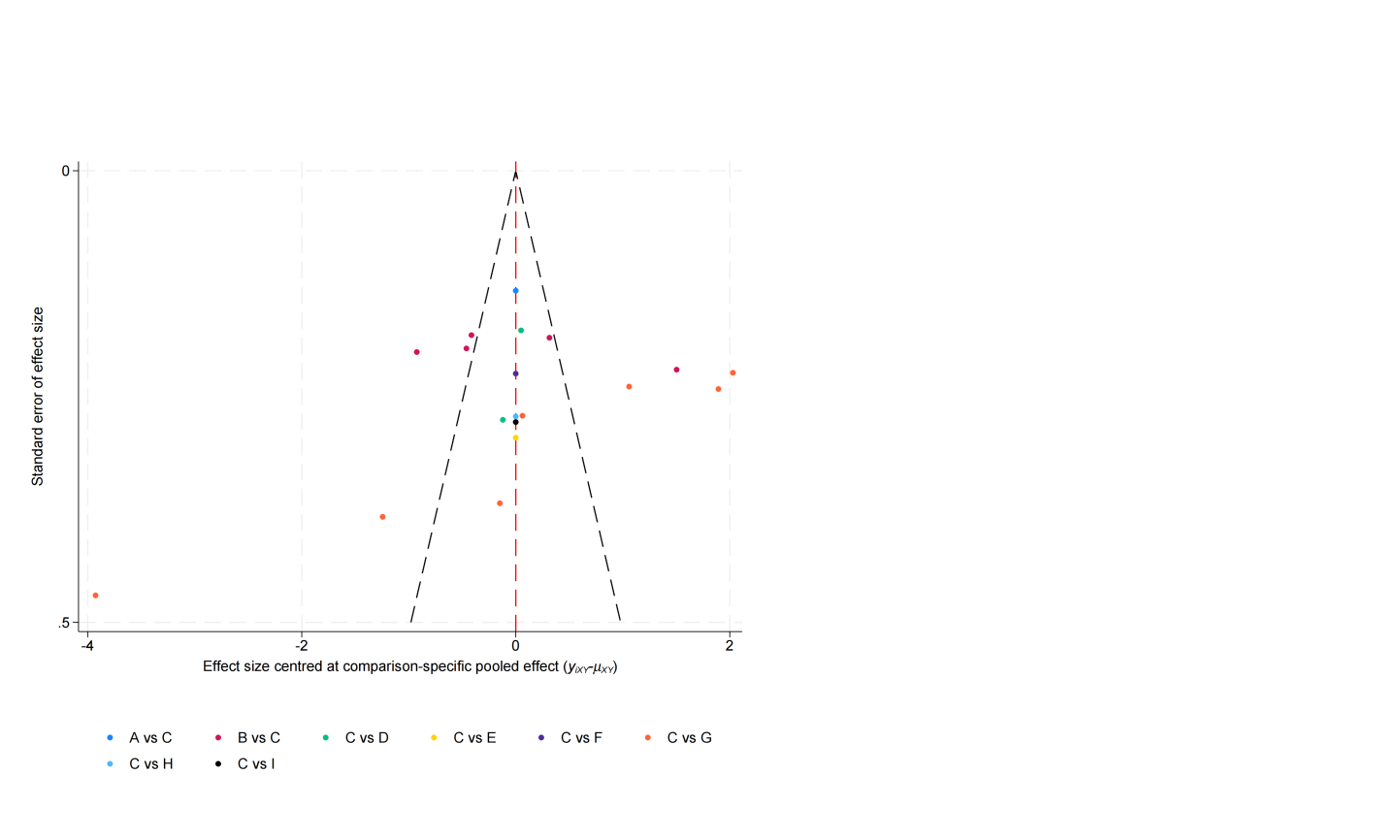


Abbreviations: CWMT , Conventional Western Medicine Treatment ;

A, Nasal Comfort Tablets + CWMT ;

B, Biyuan Tongqiao Granules + CWMT ;

C, CWMT ;

D, Cang'er Zibi Yan Pills + CWMT ;

E, Huaiqi Huang Granules + CWMT ;

F, Sanfeng Tongqiao Dropping Pills + CWMT ;

G, Tongqiao Biyan Granules + CWMT ;

H, Xinqin Granules + CWMT ;

I, Yuping Feng Granules + CWMT .

**Figure S11.4**, Funnel plot of Nasal discharge


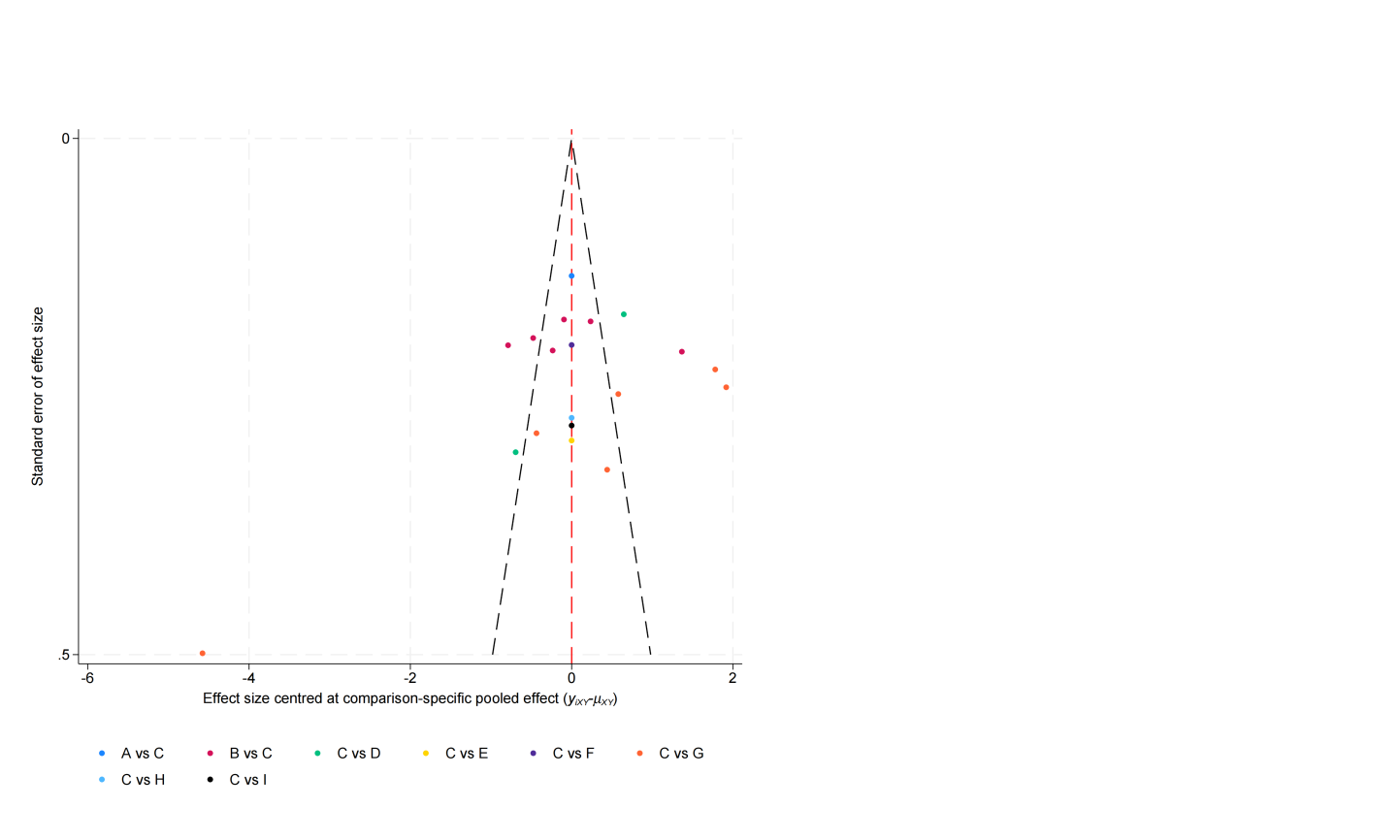


Abbreviations, CWMT , Conventional Western Medicine Treatment ;

A, Nasal Comfort Tablets + CWMT ;

B, Biyuan Tongqiao Granules + CWMT ;

C, CWMT ;

D, Cang'er Zibi Yan Pills + CWMT ;

E, Huaiqi Huang Granules + CWMT ;

F, Sanfeng Tongqiao Dropping Pills + CWMT ;

G, Tongqiao Biyan Granules + CWMT ;

H, Xinqin Granules + CWMT ;

I, Yuping Feng Granules + CWMT .

**Figure S11.5**: Funnel plot of IgE


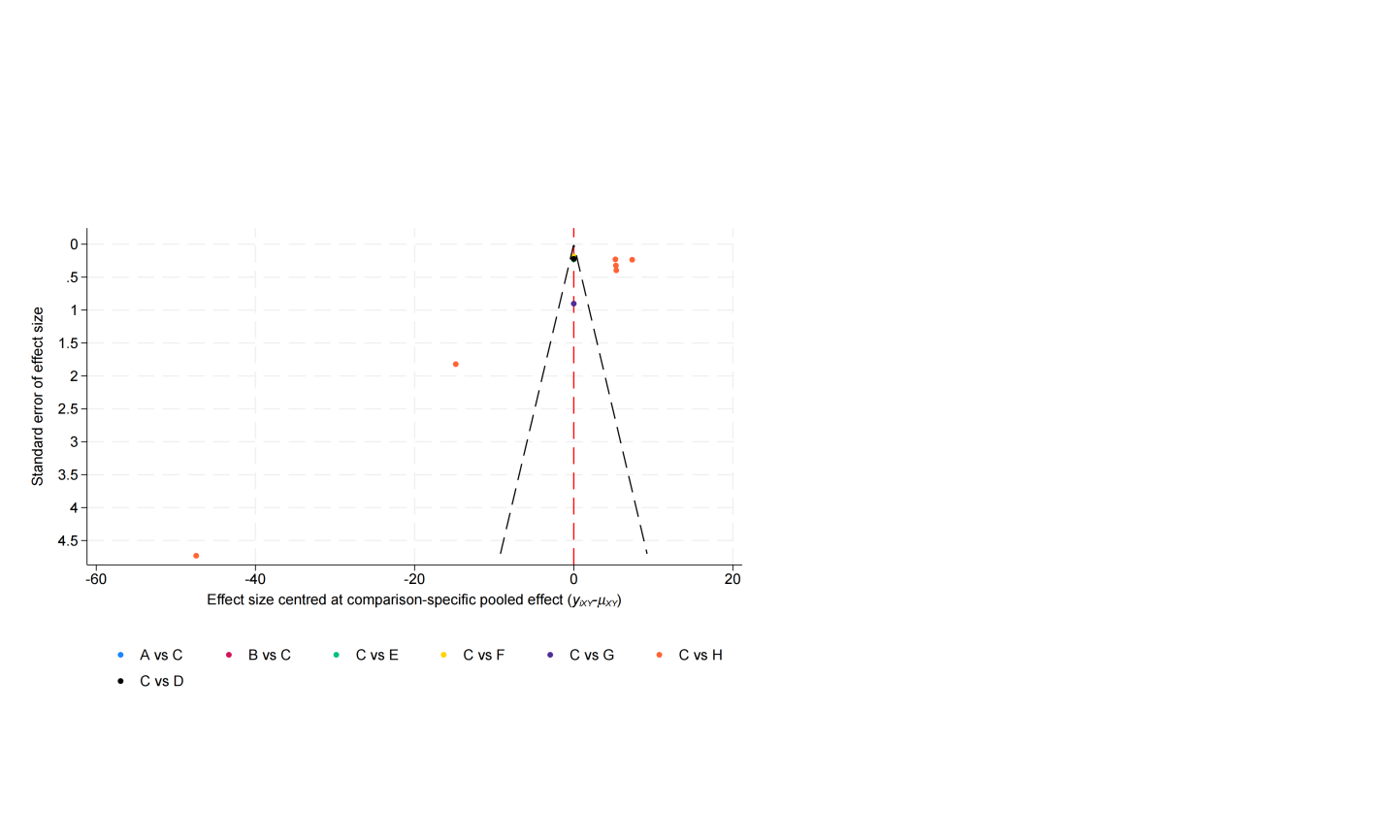


Abbreviations: CWMT , Conventional Western Medicine Treatment ;

A, Biyuan Shu Oral Liquid+ CWMT ;

B, Biyuan Tongqiao Granules + CWMT ;

C, CWMT ;

D, Sanfeng Tongqiao Dropping Pills + CWMT ;

E, Tongqiao Biyan Granules + CWMT ;

F, Xiangju Capsules + CWMT ;

G, Xinqin Granules + CWMT ;

H, Yuping Feng Granules + CWMT .

**Figure S11.6**: Funnel plot of Effective rate


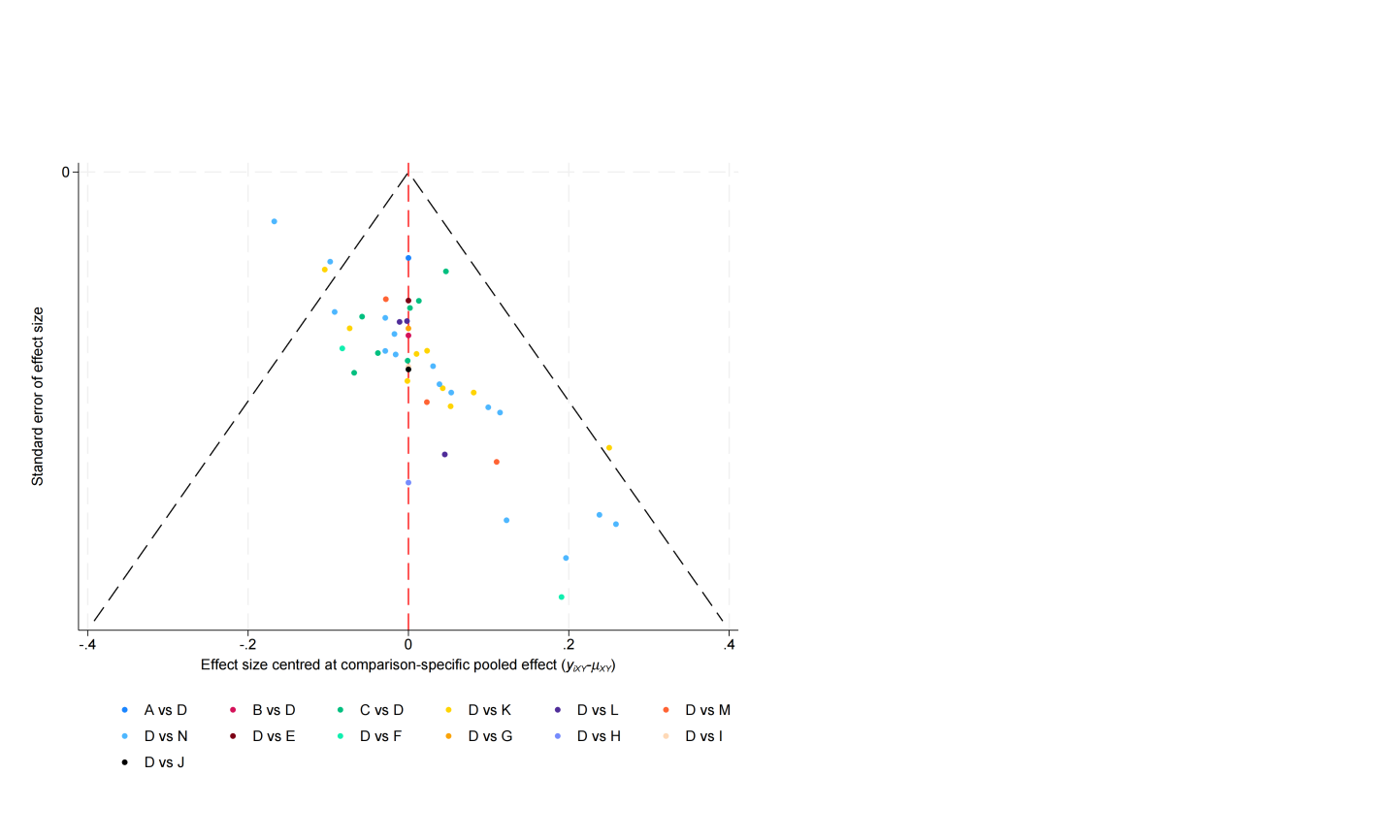


Abbreviations: CWMT , Conventional Western Medicine Treatment ;

A, Nasal Comfort Tablets + CWMT ;

B, Biyuan Shu Oral Liquid+ CWMT ;

C, Biyuan Tongqiao Granules + CWMT ;

D, CWMT ;

E, Buzhong Yiqi Pills + CWMT ;

F, Cang'er Zibi Yan Pills + CWMT ;

G, Danxi Yuping Feng Granules + CWMT ;

H, Huaiqi Huang Granules + CWMT ;

I, Lianhua Qingwen Granules + CWMT ;

J, Sanfeng Tongqiao Dropping Pills + CWMT ;

K, Tongqiao Biyan Granules + CWMT ;

L, Xiangju Capsules + CWMT ;

M, Xinqin Granules + CWMT ;

N, Yuping Feng Granules + CWMT ;

**Figure S11.7**: Funnel plot of Adverse events


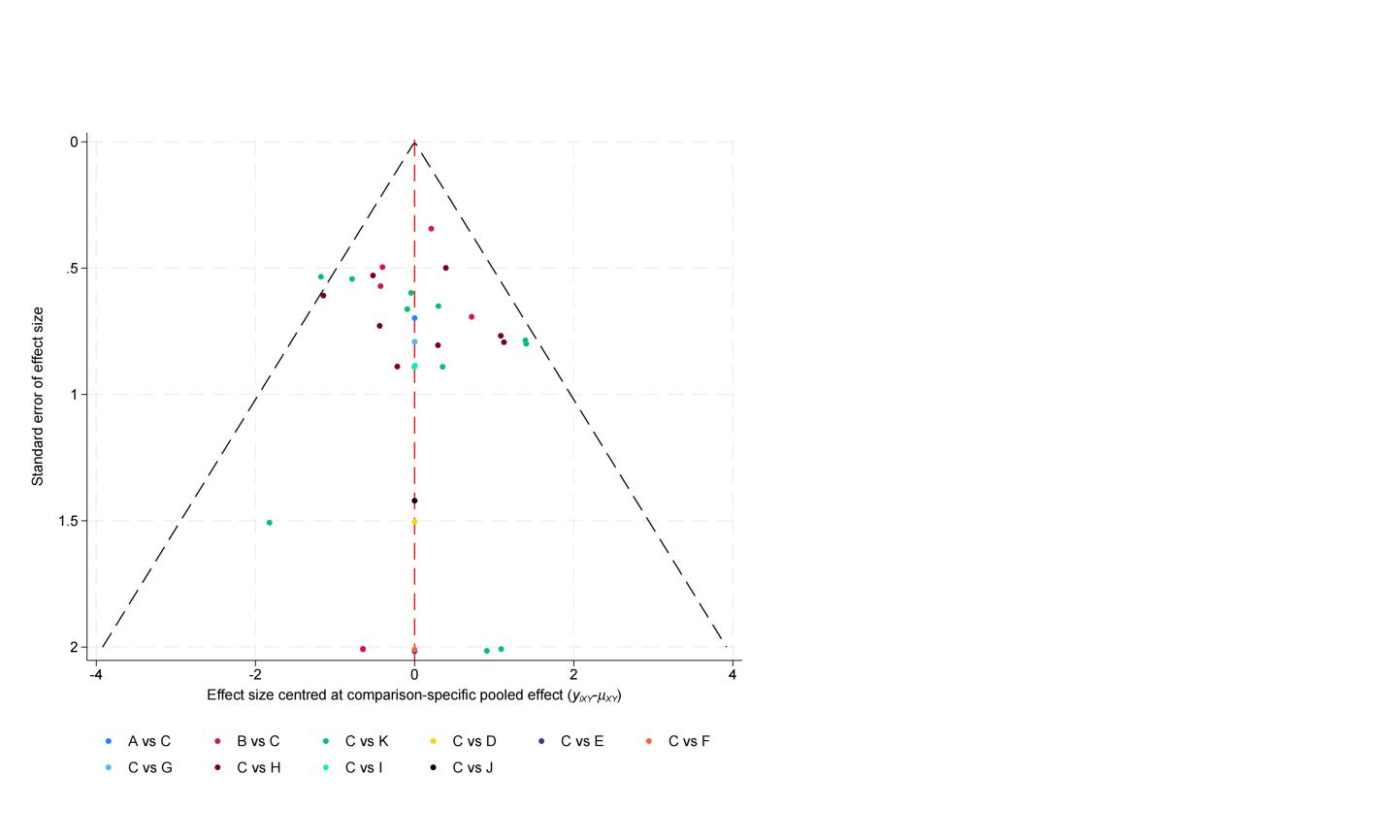


Abbreviations: CWMT , Conventional Western Medicine Treatment ;

A： Biyuan Shu Oral Liquid+ CWMT ;

B, Biyuan Tongqiao Granules + CWMT ;

C, CWMT ;

D, Buzhong Yiqi Pills + CWMT ;

E, Cang'er Zibi Yan Pills + CWMT ;

F, Lianhua Qingwen Granules + CWMT ;

G, Sanfeng Tongqiao Dropping Pills + CWMT ;

H, Tongqiao Biyan Granules + CWMT ;

I, Xiangju Capsules + CWMT ;

J, Xinqin Granules + CWMT ;

K, Yuping Feng Granules + CWMT .

**Figure S11.8:** Funnel plot of Recurrence Rate


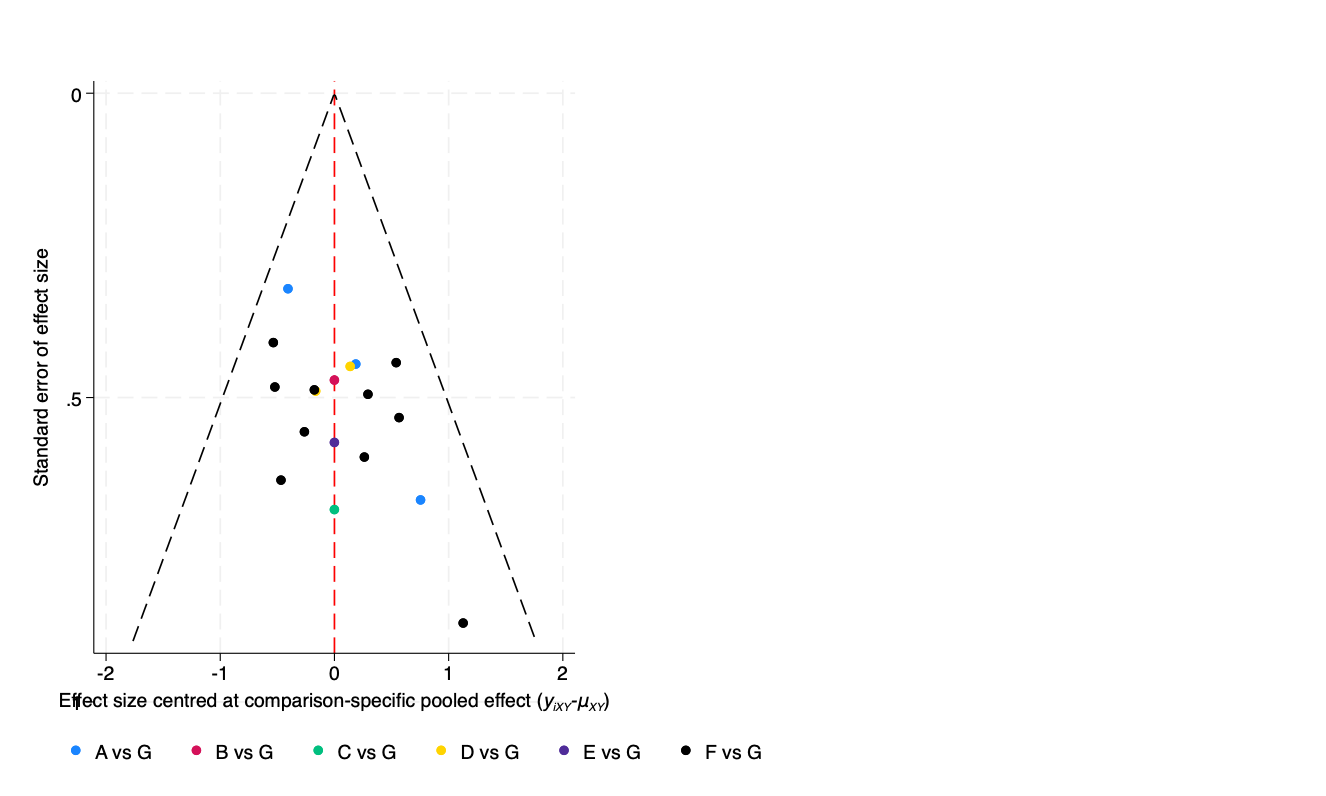


Abbreviations: CWMT , Conventional Western Medicine Treatment ;

A, Biyuan Tongqiao Granules+ CWMT

B, Danxi Yuping Feng Granules+ CWMT

C, Huaiqi Huang Granules+ CWMT

D, Tongqiao Biyan Granules+ CWMT

E, Xinqin Granules+ CWMT

F, Yuping Feng Granules+ CWMT

G , CWMT.

**Appendix 12:Summary of Adverse Events**

**Table S12.1:**Summary Table of Adverse Events by Organ System

| System Category | Specific Adverse Event | Combination Therapy Group (n) | CWMT Monotherapy Group (n) | Involving Chinese Patent Medicines | Severity/Outcome |
| --- | --- | --- | --- | --- | --- |
| Gastrointestinal System | Nausea, vomiting, and gastrointestinal discomfort | 16/5/14 | 32/5/29 | Biyuan Tongqiao Granules、Yuping Feng Granules、Tongqiao Biyan Granules、Biyuan Shu Oral Liquid、Sanfeng Tongqiao Dropping Pills、Xiangju Capsules | Mild、transient、resolved after drug withdrawal |
| Central nervous system | Headache, dizziness, somnolence, and insomnia | 20/7/19/0 | 45/11/40/1 | Biyuan Shu Oral Liquid、Biyuan Tongqiao Granules、Yuping Feng Granules、Tongqiao Biyan Granules、Buzhong Yiqi Pills、Xiangju Capsules、Sanfeng Tongqiao Dropping Pills | Mild、 well-tolerated |
| Cutaneous Reaction | Rash | 2 | 7 | Tongqiao Biyan Granules、Yuping Feng Granules | Mild、resolved after drug withdrawal |
| Local Nasal Reactions | Nasal dryness, epistaxis, and nasal irritation | 2/13/3 | 3/15/3 | Biyuan Tongqiao Granules、Tongqiao Biyan Granules、Xinqin Granules | Mild、transient |
| Metabolic Response | Facial flushing、dry mouth | 16/14 | 13/24 | Biyuan Shu Oral Liquid、Biyuan Tongqiao Granules、Yuping Feng Granules、Tongqiao Biyan Granules、Buzhong Yiqi Pills | Mild、well-tolerated |

**Appendix 13:Sensitivity Analysis**

**Table S13.1:**Sensitivity Analysis and SUCRA Values for Nasal Obstruction

| Treatment | Raw Data | | Sensitivity Analysis: Exclusion of Small-Sample Studies | | Sensitivity Analysis: Exclusion of High-Risk Studies | |
| --- | --- | --- | --- | --- | --- | --- |
|  | SMD | SUCRA | SMD | SUCRA | SMD | SUCRA |
| NCT+CWMT | -0.75(-3.43,1.93) | 42.1 | -0.75 (-3.62,2.12) | 41.2 | -0.75 (-3.88,2.38) | 40.2 |
| BYTG+CWMT | -1.14(-2.26,-0.02) | 51.8 | -1.14 (-2.31,0.04) | 51.7 | -1.14 (-2.55,0.27) | 49.3 |
| CBP+CWMT | -1.24(-3.19,0.71) | 54 | -1.24 (-3.29,0.80) | 53.4 | -1.24 (-3.48,0.99) | 52.1 |
| HG+CWMT | -1.10(-3.88,1.68) | 49.8 | NA | NA | NA | NA |
| STDP+CWMT | -1.21(-3.96,1.54) | 51.9 | -1.21 (-4.09,1.68) | 52.1 | -1.21 (-4.36,1.94) | 50 |
| TBG+CWMT | -1.79(-2.84,-0.74) | 72.3 | -1.84 (-3.03,-0.65) | 72.8 | -2.10 (-3.69,-0.51) | 75.3 |
| XG+CWMT | -0.72(-3.48,2.05) | 40.8 | -0.72 (-3.62,2.19) | 41.4 | NA | NA |
| YG+CWMT | -2.07(-4.85,0.71) | 72.1 | -2.07 (-4.98,0.84) | 72.5 | -2.07 (-5.25,1.10) | 69.1 |

Abbreviations: CWMT , Conventional Western Medicine Treatment ;

NCT ,Nasal Comfort Tablets ;

BYTG , Biyuan Tongqiao Granules ;

CBP , Cang'er Zibi Yan Pills ;

HG , Huaiqi Huang Granules ;

STDP , Sanfeng Tongqiao Dropping Pills ;

TBG , Tongqiao Biyan Granules ;

XG , Xinqin Granules ;

YG , Yuping Feng Granules .

**Table S13.2:**Sensitivity Analysis and SUCRA Values for Nasal pruritus

| Treatment | Raw Data | | Sensitivity Analysis: Exclusion of Small-Sample Studies | | Sensitivity Analysis: Exclusion of High-Risk Studies | |
| --- | --- | --- | --- | --- | --- | --- |
|  | SMD | SUCRA | SMD | SUCRA | SMD | SUCRA |
| NCT+CWMT | -0.52(-1.80,0.75) | 37.8 | -0.52 (-1.87,0.82) | 35.3 | -0.52 (-1.14,0.10) | 32.4 |
| BYTG+CWMT | -0.70(-1.46,0.05) | 45.3 | -0.70 (-1.49,0.09) | 42.3 | -0.34 (-0.82,0.14) | 22.1 |
| CBP+CWMT | -1.04(-1.98,-0.10) | 62.5 | -1.04 (-2.03,-0.05) | 60 | -1.01 (-1.52,-0.50) | 62.6 |
| HG+CWMT | -0.18(-1.55,1.20) | 25.1 | NA | NA | NA | NA |
| STDP+CWMT | -1.57(-2.90,-0.24) | 81.9 | -1.57 (-2.97,-0.18) | 80.1 | -1.57 (-2.29,-0.85) | 92.4 |
| TBG+CWMT | -1.20(-1.75,-0.65) | 71.8 | -1.13 (-1.76,-0.50) | 65.4 | -1.11 (-1.53,-0.69) | 69.1 |
| XG+CWMT | -0.80(-2.16,0.56) | 50.4 | -0.80 (-2.22,0.62) | 47.6 | NA | NA |
| YG+CWMT | -1.13(-2.48,0.21) | 64.7 | -1.13 (-2.55,0.28) | 62.3 | -1.13 (-1.89,-0.38) | 69.2 |

Abbreviations: CWMT , Conventional Western Medicine Treatment ;

NCT ,Nasal Comfort Tablets ;

BYTG , Biyuan Tongqiao Granules ;

CBP , Cang'er Zibi Yan Pills ;

HG , Huaiqi Huang Granules ;

STDP , Sanfeng Tongqiao Dropping Pills ;

TBG , Tongqiao Biyan Granules ;

XG , Xinqin Granules ;

YG , Yuping Feng Granules .

**Table S13.2:**Sensitivity Analysis and SUCRA Values for Paroxysmal sneezing

| Treatment | Raw Data | | Sensitivity Analysis: Exclusion of Small-Sample Studies | | Sensitivity Analysis: Exclusion of High-Risk Studies | |
| --- | --- | --- | --- | --- | --- | --- |
|  | SMD | SUCRA | SMD | SUCRA | SMD | SUCRA |
| NCT+CWMT | -0.68(-3.70,2.33) | 43.1 | -0.68 (-3.93,2.56) | 40.7 | -0.68 (-4.15,2.78) | 41.5 |
| BYTG+CWMT | -1.17(-2.55,0.22) | 54.8 | -1.17 (-2.62,0.29) | 52.6 | -1.09 (-2.83,0.65) | 49.9 |
| CBP+CWMT | -0.93(-3.12,1.26) | 47.6 | -0.93 (-3.24,1.38) | 46 | -0.93 (-3.40,1.53) | 46 |
| HG+CWMT | -0.43(-3.55,2.69) | 37.2 | NA | NA | NA | NA |
| STDP+CWMT | -1.41(-4.51,1.69) | 58.2 | -1.41 (-4.68,1.85) | 56.7 | -1.41 (-4.90,2.07) | 54.4 |
| TBG+CWMT | -2.09(-3.27,-0.91) | 78.8 | -2.07 (-3.41,-0.72) | 76.9 | -2.27 (-4.03,-0.52) | 78.4 |
| XG+CWMT | -0.90(-4.01,2.21) | 46.7 | -0.90 (-4.18,2.38) | 45.4 | NA | NA |
| YG+CWMT | -1.71(-4.83,1.40) | 63.8 | -1.71 (-5.00,1.57) | 63.3 | -1.71 (-5.21,1.79) | 61.9 |

Abbreviations: CWMT , Conventional Western Medicine Treatment ;

NCT ,Nasal Comfort Tablets ;

BYTG , Biyuan Tongqiao Granules ;

CBP , Cang'er Zibi Yan Pills ;

HG , Huaiqi Huang Granules ;

STDP , Sanfeng Tongqiao Dropping Pills ;

TBG , Tongqiao Biyan Granules ;

XG , Xinqin Granules ;

YG , Yuping Feng Granules .

**Table S13.2:**Sensitivity Analysis and SUCRA Values for Nasal discharge

| Treatment | Raw Data | | Sensitivity Analysis: Exclusion of Small-Sample Studies | | Sensitivity Analysis: Exclusion of High-Risk Studies | |
| --- | --- | --- | --- | --- | --- | --- |
|  | SMD | SUCRA | SMD | SUCRA | SMD | SUCRA |
| NCT+CWMT | -0.70(-3.81,2.42) | 47.9 | -0.70 (-4.05,2.65) | 46.4 | -0.70 (-4.18,2.78) | 44.5 |
| BYTG+CWMT | -0.94(-2.24,0.36) | 54.2 | -0.94 (-2.31,0.43) | 51.8 | -0.89 (-2.46,0.67) | 49.2 |
| CBP+CWMT | -1.01(-3.28,1.26) | 55.7 | -1.01 (-3.40,1.38) | 53.6 | -1.01 (-3.49,1.47) | 51.3 |
| HG+CWMT | -0.20(-3.42,3.03) | 37.6 | NA | NA | NA | NA |
| STDP+CWMT | -0.04(-3.23,3.16) | 33.7 | -0.04 (-3.40,3.33) | 32.3 | -0.04 (-3.53,3.46) | 32.5 |
| TBG+CWMT | -1.88(-3.20,-0.56) | 78.2 | -1.97 (-3.50,-0.45) | 78.2 | -2.46 (-4.22,-0.69) | 84.6 |
| XG+CWMT | -0.85(-4.07,2.36) | 49.7 | -0.85 (-4.24,2.54) | 49 | NA | NA |
| YG+CWMT | -1.71(-4.93,1.51) | 67.7 | -1.71 (-5.10,1.68) | 65.9 | -1.71 (-5.23,1.81) | 65.1 |

Abbreviations: CWMT , Conventional Western Medicine Treatment ;

NCT ,Nasal Comfort Tablets ;

BYTG , Biyuan Tongqiao Granules ;

CBP , Cang'er Zibi Yan Pills ;

HG , Huaiqi Huang Granules ;

STDP , Sanfeng Tongqiao Dropping Pills ;

TBG , Tongqiao Biyan Granules ;

XG , Xinqin Granules ;

YG , Yuping Feng Granules .

**Table S13.2:**Sensitivity Analysis and SUCRA Values for Effective rate

| Treatment | Raw Data | | Sensitivity Analysis: Exclusion of Small-Sample Studies | | Sensitivity Analysis: Exclusion of High-Risk Studies | |
| --- | --- | --- | --- | --- | --- | --- |
|  | RR | SUCRA | RR | SUCRA | RR | SUCRA |
| NCT+CWMT | 1.08(0.95,1.24) | 30.1 | 1.08 (0.95,1.24) | 31 | 1.08 (1.00,1.18) | 26 |
| BYSOL+CWMT | 1.16(0.97,1.39) | 53.2 | 1.16 (0.97,1.39) | 54.1 | 1.16 (1.00,1.34) | 56.1 |
| BYTG+CWMT | 1.15(1.08,1.23) | 49.5 | 1.15 (1.08,1.23) | 50.6 | 1.15 (1.08,1.21) | 50.5 |
| BZYQP+CWMT | 1.09(0.93,1.27) | 31.9 | 1.09 (0.93,1.27) | 33 | NA | NA |
| CBP+CWMT | 1.25(1.06,1.49) | 77.2 | 1.25 (1.06,1.49) | 79.4 | 1.24 (1.07,1.43) | 79.9 |
| DYG+CWMT | 1.15(0.96,1.37) | 50.3 | 1.15 (0.96,1.37) | 51.5 | NA | NA |
| HG+CWMT | 1.24(0.92,1.66) | 67.7 | NA | NA | NA | NA |
| LQG+CWMT | 1.15(0.94,1.41) | 51.7 | 1.15 (0.94,1.41) | 52.6 | 1.15 (0.97,1.37) | 53.1 |
| STDP+CWMT | 1.10(0.90,1.35) | 37.7 | 1.10 (0.90,1.35) | 38.1 | 1.10 (0.92,1.31) | 36.5 |
| TBG+CWMT | 1.17(1.09,1.25) | 56.9 | 1.18 (1.10,1.27) | 65.1 | 1.21 (1.10,1.32) | 75.2 |
| XC+CWMT | 1.18(1.05,1.31) | 59.6 | 1.18 (1.05,1.31) | 60.7 | 1.16 (1.01,1.33) | 55.9 |
| XG+CWMT | 1.21(1.07,1.36) | 68.4 | 1.21 (1.07,1.36) | 71 | 1.16 (1.03,1.30) | 56.7 |
| YG+CWMT | 1.18(1.11,1.24) | 60 | 1.17 (1.10,1.23) | 57.6 | 1.16 (1.09,1.23) | 57.3 |

Abbreviations: CWMT , Conventional Western Medicine Treatment ;

NCT ,Nasal Comfort Tablets ;

BYSOL , Biyuan Shu Oral Liquid;

BYTG , Biyuan Tongqiao Granules ;

BZYQP , Buzhong Yiqi Pills ;

CBP , Cang'er Zibi Yan Pills ;

DYG , Danxi Yuping Feng Granules ;

HG , Huaiqi Huang Granules ;

LQG , Lianhua Qingwen Granules ;

STDP , Sanfeng Tongqiao Dropping Pills ;

TBG , Tongqiao Biyan Granules ;

XC , Xiangju Capsules ;

XG , Xinqin Granules ;

YG , Yuping Feng Granules .

**Appendix 14:Data for regression analysis**

**Table S14.1:**Regression Analysis of Covariates (Age, Mechanism, Dosage Form, and CWMT) .The regression coefficients for all covariates had P values of ≥ 0.05, with 95% CIs crossing 0.

| **Factors** | Nasal obstruction | | | Nasal pruritus | | | Paroxysmal sneezing | | | Nasal discharge | | | Effective rate | | |
| --- | --- | --- | --- | --- | --- | --- | --- | --- | --- | --- | --- | --- | --- | --- | --- |
|  | Coefficient | 95%CI | P value | Coefficient | 95%CI | P value | Coefficient | 95%CI | P value | Coefficient | 95%CI | P value | Coefficient | 95%CI | P value |
| Age (years) | 0.04 | (-0.05, 0.13) | 0.28 | 0.03 | (-0.06, 0.12) | 0.41 | -0.02 | (-0.11, 0.07) | 0.63 | 0.05 | (-0.04, 0.14) | 0.24 | 0.08 | (-0.02, 0.14) | 0.13 |
| Mechanism | -0.06 | (-0.16, 0.04) | 0.24 | -0.04 | (-0.14, 0.06) | 0.45 | 0.03 | (-0.07, 0.13) | 0.55 | -0.05 | (-0.15, 0.05) | 0.31 | 0.13 | (0.01, 0.25) | 0.06 |
| Dosage form | 0.07 | (-0.03, 0.17) | 0.16 | 0.05 | (-0.05, 0.15) | 0.33 | 0.02 | (-0.08, 0.12) | 0.71 | 0.06 | (-0.04, 0.16) | 0.22 | -0.14 | (-0.24, 0.16) | 0.72 |
| CWMT | 0.05 | (-0.06, 0.16) | 0.38 | -0.03 | (-0.12, 0.06) | 0.47 | 0.04 | (-0.05, 0.13) | 0.42 | 0.02 | (-0.07, 0.11) | 0.64 | 0.07 | (-0.04, 0.18) | 0.29 |

Abbreviations: CWMT , Conventional Western Medicine Treatment.

**Appendix 15:Subgroup Analysis**

**Table S15.1:**Comparison of the effects of CPMs with different Treatment Course

| CPM | Treatment Course | Nasal obstruction | Nasal pruritus | Paroxysmal sneezing | Nasal discharge | Effective rate |
| --- | --- | --- | --- | --- | --- | --- |
| NCT+CWMT | ≤4 wk | -0.75 (-4.37,2.87) | -0.52 (-2.14,1.10) | -0.68 (-4.60,3.24) | -0.70 (-5.26,3.86) | 1.08 (0.98,1.20) |
|  | 6–8 wk | NA | NA | NA | NA | NA |
|  | ≥12 wk | NA | NA | NA | NA | NA |
| BYSOL+CWMT | ≤4 wk | NA | NA | NA | NA | 1.16 (0.99,1.36) |
|  | 6–8 wk | NA | NA | NA | NA | NA |
|  | ≥12 wk | NA | NA | NA | NA | NA |
| BYTG+CWMT | ≤4 wk | -0.82 (-2.92,1.27) | -0.74 (-1.90,0.42) | -0.82 (-3.09,1.45) | -0.68 (-3.31,1.96) | 1.13 (1.05,1.21) |
|  | 6–8 wk | -1.64 (-3.44,0.15) | -0.63 (-1.03,-0.22) | -1.68 (-3.61,0.24) | -1.38 (-3.18,0.42) | 1.20 (1.09,1.32) |
|  | ≥12 wk | NA | NA | NA | -0.84 (-1.19,-0.50) | 1.14 (0.97,1.35) |
| BZYQP+CWMT | ≤4 wk | NA | NA | NA | NA | NA |
|  | 6–8 wk | NA | NA | NA | NA | NA |
|  | ≥12 wk | NA | NA | NA | NA | 1.09 (0.97,1.22) |
| CBP+CWMT | ≤4 wk | NA | NA | NA | NA | NA |
|  | 6–8 wk | -1.57 (-4.15,1.01) | -1.27 (-1.83,-0.71) | -1.02 (-3.76,1.73) | -1.69 (-4.27,0.90) | 1.56 (1.08,2.26) |
|  | ≥12 wk | -0.93 (-1.29,-0.56) |  | -0.85 (-1.21,-0.49) | -0.34 (-0.68,-0.01) | 1.19 (1.02,1.39) |
| DYG+CWMT | ≤4 wk | NA | NA | NA | NA | NA |
|  | 6–8 wk | NA | NA | NA | NA | NA |
|  | ≥12 wk | NA | NA | NA | NA | NA |
| HG+CWMT | ≤4 wk | -1.10 (-4.76,2.56) | -0.17 (-1.87,1.52) | -0.43 (-4.39,3.52) | -0.20 (-4.78,4.39) | 1.24 (0.94,1.64) |
|  | 6–8 wk | NA | NA | NA | NA | NA |
|  | ≥12 wk | NA | NA | NA | NA | NA |
| LQG+CWMT | ≤4 wk | NA | NA | NA | NA | 1.15 (0.96,1.38) |
|  | 6–8 wk | NA | NA | NA | NA | NA |
|  | ≥12 wk | NA | NA | NA | NA | NA |
| STDP+CWMT | ≤4 wk | NA | -1.57 (-3.23,0.09) | -1.41 (-5.35,2.53) | -0.04 (-4.60,4.53) | 1.10 (0.91,1.32) |
|  | 6–8 wk | NA | NA | NA | NA | NA |
|  | ≥12 wk | NA | NA | NA | NA | NA |
| TBG+CWMT | ≤4 wk | NA | -1.25 (-2.00,-0.50) | -2.34 (-4.11,-0.56) | -1.95 (-4.25,0.35) | 1.14 (1.07,1.22) |
|  | 6–8 wk | -0.72 (-3.27,1.83) | -0.95 (-1.42,-0.48) | -1.03 (-3.77,1.70) | -1.30 (-3.87,1.26) | 1.27 (1.04,1.54) |
|  | ≥12 wk | -1.92 (-2.45,-1.40) |  | -2.03 (-2.56,-1.50) | -2.32 (-2.88,-1.76) | 1.22 (1.01,1.47) |
| XC+CWMT | ≤4 wk | NA | NA | NA | NA | 1.17 (1.07,1.29) |
|  | 6–8 wk | NA | NA | NA | NA | NA |
|  | ≥12 wk | NA | NA | NA | NA | NA |
| XG+CWMT | ≤4 wk | -0.72 (-4.37,2.93) | -0.80 (-2.48,0.88) | -0.90 (-4.85,3.05) | -0.85 (-5.43,3.73) | 1.19 (1.06,1.34) |
|  | 6–8 wk | NA | NA | NA | NA | 1.22 (1.00,1.49) |
|  | ≥12 wk | NA | NA | NA | NA | NA |
| YG+CWMT | ≤4 wk | -2.07 (-5.73,1.59) | -1.13 (-2.81,0.54) | -1.71 (-5.66,2.23) | -1.71 (-6.29,2.87) | 1.20 (1.14,1.26) |
|  | 6–8 wk | NA | NA | NA | NA | NA |
|  | ≥12 wk | NA | NA | NA | NA | 1.02 (0.97,1.06) |

Abbreviations: CPM,Chinese Patent Medicine ;

wk , week ;

CWMT , Conventional Western Medicine Treatment ;

NCT ,Nasal Comfort Tablets ;

BYSOL , Biyuan Shu Oral Liquid;

BYTG , Biyuan Tongqiao Granules ;

BZYQP , Buzhong Yiqi Pills ;

CBP , Cang'er Zibi Yan Pills ;

DYG , Danxi Yuping Feng Granules ;

HG , Huaiqi Huang Granules ;

LQG , Lianhua Qingwen Granules ;

STDP , Sanfeng Tongqiao Dropping Pills ;

TBG , Tongqiao Biyan Granules ;

XC , Xiangju Capsules ;

XG , Xinqin Granules ;

YG , Yuping Feng Granules .

**Figure S15.1:Forest Plots of Network Meta-Analysis Comparing Various Interventions versus Control Group for Allergic Rhinitis in Children by TCM Syndrome Differentiation**

**Figure S15.1.1:**Forest plots of interventions for the External Pathogen Invading the Lung syndrome versus control group.

**Figure S15.1.1a:**Forest Plot for Nasal obstruction: Interventions for External Pathogen Invading the Lung Syndrome versus Control.Results are expressed as SMD and 95% CI, derived from a random-effects model.An SMD < 0 favors the intervention, indicating a reduction in symptom scores compared to the control. The size of the data markers corresponds to the relative weight of each comparison in the network.


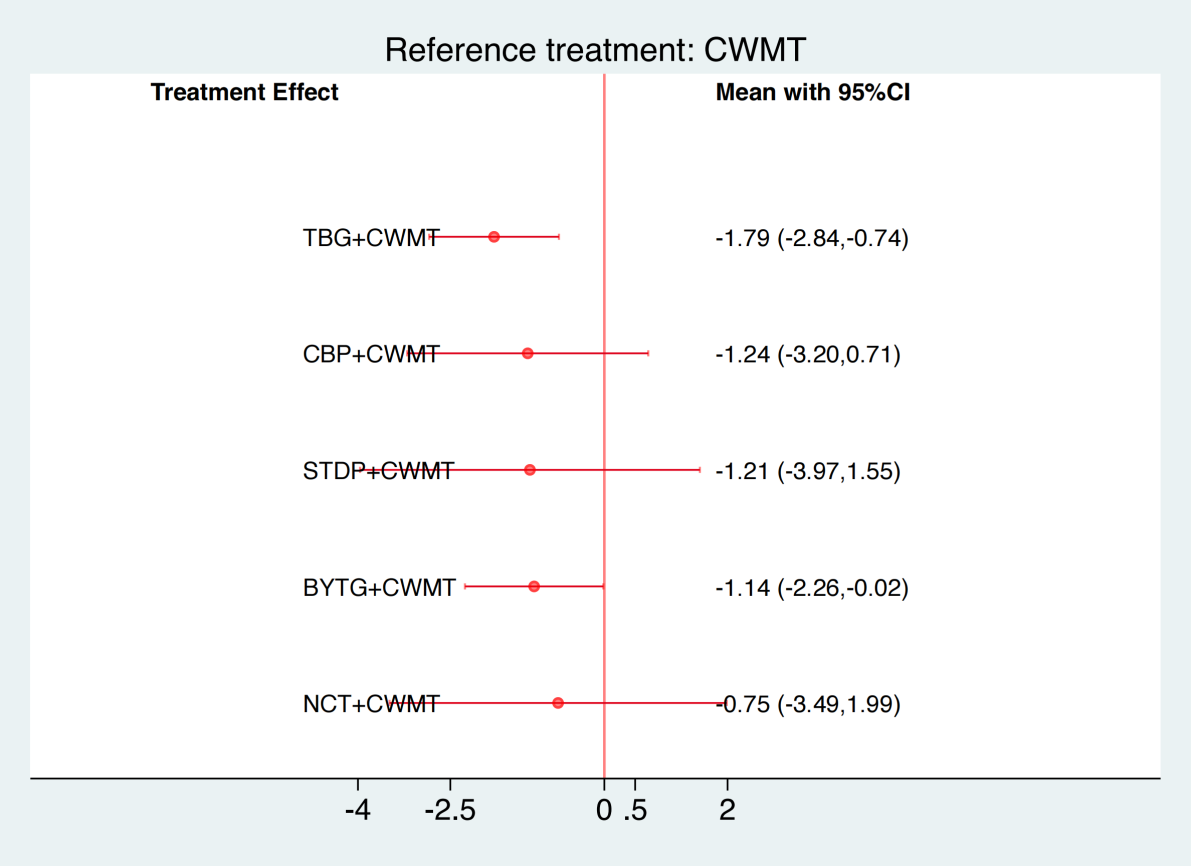


Abbreviations: TCM , Traditional Chinese Medicine ;

SMD, Standardized Mean Difference ;

CI, confidence intervals ;

CWMT , Conventional Western Medicine Treatment ;

NCT ,Nasal Comfort Tablets ;

BYTG , Biyuan Tongqiao Granules ;

CBP , Cang'er Zibi Yan Pills ;

STDP , Sanfeng Tongqiao Dropping Pills ;

TBG , Tongqiao Biyan Granules .

**Figure S15.1.1b:** Forest Plot for Nasal pruritus: Interventions for External Pathogen Invading the Lung Syndrome versus Control.Results are expressed as SMD and 95% CI, derived from a random-effects model.An SMD < 0 favors the intervention, indicating a reduction in symptom scores compared to the control. The size of the data markers corresponds to the relative weight of each comparison in the network.


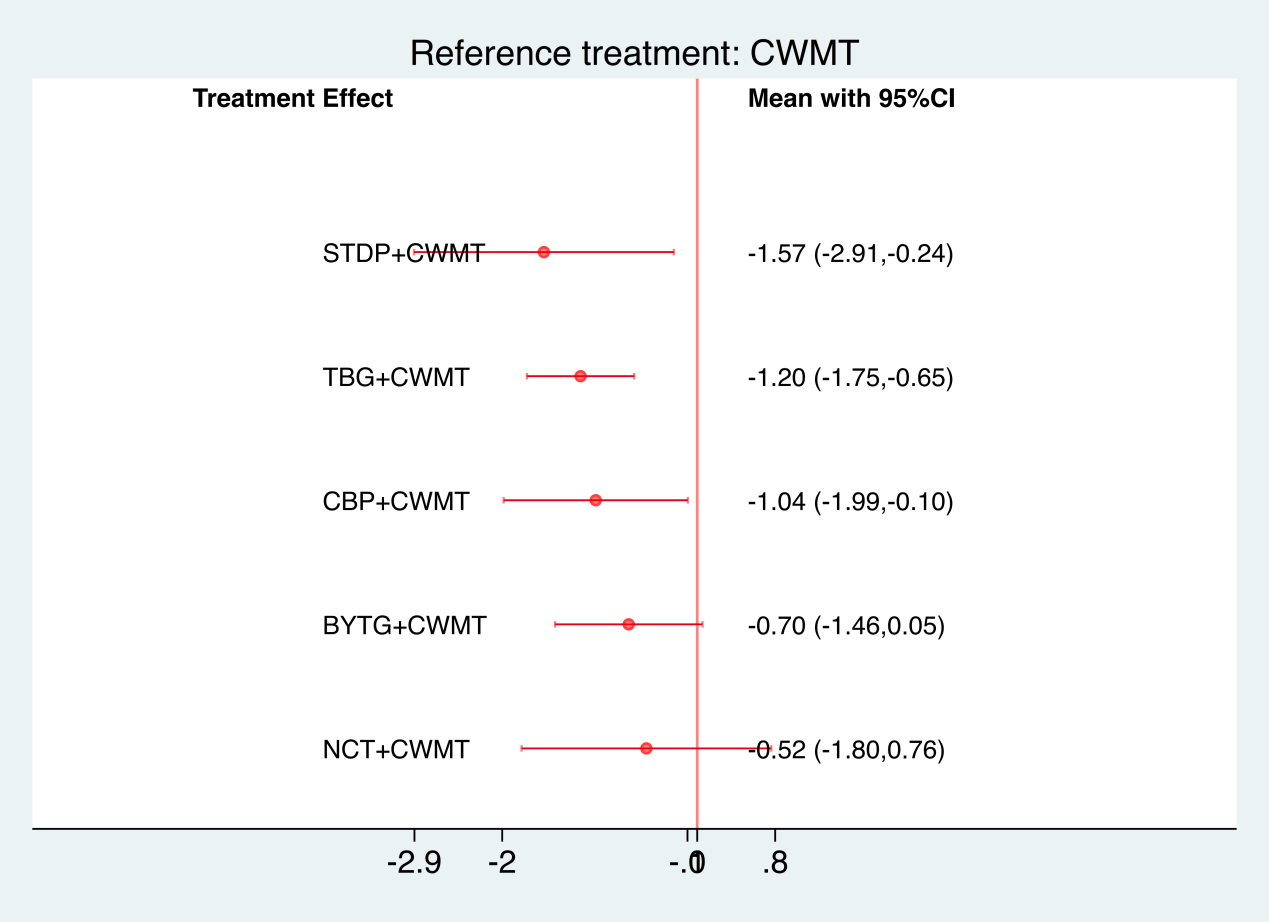


Abbreviations: SMD, Standardized Mean Difference ;

CI, confidence intervals ;

CWMT , Conventional Western Medicine Treatment ;

NCT ,Nasal Comfort Tablets ;

BYTG , Biyuan Tongqiao Granules ;

CBP , Cang'er Zibi Yan Pills ;

STDP , Sanfeng Tongqiao Dropping Pills ;

TBG , Tongqiao Biyan Granules .

**Figure S15.1.1c:** Forest Plot for Paroxysmal sneezing: Interventions for External Pathogen Invading the Lung Syndrome versus Control.Results are expressed as SMD and 95% CI, derived from a random-effects model.An SMD < 0 favors the intervention, indicating a reduction in symptom scores compared to the control. The size of the data markers corresponds to the relative weight of each comparison in the network.


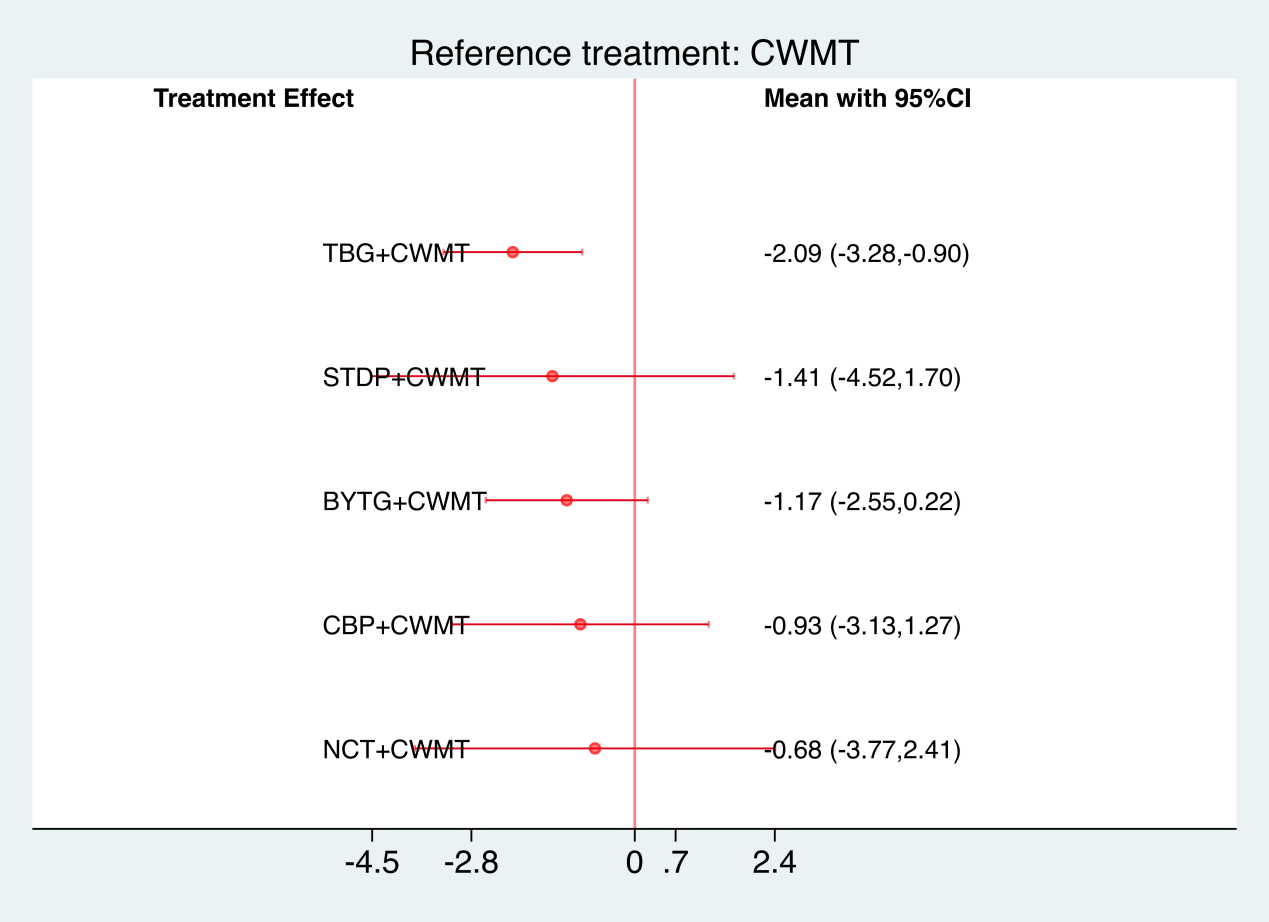


Abbreviations: SMD, Standardized Mean Difference ;

CI, confidence intervals ;

CWMT , Conventional Western Medicine Treatment ;

NCT ,Nasal Comfort Tablets ;

BYTG , Biyuan Tongqiao Granules ;

CBP , Cang'er Zibi Yan Pills ;

STDP , Sanfeng Tongqiao Dropping Pills ;

TBG , Tongqiao Biyan Granules .

**Figure S15.1.1d:** Forest Plot for Nasal discharge: Interventions for External Pathogen Invading the Lung Syndrome versus Control.Results are expressed as SMD and 95% CI, derived from a random-effects model.An SMD < 0 favors the intervention, indicating a reduction in symptom scores compared to the control. The size of the data markers corresponds to the relative weight of each comparison in the network.


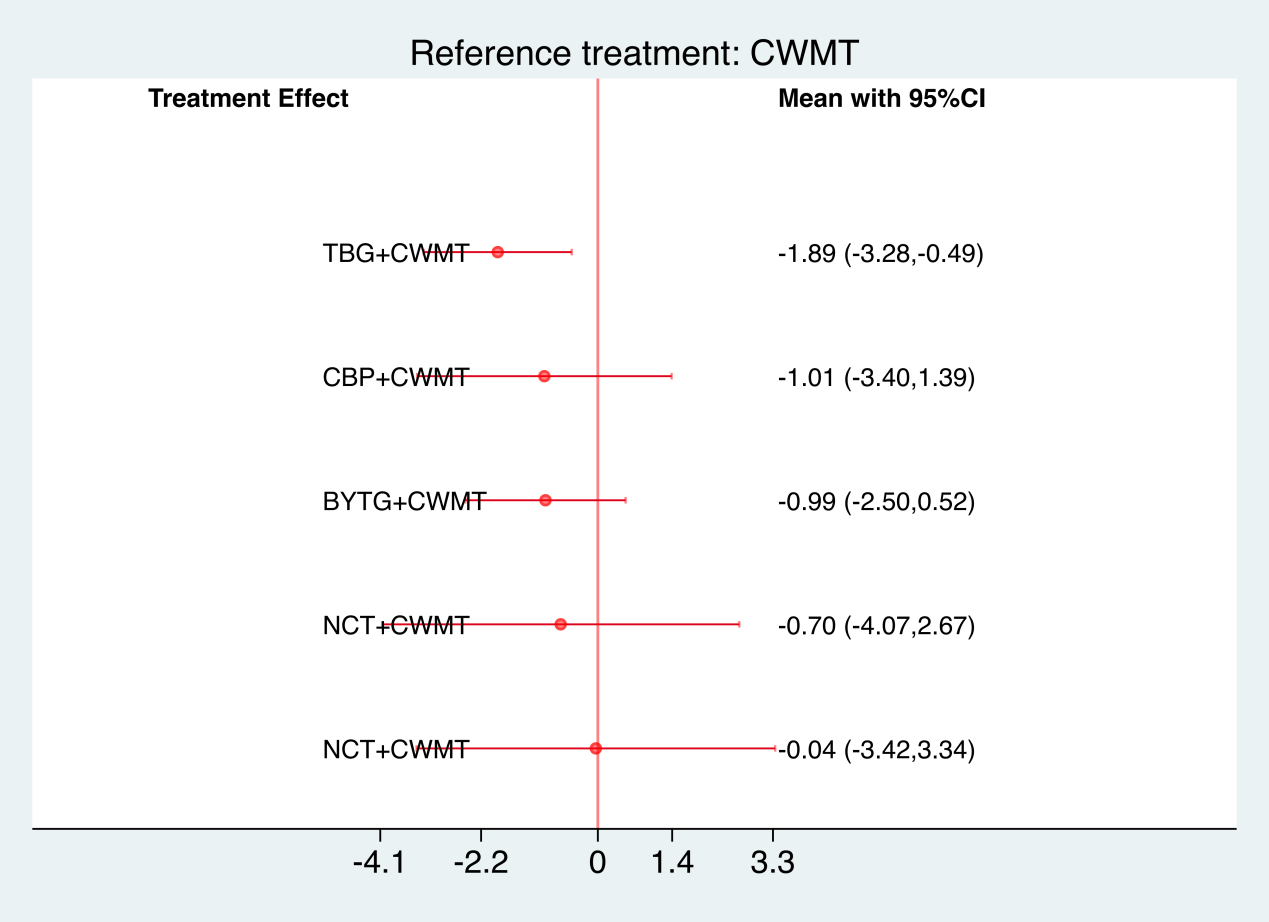


Abbreviations: SMD, Standardized Mean Difference ;

CI, confidence intervals ;

CWMT , Conventional Western Medicine Treatment ;

NCT ,Nasal Comfort Tablets ;

BYTG , Biyuan Tongqiao Granules ;

CBP , Cang'er Zibi Yan Pills ;

STDP , Sanfeng Tongqiao Dropping Pills ;

TBG , Tongqiao Biyan Granules .

**Figure S15.1.1e:**Forest Plot for Effective rate: Interventions for External Pathogen Invading the Lung Syndrome versus Control.Results are expressed as RR and 95% CI, derived from a random-effects model.An RR > 1 favors the intervention, indicating a higher response rate compared to the control. The size of the data markers corresponds to the relative weight of each comparison in the network.


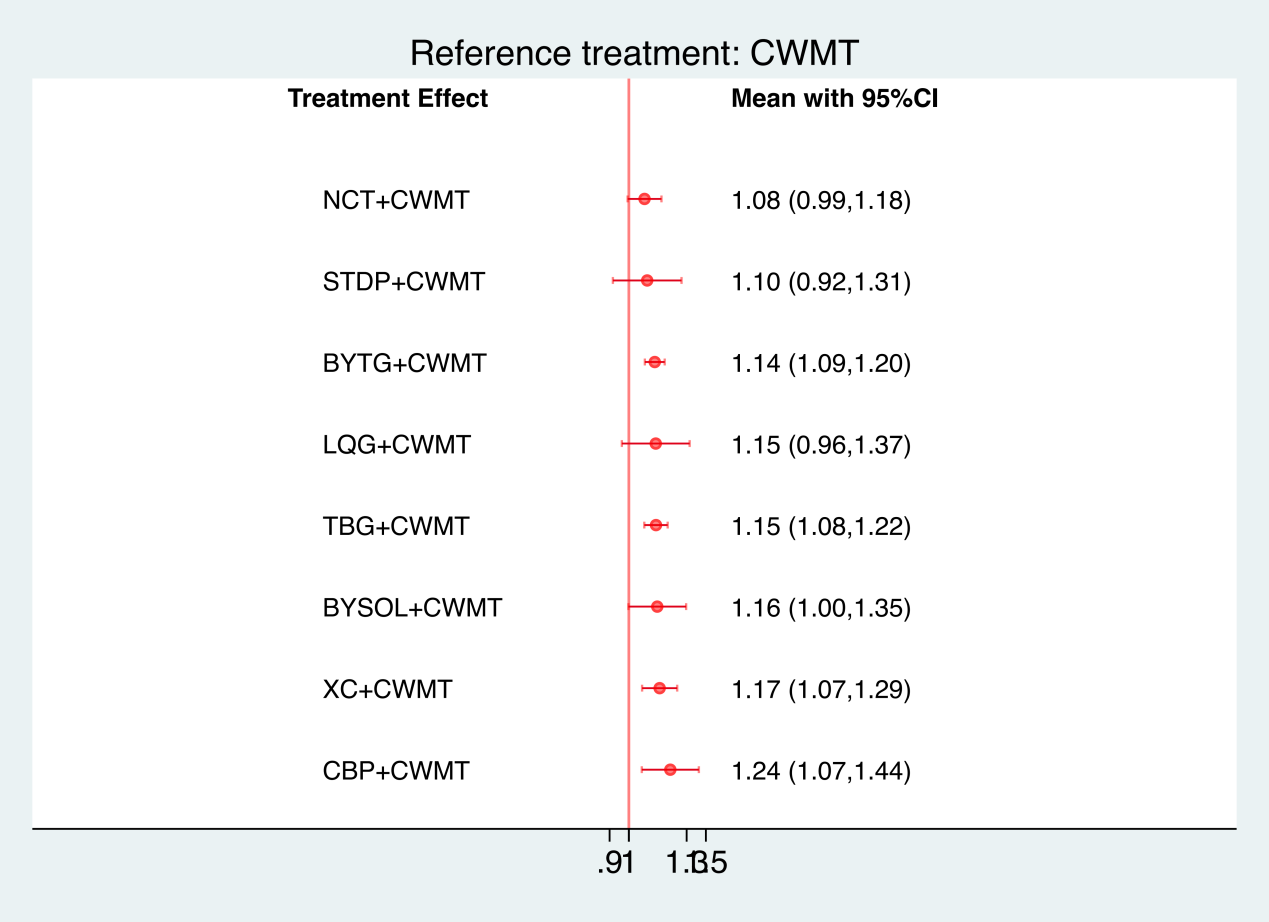


Abbreviations: RR, Risk Ratio ;

CI, confidence intervals ;

CWMT , Conventional Western Medicine Treatment ;

NCT ,Nasal Comfort Tablets ;

BYTG , Biyuan Tongqiao Granules ;

CBP , Cang'er Zibi Yan Pills ;

STDP , Sanfeng Tongqiao Dropping Pills ;

TBG , Tongqiao Biyan Granules .

**Figure S15.1.2:**Forest plots of interventions for the Wei Qi Deficiency and Instability syndrome versus control group.

**Figure S15.1.2a:** Forest Plot for Effective rate: Interventions for Wei Qi Deficiency and Instability Syndrome versus Control.Results are expressed as RR and 95% CI, derived from a random-effects model.An RR > 1 favors the intervention, indicating a higher response rate compared to the control. The size of the data markers corresponds to the relative weight of each comparison in the network.


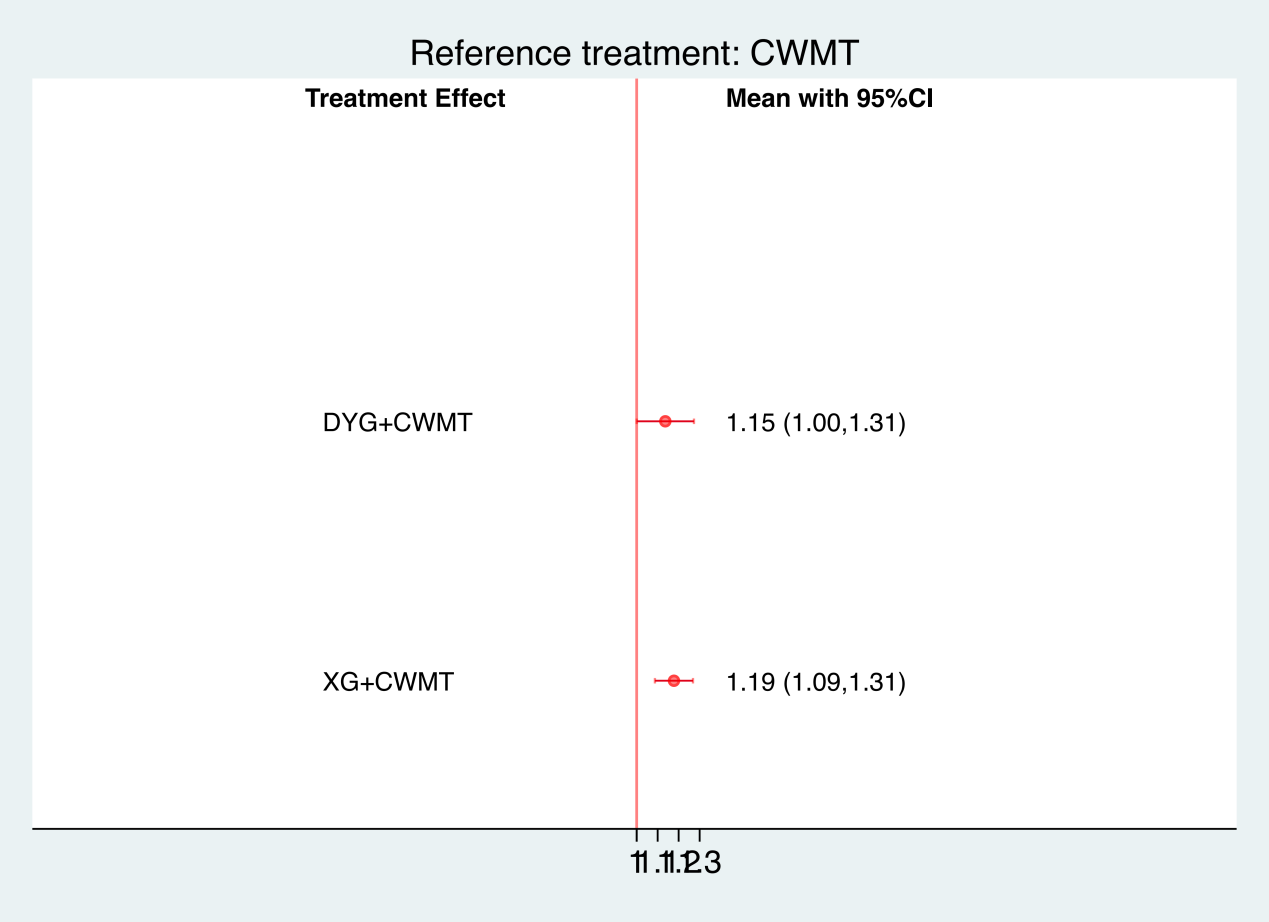


Abbreviations: RR, Risk Ratio ;

CI, confidence intervals ;

CWMT , Conventional Western Medicine Treatment ;

DYG , Danxi Yuping Feng Granules ;

XG , Xinqin Granules .

**Figure S15.1.3:**Forest plots of interventions for the Deficiency of Vital Qi syndrome versus control group.

**Figure S15.1.3a:** Forest Plot for Nasal obstruction: Interventions for Deficiency of Vital Qi Syndrome versus Control.Results are expressed as SMD and 95% CI, derived from a random-effects model.An SMD < 0 favors the intervention, indicating a reduction in symptom scores compared to the control. The size of the data markers corresponds to the relative weight of each comparison in the network.


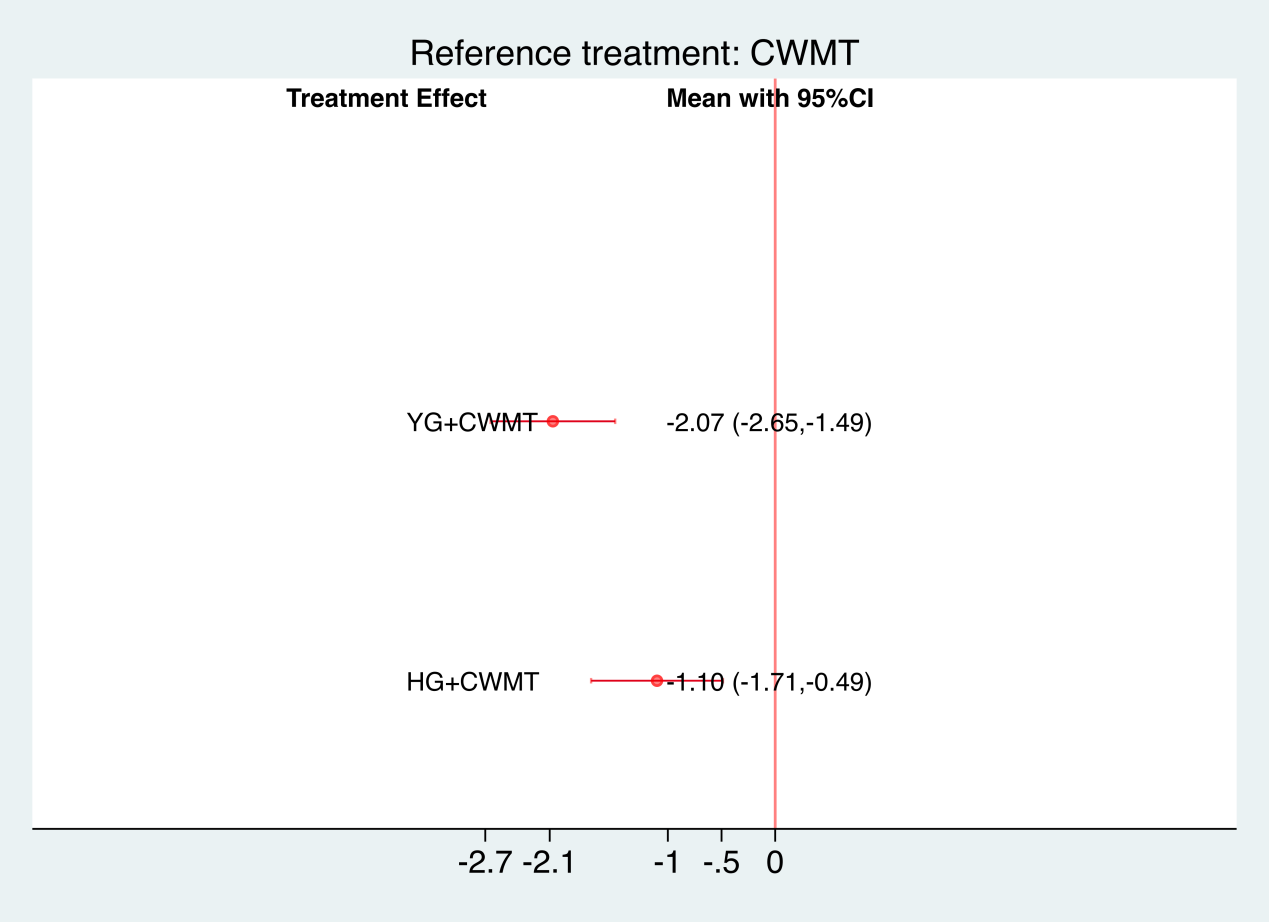


Abbreviations: SMD, Standardized Mean Difference ;

CI, confidence intervals ;

CWMT , Conventional Western Medicine Treatment ;

HG , Huaiqi Huang Granules ;

YG , Yuping Feng Granules .

**Figure S15.1.3b:** Forest Plot for Nasal pruritus: Interventions for Deficiency of Vital Qi Syndrome versus Control.Results are expressed as SMD and 95% CI, derived from a random-effects model.An SMD < 0 favors the intervention, indicating a reduction in symptom scores compared to the control. The size of the data markers corresponds to the relative weight of each comparison in the network.


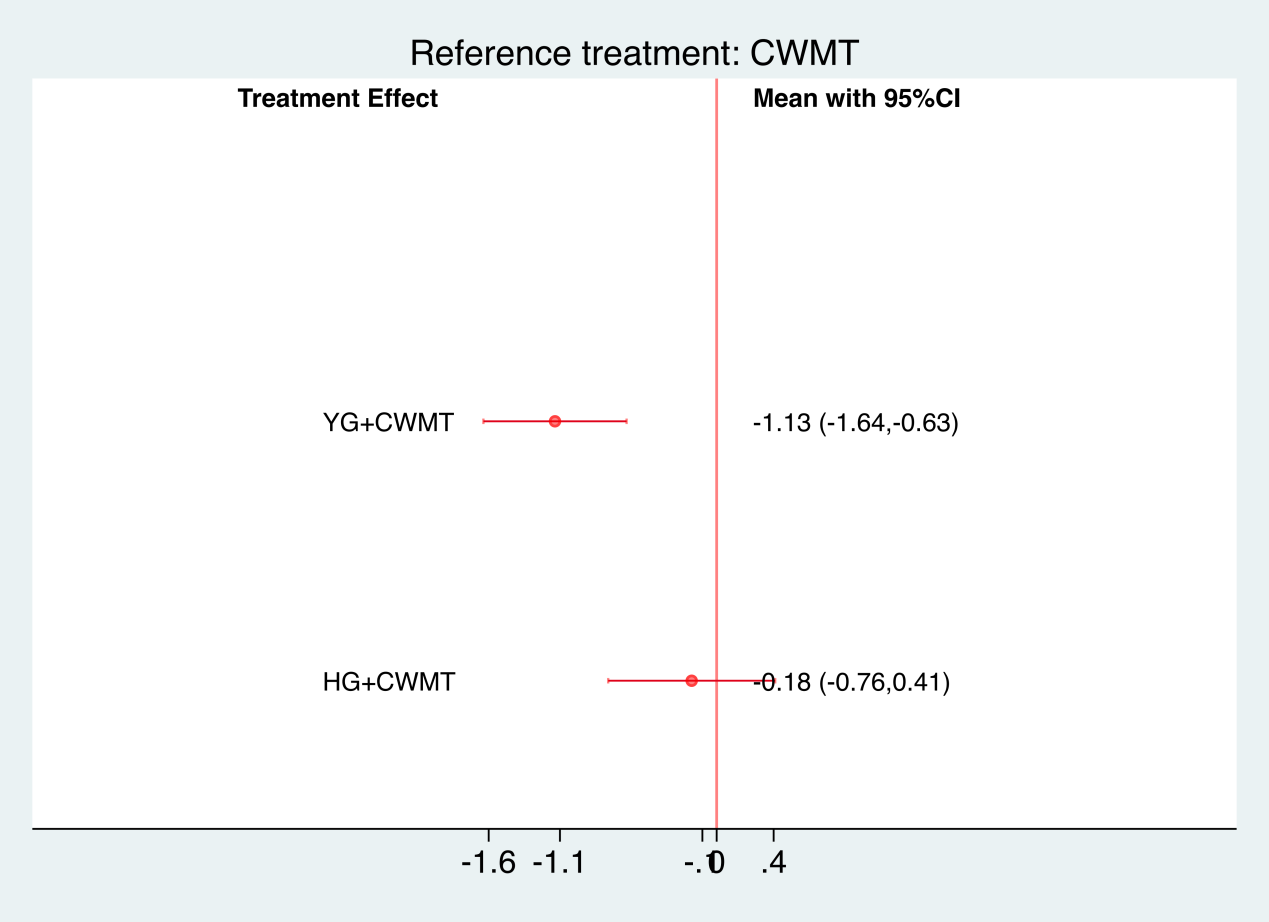


Abbreviations: SMD, Standardized Mean Difference ;

CI, confidence intervals ;

CWMT , Conventional Western Medicine Treatment ;

HG , Huaiqi Huang Granules ;

YG , Yuping Feng Granules .

**Figure S15.1.3c:** Forest Plot for Paroxysmal sneezing: Interventions for Deficiency of Vital Qi Syndrome versus Control.Results are expressed as SMD and 95% CI, derived from a random-effects model.An SMD < 0 favors the intervention, indicating a reduction in symptom scores compared to the control. The size of the data markers corresponds to the relative weight of each comparison in the network.


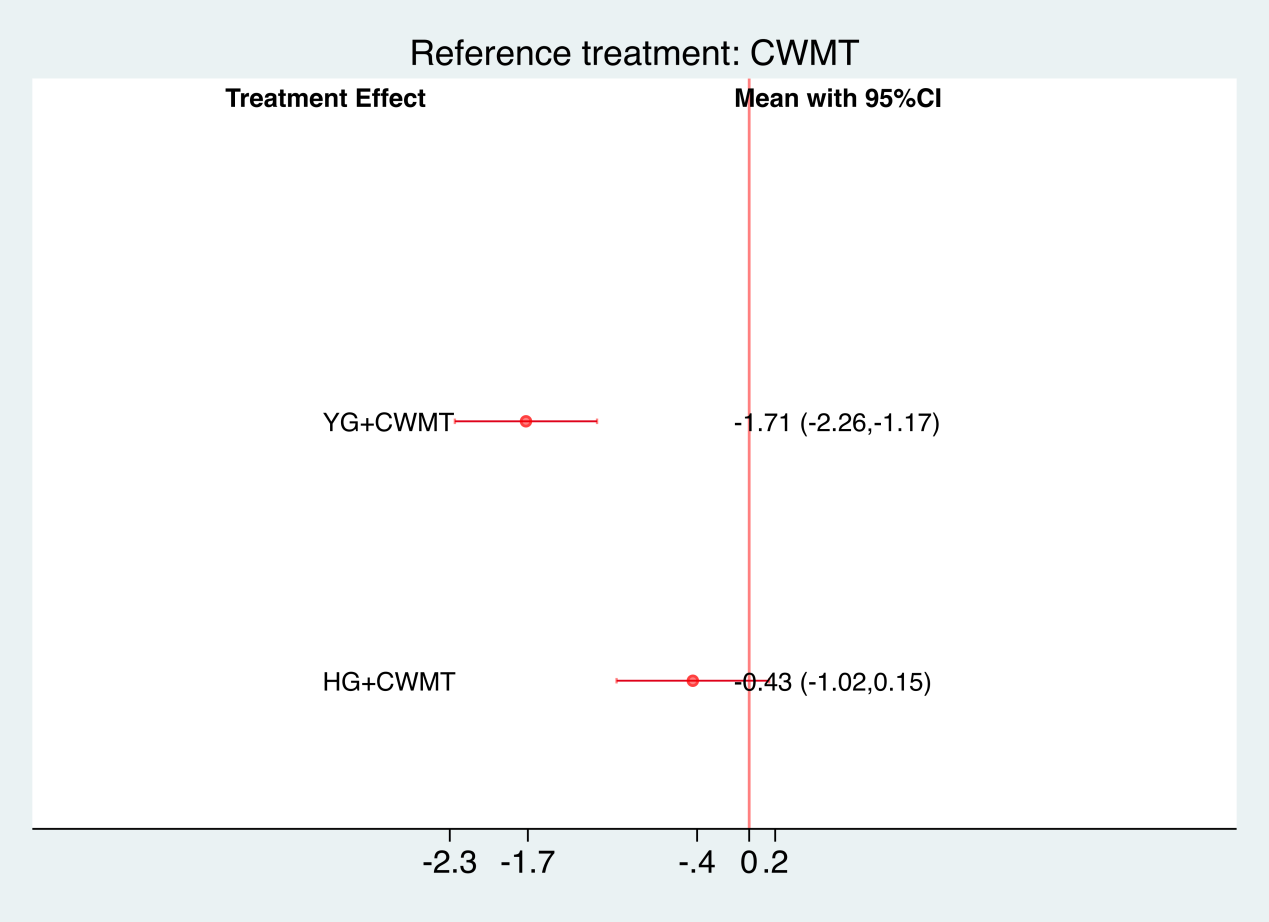


Abbreviations: SMD, Standardized Mean Difference ;

CI, confidence intervals ;

CWMT , Conventional Western Medicine Treatment ;

HG , Huaiqi Huang Granules ;

YG , Yuping Feng Granules .

**Figure S15.1.3d:** Forest Plot for Nasal discharge: Interventions for Deficiency of Vital Qi Syndrome versus Control.Results are expressed as SMD and 95% CI, derived from a random-effects model.An SMD < 0 favors the intervention, indicating a reduction in symptom scores compared to the control. The size of the data markers corresponds to the relative weight of each comparison in the network.


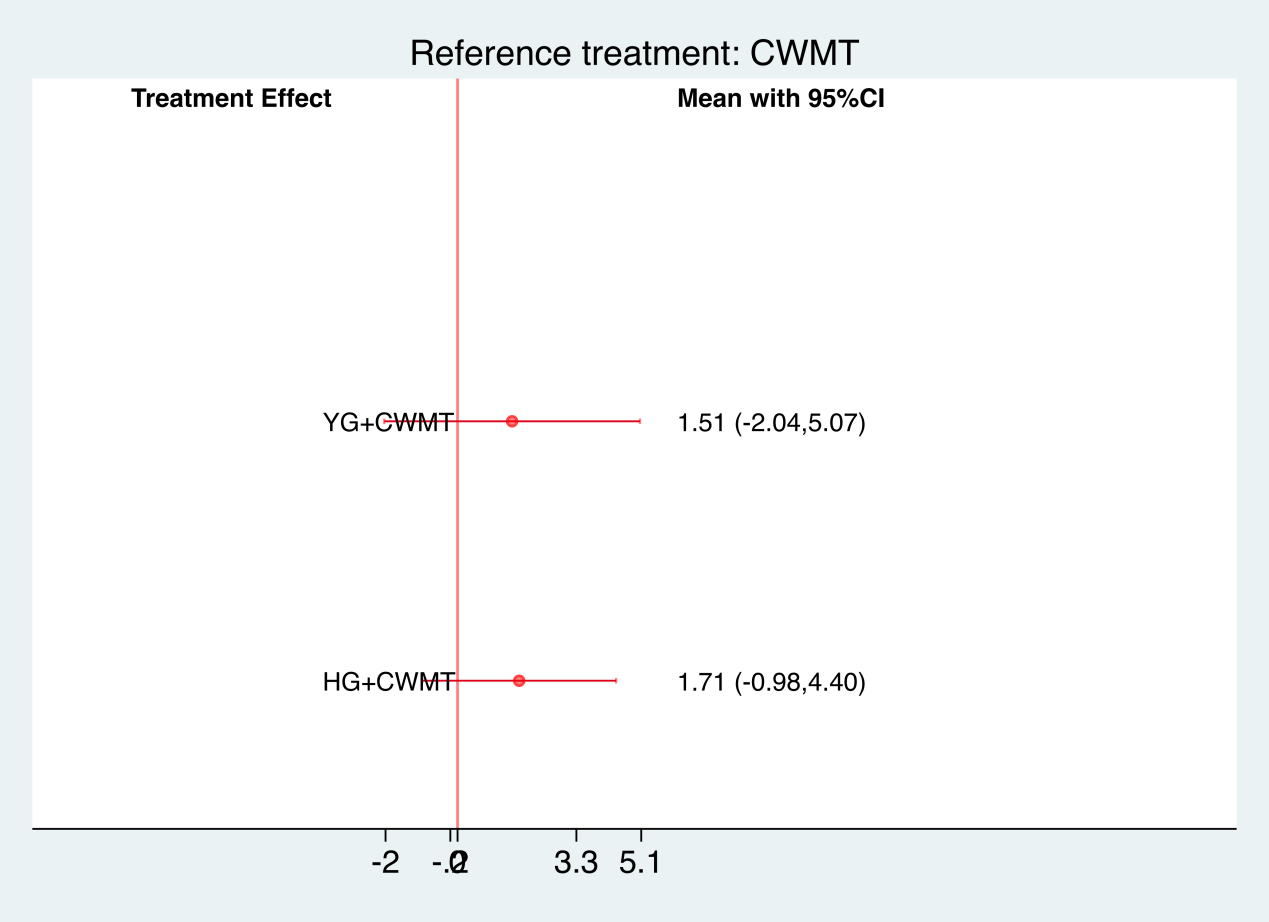


Abbreviations: SMD, Standardized Mean Difference ;

CI, confidence intervals ;

CWMT , Conventional Western Medicine Treatment ;

HG , Huaiqi Huang Granules ;

YG , Yuping Feng Granules .

**Figure S15.1.3e:**Effective rate Forest Plot for Effective rate: Interventions for Deficiency of Vital Qi Syndrome versus Control.Results are expressed as RR and 95% CI, derived from a random-effects model.An RR > 1 favors the intervention, indicating a higher response rate compared to the control. The size of the data markers corresponds to the relative weight of each comparison in the network.


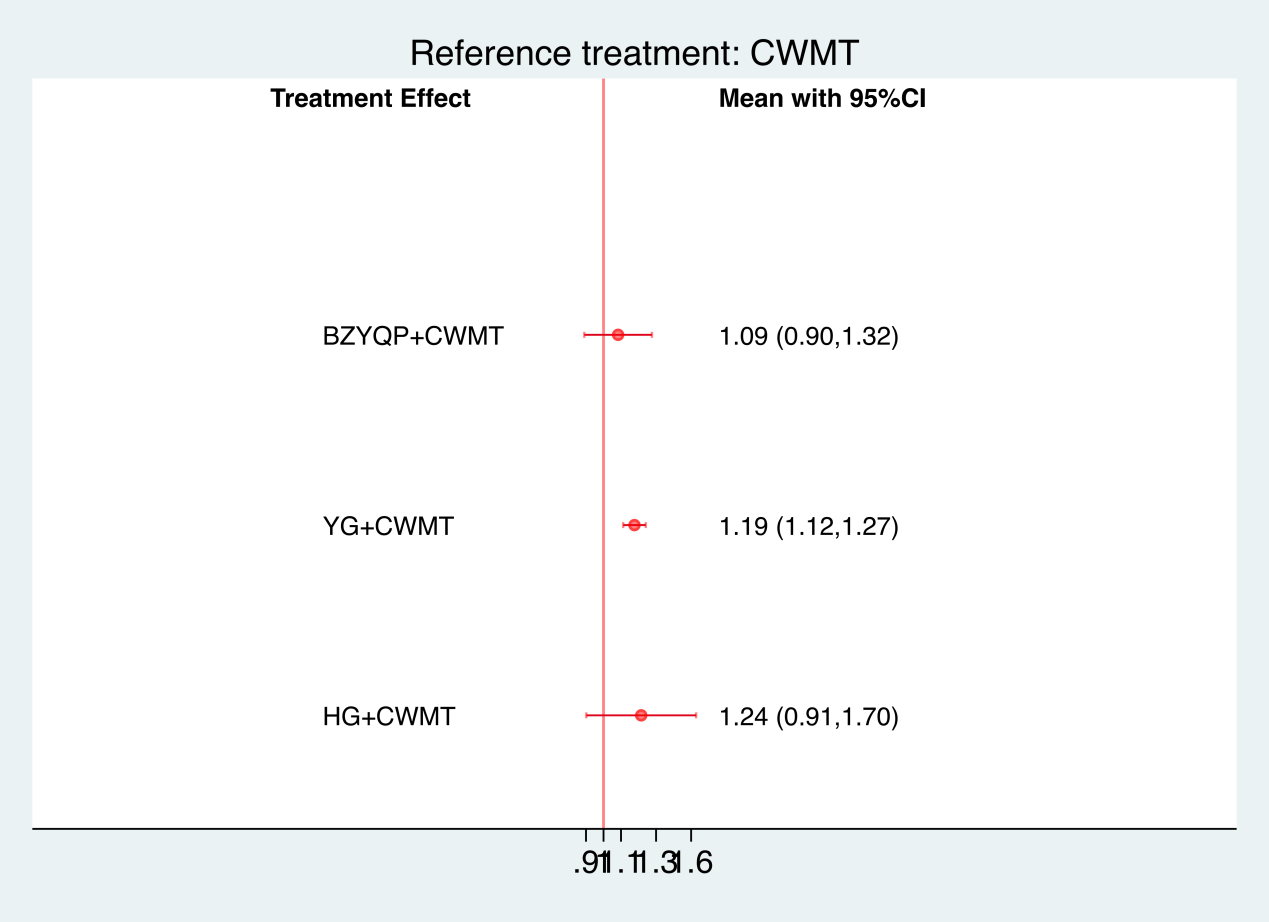


Abbreviations: SMD, Standardized Mean Difference ;

CI, confidence intervals ;

CWMT , Conventional Western Medicine Treatment ;

BZYQP , Buzhong Yiqi Pills ;

HG , Huaiqi Huang Granules ;

YG , Yuping Feng Granules .
